# Supplementary material for: Design, Synthesis, Antifungal Evaluation, Structure–Activity Relationship (SAR) Study, and Molecular Docking of Novel Spirotryprostatin A Derivatives
Source: Molecules. 2024 Feb 15;29(4):864. doi: 10.3390/molecules29040864 (PMC11154411; doi:10.3390/molecules29040864)
Supplement: Supplementary file 1 [file molecules-29-00864-s001.zip › molecules-2856196-supplementary.pdf]

Design, Synthesis, Antifungal Evaluation, Structure–Activity Relationship (SAR) Study, and Molecular Docking of Novel Spirotryprostatin A Derivatives

Yang-Min Ma *, Xia Miao, Bin Jia, Zhao-Yang Sun, Si-Yue Ma and Cong Yan

Key Laboratory of Chemical Additives for China National Light Industry, College of Chemistry and Chemical Engineering, Shaanxi University of Science and Technology, Xi'an 710021, China; 200811042@sust.edu.cn (X.M.); jiabin@sust.edu.cn (B.J.); 210811009@sust.edu.cn (Z.-Y.S.); masiyue@sust.edu.cn (S.-Y.M.); 230812183@sust.edu.cn (C.Y.)

* Correspondence: mym63@sina.com

CONTENT

1. Molecular Docking S1

2. Melt points and HRMS data spectra S3

3. NMR data of compounds S5

4. Spectrums of azomethine ylide **1a-1s** S30

5. Spectrums of 2-oxoindolin-3-ylidene bodies **2a-2g** S51

6. Spectrums of spirooxindole intermediates **3a-3x** S59

7. Spectrums of target compounds **4a-4s** S110

8. Crystallographic data S148

1. Molecular Docking

Table S1 Molecular docking data of compound **3a, 3b, 3d, 3k** and **3q**.

Compound	3a	3b	3d	3k	3q
ΔG_b(kcal/mol)	-7.4	-7.29	-7.28	-7.64	-7.62

Table S2 Molecular docking data of compound **4a, 4d, 4k** and **4q**.

CompD.	ΔG_b(kcal/mol)	Hydrophobic interactions		Hydrogen bonds		Halogen bonds		ClogP
		Residue	AA	Residue	AA	Residue	AA	
4a	-8.93	30C	ILE	91D	TYR	N/A		3.3239
		35C	TRP					
		43C	ILE					
		46C	ARG					
		46C	ARG					
		50C	ILE	173B	TRP			
		91D	TYR					
		173B	TRP					
		218B	ILE					
4d	-9.36	30C	ILE	91D	TYR	N/A		3.4669
		35C	TRP					
		35C	TRP					
		43C	ILE					
		46C	ARG					
		50C	ILE	173B	TRP			

		91D	TYR					
		169B	PRO					
		172B	TRP					
		218B	ILE					
		30C	ILE					
		35C	TRP					
		43C	ILE	91D	TYR			
		46C	ARG					
4k	-9.42	46C	ARG			39C	MET	4.2069
		50C	ILE					
		172B	TRP	173B	TRP			
		173B	TRP					
		218B	ILE					
		30C	ILE					
		35C	TRP					
		46C	ARG	91D	TYR			
		46C	ARG					
4q	-9.16	50C	ILE			N/A		3.2429
		91D	TYR					
		172B	TRP	173B	TRP			
		173B	TRP					
		218B	ILE					

2. Melt points and HRMS data spectra

Table S3 Melt points data of compounds **2a-2g**

Compd.	Formula	m.p./℃
**2a**	$C_{15}H_{11}NO$	170-172
**2b**	$C_{15}H_{10}BrNO$	180-183
**2c**	$C_{16}H_{13}NO_2$	214-216
**2d**	$C_{12}H_{11}NO$	148-150
**2e**	$C_{13}H_{13}NO$	119-121
**2f**	$C_{14}H_{15}NO$	127-130
**2g**	$C_{15}H_{17}NO$	150-152

Table S4 Melt points and HRMS data of compounds **3a-3z**

Compd.	Formula	m.p./℃	HRMS(Calcd), m/z
**3a**	$C_{25}H_{22}N_2O_3$	181-183	399.17029[a]
**3b**	$C_{25}H_{21}FN_2O_3$	375-377	417.16130[a]
**3c**	$C_{25}H_{21}FN_2O_3$	189-192	417.16105[a]
**3d**	$C_{25}H_{21}FN_2O_3$	165-168	417.16045[a]
**3e**	$C_{25}H_{21}ClN_2O_3$	188-191	433.13242[a]
**3f**	$C_{25}H_{21}ClN_2O_3$	158-161	433.13214[a]
**3g**	$C_{25}H_{21}ClN_2O_3$	180-183	433.13203[a]
**3h**	$C_{25}H_{21}BrN_2O_3$	195-197	477.08041[a]
**3i**	$C_{25}H_{21}BrN_2O_3$	143-145	477.08144[a]
**3j**	$C_{25}H_{21}BrN_2O_3$	189-191	477.08105[a]
**3k**	$C_{26}H_{21}F_3N_2O_3$	165-167	467.40047[a]
**3l**	$C_{26}H_{21}F_3N_2O_3$	166-168	467.15780[a]
**3m**	$C_{26}H_{21}F_3N_2O_3$	177-180	467.15715[a]
**3n**	$C_{25}H_{21}N_3O_5$	190-193	444.15518[a]
**3o**	$C_{25}H_{21}N_3O_5$	148-150	444.15484[a]
**3p**	$C_{25}H_{21}N_3O_5$	188-190	444.15397[a]
**3q**	$C_{26}H_{24}N_2O_4$	211-213	429.17924[a]
**3r**	$C_{26}H_{24}N_2O_4$	115-118	429.18090[a]
**3s**	$C_{26}H_{24}N_2O_4$	115-117	429.18148[a]
**3u**	$C_{25}H_{21}BrN_2O_3$	192-194	477.08217[a]
**3v**	$C_{26}H_{24}N_2O_4$	207-210	429.18141[a]
**3w**	$C_{22}H_{22}N_2O_3$	189-191	363.17108[a]

**3x**	$C_{23}H_{24}N_2O_3$	200-202	377.18645[a]
**3y**	$C_{24}H_{26}N_2O_3$	207-209	391.2029[a]
**3z**	$C_{25}H_{28}N_2O_3$	215-217	405.2290[a]

[a] HRMS for compounds are $[M+H]^+$

Table S5 Melt points and HRMS data of compounds **4a-4s**

Compd.	Formula	m.p./℃	HRMS(Calcd), m/z
**4a**	$C_{29}H_{25}N_3O_3$	175-178	486.1783[a]
**4b**	$C_{29}H_{24}FN_3O_3$	180-182	482.18801[b]
**4c**	$C_{29}H_{24}FN_3O_3$	156-158	482.18599[b]
**4d**	$C_{29}H_{24}FN_3O_3$	179-182	482.18656[b]
**4e**	$C_{29}H_{24}ClN_3O_3$	188-190	498.15868[b]
**4f**	$C_{29}H_{24}ClN_3O_3$	175-177	498.15932[b]
**4g**	$C_{29}H_{24}ClN_3O_3$	200-202	498.15844[b]
**4h**	$C_{29}H_{24}BrN_3O_3$	167-170	542.10605[b]
**4i**	$C_{29}H_{24}BrN_3O_3$	183-185	542.26679[b]
**4j**	$C_{29}H_{24}BrN_3O_3$	191-193	542.10791[b]
**4k**	$C_{30}H_{24}F_3N_3O_3$	166-168	532.18523[b]
**4l**	$C_{30}H_{24}F_3N_3O_3$	195-198	532.18479[b]
**4m**	$C_{30}H_{24}F_3N_3O_3$	201-203	532.18582[b]
**4n**	$C_{29}H_{24}N_4O_5$	186-189	509.18365[b]
**4o**	$C_{29}H_{24}N_4O_5$	191-193	509.18314[b]
**4p**	$C_{29}H_{24}N_4O_5$	176-179	509.18190[b]
**4q**	$C_{30}H_{27}N_3O_4$	213-215	494.20764[b]
**4r**	$C_{30}H_{27}N_3O_4$	182-185	494.20786[b]
**4s**	$C_{30}H_{27}N_3O_4$	204-206	494.20615[b]

[a]HRMS for compounds are $[M+Na]^+$, [b] HRMS for compounds are $[M+H]^+$

3. NMR data of compounds

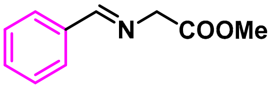

Methyl (E)-2-(benzylideneamino)acetate (**1a**). Yellow oil; yield 86%; ^{1}H NMR (400 MHz, $CDCl_3$) δ 8.28 (s, 1H), 7.80-7.74 (m, 2H), 7.45-7.39 (m, 3H), 4.41 (d, J = 1.3 Hz, 2H), 3.77 (s, 3H). ^{13}C NMR (101 MHz, $CDCl_3$) δ 170.63, 165.54, 135.59, 131.33, 129.79, 128.55, 62.04, 52.21.

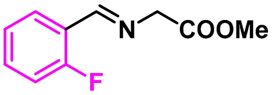

Methy-(E)-2-((2-fluorobenzylidene)amino)acetate (**1b**). Yellow oil; yield 88%; ^{1}H NMR (400 MHz, $CDCl_3$) δ 8.50 (s, 1H), 7.95 (t, J = 7.5 Hz, 1H), 7.39-7.25 (m, 1H), 7.08 (q, J = 7.6 Hz, 1H), 6.99 (dd, J = 10.6, 8.4 Hz, 1H), 4.35 (s, 2H), 3.68 (s, 3H). ^{13}C NMR (151 MHz, $CDCl_3$) δ 186.96, 170.28, 158.61, 136.33, 132.82, 127.85, 124.33, 115.72, 62.08, 51.99.

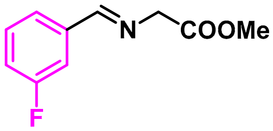

Methy-(E)-2-((3-fluorobenzylidene)amino)acetate (**1c**). Yellow oil; yield 88%; ^{1}H NMR (400 MHz, $CDCl_3$) δ 8.13 (s, 1H), 7.47-7.33 (m, 2H), 7.31-7.17 (m, 1H), 7.01 (t, J = 8.2 Hz, 1H), 4.29 (s, 2H), 3.65 (d, J = 2.3 Hz, 3H). ^{13}C NMR (101 MHz, $CDCl_3$) δ 170.32, 164.07, 161.73, 137.91, 130.26, 124.72, 118.28, 114.30, 61.67, 52.12.

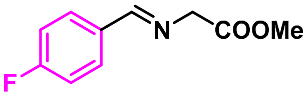

Methy-(E)-2-((4-fluorobenzylidene)amino)acetate (**1d**). Yellow oil; yield 92%; ^{1}H NMR (400 MHz, $CDCl_3$) δ 8.17 (d, J = 7.0 Hz, 1H), 7.70 (dq, J = 8.8, 3.8, 2.5 Hz, 2H), 7.01 (dt, J = 8.0, 5.4 Hz, 2H), 4.33 (s, 2H), 3.70 (s, 3H). ^{13}C NMR (151 MHz, $CDCl_3$) δ 188.22, 170.05, 161.35, 134.12, 133.71, 131.28, 129.97, 124.36, 61.67, 52.23.

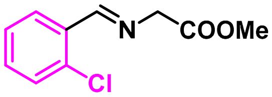

Methyl (E)-2-((2-chlorobenzylidene)amino)acetate (**1e**). Yellow oil; yield 88%; ^{1}H NMR (400 MHz, $CDCl_3$) δ 8.63 (s, 1H), 8.05-7.98 (m, 1H), 7.28-7.25 (m, 1H), 7.24-7.16 (m, 1H), 4.38 (s, 2H), 3.68 (s, 3H). ^{13}C NMR (101 MHz, $CDCl_3$) δ 170.23, 161.91, 135.34, 132.11, 129.72, 128.56, 127.02, 61.91, 52.06.

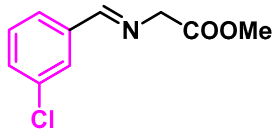

Methyl (E)-2-((3-chlorobenzylidene)amino)acetate (**1f**). Yellow oil; yield 91%; ^{1}H NMR (400 MHz, $CDCl_3$) δ 8.25 (s, 1H), 8.00 (d, *J* = 1.8 Hz, 1H), 7.68 (d, *J* = 7.7 Hz, 1H), 7.59 (dd, *J* = 7.4, 2.5 Hz, 1H), 7.31 (d, *J* = 15.3 Hz, 1H), 4.44 (d, *J* = 1.4 Hz, 2H), 3.80 (s, 3H). ^{13}C NMR (101 MHz, $CDCl_3$) δ 170.41, 164.25, 134.47, 132.49, 131.95, 131.02, 129.93, 125.84, 61.90, 52.26.

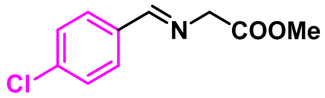

Methyl (E)-2-((4-chlorobenzylidene)amino)acetate (**1g**). Yellow oil; yield 84%; ^{1}H NMR (400 MHz, $CDCl_3$) δ 8.25 (d, J = 6.1 Hz, 1H), 7.75-7.65 (m, 2H), 7.39 (tt, J = 9.1, 4.5 Hz, 2H), 4.41 (d, J = 5.1 Hz, 2H), 3.76 (dt, J = 6.4, 3.2 Hz, 3H). ^{13}C NMR (101 MHz, CDC13) δ 170.47, 164.14, 130.97, 129.74, 129.52, 128.99, 61.90, 52.27.

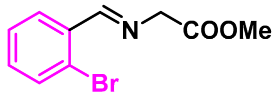

Methyl (E)-2-((2-bromobenzylidene)amino)acetate (**1h**). Yellow oil; yield 98%; ^{1}H NMR (400 MHz, $CDCl_3$) δ 8.09 (s, 1H), 7.84 (s, 1H), 7.52 (d, J = 7.8 Hz, 1H), 7.43 (d, J = 8.2 Hz, 1H), 7.16 (t, J = 7.9 Hz, 1H), 4.30 (s, 1H), 3.66 (s, 3H). ^{13}C NMR (151 MHz, $CDCl_3$) δ 170.33, 164.37, 135.35, 133.04, 129.08, 127.67, 125.37, 61.90, 52.22.

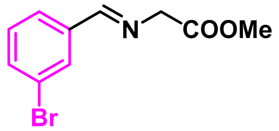

Methyl (E)-2-((3-bromobenzylidene)amino)acetate (**1i**). Yellow oil; yield 92%; ^{1}H NMR (400 MHz, $CDCl_3$) δ 8.58 (s, 1H), 8.01 (dd, J = 7.8, 1.9 Hz, 1H), 7.48 (d, J = 7.9 Hz, 1H), 7.26 (d, J = 15.0 Hz, 1H), 7.20 (dt, J = 9.4, 4.7 Hz, 1H), 4.39 (s, 2H), 3.70 (s, 3H). ^{13}C NMR (101 MHz, $CDCl_3$) δ 167.34, 161.36, 131.00, 130.07, 129.45, 126.12, 124.71, 122.41, 58.91, 49.24.

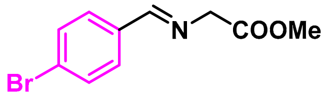

Methyl (E)-2-((4-bromobenzylidene)amino)acetate (**1j**). Yellow oil; yield 98%; ^{1}H NMR (400 MHz, $CDCl_3$) δ 8.17 (s, 1H), 7.58 (d, J = 8.5 Hz, 2H), 7.48 (d, J = 8.6 Hz, 2H), 4.34 (s, 2H), 3.71 (s, 3H). ^{13}C NMR (101 MHz, $CDCl_3$) δ 170.36, 164.18, 134.47, 131.89, 129.92, 125.76, 61.83, 52.21.

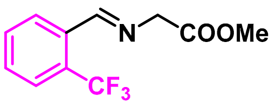

Methyl (E)-2-((2-(trifluoromethyl)benzylidene)amino)acetate (**1k**). Yellow oil; yield 93%; ^{1}H NMR (400 MHz, $CDCl_3$) δ 8.67 (s, 1H), 8.30 (d, J = 7.8 Hz, 1H), 7.70 (dd, J = 8.1, 2.8 Hz, 1H), 7.61 (td, J = 7.7, 2.5 Hz, 1H), 7.55 (dt, J = 9.8, 4.9 Hz, 1H), 4.50 (s, 2H), 3.80 (s, 3H). ^{13}C NMR (101 MHz, $CDCl_3$) δ 170.19, 161.63, 133.55, 132.10, 130.70, 128.60, 125.61, 122.75, 62.02, 52.22.

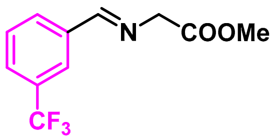

Methyl (E)-2-((2-(trifluoromethyl)benzylidene)amino)acetate (**1l**). Yellow oil; yield 84%; ^{1}H NMR (400 MHz, $CDCl_3$) δ 8.35 (d, J = 4.6 Hz, 1H), 8.08 (d, J = 4.2 Hz, 1H), 7.95 (d, J = 7.3 Hz, 1H), 7.70 (d, J = 7.1 Hz, 1H), 7.56 (q, J = 7.6, 7.0 Hz, 1H), 4.46 (s, 2H), 3.79 (s, 3H). ^{13}C NMR (101 MHz, $CDCl_3$) δ 170.29, 163.94, 136.27, 131.75, 129.24, 127.74, 125.12, 61.79, 52.28.

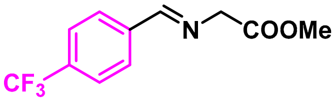

Methyl (E)-2-((2-(trifluoromethyl)benzylidene)amino)acetate (**1m**). Yellow oil; yield 83%; ^{1}H NMR (400 MHz, $CDCl_3$) δ 8.37 (s, 1H), 7.92 (d, J = 8.0 Hz, 2H), 7.70 (d, J = 8.1 Hz, 2H), 4.48 (s, 2H), 3.81 (s, 3H). ^{13}C NMR (101 MHz, $CDCl_3$) δ 170.36, 164.12, 138.74, 133.06, 128.86, 125.78, 122.63, 62.01, 52.41.

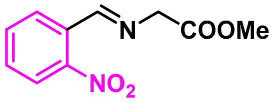

Methyl-(E)-2-((2-nitrobenzylidene)amino)acetate (**1n**). Yellow oil; yield 82%; ^{1}H NMR (400 MHz, $CDCl_3$) δ 8.70 (s, 1H), 8.12 (dd, J = 7.7, 1.9 Hz, 1H), 8.06-7.80 (m, 1H), 7.70-7.61 (m, 1H), 7.37 (d, J = 7.5 Hz, 1H), 4.50 (s, 2H), 3.82 (s, 3H). ^{13}C NMR (101 MHz, $CDCl_3$) δ 170.39, 164.47, 135.38, 134.02, 133.95, 133.09, 132.44, 129.92, 129.14, 127.96, 127.72, 125.41, 61.96, 52.27.

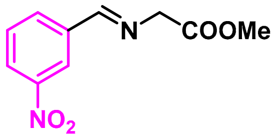

Methyl-(E)-2-((3-nitrobenzylidene)amino)acetate (**1o**). Yellow oil; yield 85%; ^{1}H NMR (400 MHz, $CDCl_3$) δ 8.61 (s, 1H), 8.40 (s, 1H), 8.30 (d, J = 10.9 Hz, 1H), 8.14 (s, 1H), 7.62 (d, J = 6.1 Hz, 1H), 4.49 (s, 2H), 3.80 (s, 3H). ^{13}C NMR (101 MHz, $CDCl_3$) δ 170.12, 162.96, 148.55, 137.20, 134.00, 129.80, 125.68, 123.27, 61.66, 52.35.

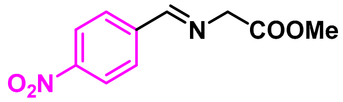

Methyl-(E)-2-((4-nitrobenzylidene)amino)acetate (**1p**). Yellow oil; yield 88%; ^{1}H NMR (400 MHz, $CDCl_3$) δ 8.39 (s, 1H), 8.26 (d, J = 6.7 Hz, 2H), 7.95 (d, J = 8.7 Hz, 2H), 4.47 (s, 2H), 3.79 (s, 3H). ^{13}C NMR (101 MHz, $CDCl_3$) δ 170.03, 163.18, 149.40, 140.94, 129.27, 123.95, 61.88, 52.40.

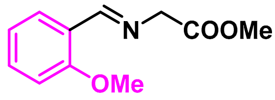

Methyl (E)-2-((2-methoxybenzylidene)amino)acetate (**1q**). Yellow oil; yield 96%; ^{1}H NMR (400 MHz, $CDCl_3$) δ 8.21 (s, 1H), 7.72 (d, J = 8.7 Hz, 2H), 6.93 (d, J = 8.7 Hz, 2H), 4.38 (s, 1H), 3.84 (s, 3H), 3.77 (s, 3H). ^{13}C NMR (151 MHz, $CDCl_3$) δ 170.86, 164.75, 162.09, 132.00, 130.15, 128.51, 114.31, 114.01, 61.96, 55.37, 52.12.

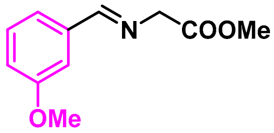

Methyl (E)-2-((3-methoxybenzylidene)amino)acetate (**1r**). Yellow oil; yield 86%; ^{1}H NMR (400 MHz, $CDCl_3$) δ 8.19 (s, 1H), 7.45-7.27 (m, 2H), 7.23 (q, J = 6.8, 4.8 Hz, 1H), 7.02-6.90 (m, 1H), 4.35 (s, 2H), 3.76 (s, 3H), 3.70 (s, 3H). ^{13}C NMR (151 MHz, $CDCl_3$) δ 189.85, 161.85, 135.99, 128.55, 124.82, 120.67, 111.64, 55.64, 52.10, 51.13.

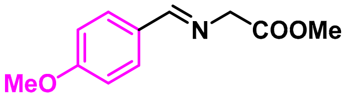

Methyl (E)-2-((4-methoxybenzylidene)amino)acetate (**1s**). Yellow oil; yield 86%; ^{1}H NMR (400 MHz, $CDCl_3$) δ 8.06 (s, 1H), 7.60 (d, J = 8.4 Hz, 2H), 6.79 (d, J = 8.4 Hz, 2H), 4.24 (s, 2H), 3.68 (s, 3H), 3.63 (s, 3H). ^{13}C NMR (151 MHz, $CDCl_3$) δ 170.85, 164.76, 162.08, 131.98, 130.15, 114.31, 61.90, 55.57, 52.09.

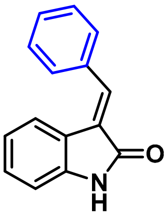

(E)-3-benzylideneindolin-2-one (**2a**). Yield: 89%; yellow solid. m.p. 170~172°C; ^{1}H NMR (400 MHz, $CDCl_3$) δ 9.19 (t, J = 13.2 Hz, 1H), 7.89 (s, 1H), 7.75-7.65 (m, 3H), 7.57-7.45 (m, 3H), 7.27 (dd, J = 15.0, 7.4 Hz, 1H), 6.98 (d, J = 7.8 Hz, 1H), 6.91 (t, J = 7.7 Hz, 1H). ^{13}C NMR (101 MHz, $CDCl_3$) δ 170.85, 141.91, 137.60, 134.91, 130.00, 129.76, 129.42, 128.74, 127.82, 123.07, 121.80 (d, J = 14.4 Hz), 110.51.

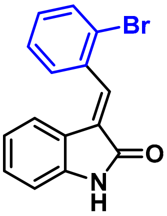

(E)-3-(2-bromobenzylidene)indolin-2-one (**2b**). Yield: 78%; yellow solid. m.p. 180~183°C; ^{1}H NMR (400 MHz, $CDCl_3$) δ 7.85 (s, 1H), 7.75 (t, J = 7.4 Hz, 2H), 7.45 (t, J = 7.6 Hz, 1H), 7.40-7.30 (m, 3H), 6.96-6.83 (m, 2H). ^{13}C NMR (151 MHz, $CDCl_3$) δ 170.11, 142.00, 135.84, 135.45, 133.22, 130.89, 130.30, 129.00, 127.29, 124.25, 123.21, 121.91, 121.35, 110.57.

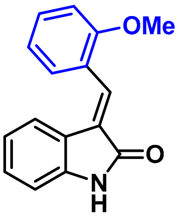

(E)-3-(2-methoxybenzylidene)indolin-2-one (**2c**). Yield: 75%; yellow solid. m.p. 214~216°C; ^{1}H NMR (400 MHz, $CDCl_3$) δ 7.82 (s, 1H), 7.74-7.62 (m, 3H), 7.23 (d, J = 7.8 Hz, 1H), 7.03 (d, J = 8.6 Hz, 2H), 6.97-6.87 (m, 2H), 3.92 (d, J = 1.6 Hz, 3H). ^{13}C NMR (151 MHz, $CDCl_3$) δ 170.05, 158.21, 141.24, 134.21, 131.59, 129.94, 129.54, 127.01, 123.72, 123.02, 122.07, 121.76, 120.16, 110.95, 110.00, 55.54.

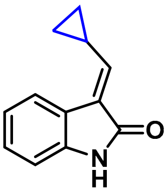

(E)-3-(cyclopropylmethylene)indolin-2-one (**2d**). Yield: 87%; reddish brown solid. m.p. 148~150°C; ^{1}H NMR (600 MHz, $CDCl_3$) δ 9.21 (s, 1H), 7.67 (d, J = 7.5 Hz, 1H), 7.20 (t, J = 7.7 Hz, 1H), 7.02 (t, J = 7.6 Hz, 1H), 6.95 (dd, J = 22.8, 7.7 Hz, 1H), 6.41 (d, J = 11.2 Hz, 1H), 2.27 (dt, J = 11.9, 5.9 Hz, 1H), 1.22 (dd, J = 17.5, 7.2 Hz, 2H), 0.91 (d, J = 3.3 Hz, 2H). ^{13}C NMR (151 MHz, $CDCl_3$) δ 170.11, 147.92, 140.54, 128.35, 126.03, 123.13, 122.69, 121.88, 110.16, 13.33, 10.50.

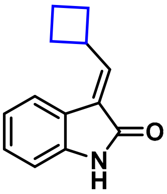

(E)-3-(cyclobutylmethylene)indolin-2-one (**2e**). Yield: 79%; yellowish green solid. m.p. 119~121°C; ^{1}H NMR (400 MHz, $CDCl_3$) δ 10.42 (s, 1H), 7.54 (d, J = 7.6 Hz, 1H), 7.20 (t, J = 7.7 Hz, 1H), 6.96 (t, J = 7.6 Hz, 1H), 6.91 (d, J = 9.3 Hz, 1H), 6.82 (d, J = 7.7 Hz, 1H), 3.81 (h, J =

8.2, 7.6 Hz, 1H), 2.45-2.29 (m, 2H), 2.11-1.85 (m, 4H). ^{13}C NMR (101 MHz, $CDCl_3$) δ 168.75, 145.05, 142.54, 129.53, 126.90, 124.05, 122.31, 121.87, 110.18, 34.63, 28.86, 19.10.

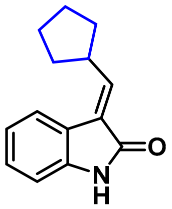

(E)-3-(cyclopentylmethylene)indolin-2-one (**2f**). Yield: 85%; orange solid. m.p. 127~130°C; ^{1}H NMR (400 MHz, $CDCl_3$) δ 10.42 (s, 1H), 7.59 (d, J = 7.6 Hz, 1H), 7.20 (t, J = 7.7 Hz, 1H), 6.96 (t, J = 8.2 Hz, 1H), 6.84 (d, J = 7.7 Hz, 1H), 6.72 (d, J = 10.0 Hz, 1H), 3.34 (s, 1H), 2.05-1.90 (m, 2H), 1.70 (dqd, J = 15.2, 7.8, 3.6 Hz, 4H), 1.43 (ddt, J = 16.3, 8.3, 4.9 Hz, 2H). ^{13}C NMR (151 MHz, $CDCl_3$) δ 170.37, 147.88, 140.96, 128.74, 126.76, 123.50, 122.76, 122.03, 110.21, 39.84, 33.22, 25.82, 25.68.

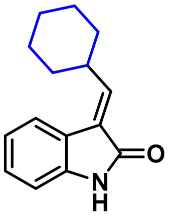

(E)-3-(cyclohexylmethylene)indolin-2-one (**2g**). Yield: 92%; yellow solid. m.p. 150~152°C; ^{1}H NMR (400 MHz, $CDCl_3$) δ 10.47 (s, 1H), 7.55 (d, J = 7.6 Hz, 1H), 7.24 (t, J = 7.7 Hz, 1H), 7.01 (t, J = 7.6 Hz, 1H), 6.86 (d, J = 7.7 Hz, 1H), 6.64 (d, J = 9.8 Hz, 1H), 3.05-2.86 (m, 1H), 1.88-1.61 (m, 5H), 1.54-1.38 (m, 2H), 1.31 (q, J = 13.7 Hz, 3H). ^{13}C NMR (101 MHz, $CDCl_3$) δ 168.81, 145.69, 142.63, 129.56, 127.02, 123.97, 122.17, 121.97, 110.29, 37.58, 31.71, 25.85, 25.42.

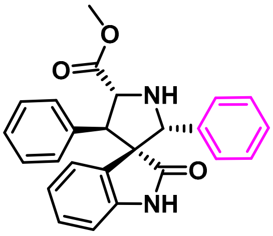

Methyl-(2'R, 3S, 4'R, 5'R)-2-oxo-4'-phenylspiro[indoline-3, 3'-pyrrolidine]-5'-carboxylate (**3a**). Yield: 97%; white solid. m.p. 181~183°C; ^{1}H NMR (600 MHz, DMSO-d_6) δ 10.06 (s, 1H), 7.20 (d, J = 8.5 Hz, 5H), 7.15 (d, J = 6.3 Hz, 4H), 6.97 (t, J = 7.6 Hz, 1H), 6.92 (d, J = 6.9 Hz, 2H), 6.77-6.65 (m, 2H), 6.59-6.46 (m, 1H), 4.67 (q, J = 9.0, 7.2 Hz, 2H), 4.00 (d, J = 8.1 Hz, 1H), 3.67 (s, 3H). ^{13}C NMR (151 MHz, DMSO-d_6) δ 175.94, 170.60, 141.54, 135.19, 127.34, 126.50, 126.02, 125.93, 125.38, 125.02, 123.28, 122.43, 119.13, 106.93, 68.66, 61.79, 61.61, 55.07, 49.96. ESI-MS: m/z 399.17029 [M + H]$^+$.

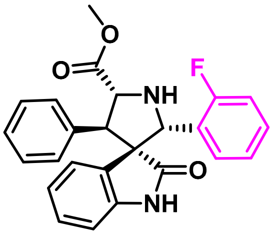

Methyl-(2'R, 3S, 4'R, 5'R)-2'-(2-fluorophenyl)-2-oxo-4'-phenylspiro[indoline-3, 3'-pyrrolidine]-5'-carboxylate (**3b**). Yield: 82%; white solid. m.p. 375~377°C; ^{1}H NMR (400 MHz, DMSO-d$_6$) δ 10.14 (s, 1H), 7.96 (d, J = 7.5 Hz, 1H), 7.42 (d, J = 7.5 Hz, 1H), 7.32-7.18 (m, 2H), 7.15-7.03 (m, 4H), 7.01-6.89 (m, 4H), 6.58 (d, J = 7.7 Hz, 1H), 4.87 (d, J = 8.4 Hz, 1H), 4.77 (dd, J = 10.5, 8.5 Hz, 1H), 4.28 (t, J = 8.5 Hz, 1H), 3.95 (d, J = 10.5 Hz, 1H), 3.65 (s, 3H). ^{13}C NMR (101 MHz, DMSO-d$_6$) δ 176.74, 173.94, 141.76, 135.62, 131.10, 130.40, 129.11, 129.03, 128.86, 128.43, 128.37, 127.69, 125.24, 124.21, 121.75, 114.58, 109.36, 63.94, 63.70, 63.11, 56.40, 52.48. ESI-MS: m/z 417.16130 [M + H]$^+$.

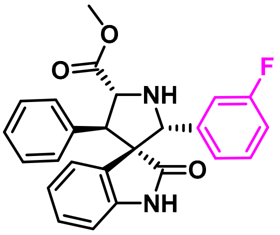

Methyl-(2'R, 3S, 4'R, 5'R)-2'-(3-fluorophenyl)-2-oxo-4'-phenylspiro[indoline-3, 3'-pyrrolidine]-5'-carboxylate (**3c**). Yield: 80%; white solid. m.p. 189~192°C; ^{1}H NMR (400 MHz, DMSO-d$_6$) δ 10.14 (s, 1H), 7.18 (dt, J = 14.0, 6.5 Hz, 7H), 7.06-6.98 (m, 3H), 6.90 (t, J = 9.7 Hz, 2H), 6.80-6.70 (m, 2H), 6.58 (d, J = 7.7 Hz, 1H), 4.78-4.66 (m, 2H), 4.03 (d, J = 8.3 Hz, 1H), 3.91 (t, J = 9.2 Hz, 1H), 3.69 (s, 3H). ^{13}C NMR (101 MHz, DMSO-d$_6$) δ 178.64, 173.23, 160.96, 142.33, 138.01, 130.03, 129.74, 129.08, 128.59, 128.38, 127.56, 125.75, 123.03, 121.57, 114.63, 114.03, 109.40, 71.13, 64.41, 64.07, 57.51, 52.53. ESI-MS: m/z 417.16105 [M + H]$^+$.

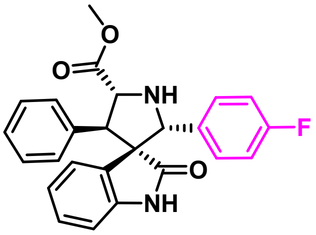

Methyl-(2'R, 3S, 4'R, 5'R)-2'-(4-fluorophenyl)-2-oxo-4'-phenylspiro[indoline-3, 3'-pyrrolidine]-5'-carboxylate (**3d**). Yield: 92%; white solid. m.p. 165~168°C; ^{1}H NMR (400 MHz, DMSO-d$_6$) δ 10.09 (s, 1H), 7.19 (tt, J = 11.5, 5.9 Hz, 5H), 7.01 (d, J = 7.4 Hz, 5H), 6.81 (d, J = 7.5 Hz, 1H), 6.73 (t, J = 7.5 Hz, 1H), 6.55 (d, J = 7.7 Hz, 1H), 4.68 (t, J = 8.6 Hz, 2H), 4.02 (d, J = 8.2 Hz, 1H), 3.81 (t, J = 9.5 Hz, 1H), 3.68 (s, 3H). ^{13}C NMR (101 MHz, DMSO-d$_6$) δ 178.97, 173.18, 142.38, 138.35, 134.65, 129.58, 129.12, 128.93, 128.85, 128.60, 128.28, 127.53, 125.75, 121.49, 115.02, 114.80, 109.35, 71.32, 64.59, 64.14, 57.71, 52.54. ESI-MS: m/z 417.16045 [M + H]$^+$.

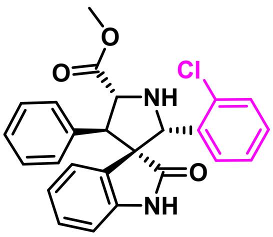

Methyl-(2'R, 3S, 4'R, 5'R)-2'-(2-chlorophenyl)-2-oxo-4'-phenylspiro[indoline-3, 3'-pyrrolidine]-5'-carboxylate (**3e**). Yield: 72%; white solid. m.p. 188~191°C; ^{1}H NMR (400 MHz, DMSO-d$_6$) δ 10.09 (s, 1H), 8.21 (d, J = 7.8 Hz, 1H), 7.64 (d, J = 7.4 Hz, 1H), 7.42 (dt, J = 8.2, 4.3 Hz, 1H), 7.26 (d, J = 4.1 Hz, 2H), 7.15-7.08 (m, 1H), 7.03 (dd, J = 13.6, 6.9 Hz, 4H), 6.90-6.82 (m, 2H), 6.57 (d, J = 7.6 Hz, 1H), 4.95 (d, J = 7.8 Hz, 1H), 4.81-4.73 (m, 1H), 4.49 (t, J = 8.0 Hz, 1H), 3.93 (d, J = 11.3 Hz, 1H), 3.64 (s, 3H). ^{13}C NMR (101 MHz, DMSO-d$_6$) δ 175.94, 174.20, 141.68, 140.09, 134.47, 132.71, 131.31, 128.81, 128.70, 128.52, 128.48, 128.26, 127.77, 126.99, 124.92, 121.85, 109.35, 66.60, 63.47, 62.69, 56.15, 52.46. ESI-MS: m/z 433.13242 [M + H]$^+$.

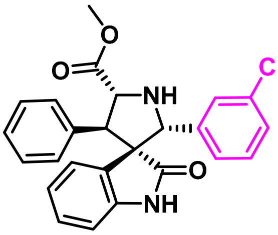

Methyl-(2'R, 3S, 4'R, 5'R)-2'-(3-chlorophenyl)-2-oxo-4'-phenylspiro[indoline-3, 3'-pyrrolidine]-5'-carboxylate (**3f**). Yield: 80%; white solid. m.p. 158~161°C; ^{1}H NMR (400 MHz, DMSO-d$_6$) δ 10.16 (s, 1H), 7.29-7.11 (m, 8H), 7.03 (t, J = 7.7 Hz, 1H), 6.91 (d, J = 7.5 Hz, 1H), 6.84 (d, J = 7.6 Hz, 1H), 6.77 (t, J = 7.3 Hz, 1H), 6.57 (d, J = 7.7 Hz, 1H), 4.74-4.66 (m, 2H), 4.02 (d, J = 8.6 Hz, 1H), 3.93 (t, J = 9.0 Hz, 1H), 3.68 (s, 3H). ^{13}C NMR (101 MHz, DMSO-d$_6$) δ 178.56, 173.21, 142.28, 141.75, 137.90, 132.93, 129.94, 129.06, 128.58, 128.41, 127.70, 127.57, 127.08, 125.77, 125.71, 121.61, 109.41, 71.05, 64.31, 64.04, 57.38, 52.53. ESI-MS: m/z 433.13214 [M + H]$^+$.

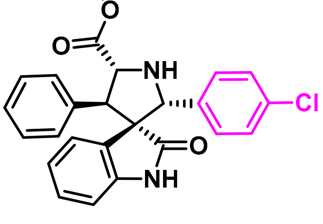

Methyl-(2'R, 3S, 4'R, 5'R)-2'-(4-chlorophenyl)-2-oxo-4'-phenylspiro[indoline-3, 3'-pyrrolidine]-5'-carboxylate (**3g**). Yield: 80%; white solid. m.p. 180~183°C; ^{1}H NMR (400 MHz, DMSO-d$_6$) δ 9.98 (d, J = 3.6 Hz, 1H), 7.16-7.10 (m, 2H), 7.05 (q, J = 7.0, 6.1 Hz, 5H), 6.92-6.84 (m, 3H), 6.73 (d, J = 7.4 Hz, 1H), 6.62 (t, J = 7.6 Hz, 1H), 6.43 (d, J = 7.7 Hz, 1H), 4.57 (dd, J =

9.3, 4.2 Hz, 2H), 3.88 (d, J = 8.4 Hz, 1H), 3.72 (t, J = 9.3 Hz, 1H), 3.55 (s, 3H). ^{13}C NMR (101 MHz, DMSO-d$_6$) δ 178.77, 173.16, 142.34, 138.15, 137.75, 132.37, 129.62, 129.09, 128.85, 128.60, 128.35, 128.12, 127.54, 125.77, 121.55, 109.39, 71.21, 64.49, 64.13, 57.61, 52.54. ESI-MS: m/z 433.13203 [M + H]$^+$.

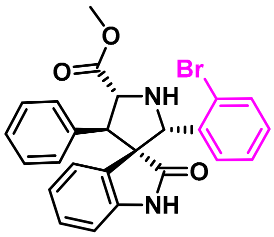

Methyl-(2'R, 3S, 4'R, 5'R)-2'-(2-bromophenyl)-2-oxo-4'-phenylspiro[indoline-3, 3'-pyrrolidine]-5'-carboxylate (**3h**). Yield: 78%; white solid. m.p. 195~197°C; ^{1}H NMR (400 MHz, DMSO-d$_6$) δ 10.05 (s, 1H), 7.33 (d, J = 8.2 Hz, 2H), 7.12 (q, J = 7.0 Hz, 5H), 6.99-6.92 (m, 1H), 6.88 (d, J = 8.2 Hz, 2H), 6.80 (d, J = 7.5 Hz, 1H), 6.69 (t, J = 7.5 Hz, 1H), 6.50 (d, J = 7.7 Hz, 1H), 4.68-4.58 (m, 2H), 3.95 (d, J = 8.4 Hz, 1H), 3.79 (t, J = 9.3 Hz, 1H). ^{13}C NMR (101 MHz, DMSO-d$_6$) δ 175.97, 174.36, 141.89, 134.47, 131.95, 131.90, 129.19, 128.97, 128.67, 128.39, 127.93, 127.63, 125.01, 123.81, 121.98, 109.50, 68.94, 63.60, 62.86, 56.28, 52.61. ESI-MS: m/z 477.08041 [M + H]$^+$.

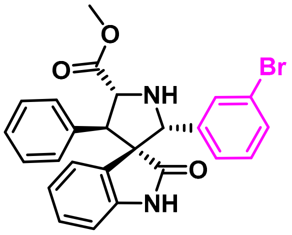

Methyl-(2'R, 3S, 4'R, 5'R)-2'-(3-bromophenyl)-2-oxo-4'-phenylspiro[indoline-3, 3'-pyrrolidine]-5'-carboxylate (**3i**). Yield: 58%; white solid. m.p. 143~145°C; ^{1}H NMR (400 MHz, DMSO-d$_6$) δ 10.17 (s, 1H), 7.39 (dd, J = 7.9, 2.0 Hz, 1H), 7.31 (d, J = 1.7 Hz, 1H), 7.15 (td, J = 12.6, 11.2, 7.1 Hz, 6H), 7.07-7.00 (m, 1H), 6.90 (dd, J = 17.1, 7.6 Hz, 2H), 6.78 (t, J = 7.5 Hz, 1H), 6.58 (d, J = 7.7 Hz, 1H), 4.70 (dt, J = 8.5, 3.7 Hz, 2H), 4.04 (t, J = 7.9 Hz, 1H), 3.93 (t, J = 9.0 Hz, 1H), 3.69 (s, 3H).^{13}C NMR (101 MHz, DMSO-d$_6$) δ 178.55, 173.18, 142.28, 141.98, 137.89, 130.58, 130.24, 129.97, 129.05, 128.58, 128.40, 127.56, 126.08, 125.78, 121.64, 121.60, 109.41, 71.02, 64.30, 64.05, 57.38, 52.53. ESI-MS: m/z 477.08144 [M + H]$^+$.

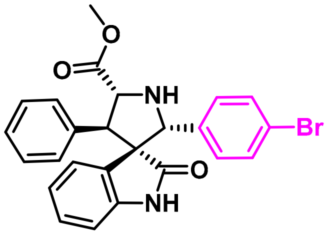

Methyl-(2'R, 3S, 4'R, 5'R)-2'-(4-bromophenyl)-2-oxo-4'-phenylspiro[indoline-3, 3'-pyrrolidine]-5'-carboxylate (**3j**). Yield: 69%; white solid. m.p. 189~191°C; ^{1}H NMR (400 MHz, DMSO-d$_6$) δ 10.11 (s, 1H), 7.39 (d, J = 8.2 Hz, 2H), 7.24-7.11 (m, 5H), 7.05-6.99 (m, 1H), 6.94 (d, J = 8.2 Hz, 2H), 6.86 (d, J = 7.5 Hz, 1H), 6.75 (t, J = 7.5 Hz, 1H), 6.57 (d, J = 7.7 Hz, 1H), 4.74-4.64 (m, 2H), 4.02 (d, J = 8.4 Hz, 1H), 3.85 (t, J = 9.3 Hz, 1H), 3.68 (s, 3H).^{13}C NMR (101 MHz, DMSO-d$_6$) δ 178.75, 173.15, 142.33, 138.19, 138.13, 131.02, 129.64, 129.21, 129.09, 128.59, 128.35, 127.54, 125.77, 121.56, 120.98, 109.40, 71.26, 64.47, 64.08, 57.62, 52.54. ESI-MS: m/z 477.08105 [M + H]$^+$.

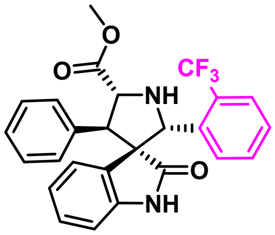

Methyl-(2'R, 3S, 4'R, 5'R)-2'-(2-(trifluoromethyl)phenyl)-2-oxo-4'-phenylspiro[indoline-3, 3'-pyrrolidine]-5'-carboxylate (**3k**). Yield: 55%; white solid. m.p. 165~167°C; ^{1}H NMR (600MHz, DMSO-d$_6$) δ 9.96 (s, 1H), 8.51 (d, J = 7.9 Hz, 1H), 7.75 (t, J = 7.5 Hz, 1H), 7.65 (d, J = 7.3 Hz, 2H), 7.54 (d, J = 7.5 Hz, 1H), 7.45 (t, J = 7.6 Hz, 1H), 7.14-7.07 (m, 1H), 7.06-6.97 (m, 5H), 6.81 (d, J = 6.9 Hz, 2H), 6.54-6.48 (m, 1H), 4.96 (d, J = 7.7 Hz, 1H), 4.74 (dd, J = 11.3, 8.2 Hz, 1H), 4.67 (t, J = 8.0 Hz, 1H), 3.63 (s, 3H).^{13}C NMR (151 MHz, DMSO-d$_6$) δ 173.31, 171.67, 139.91, 139.06, 131.41, 129.63, 129.60, 126.27, 126.07, 125.66, 125.31, 125.09, 121.99, 119.37, 106.77, 62.45, 61.37, 59.96, 53.72, 49.93. ESI-MS: m/z 467.40047 [M + H]$^+$.

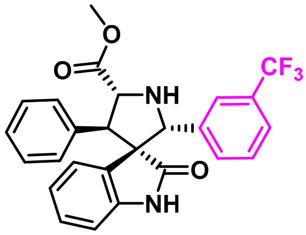

Methyl-(2'R, 3S, 4'R, 5'R)-2'-(3-(trifluoromethyl)phenyl)-2-oxo-4'-phenylspiro[indoline-3, 3'-pyrrolidine]-5'-carboxylate (**3l**). Yield: 82%; white solid. m.p. 166~168°C; ^{1}H NMR (600 MHz,

DMSO-d_6) δ 10.10 (s, 1H), 7.54 (d, J = 7.8 Hz, 1H), 7.43-7.39 (m, 2H), 7.24 (d, J = 7.8 Hz, 1H), 7.19-7.15 (m, 2H), 7.15-7.11 (m, 4H), 7.02 (t, J = 7.7 Hz, 2H), 6.78 (t, J = 7.1 Hz, 1H), 6.55 (d, J = 7.6 Hz, 1H), 4.78 (d, J = 9.3 Hz, 1H), 4.72 (t, J = 8.6 Hz, 1H), 4.05-3.98 (m, 2H), 3.67 (s, 3H). ^{13}C NMR (151 MHz, DMSO-d_6) δ 170.66, 128.55, 126.55, 126.51, 126.03, 125.91, 125.03, 123.28, 121.90, 121.33, 119.12, 106.89, 68.57, 61.81, 61.59, 54.71, 49.94. ESI-MS: m/z 467.15780 [M + H]$^+$.

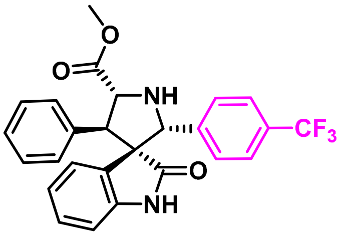

Methyl-(2'R, 3S, 4'R, 5'R)-2'-(4-(trifluoromethyl)phenyl)-2-oxo-4'-phenylspiro[indoline-3, 3'-pyrrolidine]-5'-carboxylate (**3m**). Yield: 99%; white solid. m.p. 177~180°C; ^{1}H NMR (400 MHz, $CDCl_3$) δ 7.99 (s, 1H), 7.32 (dd, J = 9.1, 4.7 Hz, 3H), 7.24 (t, J = 6.4 Hz, 2H), 7.13-7.03 (m, 3H), 6.93 (d, J = 8.3 Hz, 2H), 6.74 (td, J = 7.2, 6.7, 2.2 Hz, 1H), 6.62 (d, J = 7.8 Hz, 1H), 6.13 (dd, J = 13.6, 7.6 Hz, 1H), 4.73 (s, 1H), 4.62 (d, J = 5.2 Hz, 1H), 4.20 (d, J = 5.3 Hz, 1H), 3.85 (s, 3H). ^{13}C NMR (151 MHz, DMSO-d_6) δ 176.77, 170.58, 140.00, 136.12, 135.48, 127.03, 126.64, 126.07, 125.64, 125.31, 124.96, 124.35, 123.23, 118.85, 106.77, 69.66, 64.97, 62.34, 61.74, 55.79, 49.99. ESI-MS: m/z 467.15715 [M + H]$^+$.

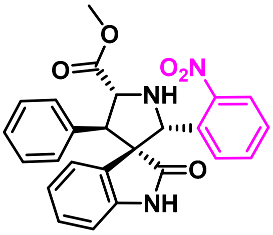

Methyl-(2'R, 3S, 4'R, 5'R)-2'-(2-nitrophenyl)-2-oxo-4'-phenylspiro[indoline-3, 3'-pyrrolidine]-5'-carboxylate (**3n**). Yield: 52%; yellow solid. m.p. 190~193°C; ^{1}H NMR (400 MHz, DMSO-d_6) δ 10.09 (s, 1H), 8.01-7.92 (m, 1H), 7.40 (d, J = 7.5 Hz, 1H), 7.30-7.19 (m, 2H), 7.13-7.05 (m, 4H), 7.01-6.89 (m, 4H), 6.58 (d, J = 7.7 Hz, 1H), 4.89 (d, J = 8.2 Hz, 1H), 4.81-4.72 (m, 1H), 4.22 (t, J = 8.5 Hz, 1H), 3.66 (s, 3H). ^{13}C NMR (101 MHz, DMSO-d_6) δ 174.56, 147.23, 141.84, 140.19, 134.18, 133.72, 132.46, 131.91, 128.79, 128.60, 128.47, 128.18, 127.91, 124.51, 124.28, 121.86, 109.44, 65.41, 64.09, 62.58, 56.09, 52.49. ESI-MS: m/z 444.15518 [M + H]$^+$.

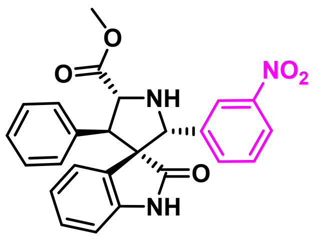

Methyl-(2'R, 3S, 4'R, 5'R)-2'-(3-nitrophenyl)-2-oxo-4'-phenylspiro[indoline-3, 3'-pyrrolidine]-5'-carboxylate (**3o**). Yield: 96%; yellow solid. m.p. 148~150°C; ^{1}H NMR (400 MHz, DMSO-d_6) δ 10.05 (s, 1H), 7.25-7.12 (m, 5H), 7.02 (d, J = 7.2 Hz, 5H), 6.82 (d, J = 7.5 Hz, 1H), 6.74 (td, J = 7.5, 1.1 Hz, 1H), 6.56 (d, J = 7.7 Hz, 1H), 4.83-4.59 (m, 2H), 4.03 (d, J = 8.3 Hz, 1H), 3.69 (s, 3H). ^{13}C NMR (101 MHz, DMSO-d_6) δ 177.85, 173.38, 147.79, 142.81, 142.00, 137.03, 134.05, 130.10, 129.57, 128.97, 128.59, 128.54, 127.65, 125.73, 122.65, 122.25, 121.85, 109.55, 70.31, 64.06, 63.95, 56.78, 52.55. ESI-MS: m/z 444.15484 [M + H]$^{+}$.

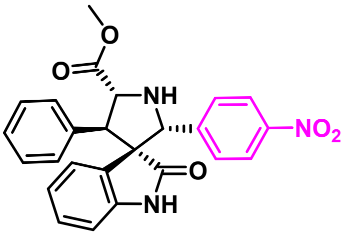

Methyl-(2'R, 3S, 4'R, 5'R)-2'-(4-nitrophenyl)-2-oxo-4'-phenylspiro[indoline-3, 3'-pyrrolidine]-5'-carboxylate (**3p**). Yield: 94%; yellow solid. m.p. 188~190°C; ^{1}H NMR (400 MHz, DMSO-d_6) δ 10.14 (s, 1H), 8.15-8.03 (m, 2H), 7.35 (d, J = 9.7 Hz, 2H), 7.20-6.98 (m, 7H), 6.82 (t, J = 6.1 Hz, 1H), 6.56 (t, J = 8.2 Hz, 1H), 4.88-4.68 (m, 2H), 4.13 (d, J = 5.6 Hz, 1H), 4.03 (d, J = 8.5 Hz, 1H), 3.67 (s, 3H).^{13}C NMR (101 MHz, DMSO-d_6) δ 177.93, 173.27, 147.17, 142.06, 137.17, 129.99, 128.99, 128.59, 128.55, 127.65, 125.76, 123.24, 121.81, 109.56, 70.66, 64.19, 64.01, 57.18, 52.55. ESI-MS: m/z 444.15397 [M + H]$^{+}$.

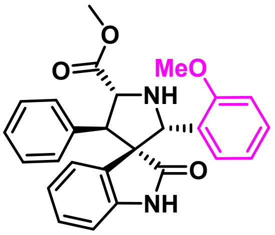

Methyl-(2'R, 3S, 4'R, 5'R)-2'-(2-methoxyphenyl)-2-oxo-4'-phenylspiro[indoline-3, 3'-pyrrolidine]-5'-carboxylate (**3q**). Yield: 74%; white solid. m.p. 211~213°C; ^{1}H NMR (600 MHz, DMSO-d_6) δ 9.90 (s, 1H), 7.63 (d, J = 7.5 Hz, 1H), 7.18-7.08 (m, 4H), 6.95 (dt, J = 13.6, 7.6 Hz, 2H), 6.79 (d, J = 7.4 Hz, 2H), 6.60-6.51 (m, 3H), 6.35 (d, J = 7.6 Hz, 1H), 4.76 (t, J = 9.4 Hz, 1H), 4.42 (d, J = 11.8 Hz, 1H), 4.12 (d, J = 9.0 Hz, 1H), 3.69 (s, 3H), 3.15 (s, 3H). ^{13}C NMR (151 MHz, DMSO-d_6) δ 177.88, 170.60, 154.81, 140.49, 134.46, 127.11, 125.85, 125.59, 125.31, 125.19,

125.09, 124.78, 124.11, 121.93, 118.23, 117.80, 108.07, 106.20, 69.71, 61.00, 60.00, 52.34, 50.11, 49.78. ESI-MS: m/z 429.17924 [M + H]$^+$.

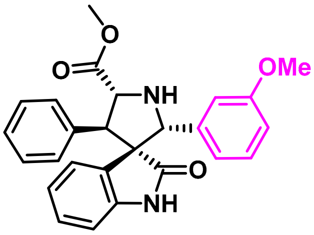

Methyl-(2'R, 3S, 4'R, 5'R)-2'-(3-methoxyphenyl)-2-oxo-4'-phenylspiro[indoline-3, 3'-pyrrolidine]-5'-carboxylate (**3r**). Yield: 78%; white solid. m.p. 115~118°C; ^{1}H NMR (600 MHz, DMSO-d_6) δ 10.04 (s, 1H), 7.23-7.11 (m, 5H), 7.05-6.94 (m, 3H), 6.79-6.61 (m, 4H), 6.53 (d, J = 7.6 Hz, 1H), 4.72-4.56 (m, 2H), 4.07-3.92 (m, 1H), 3.67 (s, 3H), 2.14 (s, 3H). ^{13}C NMR (101 MHz, DMSO-d_6) δ 180.36, 173.10, 157.43, 143.07, 137.06, 129.64, 128.38, 128.10, 127.82, 127.70, 127.42, 126.68, 124.47, 120.76, 120.37, 110.71, 108.76, 72.31, 63.58, 62.73, 54.93, 52.58, 52.33. ESI-MS: m/z 429.18090[M + H]$^+$.

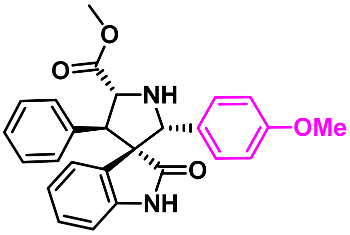

Methyl-(2'R, 3S, 4'R, 5'R)-2'-(4-methoxyphenyl)-2-oxo-4'-phenylspiro[indoline-3, 3'-pyrrolidine]-5'-carboxylate (**3s**). Yield: 67%; white solid. m.p. 115~117°C; ^{1}H NMR (600 MHz, DMSO-d_6) δ 9.89 (s, 1H), 7.63 (d, J = 7.6 Hz, 1H), 7.13 (ddd, J = 12.9, 7.3, 5.5 Hz, 4H), 7.03-6.89 (m, 2H), 6.80 (s, 2H), 6.63-6.52 (m, 3H), 6.36 (d, J = 7.6 Hz, 1H), 4.76 (t, J = 9.6 Hz, 1H), 4.43 (d, J = 12.1 Hz, 1H), 4.13 (d, J = 8.9 Hz, 1H), 3.70 (s, 3H), 3.16 (s, 3H). ^{13}C NMR (151 MHz, DMSO-d_6) δ 176.74, 170.62, 156.48, 140.09, 137.12, 126.67, 126.09, 125.65, 124.99, 123.24, 118.86, 116.40, 110.75, 109.87, 106.80, 69.42, 62.35, 61.65, 55.65, 52.60, 50.02. ESI-MS: m/z 429.18148 [M + H]$^+$.

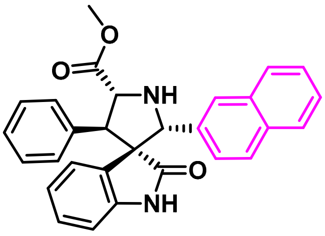

Methyl-(2'R, 3S, 4'R, 5'R)-2'-(naphthalen-2-yl)-2-oxo-4'-phenylspiro[indoline-3, 3'-pyrrolidine]-5'-carboxylate (**3t**). Yield: 79%; white solid. m.p. 213~215°C; ^{1}H NMR (600 MHz, DMSO-d_6) δ 9.82 (s, 1H), 7.87-7.81 (m, 1H), 7.81-7.73 (m, 2H), 7.62 (d, J = 8.6 Hz, 1H), 7.55 (s,

1H), 7.50-7.39 (m, 2H), 7.16-7.03 (m, 7H), 6.85-6.78 (m, 1H), 6.43 (d, J = 2.4 Hz, 1H), 4.83 (d, J = 13.7 Hz, 1H), 4.69-4.59 (m, 2H), 4.54 (dd, J = 13.7, 9.7 Hz, 1H), 3.31 (s, 3H). ^{13}C NMR (151 MHz, DMSO-d$_6$) δ 176.58, 170.63, 139.93, 135.78, 133.54, 130.43, 130.38, 127.38, 126.62, 126.25, 126.08, 125.77, 125.33, 125.00, 124.91, 123.99, 123.75, 123.35, 122.96, 122.78, 119.01, 106.82, 69.72, 65.14, 62.15, 61.86, 55.66, 50.05, 18.76, 12.07. ESI-MS: m/z 417.16130 [M + H]$^+$.

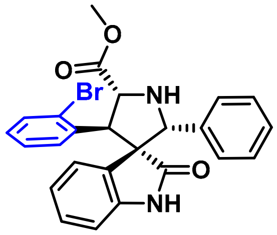

Methyl-(2'R, 3S, 4'R, 5'R)-4'-(2-bromophenyl)-2-oxo-2'-phenylspiro[indoline-3,3'-pyrrolidine]-5'-carboxylate (**3u**). Yield: 93%; white solid. m.p. 192~194°C; ^{1}H NMR (400 MHz, DMSO-d$_6$) δ 10.07 (s, 1H), 8.04 (d, J = 8.0 Hz, 1H), 7.52 (t, J = 8.3 Hz, 1H), 7.43 (d, J = 6.8 Hz, 1H), 7.23-7.10 (m, 4H), 7.00 (td, J = 7.7, 1.2 Hz, 1H), 6.83 (d, J = 8.5 Hz, 2H), 6.62 (t, J = 7.8 Hz, 1H), 6.54 (d, J = 7.7 Hz, 1H), 6.34 (d, J = 7.5 Hz, 1H), 4.80-4.72 (m, 1H), 4.64 (d, J = 12.1 Hz, 1H), 4.40 (d, J = 7.0 Hz, 1H), 3.72 (s, 3H). ^{13}C NMR (101 MHz, DMSO-d$_6$) δ 179.57, 172.95, 138.64, 136.36, 132.97, 130.47, 129.48, 128.32, 128.22, 128.16, 128.08, 127.79, 126.80, 126.71, 124.95, 121.14, 109.33, 72.09, 64.79, 63.45, 56.85, 52.73. ESI-MS: m/z 477.08217 [M + H]$^+$.

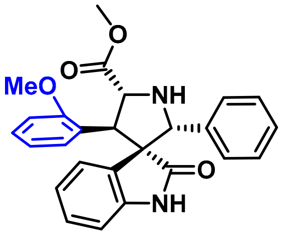

Methyl-(2'R, 3S, 4'R, 5'R)-4'-(2-methoxyphenyl)-2-oxo-2'-phenylspiro[indoline-3,3'-pyrrolidine]-5'-carboxylate (**3v**). Yield: 91%; white solid. m.p. 207~210°C; ^{1}H NMR (400 MHz, DMSO-d$_6$) δ 9.92 (s, 1H), 7.65 (d, J = 7.6 Hz, 1H), 7.22-7.09 (m, 4H), 6.96 (dd, J = 8.2, 6.9 Hz, 2H), 6.81 (d, J = 6.9 Hz, 2H), 6.64-6.52 (m, 3H), 6.37 (d, J = 8.1 Hz, 1H), 4.78 (t, J = 9.6 Hz, 1H), 4.44 (d, J = 11.9 Hz, 1H), 4.15 (d, J = 9.0 Hz, 1H), 3.71 (s, 3H), 3.54 (t, J = 11.4 Hz, 1H), 3.18 (s, 3H).^{13}C NMR (101 MHz, DMSO-d$_6$) δ 180.40, 173.12, 157.35, 143.02, 136.99, 129.63, 128.37, 128.11, 127.83, 127.72, 127.62, 127.32, 126.64, 124.46, 120.75, 120.33, 110.61, 108.73, 72.24, 63.53, 62.55, 54.87, 52.62, 52.31. ESI-MS: m/z 429.18141 [M + H]$^+$.

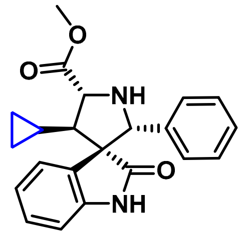

Methyl-(2'R, 3S, 4'R, 5'R)-4'-cyclopropyl-2-oxo-2'-phenylspiro[indoline-3,3'-pyrrolidine]-5'-carboxylate (**3w**). Yield: 65%; white solid. m.p. 189~191°C; ^{1}H NMR (400 MHz, DMSO-d_6) δ 10.59 (s, 1H), 8.20 (d, J = 7.4 Hz, 1H), 7.78-7.66 (m, 4H), 7.61 (t, J = 7.5 Hz, 1H), 7.47 (dd, J = 6.7, 2.9 Hz, 2H), 7.26 (d, J = 7.7 Hz, 1H), 5.14 (d, J = 11.2 Hz, 1H), 4.68-4.56 (m, 1H), 4.33 (s, 3H), 4.04 (t, J = 10.7 Hz, 1H), 2.52-2.43 (m, 1H), 1.56 (dtt, J = 9.6, 7.9, 4.9 Hz, 1H), 1.01 (qd, J = 8.8, 5.6 Hz, 1H), 0.69 (dq, J = 9.6, 4.9 Hz, 1H), 0.60-0.46 (m, 1H), 0.16 (dq, J = 9.9, 5.0 Hz, 1H). ^{13}C NMR (101 MHz, DMSO-d_6) δ 179.90, 173.52, 142.67, 138.19, 130.92, 128.20, 128.12, 127.68, 126.74, 125.95, 121.75, 109.48, 71.78, 66.34, 62.97, 59.56, 52.48, 12.39, 4.82, 4.01. ESI-MS: m/z 363.17108 [M + H]$^+$.

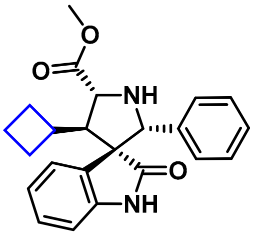

Methyl-(2'R, 3S, 4'R, 5'R)-4'-cyclobutyl-2-oxo-2'-phenylspiro[indoline-3,3'-pyrrolidine]-5'-carboxylate (**3x**). Yield: 93%; white solid. m.p. 200~202°C; ^{1}H NMR (400 MHz, DMSO-d_6) δ 10.05 (s, 1H), 7.61 (d, J = 7.4 Hz, 1H), 7.23-7.11 (m, 4H), 7.06 (t, J = 7.5 Hz, 1H), 6.88 (q, J = 4.3, 3.4 Hz, 2H), 6.69 (d, J = 7.8 Hz, 1H), 4.62-4.34 (m, 1H), 3.81 (t, J = 8.7 Hz, 1H), 3.44-3.28 (m, 4H), 2.82-2.69 (m, 1H), 2.61 (q, J = 10.7, 9.3 Hz, 1H), 1.98 (q, J = 7.1, 4.7 Hz, 1H), 1.60 (tq, J = 19.2, 8.5, 8.0 Hz, 3H), 1.09 (p, J = 9.1, 8.6 Hz, 1H), 0.98 (s, 1H). ^{13}C NMR (101 MHz, DMSO-d_6) δ 180.18, 173.71, 142.68, 138.00, 130.37, 128.36, 128.13, 127.69, 126.65, 125.47, 121.81, 109.45, 72.12, 65.45, 61.84, 61.27, 52.47, 36.75, 27.80, 27.56, 18.48. ESI-MS: m/z 377.18645 [M + H]$^+$.

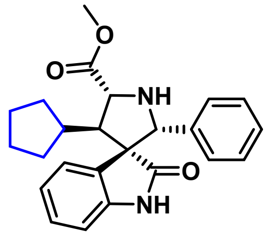

Methyl-(2'R,3S,4'R,5'R)-4'-cyclopentyl-2-oxo-2'-phenylspiro[indoline-3,3'-pyrrolidine]-5'-carboxylate (**3y**). Yield: 87%; white solid. m.p. 207~209°C; ^{1}H NMR (400 MHz, CDCl$_3$) δ 7.78 (s, 1H), 7.41 (d, J = 7.4 Hz, 1H), 7.27 (d, J = 7.8 Hz, 1H), 7.19-7.09 (m, 4H), 6.96-6.88 (m, 2H), 6.78 (d, J = 7.8 Hz, 1H), 4.53 (s, 1H), 3.93 (d, J = 9.4 Hz, 1H), 3.88 (s, 3H), 2.94 (dd, J = 11.1, 9.3 Hz, 1H), 2.05 (d, J = 22.8 Hz, 2H), 1.87 (p, J = 7.4, 6.8 Hz, 1H), 1.43-1.29 (m, 3H), 1.10 (p, J = 9.8 Hz, 1H), 0.88 (ddd, J = 14.2, 7.7, 3.8 Hz, 1H), 0.80-0.65 (m, 1H). ^{13}C NMR (101 MHz, CDCl$_3$) δ 180.52, 173.30, 141.12, 136.17, 130.94, 128.14, 127.95, 127.75, 126.15, 124.60, 122.40, 109.80, 74.31, 67.39, 63.42, 60.67, 52.47, 41.35, 31.48, 30.69, 25.50, 24.45. ESI-MS: m/z 391.2029 [M + H]$^{+}$.

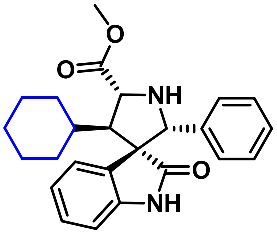

Methyl-(2'R,3S,4'R,5'R)-4'-cyclohexyl-2-oxo-2'-phenylspiro[indoline-3,3'-pyrrolidine]-5'-carboxylate (**3z**). Yield: 91%; white solid. m.p. 215~217°C; ^{1}H NMR (400 MHz, CDCl$_3$) δ 7.44-7.34 (m, 2H), 7.16 (ddd, J = 14.1, 10.1, 4.9 Hz, 4H), 6.88 (d, J = 6.7 Hz, 2H), 6.77 (d, J = 7.7 Hz, 1H), 4.48 (s, 1H), 3.98 (d, J = 9.3 Hz, 1H), 3.89 (s, 3H), 2.91 (t, J = 9.7 Hz, 1H), 1.83 (d, J = 12.7 Hz, 1H), 1.75 (d, J = 12.1 Hz, 1H), 1.65 (d, J = 10.7 Hz, 1H), 1.56 (d, J = 13.0 Hz, 1H), 1.38 (d, J = 12.9 Hz, 1H), 1.27-1.15 (m, 1H), 1.14-1.00 (m, 2H), 0.95-0.71 (m, 3H). ^{13}C NMR (101 MHz, CDCl$_3$) δ 140.89, 135.67, 128.24, 127.96, 127.86, 126.25, 124.70, 122.40, 109.74, 74.68, 65.89, 62.99, 59.02, 52.59, 38.26, 32.23, 30.48, 26.26, 26.02, 25.75. ESI-MS: m/z 405.2290 [M + H]$^{+}$.

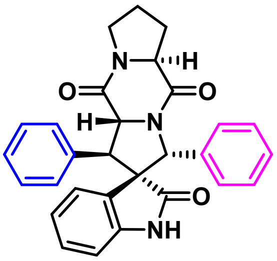

(1R,2S,3R,5aS,10aR)-1,3-diphenyl-1,5a,6,7,8,10a-hexahydro-3H,5H,10H-spiro[dipyrrolo[1,2-a:1',2'-d]pyrazine-2,3'-indoline]-2',5,10-trione (**4a**). Yield: 52%; white solid. m.p. 175~178°C;

^{1}H NMR (400 MHz, $CDCl_3$) δ 7.35 (d, J = 8.3 Hz, 3H), 7.26 (t, J = 7.5 Hz, 2H), 7.11 (dt, J = 19.5, 7.4 Hz, 4H), 7.01 (t, J = 8.7 Hz, 4H), 6.68 (d, J = 7.8 Hz, 1H), 5.41 (s, 1H), 5.19 (d, J = 11.2 Hz, 1H), 4.51 (d, J = 10.6 Hz, 2H), 3.79 (s, 1H), 3.60 (s, 1H), 2.36 (d, J = 29.0 Hz, 2H), 2.06 (s, 2H). ^{13}C NMR (151 MHz, $CDCl_3$) δ 166.12, 164.47, 133.65, 131.65, 131.36, 128.07, 127.33, 126.99, 126.74, 125.54, 122.61, 121.54, 108.93, 66.32, 61.10, 52.41, 50.60, 44.37, 28.68, 21.68, 13.11. ESI-MS: m/z 486.1783 [M + Ma]$^+$.

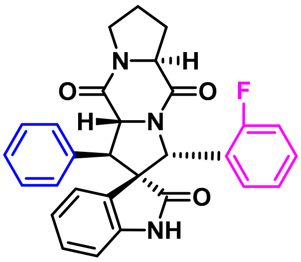

(1R, 2R, 3R, 5aS, 10aR)-3-(2-fluorophenyl)-1-phenyl-1, 5a, 6, 7, 8, 10a-hexahydro-3H, 5H, 10H-spiro[dipyrrolo[1, 2-a:1', 2'-d]pyrazine-2, 3'-indoline]-2', 5, 10-trione (**4b**). Yield: 47%; white solid. m.p. 180~182°C; ^{1}H NMR (400 MHz, DMSO-d_6) δ 10.30 (s, 1H), 7.88 (dd, J = 17.2, 7.4 Hz, 1H), 7.65 (d, J = 7.4 Hz, 1H), 7.55-7.29 (m, 3H), 7.20-7.11 (m, 1H), 7.11-6.96 (m, 6H), 6.93 (d, J = 8.3 Hz, 2H), 6.65 (d, J = 7.7 Hz, 1H), 5.66 (d, J = 11.5 Hz, 1H), 5.15 (s, 1H), 4.81 (t, J = 7.7 Hz, 1H), 4.15 (d, J = 11.4 Hz, 1H), 3.74-3.53 (m, 1H), 3.51-3.37 (m, 1H), 2.22 (dq, J = 12.4, 6.3 Hz, 1H), 2.10-1.98 (m, 1H), 1.91 (qd, J = 12.3, 5.8 Hz, 2H). ^{13}C NMR (151 MHz, $CDCl_3$) δ 182.07, 168.29, 161.33, 140.96, 139.27, 138.60, 129.90, 128.77, 128.11, 127.57, 126.65, 126.19, 124.66, 124.07, 123.36, 115.94, 110.63, 105.97, 67.86, 62.27, 61.99, 57.18, 49.58, 37.22, 28.68, 23.90. ESI-MS: m/z 482.18801 [M + H]$^+$.

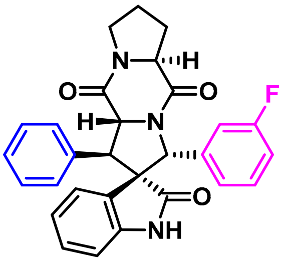

(1R, 2R, 3R, 5aS, 10aR)-3-(3-fluorophenyl)-1-phenyl-1, 5a, 6, 7, 8, 10a-hexahydro-3H, 5H, 10H-spiro[dipyrrolo[1, 2-a:1', 2'-d]pyrazine-2, 3'-indoline]-2', 5, 10-trione (**4c**). Yield: 53%; white solid. m.p. 156~158°C; ^{1}H NMR (600 MHz, $CDCl_3$) δ 7.30 (d, J = 7.4 Hz, 1H), 7.24-7.16 (m, 3H), 7.10-7.03 (m, 1H), 7.00 (d, J = 7.3 Hz, 1H), 6.96 (t, J = 7.4 Hz, 3H), 6.86 (dd, J = 14.9, 7.1 Hz, 2H), 6.76-6.71 (m, 1H), 6.64 (d, J = 9.5 Hz, 1H), 5.30 (s, 1H), 4.45-4.35 (m, 2H), 4.14 (d, J = 13.0 Hz, 1H), 3.75-3.65 (m, 1H), 3.51-3.42 (m, 1H), 2.31-2.18 (m, 2H), 1.92 (dd, J = 14.4, 5.8 Hz, 2H). ^{13}C NMR (151MHz, $CDCl_3$) δ 171.67, 166.32, 164.46, 142.09, 137.99, 131.89, 128.95, 128.75, 128.72, 128.13, 127.76, 127.57, 127.08, 126.87, 126.66, 123.07, 122.32, 121.65, 114.33,

109.38, 67.29, 61.61, 60.05, 50.39, 44.35, 28.67, 24.55, 22.74. ESI-MS: m/z 482.18599 [M + H]$^+$.

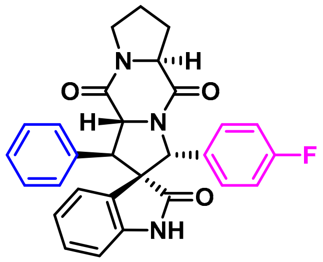

(1R, 2R, 3R, 5aS, 10aR)-3-(4-fluorophenyl)-1-phenyl-1, 5a, 6, 7, 8, 10a-hexahydro-3H, 5H, 10H-spiro[dipyrrolo[1, 2-a:1', 2'-d]pyrazine-2, 3'-indoline]-2', 5, 10-trione (**4d**). Yield: 66%; white solid. m.p. 179~182°C; ^{1}H NMR (400 MHz, DMSO-d_6) δ 10.44 (s, 1H), 7.67 (dd, *J* = 14.2, 7.7 Hz, 2H), 7.57 (t, *J* = 7.8 Hz, 1H), 7.30 (s, 2H), 7.17 (td, *J* = 7.7, 1.2 Hz, 1H), 7.06 (ddd, *J* = 15.5, 7.1, 2.3 Hz, 4H), 6.98 (dd, *J* = 6.8, 3.0 Hz, 2H), 6.65 (d, *J* = 7.7 Hz, 1H), 5.72 (d, *J* = 11.4 Hz, 1H), 5.35 (s, 1H), 4.86 (t, *J* = 7.5 Hz, 1H), 4.13 (d, *J* = 11.3 Hz, 1H), 3.59 (dt, *J* = 11.1, 7.1 Hz, 1H), 3.43 (ddd, *J* = 11.8, 7.4, 5.2 Hz, 1H), 2.23 (dq, *J* = 12.9, 6.3 Hz, 1H), 2.11-2.02 (m, 1H), 1.94 (dt, *J* = 14.7, 7.1 Hz, 1H), 1.83 (d, *J* = 25.7 Hz, 1H), 1.39-1.23 (m, 1H). ^{13}C NMR (101 MHz, DMSO-d$_6$) δ 173.92, 168.10, 166.42, 141.45, 139.58, 134.57, 131.04, 129.58, 129.13, 128.64, 128.24, 127.65, 124.78, 122.19, 110.05, 67.78, 62.24, 60.27, 50.16, 45.54, 27.06, 24.04, 21.27, 14.60. ESI-MS: m/z 482.18656 [M + H]$^+$.

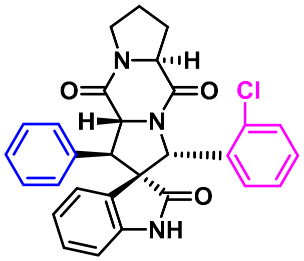

(1R, 2R, 3R, 5aS, 10aR)-3-(2-chlorophenyl)-1-phenyl-1, 5a, 6, 7, 8, 10a-hexahydro-3H, 5H, 10H-spiro[dipyrrolo[1, 2-a:1', 2'-d]pyrazine-2, 3'-indoline]-2', 5, 10-trione (**4e**). Yield: 51%; white solid. m.p. 188~190°C; ^{1}H NMR (600 MHz, CDCl$_3$) δ 7.38 (dd, *J* = 10.6, 7.4 Hz, 1H), 7.25-7.18 (m, 5H), 7.09 (t, *J* = 7.5 Hz, 1H), 7.02-6.97 (m, 1H), 6.94 (d, *J* = 7.6 Hz, 2H), 6.82 (t, *J* = 6.8 Hz, 2H), 6.77 (d, *J* = 7.8 Hz, 1H), 5.71 (s, 1H), 5.04 (d, *J* = 11.6 Hz, 1H), 4.43 (t, *J* = 7.9 Hz, 1H), 3.91 (t, *J* = 12.1 Hz, 1H), 2.27 (ddt, *J* = 38.6, 13.8, 7.4 Hz, 2H), 0.80 (dd, *J* = 18.8, 11.9 Hz, 4H). ^{13}C NMR (151MHz, CDCl$_3$) δ 172.03, 166.13, 142.47, 132.43, 132.06, 131.48, 128.40, 128.27, 128.08, 127.71, 127.53, 126.97, 126.77, 126.12, 125.18, 122.18, 121.51, 109.15, 64.28, 61.48, 60.12, 50.29, 44.33, 28.67, 26.51, 24.59 . ESI-MS: m/z 498.15868 [M + H]$^+$.

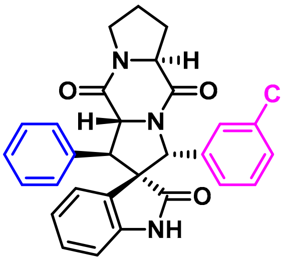

(1R, 2R, 3R, 5aS, 10aR)-3-(3-chlorophenyl)-1-phenyl-1, 5a, 6, 7, 8, 10a-hexahydro-3H, 5H, 10H-spiro[dipyrrolo[1, 2-a:1', 2'-d]pyrazine-2, 3'-indoline]-2', 5, 10-trione (**4f**). Yield: 77%; white solid. m.p. 175~177°C; ^{1}H NMR (600 MHz, $CDCl_3$) δ 8.23 (s, 1H), 7.25-7.23 (m, 1H), 7.14-7.08 (m, 3H), 7.02-6.98 (m, 3H), 6.95-6.91 (m, 3H), 6.90-6.87 (m, 3H), 5.22 (s, 1H), 5.08 (d, J = 11.2 Hz, 1H), 4.43-4.32 (m, 2H), 3.68 (dt, J = 11.2, 7.7 Hz, 1H), 3.52-3.43 (m, 1H), 2.30 (ddd, J = 38.2, 12.9, 6.5 Hz, 2H), 1.83-1.73 (m, 1H), 1.64 (s, 1H). ^{13}C NMR (151MHz, $CDCl_3$) δ 172.92, 164.46, 138.97, 137.46, 133.33, 132.03, 128.69, 128.11, 127.44, 127.39, 127.29, 127.10, 126.83, 122.61, 121.59, 109.32, 60.04, 59.85, 53.53, 49.91, 44.42, 28.67, 26.46, 22.76. ESI-MS: m/z 498.15932 [M + H]$^{+}$.

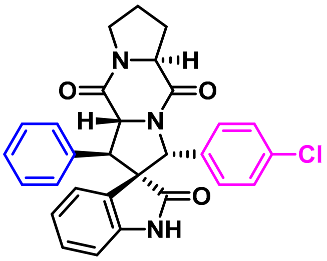

(1R, 2R, 3R, 5aS, 10aR)-3-(4-chlorophenyl)-1-phenyl-1, 5a, 6, 7, 8, 10a-hexahydro-3H, 5H, 10H-spiro[dipyrrolo[1, 2-a:1', 2'-d]pyrazine-2, 3'-indoline]-2', 5, 10-trione (**4g**). Yield: 72%; white solid. m.p. 200~202°C; ^{1}H NMR (600 MHz, $CDCl_3$) δ 8.15 (s, 1H), 7.27-7.22 (m, 2H), 7.10 (t, J = 7.7 Hz, 1H), 6.99 (dt, J = 14.2, 7.2 Hz, 3H), 6.89 (dt, J = 8.4, 6.2 Hz, 7H), 5.22 (s, 1H), 5.08 (d, J = 11.1 Hz, 1H), 4.05 (q, J = 7.1 Hz, 2H), 3.47 (ddd, J = 12.2, 8.0, 4.7 Hz, 2H), 2.25 (dtd, J = 11.9, 8.2, 7.3, 3.0 Hz, 2H), 2.22-2.15 (m, 2H). ^{13}C NMR (151MHz, $CDCl_3$) δ 173.07, 166.29, 138.94, 135.21, 132.22, 129.65, 127.92, 127.45, 127.40, 127.03, 126.67, 122.68, 121.51, 109.40, 67.87, 60.10, 59.39, 49.65, 44.33, 28.68, 22.77, 20.04. ESI-MS: m/z 498.15844 [M + H]$^{+}$.

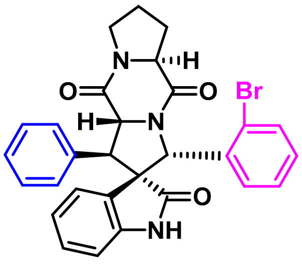

(1R, 2R, 3R, 5aS, 10aR)-3-(2-bromophenyl)-1-phenyl-1, 5a, 6, 7, 8, 10a-hexahydro-3H, 5H, 10H-spiro[dipyrrolo[1, 2-a:1', 2'-d]pyrazine-2, 3'-indoline]-2', 5, 10-trione (**4h**). Yield: 59%;

white solid. m.p. 167~170°C; ^{1}H NMR (600 MHz, $CDCl_3$) δ 8.47 (s, 1H), 7.31-7.28 (m, 1H), 7.24-7.22 (m, 2H), 7.13-7.08 (m, 2H), 6.99 (dd, J = 10.2, 5.0 Hz, 3H), 6.93-6.89 (m, 5H), 5.23 (s, 1H), 5.08 (d, J = 11.2 Hz, 1H), 4.18 (d, J = 12.0 Hz, 1H), 4.04 (q, J = 7.1 Hz, 1H), 3.71-3.62 (m, 1H), 3.49-3.41 (m, 1H), 2.36-2.13 (m, 4H). ^{13}C NMR (151MHz, $CDCl_3$) δ 173.14, 164.49, 139.03, 137.76, 137.17, 132.02, 130.18, 129.31, 128.94, 128.09, 127.45, 127.38, 127.10, 126.81, 126.72, 126.59, 123.28, 122.58, 121.57, 109.44, 67.20, 59.40, 53.49, 49.80, 44.44, 26.45, 22.77, 13.18. ESI-MS: m/z 542.10605 [M + H]$^+$.

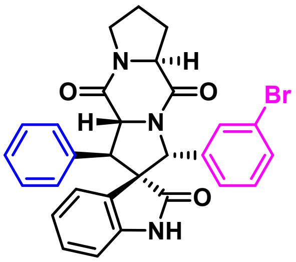

(1R, 2R, 3R, 5aS, 10aR)-3-(3-bromophenyl)-1-phenyl-1, 5a, 6, 7, 8, 10a-hexahydro-3H, 5H, 10H-spiro[dipyrrolo[1, 2-a:1', 2'-d]pyrazine-2, 3'-indoline]-2', 5, 10-trione (**4i**). Yield: 67%; white solid. m.p. 183~185°C; ^{1}H NMR (600 MHz, $CDCl_3$) δ 7.42 (t, J = 9.3 Hz, 1H), 7.35 (dd, J = 20.1, 7.7 Hz, 2H), 7.28 (q, J = 7.7 Hz, 1H), 7.13 (q, J = 7.9 Hz, 2H), 7.10-7.03 (m, 1H), 7.01-6.97 (m, 1H), 6.96-6.89 (m, 3H), 6.83 (d, J = 7.7 Hz, 2H), 5.74 (s, 1H), 5.08 (d, J = 11.5 Hz, 1H), 4.42 (t, J = 8.0 Hz, 1H), 4.23 (d, J = 11.7 Hz, 1H), 3.69 (dt, J = 11.6, 7.6 Hz, 1H), 3.53-3.44 (m, 1H), 2.47-2.35 (m, 1H), 2.30 (dt, J = 13.8, 6.4 Hz, 1H), 2.27-2.17 (m, 2H). ^{13}C NMR (151MHz, $CDCl_3$) δ 172.83, 164.47, 138.99, 133.65, 131.64, 131.37, 128.47, 128.06, 127.33, 127.00, 126.74, 126.24, 125.54, 123.03, 122.61, 121.53, 108.94, 66.32, 61.10, 59.85, 50.59, 28.68, 21.68, 13.11. ESI-MS: m/z 542.26679 [M + H]$^+$.

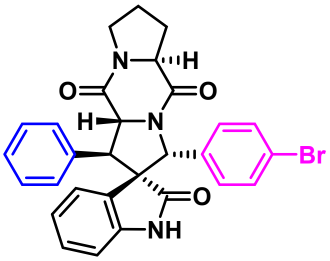

(1R, 2R, 3R, 5aS, 10aR)-3-(4-bromophenyl)-1-phenyl-1, 5a, 6, 7, 8, 10a-hexahydro-3H, 5H, 10H-spiro[dipyrrolo[1, 2-a:1', 2'-d]pyrazine-2, 3'-indoline]-2', 5, 10-trione (**4j**). Yield: 72%; white solid. m.p. 191~193°C; ^{1}H NMR (600 MHz, $CDCl_3$) δ 8.21 (s, 1H), 7.27 (d, J = 7.5 Hz, 2H), 7.12 (dd, J = 16.7, 7.7 Hz, 2H), 7.07-7.00 (m, 5H), 6.88 (d, J = 7.6 Hz, 2H), 6.78-6.72 (m, 3H), 5.31 (s, 1H), 5.02 (d, J = 11.3 Hz, 1H), 4.39 (t, J = 7.9 Hz, 1H), 4.34 (d, J = 11.3 Hz, 1H), 3.72-3.61 (m, 1H), 3.52-3.42 (m, 1H), 2.36-2.22 (m, 2H), 1.93-1.83 (m, 2H). ^{13}C NMR (151MHz, $CDCl_3$) δ 172.80, 166.17, 138.91, 135.11, 131.29, 130.23, 129.93, 129.27, 129.12, 128.20, 127.50,

127.26, 123.24, 122.56, 121.70, 120.90, 109.65, 68.52, 60.13, 52.82, 49.26, 44.39, 28.68, 26.45, 22.74. ESI-MS: m/z 542.10791 [M + H]$^+$.

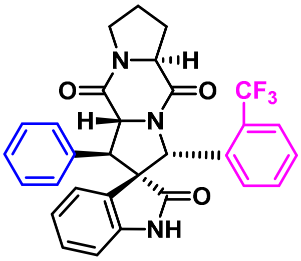

(1R, 2R, 3R, 5aS, 10aR)-3-(2-(trifluoromethyl)phenyl)-1-phenyl-1, 5a, 6, 7, 8, 10a-hexahydro-3H, 5H, 10H-spiro[dipyrrolo[1, 2-a:1', 2'-d]pyrazine-2, 3'-indoline]-2', 5, 10-trione (**4k**). Yield: 41%; white solid. m.p. 166~168°C; ^{1}H NMR (400 MHz, DMSO-d_6) δ 10.37 (d, J = 63.0 Hz, 1H), 8.17 (dd, J = 26.3, 8.7 Hz, 2H), 7.73 (t, J = 8.1 Hz, 1H), 7.41-6.84 (m, 9H), 6.62 (dd, J = 32.2, 7.7 Hz, 1H), 5.72 (dd, J = 24.8, 11.9 Hz, 1H), 5.36 (s, 1H), 4.84 (t, J = 7.8 Hz, 1H), 4.20-4.06 (m, 1H), 3.75-3.58 (m, 1H), 3.27-3.10 (m, 1H), 2.29-1.83 (m, 4H). ^{13}C NMR (151 MHz,) δ 183.01, 169.96, 139.85, 138.58, 134.63, 132.09, 129.99, 129.25, 128.28, 127.16, 126.65, 124.99, 124.85, 123.67, 122.04, 107.86, 67.83, 64.31, 63.17, 61.53, 49.42, 35.88, 28.69, 22.04. ESI-MS: m/z 532.18523 [M + H]$^+$.

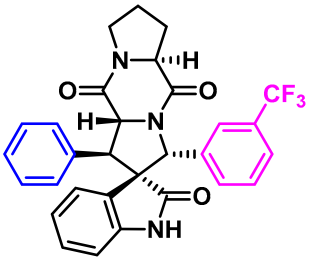

(1R, 2R, 3R, 5aS, 10aR)-3-(3-(trifluoromethyl)phenyl)-1-phenyl-1, 5a, 6, 7, 8, 10a-hexahydro-3H, 5H, 10H-spiro[dipyrrolo[1, 2-a:1', 2'-d]pyrazine-2, 3'-indoline]-2', 5, 10-trione (**4l**). Yield: 53%; white solid. m.p. 195~198°C; ^{1}H NMR (600 MHz, $CDCl_3$) δ 8.52 (s, 1H), 7.43 (t, J = 7.1 Hz, 1H), 7.23 (q, J = 7.6, 6.6 Hz, 2H), 7.06 (dd, J = 18.3, 8.6 Hz, 2H), 6.99 (q, J = 7.3, 6.5 Hz, 3H), 6.96-6.90 (m, 3H), 6.87 (d, J = 9.1 Hz, 2H), 5.33 (s, 1H), 5.10 (d, J = 11.1 Hz, 1H), 4.39 (t, J = 7.8 Hz, 1H), 4.32 (d, J = 11.0 Hz, 1H), 3.65 (dd, J = 13.1, 6.1 Hz, 1H), 3.45 (dd, J = 9.5, 4.9 Hz, 1H), 2.24 (p, J = 6.7 Hz, 1H), 2.17 (dq, J = 14.5, 7.9 Hz, 1H), 1.90 (p, J = 8.3, 7.2 Hz, 2H). ^{13}C NMR (151MHz, $CDCl_3$) δ 173.07, 164.53, 139.06, 136.34, 131.95, 129.12, 128.16, 127.90, 127.38, 127.09, 126.82, 126.61, 123.93, 123.37, 122.61, 121.61, 109.31, 67.24, 60.02, 52.46, 49.59, 44.46, 28.68, 26.42, 22.73. ESI-MS: m/z 532.18479 [M + H]$^+$.

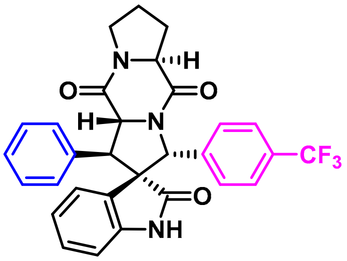

(1R, 2R, 3R, 5aS, 10aR)-3-(4-(trifluoromethyl)phenyl)-1-phenyl-1, 5a, 6, 7, 8, 10a-hexahydro-3H, 5H, 10H-spiro[dipyrrolo[1, 2-a:1', 2'-d]pyrazine-2, 3'-indoline]-2', 5, 10-trione (**4m**). Yield: 68%; white solid. m.p. 201~203°C; ^{1}H NMR (600 MHz, $CDCl_3$) δ 7.93 (s, 1H), 7.24 (d, *J* = 7.5 Hz, 1H), 7.18-7.12 (m, 3H), 7.02 (dt, *J* = 14.0, 7.3 Hz, 2H), 6.94 (dd, *J* = 12.0, 6.7 Hz, 2H), 6.88 (d, *J* = 7.9 Hz, 2H), 6.82 (d, *J* = 7.6 Hz, 2H), 5.27 (s, 1H), 5.07 (d, *J* = 11.0 Hz, 1H), 4.40 (dd, *J* = 14.7, 6.9 Hz, 2H), 3.72-3.63 (m, 1H), 3.52-3.43 (m, 1H), 2.31-2.25 (m, 1H), 2.19 (dd, *J* = 21.7, 8.6 Hz, 1H), 1.97-1.90 (m, 2H). ^{13}C NMR (151MHz, $CDCl_3$) δ 166.27, 164.42, 133.80, 132.92, 131.93, 129.31, 128.21, 127.74, 127.39, 127.11, 126.82, 122.70, 121.74, 109.44, 61.30, 60.09, 52.41, 49.98, 44.38, 28.68, 21.68, 13.11. ESI-MS: m/z 532.18582 [M + H]$^{+}$.

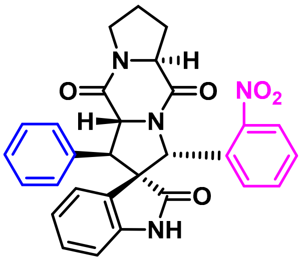

(1R, 2R, 3R, 5aS, 10aR)-3-(2-nitrophenyl)-1-phenyl-1, 5a, 6, 7, 8, 10a-hexahydro-3H, 5H, 10H-spiro[dipyrrolo[1, 2-a:1', 2'-d]pyrazine-2, 3'-indoline]-2', 5, 10-trione (**4n**). Yield: 54%; white solid. m.p. 186~189°C; ^{1}H NMR (600 MHz, $CDCl_3$) δ 8.08 (s, 1H), 7.64 (t, *J* = 7.6 Hz, 1H), 7.46 (t, *J* = 7.8 Hz, 1H), 7.32 (d, *J* = 7.4 Hz, 1H), 7.26 (s, 1H), 7.17-7.12 (m, 2H), 7.06 (t, *J* = 7.6 Hz, 1H), 7.01 (d, *J* = 7.3 Hz, 1H), 6.95 (t, *J* = 7.6 Hz, 3H), 6.82 (d, *J* = 7.8 Hz, 2H), 5.87 (s, 1H), 5.14 (d, *J* = 11.5 Hz, 1H), 4.44 (t, *J* = 7.9 Hz, 1H), 4.23 (d, *J* = 11.5 Hz, 1H), 3.73-3.67 (m, 1H), 3.53-3.48 (m, 1H), 2.30 (dq, *J* = 12.6, 7.2 Hz, 1H), 2.20 (dd, *J* = 14.2, 8.0 Hz, 1H), 1.97-1.93 (m, 2H). ^{13}C NMR (151MHz, $CDCl_3$) δ 173.21, 164.37, 146.44, 139.20, 133.30, 131.93, 131.09, 128.86, 128.24, 128.03, 127.25, 127.00, 126.83, 126.16, 124.70, 122.26, 121.55, 109.10, 63.33, 60.12, 52.43, 50.69, 44.39, 28.68, 26.42, 22.80. ESI-MS: m/z 509.18365 [M + H]$^{+}$.

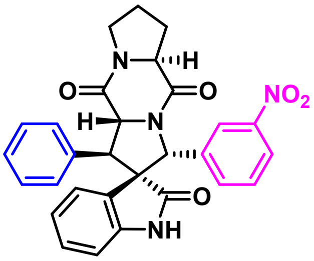

(1R, 2R, 3R, 5aS, 10aR)-3-(3-nitrophenyl)-1-phenyl-1, 5a, 6, 7, 8, 10a-hexahydro-3H, 5H, 10H-spiro[dipyrrolo[1, 2-a:1', 2'-d]pyrazine-2, 3'-indoline]-2', 5, 10-trione (**4o**). Yield: 63%; white solid. m.p. 191~193°C; ^{1}H NMR (600 MHz, $CDCl_3$) δ 8.14-8.03 (m, 1H), 7.86-7.73 (m, 1H), 7.52-7.32 (m, 2H), 7.31-7.21 (m, 2H), 7.13 (dd, J = 10.6, 7.1 Hz, 1H), 7.08-6.98 (m, 2H), 6.95 (dt, J = 12.3, 5.8 Hz, 2H), 6.88 (d, J = 8.9 Hz, 1H), 6.83 (dd, J = 10.2, 7.8 Hz, 1H), 5.42 (d, J = 5.8 Hz, 1H), 5.10 (dd, J = 33.8, 11.2 Hz, 1H), 4.44 (t, J = 7.8 Hz, 1H), 4.32 (dd, J = 11.2, 7.4 Hz, 1H), 3.48 (td, J = 10.4, 8.8, 6.4 Hz, 1H), 3.37-3.30 (m, 1H), 2.29 (dq, J = 13.1, 6.6 Hz, 1H), 2.21 (td, J = 14.8, 13.2, 7.9 Hz, 1H), 1.95 (td, J = 11.7, 10.7, 7.9 Hz, 2H). ^{13}C NMR (151MHz, $CDCl_3$) δ 166.53, 164.33, 147.16, 141.30, 138.75, 137.40, 131.64, 128.49, 128.39, 127.69, 127.32, 127.16, 126.86, 122.74, 122.38, 122.15, 121.87, 109.34, 66.70, 65.43, 60.00, 49.28, 44.55, 28.68, 26.41, 22.78. ESI-MS: m/z 509.18314 [M + H]$^+$.

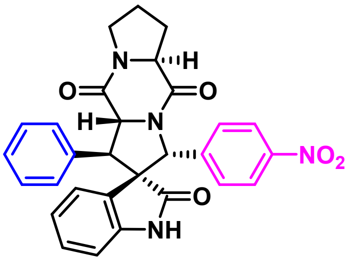

(1R, 2R, 3R, 5aS, 10aR)-3-(4-nitrophenyl)-1-phenyl-1, 5a, 6, 7, 8, 10a-hexahydro-3H, 5H, 10H-spiro[dipyrrolo[1, 2-a:1', 2'-d]pyrazine-2, 3'-indoline]-2', 5, 10-trione (**4p**). Yield: 75%; white solid. m.p. 176~179°C; ^{1}H NMR (400 MHz, $CDCl_3$) δ 8.22 (d, J = 8.3 Hz, 2H), 7.62 (s, 1H), 7.35 (d, J = 7.5 Hz, 1H), 7.24 (t, J = 7.6 Hz, 3H), 7.19-7.05 (m, 4H), 6.99 (d, J = 7.5 Hz, 2H), 6.67 (d, J = 7.8 Hz, 1H), 5.50 (s, 1H), 5.23 (d, J = 11.0 Hz, 1H), 4.54 (t, J = 7.9 Hz, 1H), 4.48 (d, J = 11.0 Hz, 1H), 3.84-3.72 (m, 1H), 3.66-3.56 (m, 1H), 2.40 (dt, J = 12.6, 6.5 Hz, 1H), 2.36-2.23 (m, 1H), 2.07 (d, J = 10.0 Hz, 2H).^{13}C NMR (151 MHz, $CDCl_3$) δ 182.10, 169.57, 146.57, 145.32, 140.29, 138.68, 137.30, 128.89, 128.35, 127.89, 127.73, 125.47, 125.00, 124.50, 123.56, 110.68, 68.35, 64.12, 63.74, 62.94, 50.56, 39.52, 30.91, 28.68. ESI-MS: m/z 509.18190 [M + H]$^+$.

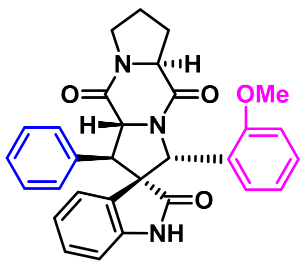

(1R, 2R, 3R, 5aS, 10aR)-3-(2-methoxyphenyl)-1-phenyl-1, 5a, 6, 7, 8, 10a-hexahydro-3H, 5H, 10H-spiro[dipyrrolo[1, 2-a:1', 2'-d]pyrazine-2, 3'-indoline]-2', 5, 10-trione (**4q**). Yield: 46%; white solid. m.p. 213~215°C; ^{1}H NMR (600 MHz, $CDCl_3$) δ 7.33-7.21 (m, 1H), 7.19-7.05 (m, 2H), 7.02-6.90 (m, 4H), 6.91-6.82 (m, 2H), 6.82-6.68 (m, 2H), 6.56-6.43 (m, 2H), 5.36-5.26 (m, 1H), 5.13-4.97 (m, 1H), 4.49-4.38 (m, 1H), 4.15 (d, *J* = 13.2 Hz, 1H), 4.06-3.94 (m, 1H), 3.65 (s, 3H), 3.46 (ddt, *J* = 12.1, 7.8, 3.8 Hz, 1H), 2.34-2.16 (m, 2H), 1.94 (dt, *J* = 11.2, 6.0 Hz, 2H). ESI-MS: m/z 494.20764 [M + H]$^{+}$. ^{13}C NMR (101 MHz, $CDCl_3$) δ 173.82, 165.67, 159.33, 139.85, 133.47, 130.72, 128.98, 128.48, 128.16, 127.70, 123.87, 122.61, 113.96, 110.14, 68.67, 62.43, 62.07, 61.22, 55.16, 50.72, 45.43, 27.55, 23.86.

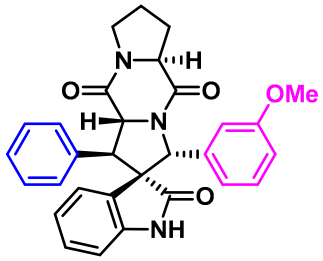

(1R, 2R, 3R, 5aS, 10aR)-3-(3-methoxyphenyl)-1-phenyl-1, 5a, 6, 7, 8, 10a-hexahydro-3H, 5H, 10H-spiro[dipyrrolo[1, 2-a:1', 2'-d]pyrazine-2, 3'-indoline]-2', 5, 10-trione (**4r**). Yield: 65%; white solid. m.p. 182~185°C; ^{1}H NMR (400 MHz, $CDCl_3$) δ 7.47-7.31 (m, 1H), 7.26-7.18 (m, 1H), 7.17-7.01 (m, 6H), 6.97 (d, *J* = 8.2 Hz, 2H), 6.87 (dd, *J* = 12.4, 8.4 Hz, 2H), 6.64 (d, *J* = 7.7 Hz, 1H), 5.39 (s, 1H), 5.13 (dd, *J* = 28.0, 10.9 Hz, 1H), 4.52 (dt, *J* = 15.2, 9.4 Hz, 2H), 3.84-3.75 (m, 4H), 3.58 (ddd, *J* = 12.0, 7.8, 4.4 Hz, 1H), 2.34 (tt, *J* = 13.1, 6.6 Hz, 2H), 2.07-1.94 (m, 2H), 1.66 (s, 1H). ^{13}C NMR (151 MHz, $CDCl_3$) δ 181.89, 167.27, 159.29, 142.18, 139.80, 137.75, 133.41, 130.68, 128.95, 12844, 128.13, 127.68, 123.84, 122.58, 113.91, 110.41, 105.17, 68.61, 62.39, 61.18, 55.14, 50.69, 45.39, 27.51, 23.83, 14.22. ESI-MS: m/z 494.20786 [M + H]$^{+}$.

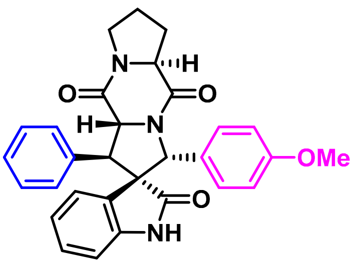

(1R, 2R, 3R, 5aS, 10aR)-3-(4-methoxyphenyl)-1-phenyl-1, 5a, 6, 7, 8, 10a-hexahydro-3H, 5H, 10H-spiro[dipyrrolo[1, 2-a:1', 2'-d]pyrazine-2, 3'-indoline]-2', 5, 10-trione (**4s**). Yield: 61%; white solid. m.p. 204~206°C; ^{1}H NMR (600 MHz, $CDCl_3$) δ 7.33-7.22 (m, 1H), 7.18-7.12 (m, 1H), 7.10-7.04 (m, 1H), 7.03-6.91 (m, 6H), 6.91-6.84 (m, 2H), 6.83-6.71 (m, 2H), 5.27 (d, *J* = 4.1 Hz, 1H), 4.47-4.35 (m, 2H), 4.02-3.94 (m, 1H), 3.66 (d, *J* = 8.5 Hz, 3H), 3.61 (d, *J* = 11.3 Hz, 1H), 3.52-3.42 (m, 1H), 2.30-2.19 (m, 2H), 1.96-1.92 (m, 2H). ^{13}C NMR (151MHz, $CDCl_3$) δ 166.26, 164.67, 158.46, 136.26, 132.28, 132.14, 129.68, 129.02, 128.42, 128.16, 127.81, 127.02, 126.77, 126.60, 122.36, 121.43, 112.53, 109.23, 67.81, 61.67, 54.00, 52.43, 50.37, 44.36, 26.43, 22.79, 13.12. ESI-MS: m/z 494.20615 [M + H]$^{+}$.

4. Spectrums of azomethine ylide 1a-1s

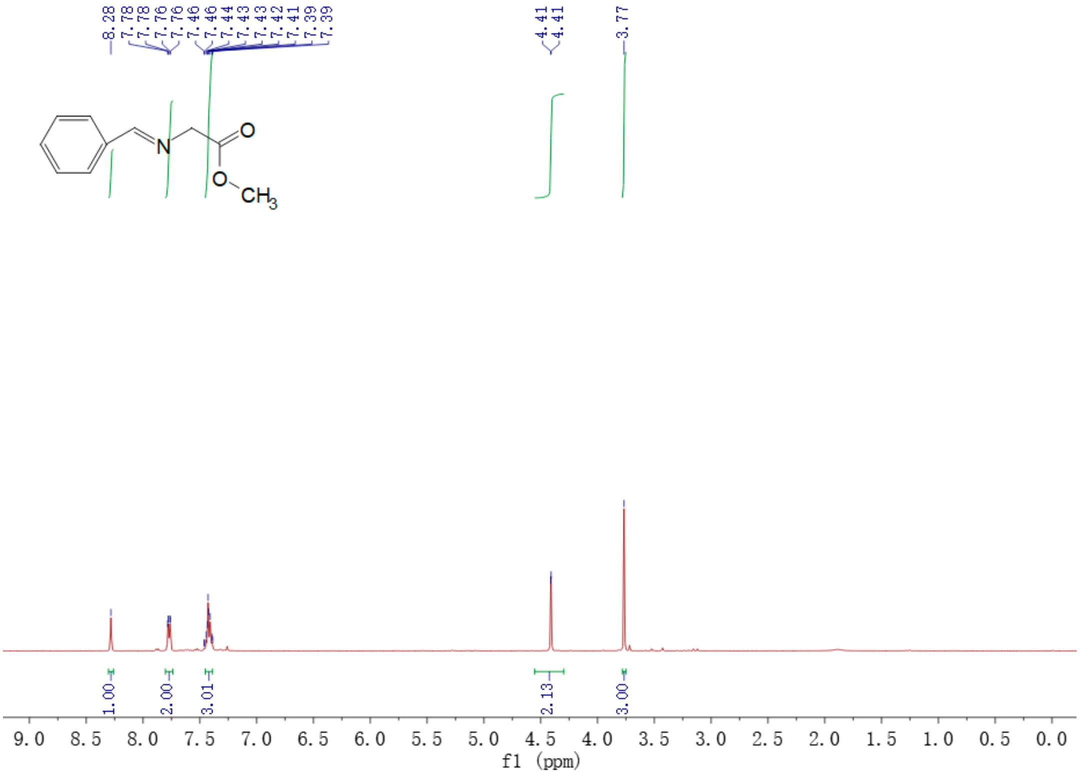

Fig. S1 The ^{1}H NMR (400 MHz, $CDCl_3$) of **1a**

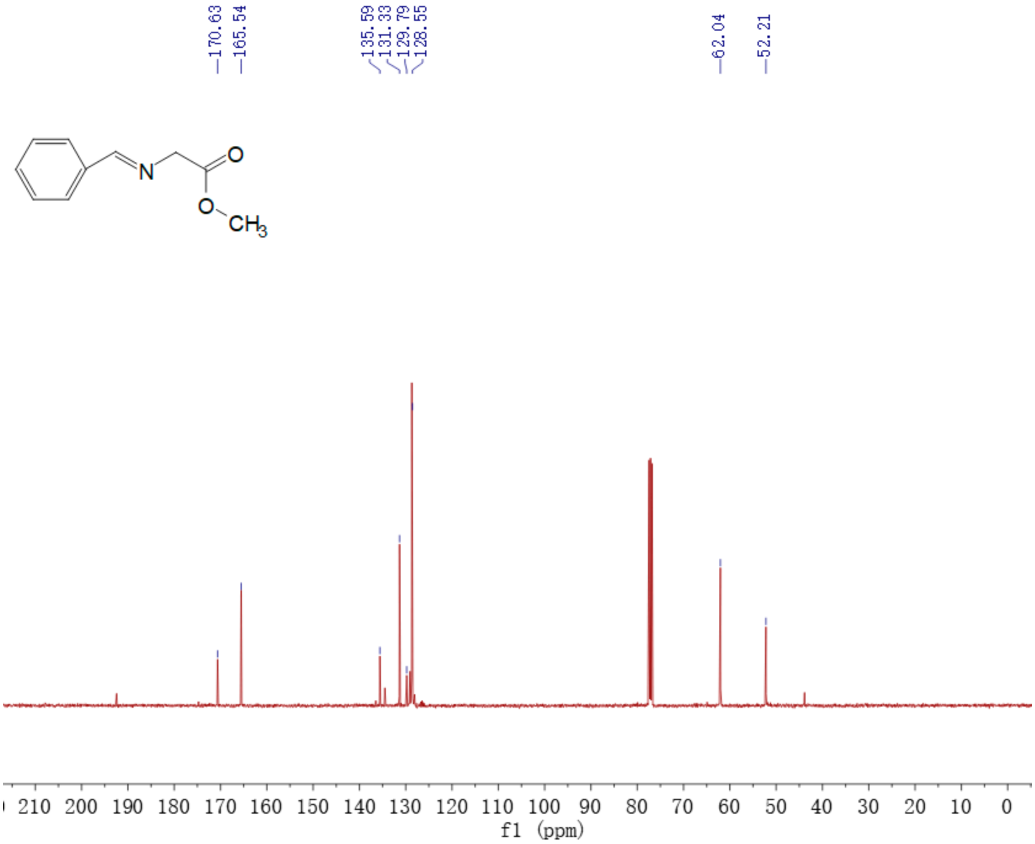

Fig. S2 The ^{13}C NMR (101 MHz, $CDCl_3$) of **1a**

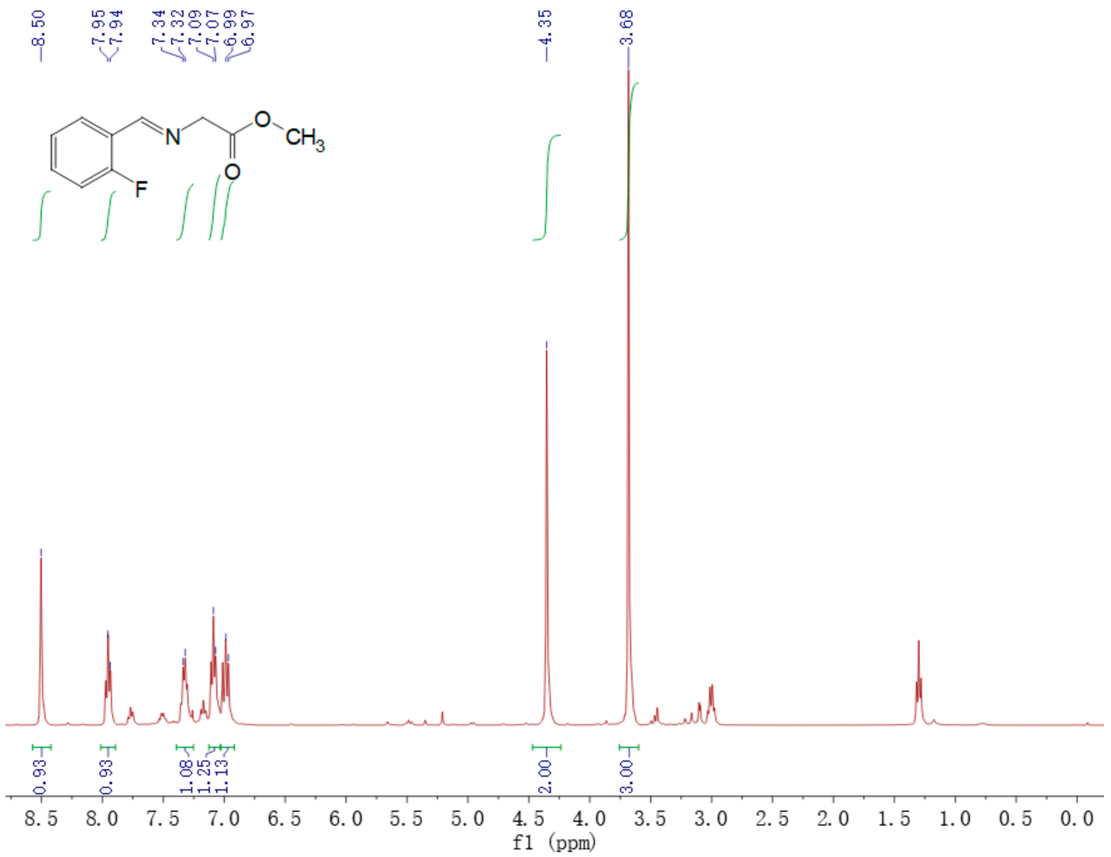

Fig. S3 The ^{1}H NMR (400 MHz, CDCl3) of **1b**

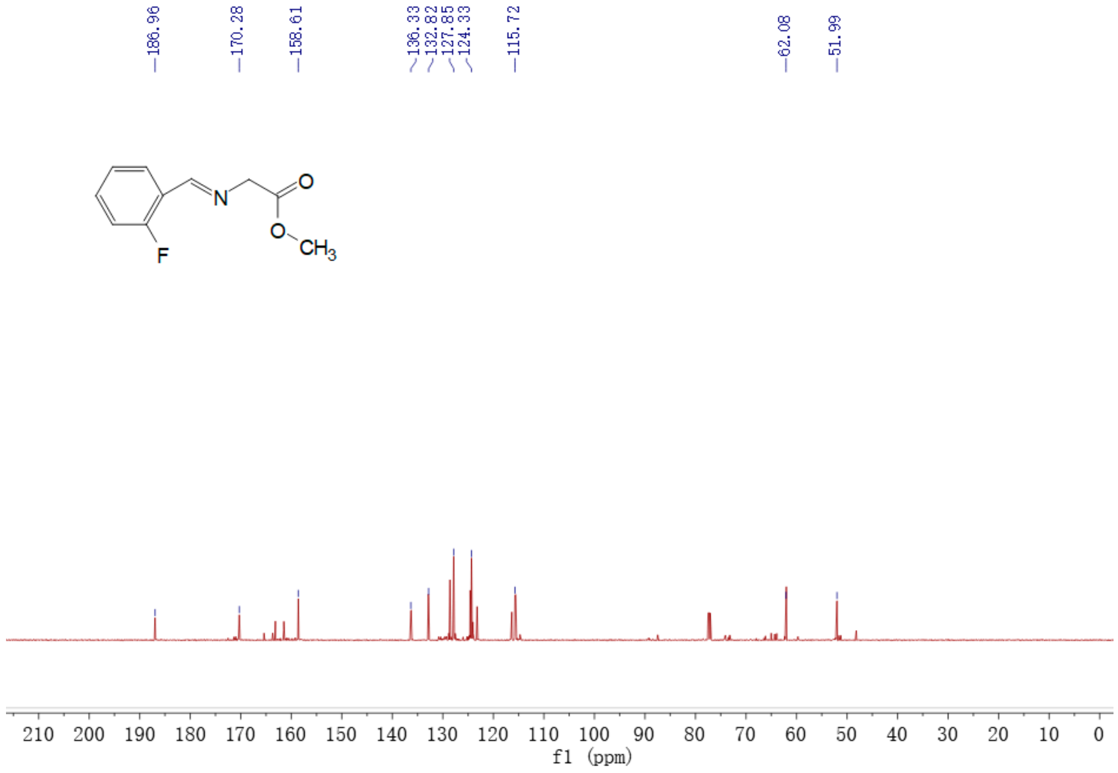

Fig. S4 The ^{13}C NMR (151 MHz, CDCl3) of **1b**

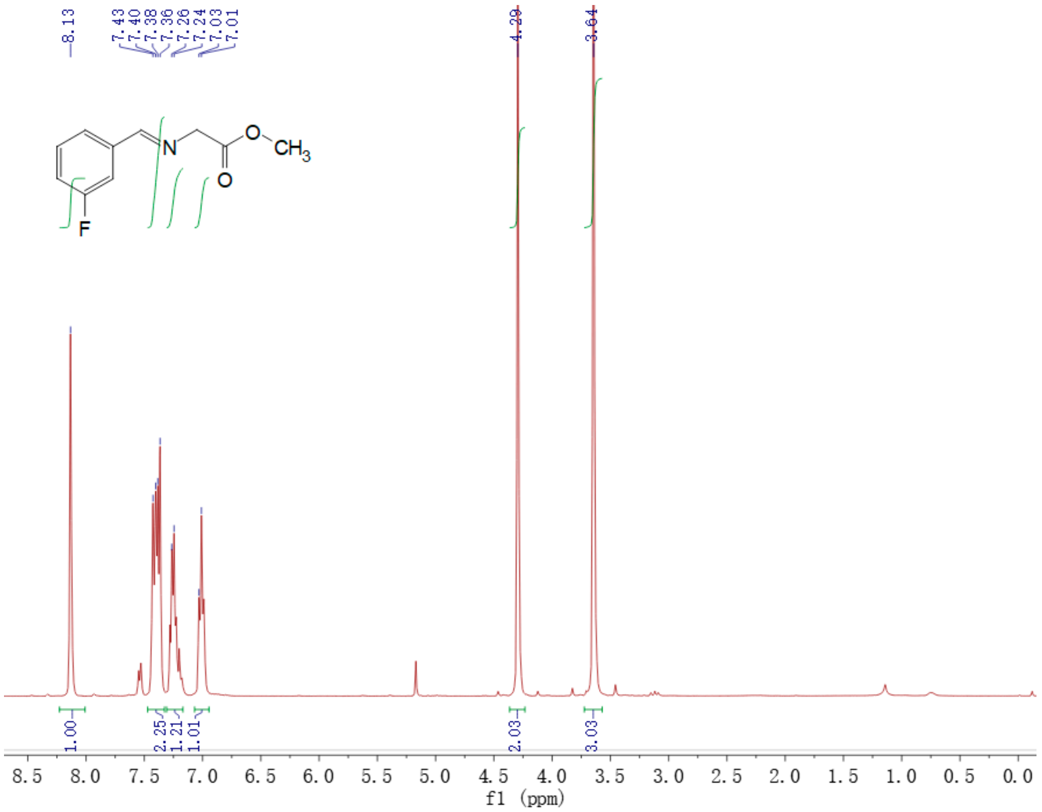

Fig. S5 The ^{1}H NMR (400 MHz, CDCl3) of **1c**

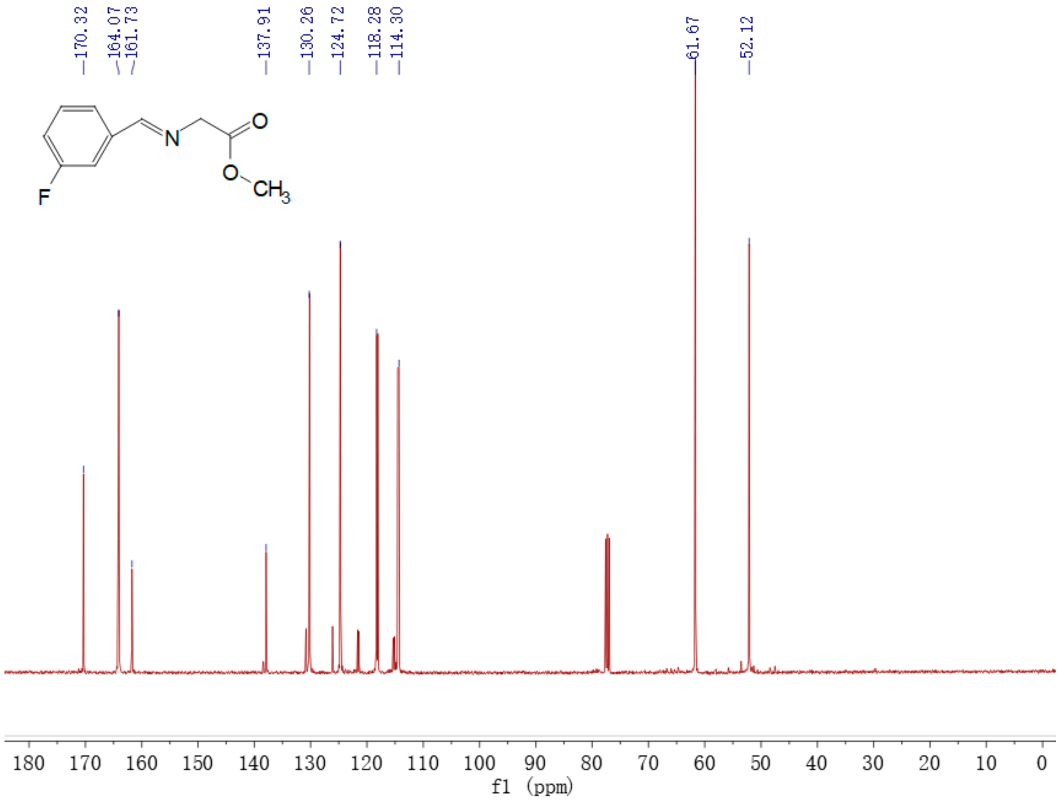

Fig. S6 The ^{13}C NMR (101 MHz, CDCl3) of **1c**

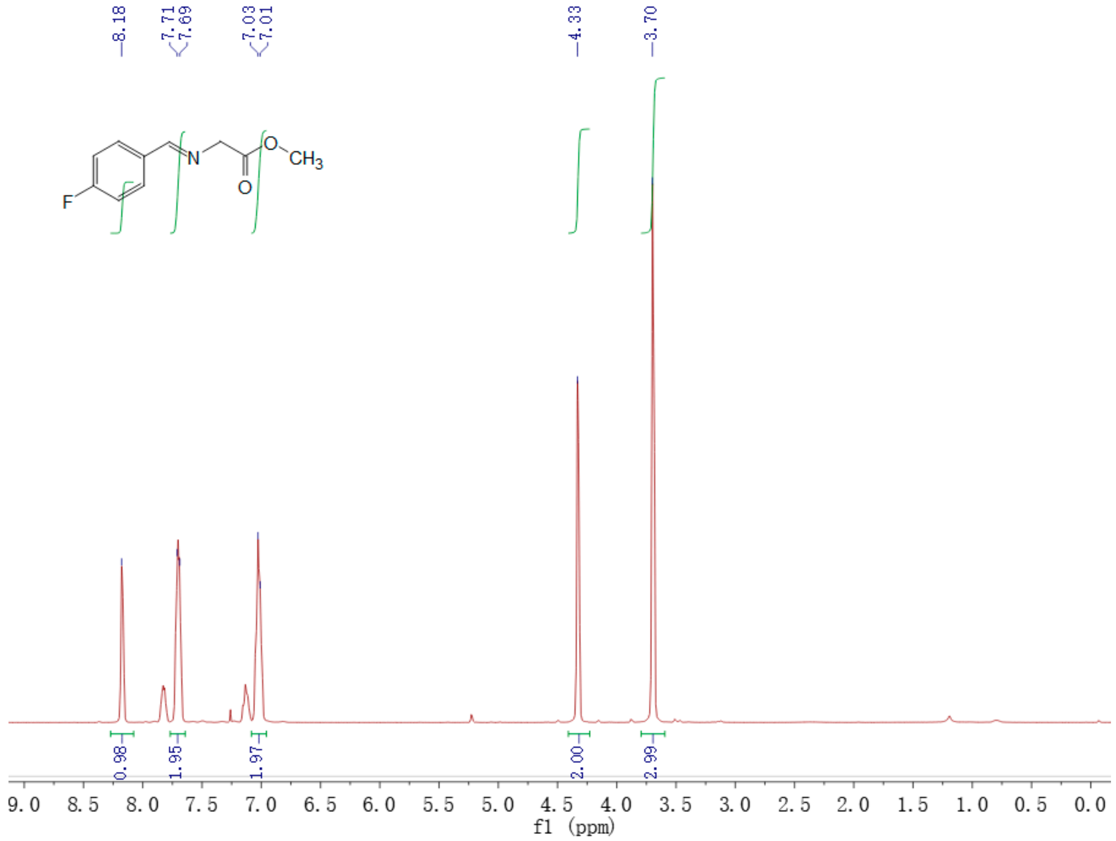

Fig. S7 The ^{1}H NMR (400 MHz, CDCl3) of **1d**

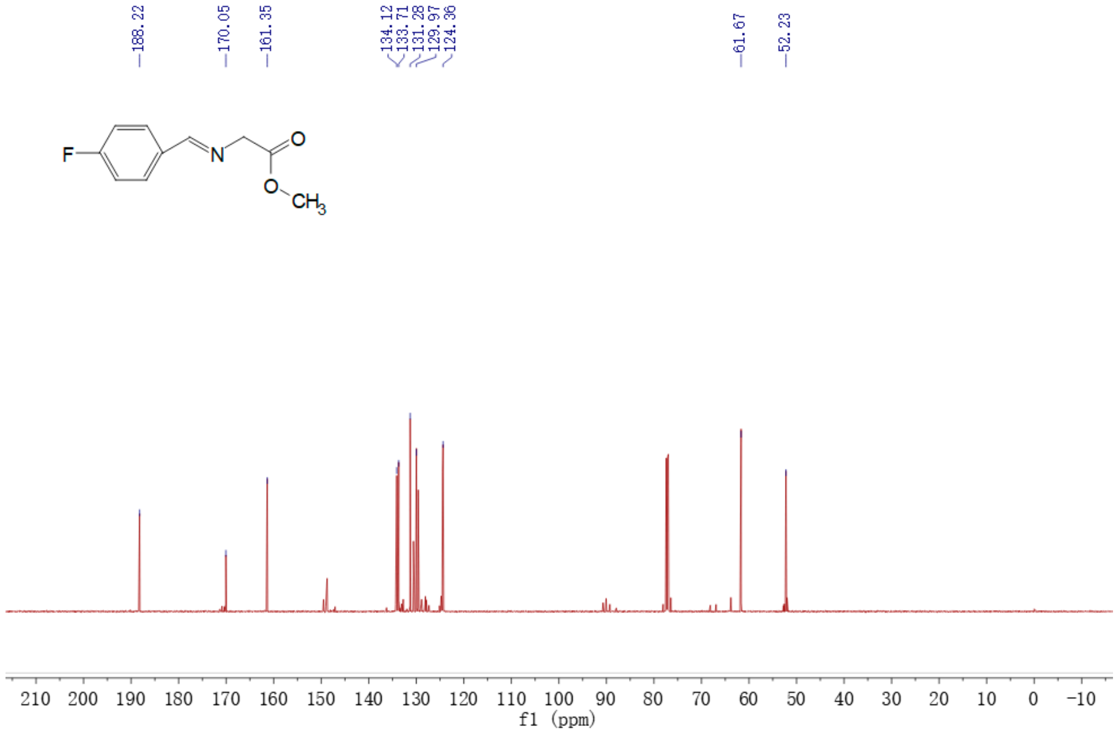

Fig. S8 The ^{13}C NMR (151 MHz, CDCl3) of **1d**

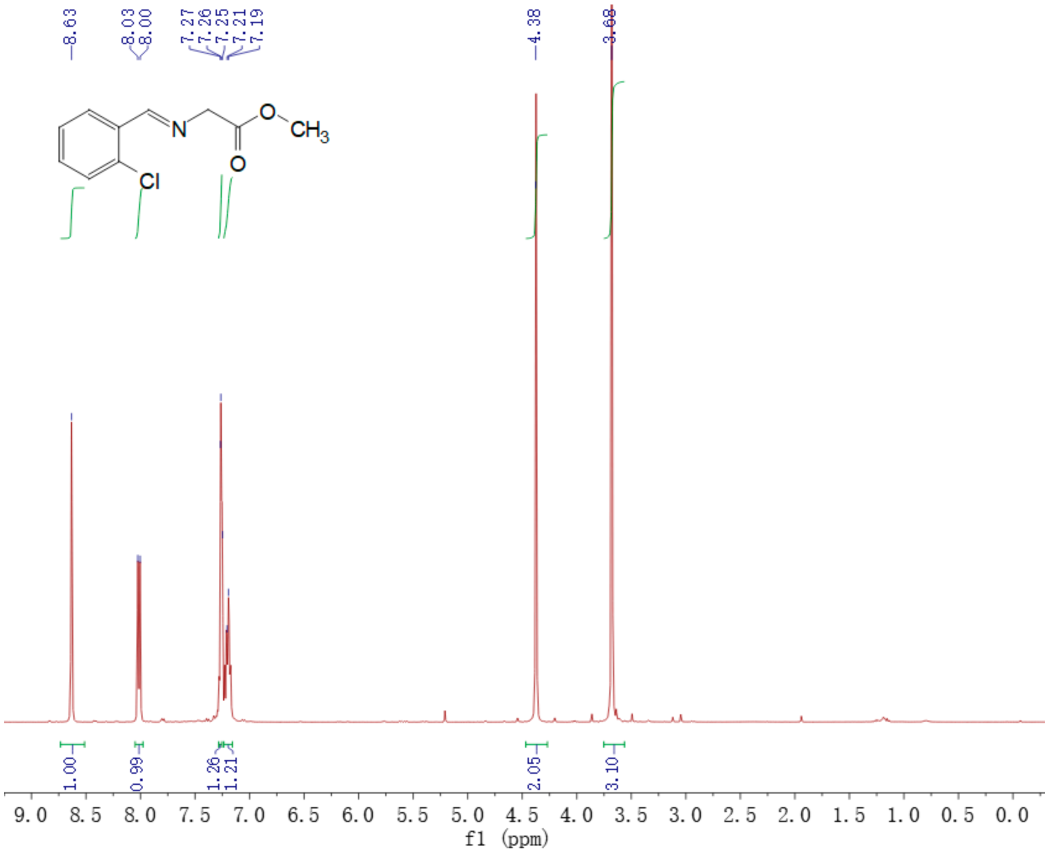

Fig. S9 The ^{1}H NMR (400 MHz, CDCl3) of **1e**

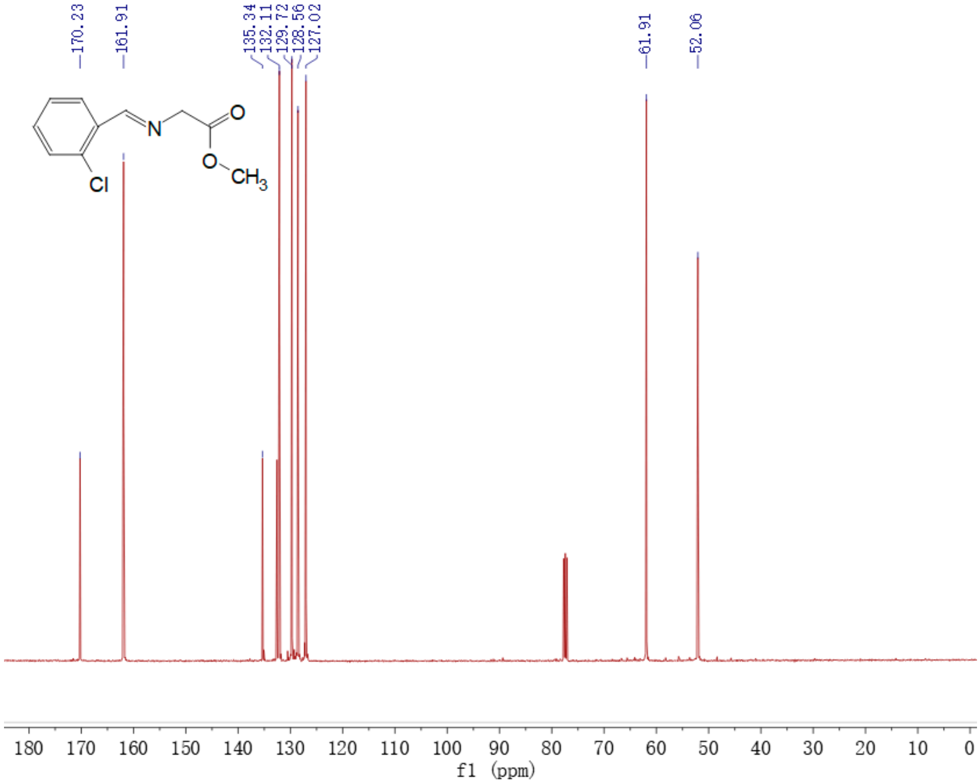

Fig. S10 The ^{13}C NMR (101 MHz, CDCl3) of **1e**

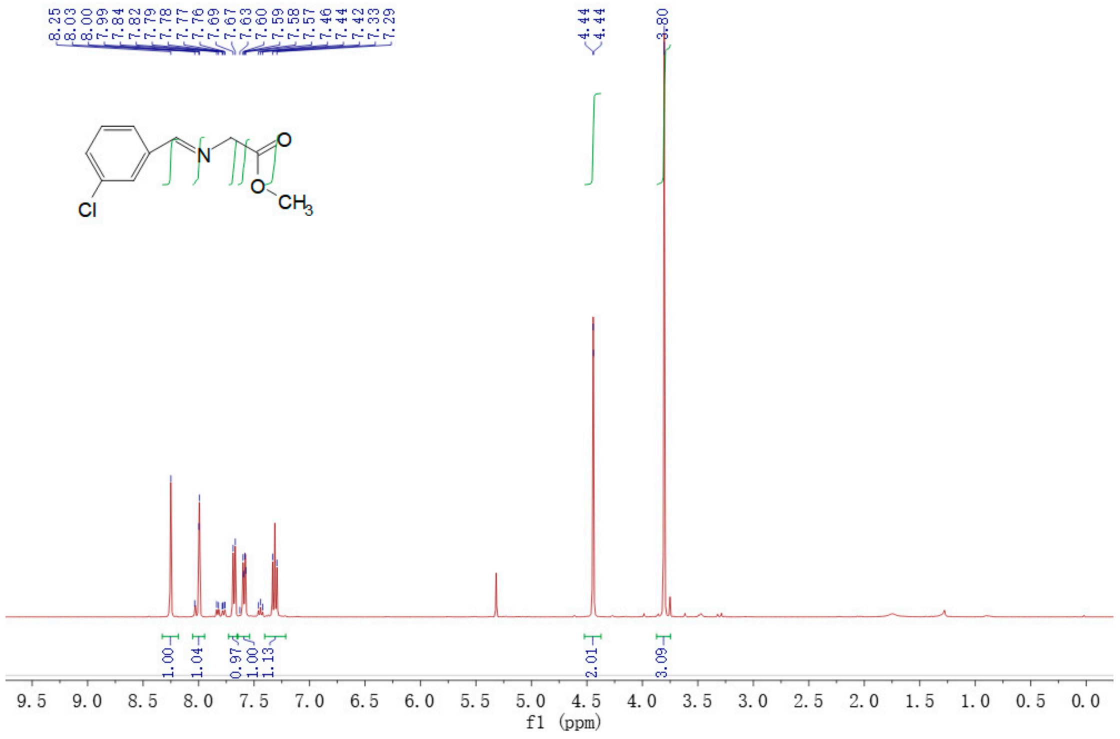

Fig. S11 The ^{1}H NMR (400 MHz, $CDCl_3$) of **1f**

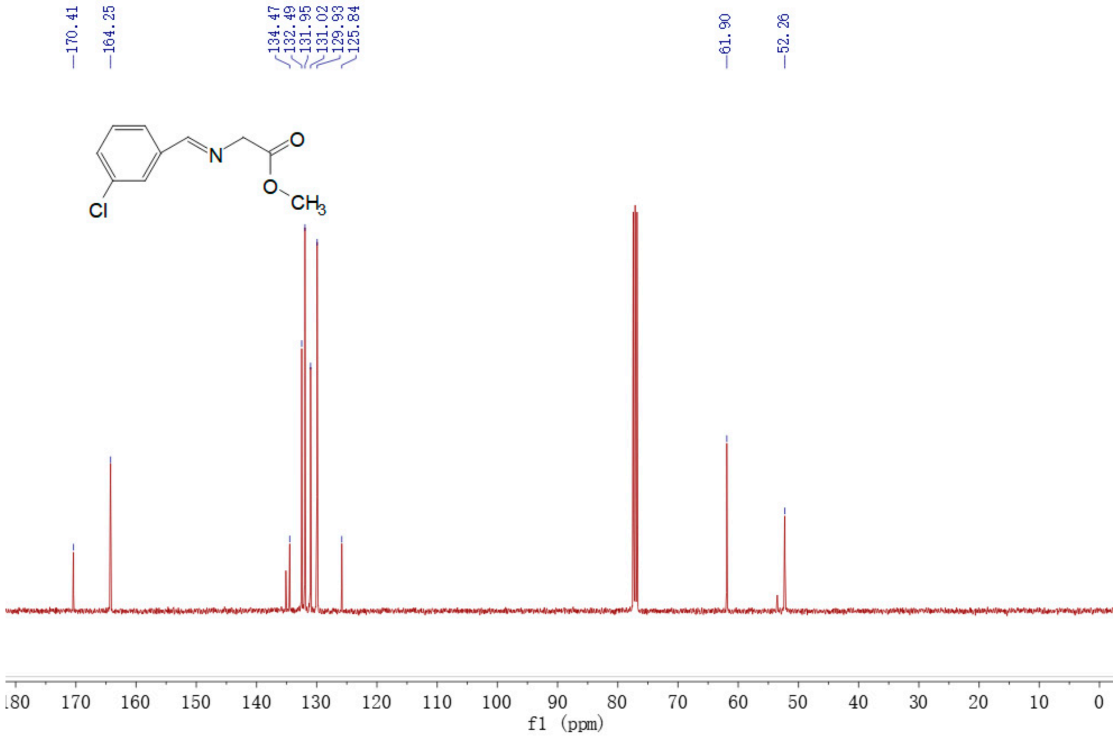

Fig. S12 The ^{13}C NMR (101 MHz, $CDCl_3$) of **1f**

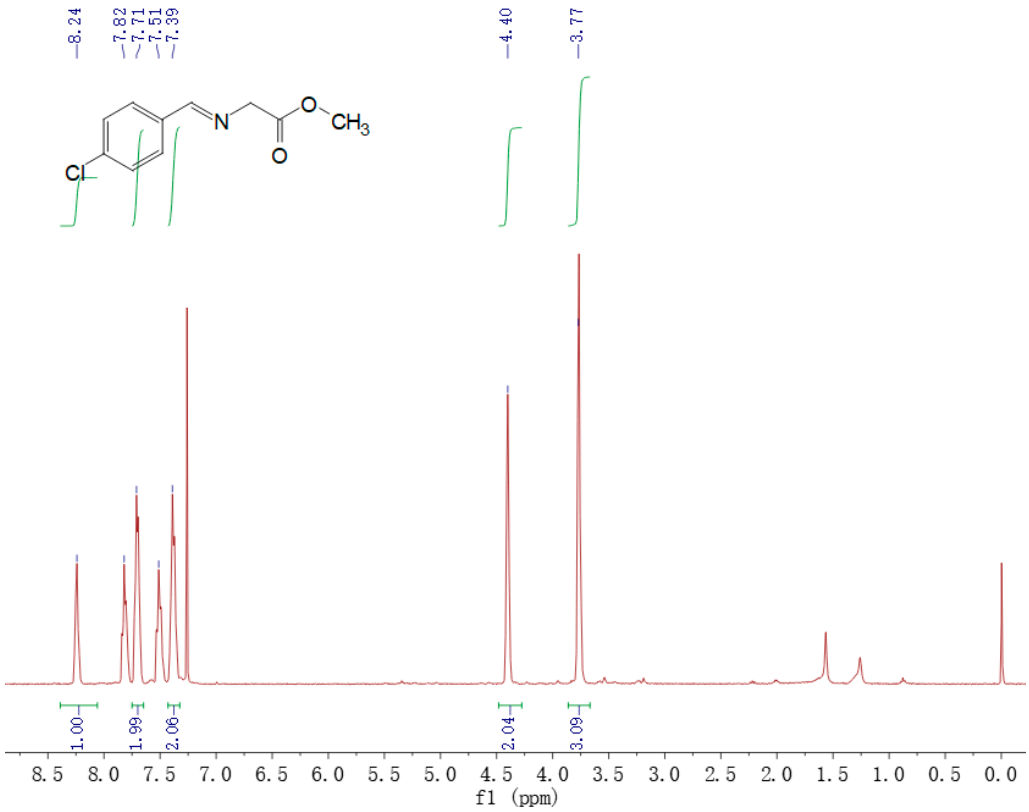

Fig. S13 The ^{1}H NMR (400 MHz, CDCl3) of **1g**

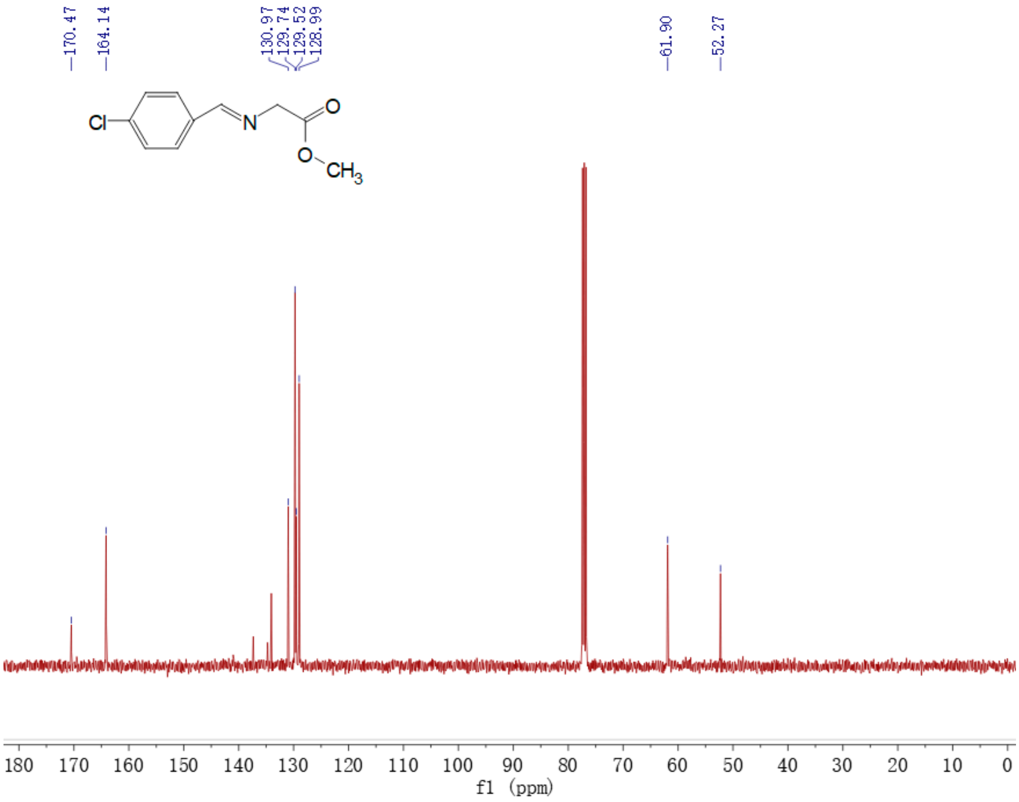

Fig. S14 The ^{13}C NMR (101 MHz, CDCl3) of **1g**

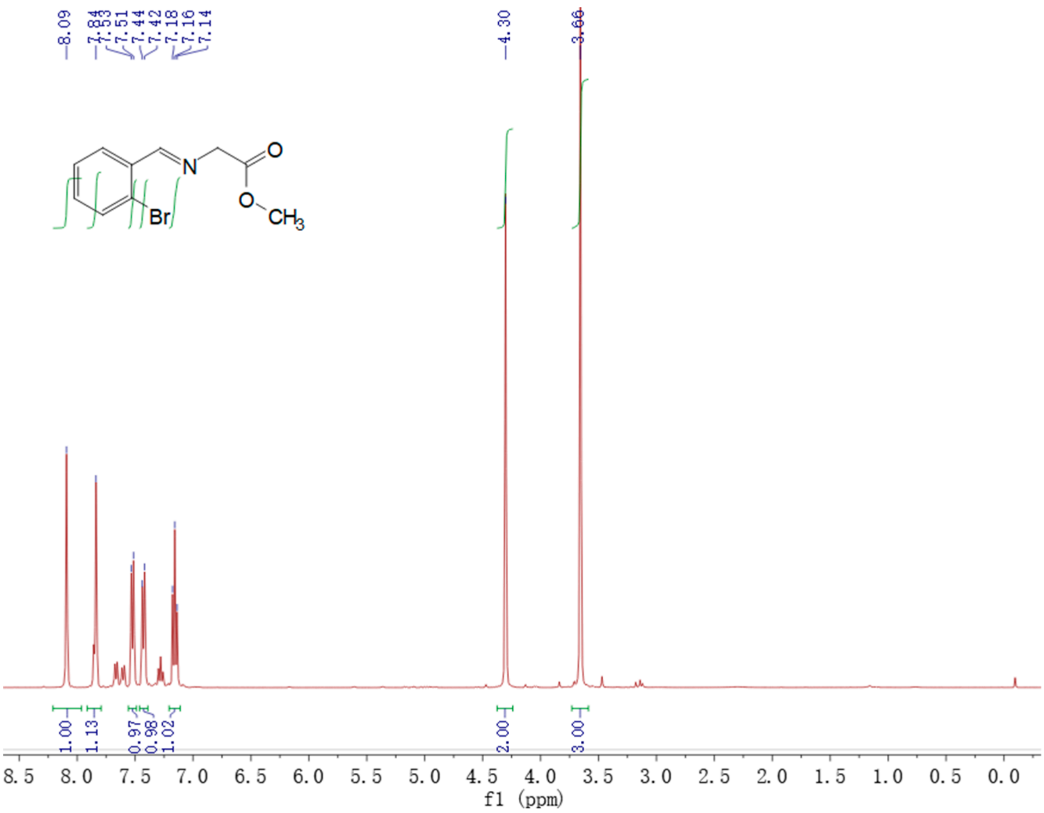

Fig. S15 The ^{1}H NMR (400 MHz, $CDCl_3$) of **1h**

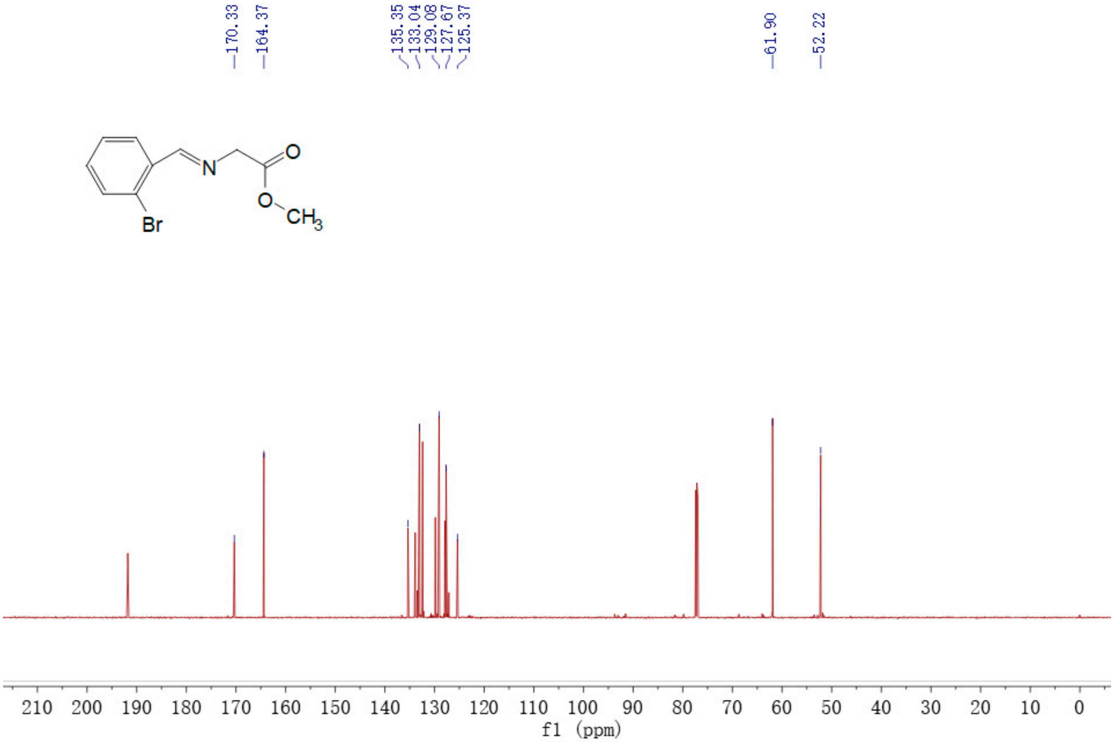

Fig. S16 The ^{13}C NMR (151 MHz, $CDCl_3$) of **1h**

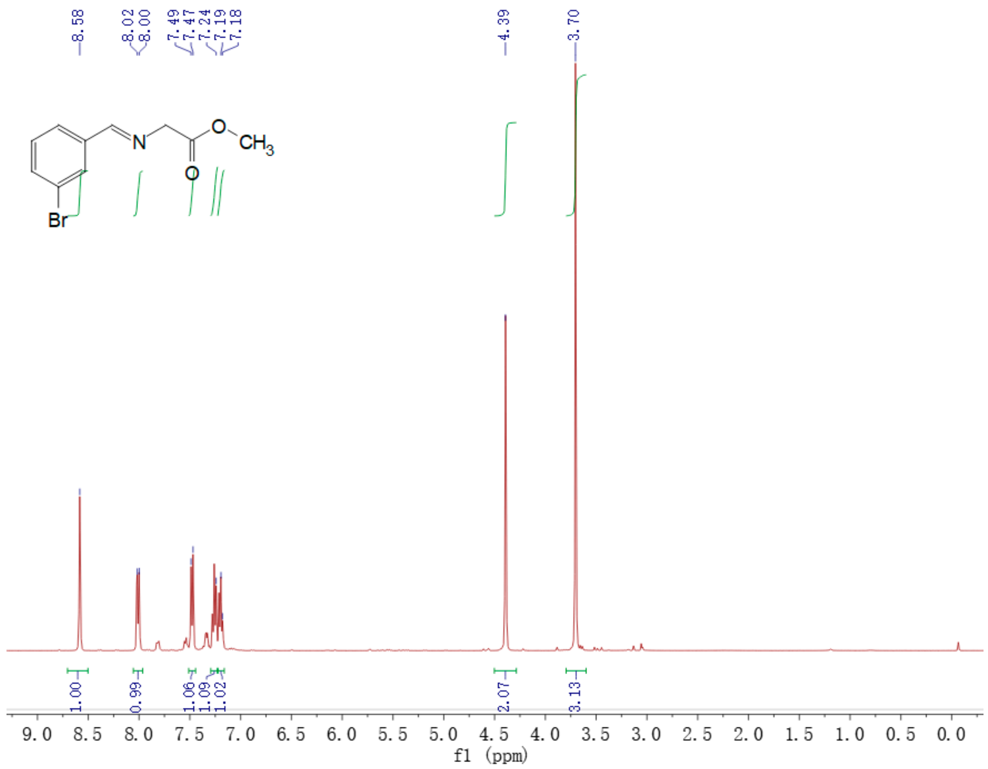

Fig. S17 The ^{1}H NMR (400 MHz, CDCl₃) of **1i**

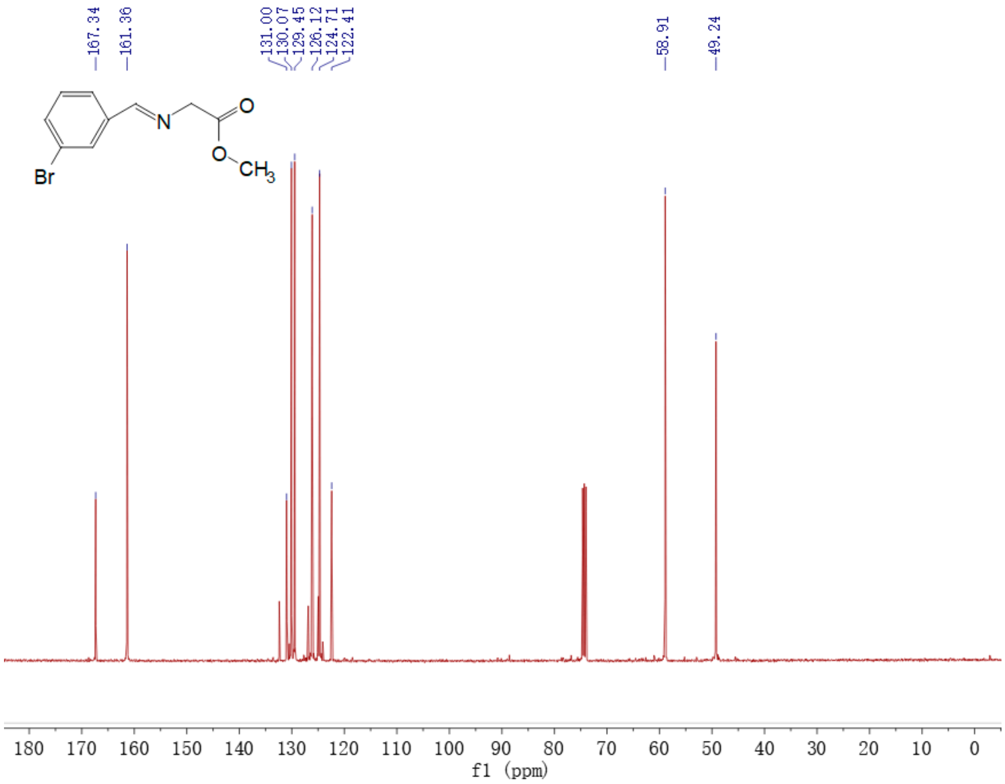

Fig. S18 The ^{13}C NMR (101 MHz, CDCl₃) of **1i**

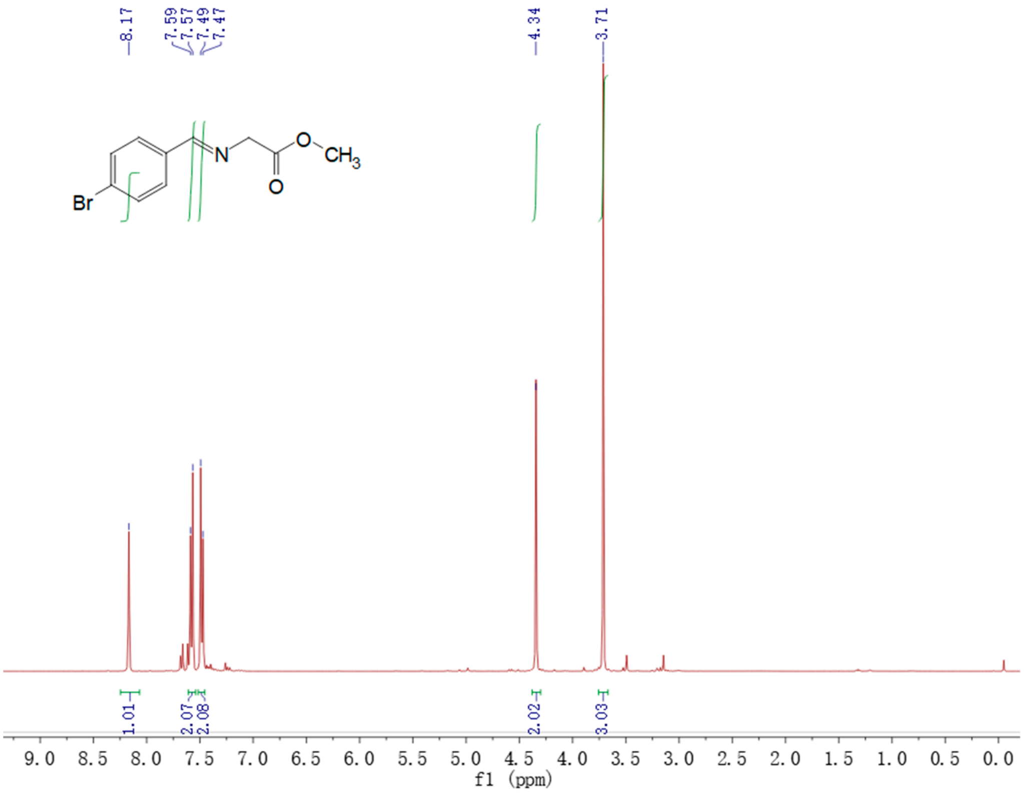

Fig. S19 The ^{1}H NMR (400 MHz, $CDCl_3$) of **1j**

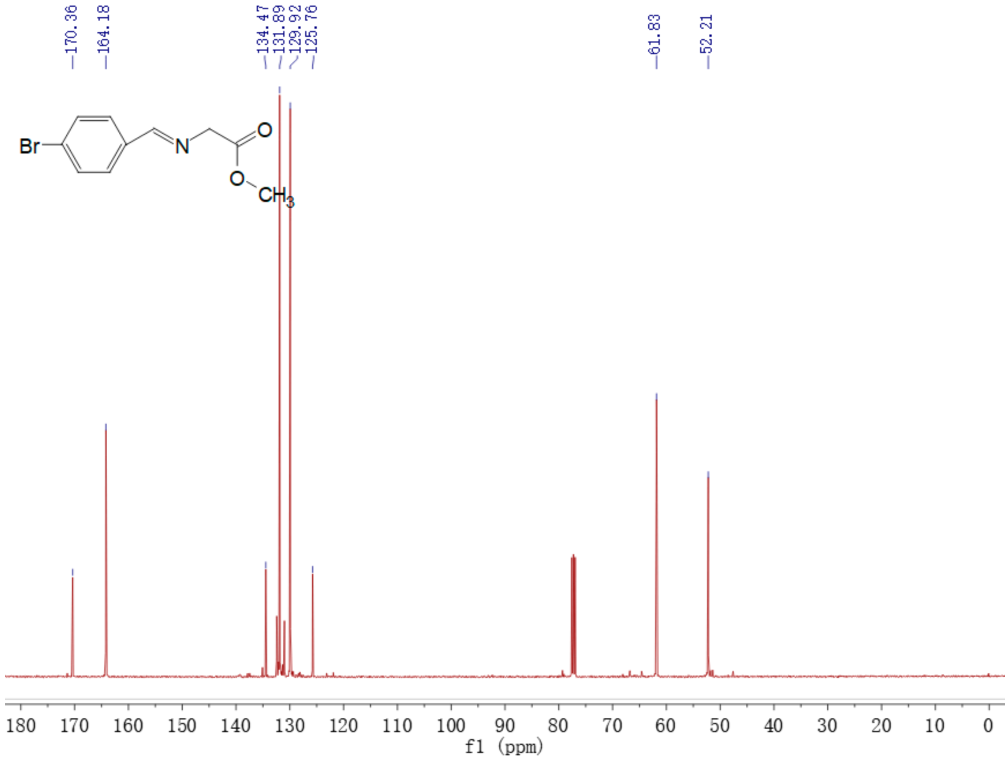

Fig. S20 The ^{13}C NMR (101 MHz, $CDCl_3$) of **1j**

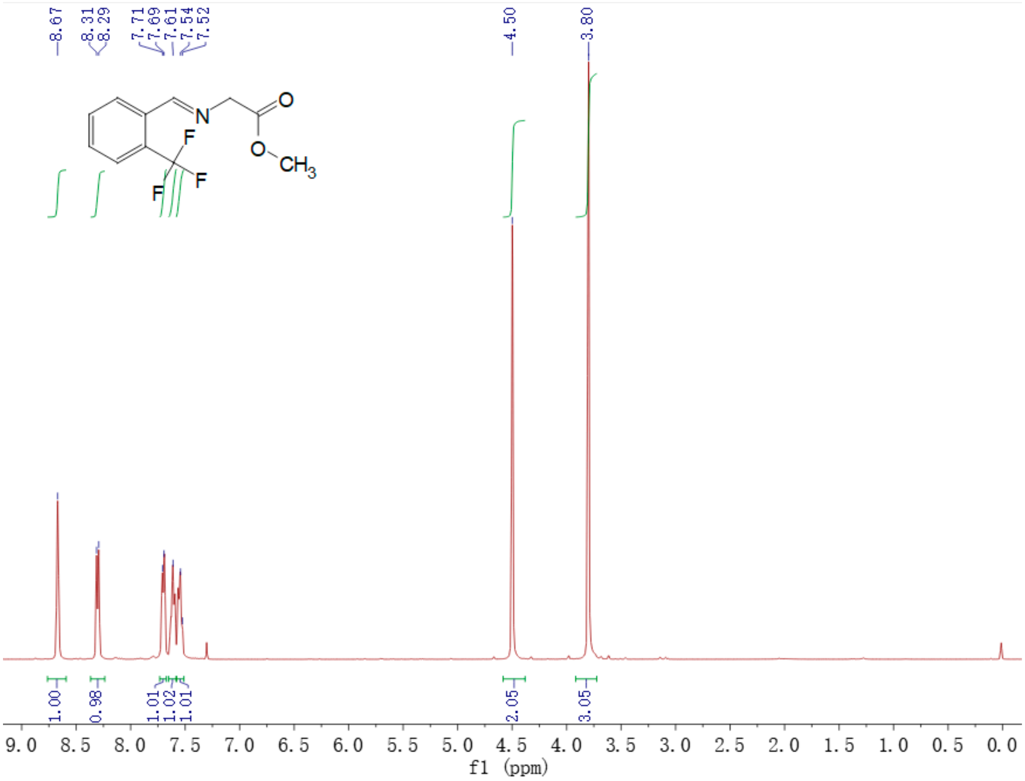

Fig. S21 The ^{1}H NMR (400 MHz, $CDCl_3$) of **1k**

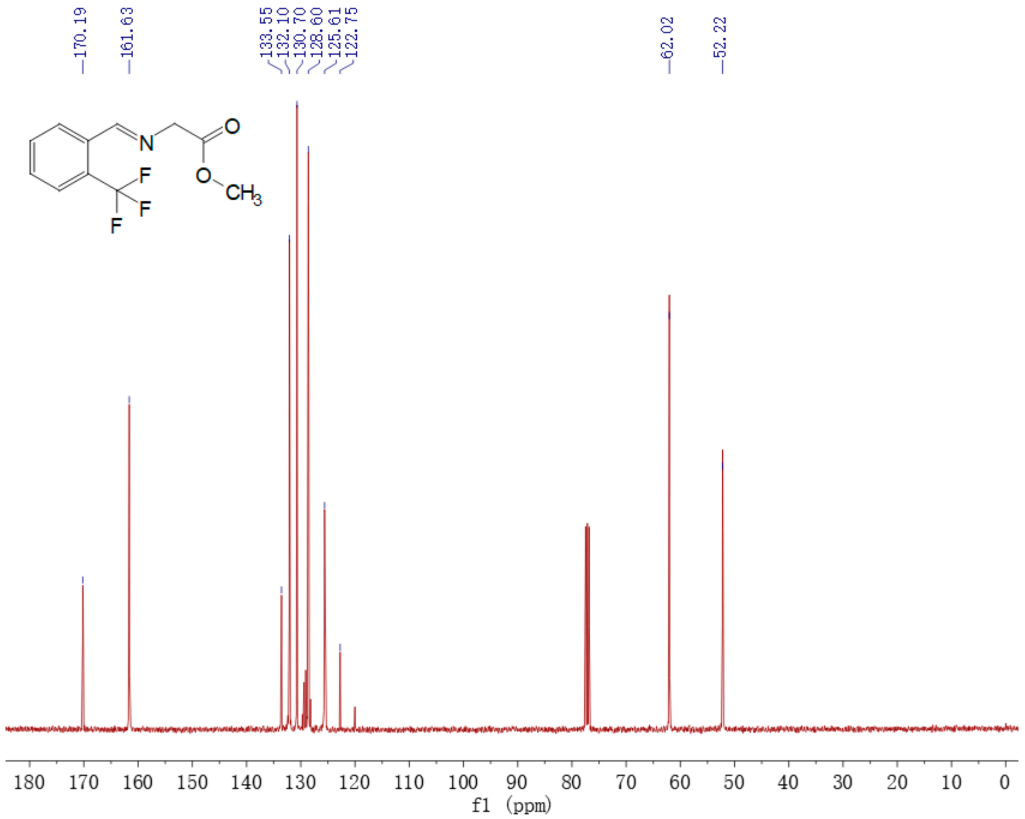

Fig. S22 The ^{13}C NMR (101 MHz, $CDCl_3$) of **1k**

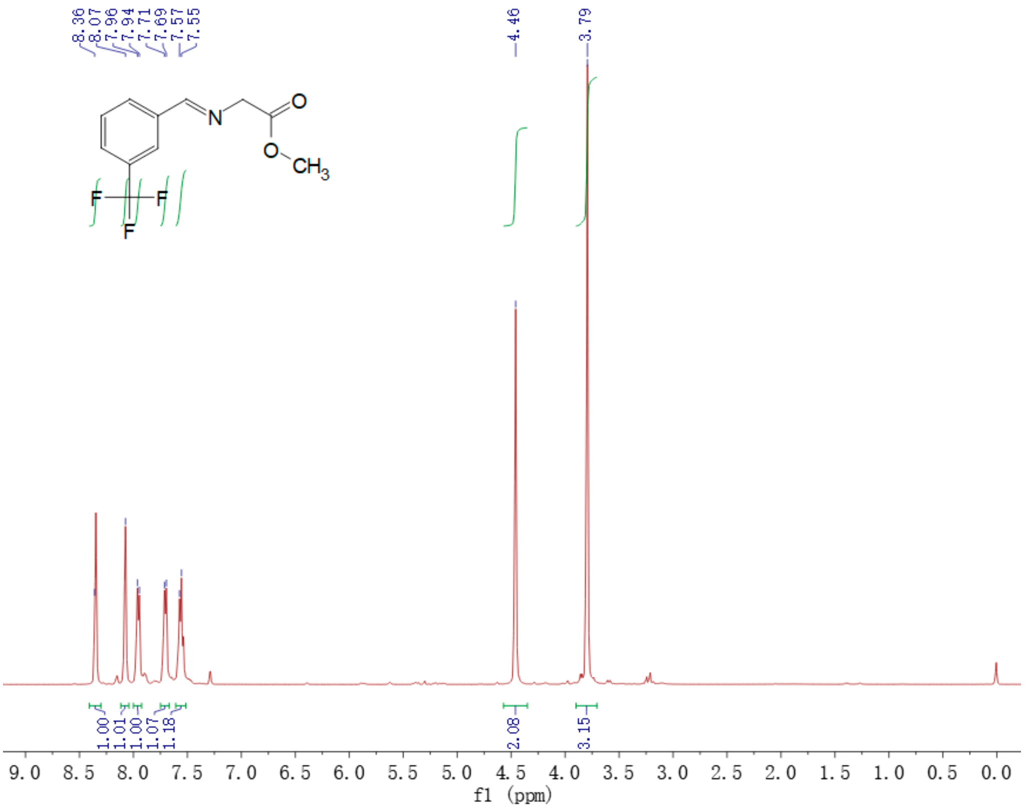

Fig. S23 The ^{1}H NMR (400 MHz, $CDCl_3$) of **1l**

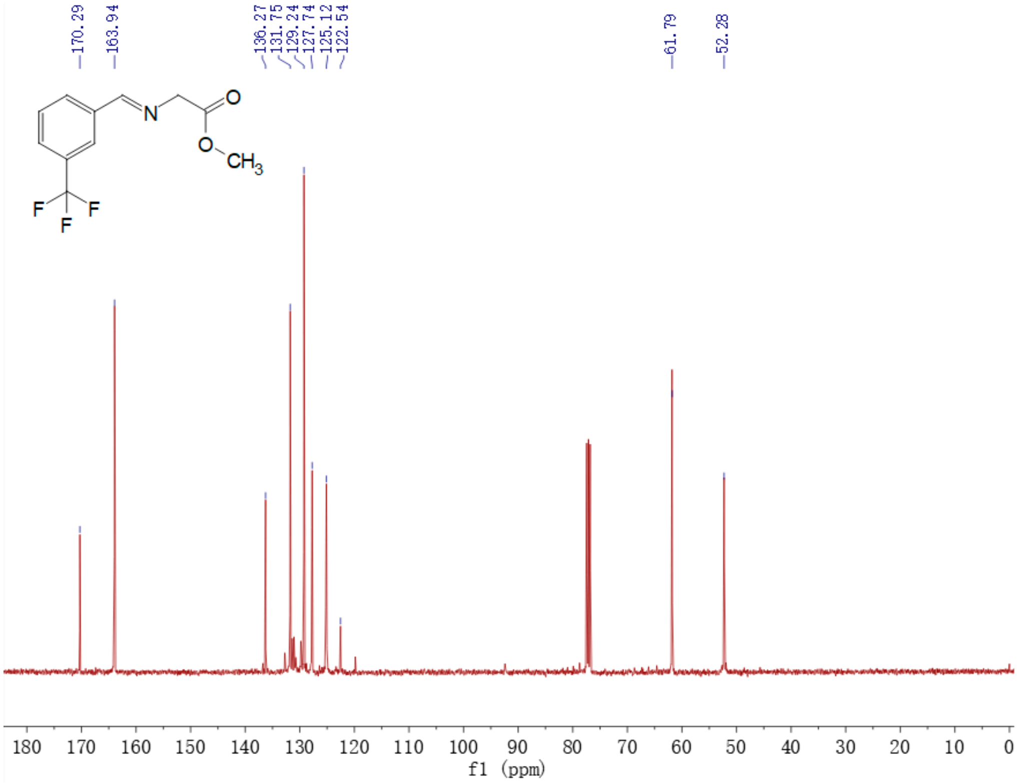

Fig. S24 The ^{13}C NMR (101 MHz, $CDCl_3$) of **1l**

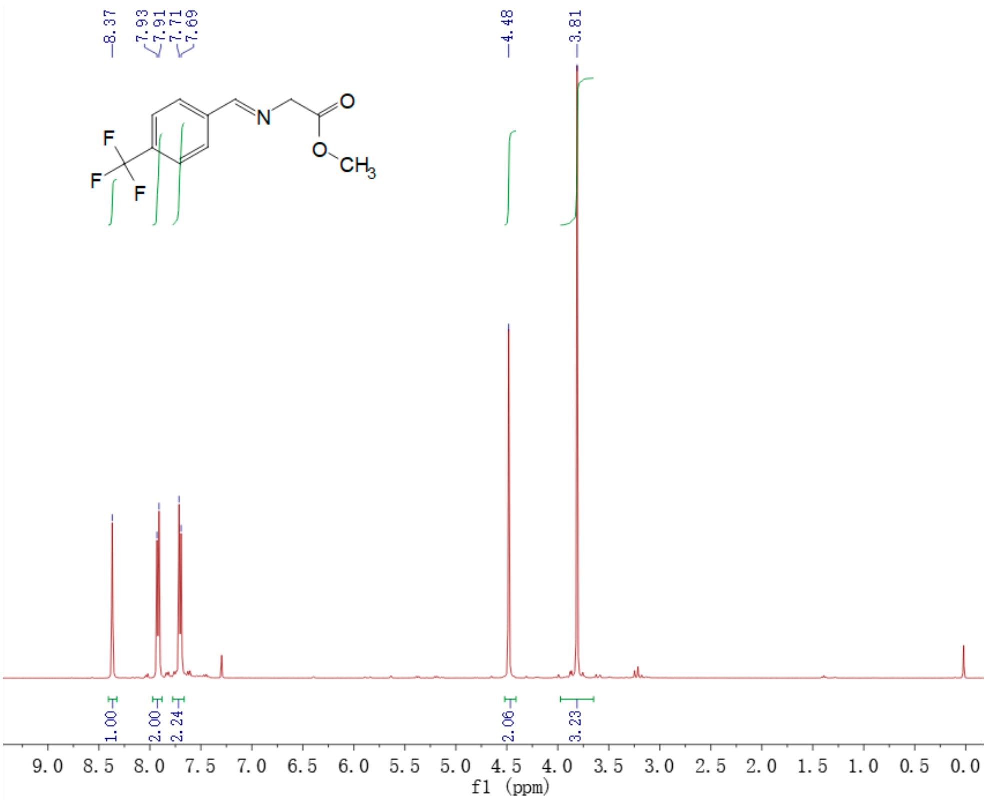

Fig. S25 The ^{1}H NMR (400 MHz, CDCl3) of **1m**

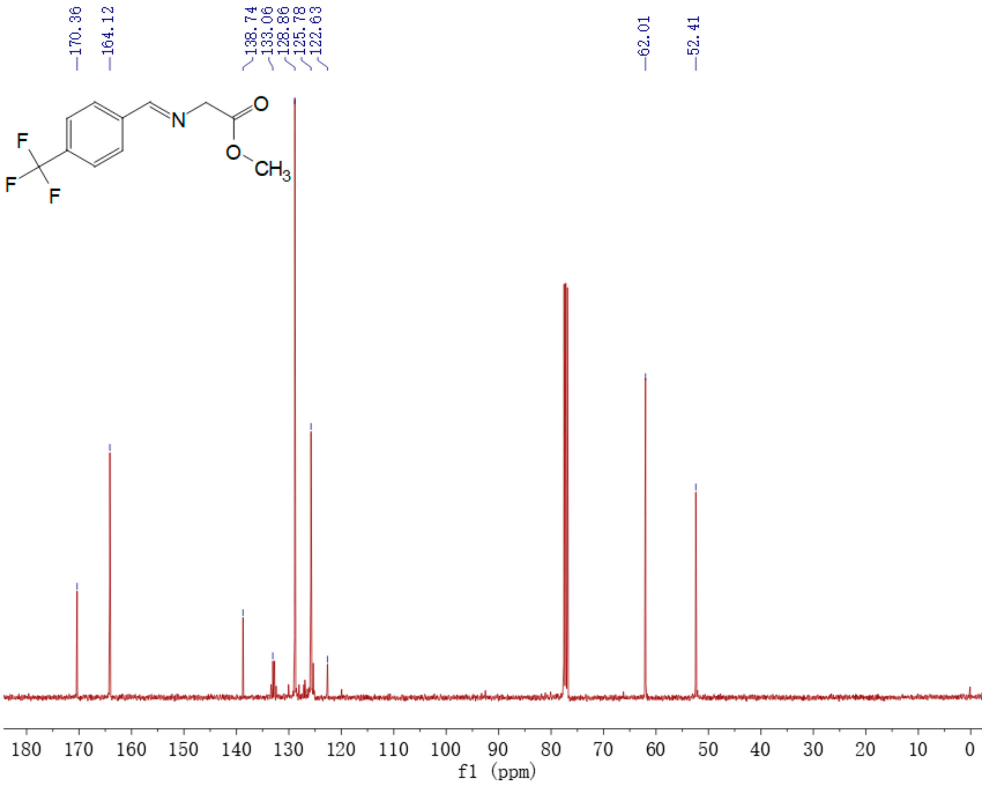

Fig. S26 The ^{13}C NMR (101 MHz, CDCl3) of **1m**

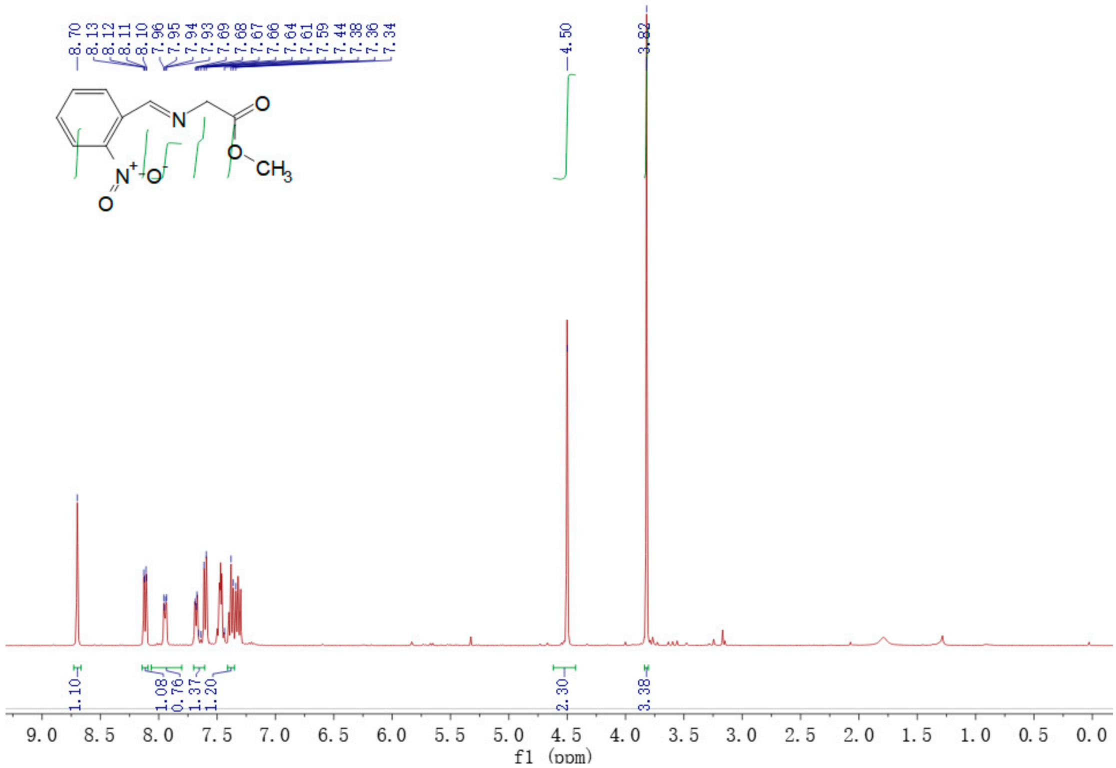

Fig. S27 The ^{1}H NMR (101 MHz, CDCl3) of **1n**

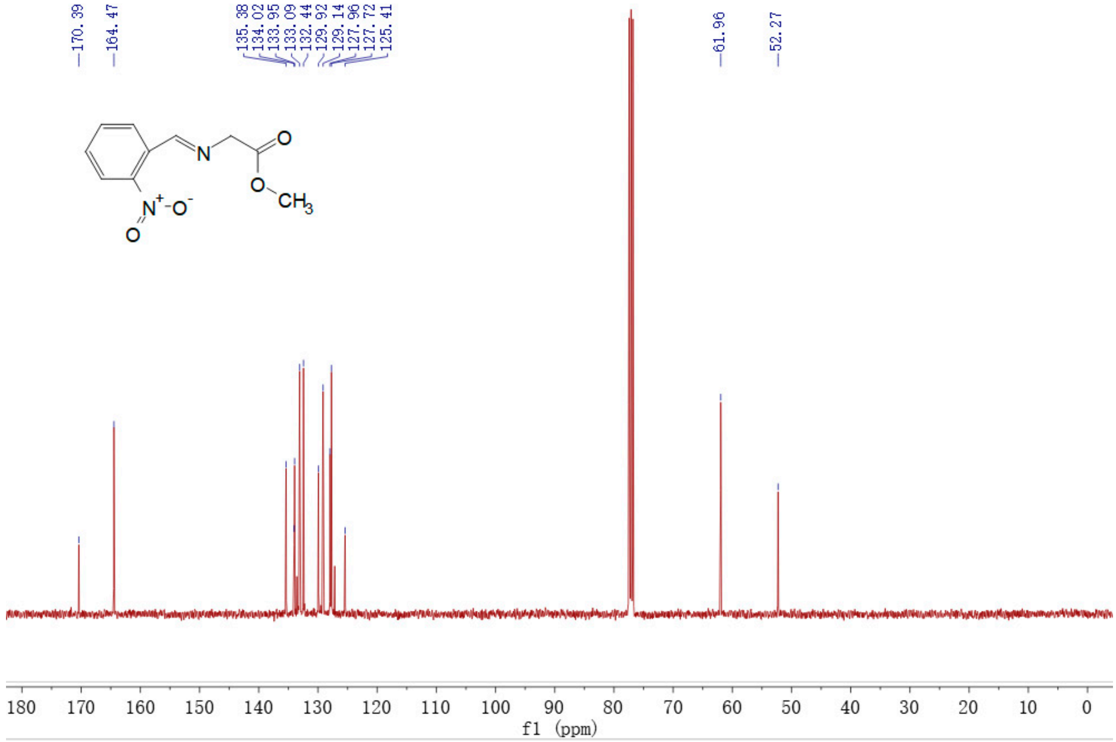

Fig. S28 The ^{13}C NMR (101 MHz, CDCl3) of **1n**

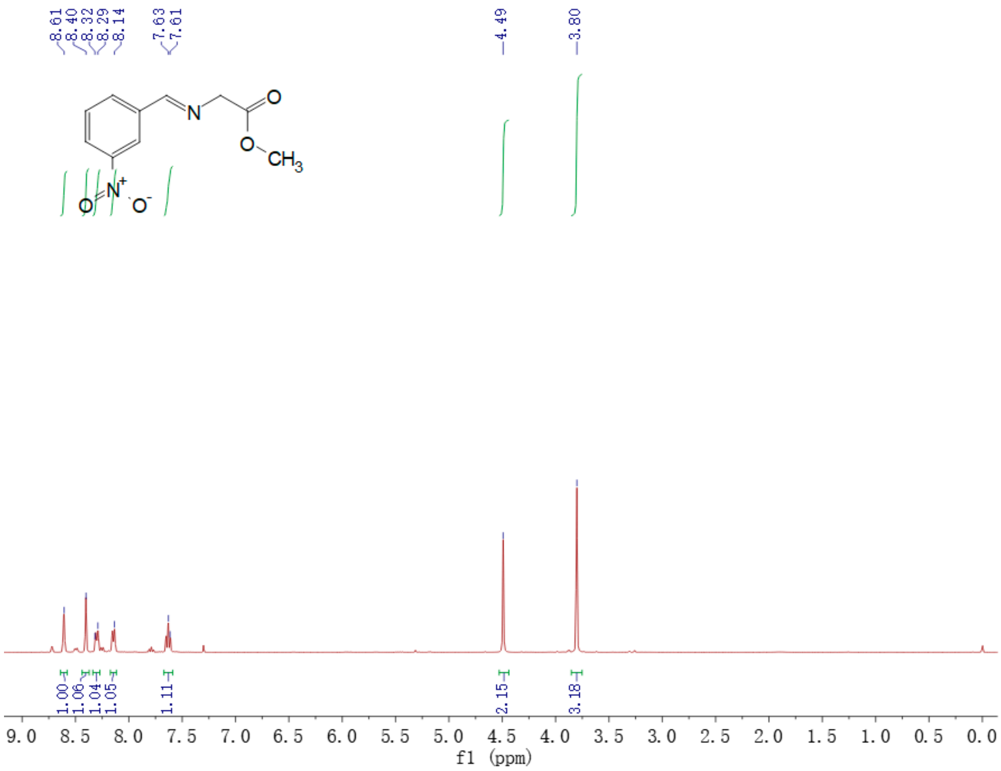

Fig. S29 The ^{1}H NMR (400 MHz, CDCl3) of **1o**

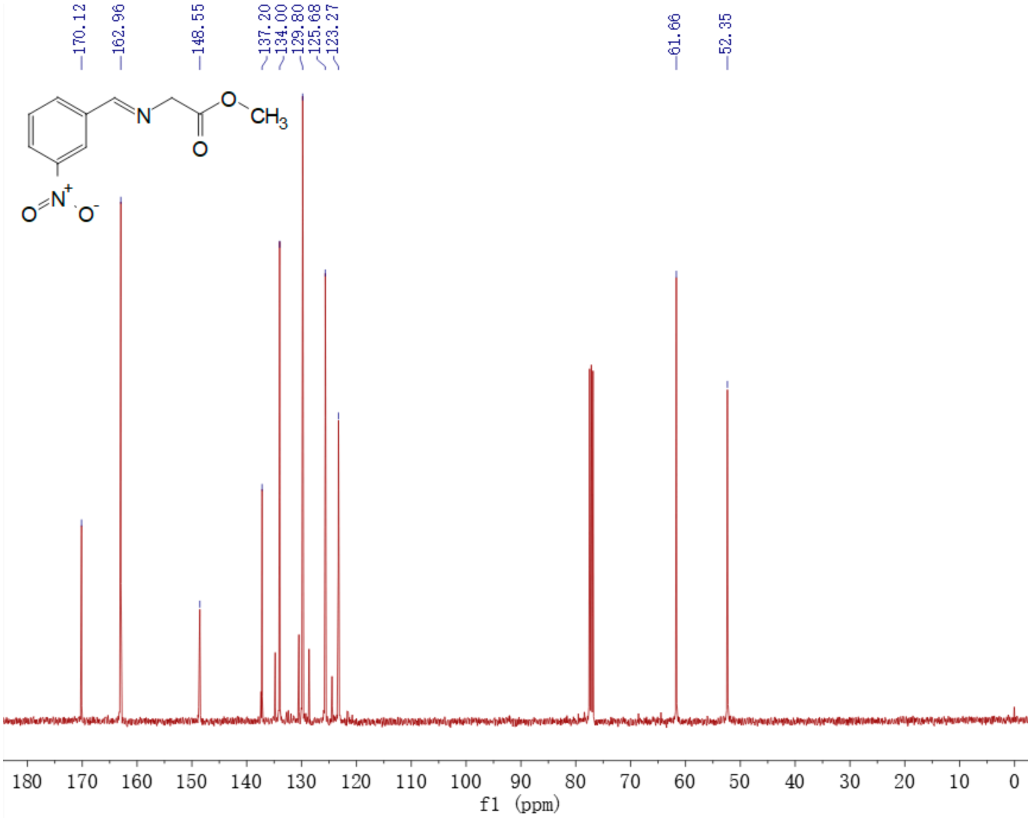

Fig. S30 The ^{13}C NMR (101 MHz, CDCl3) of **1o**

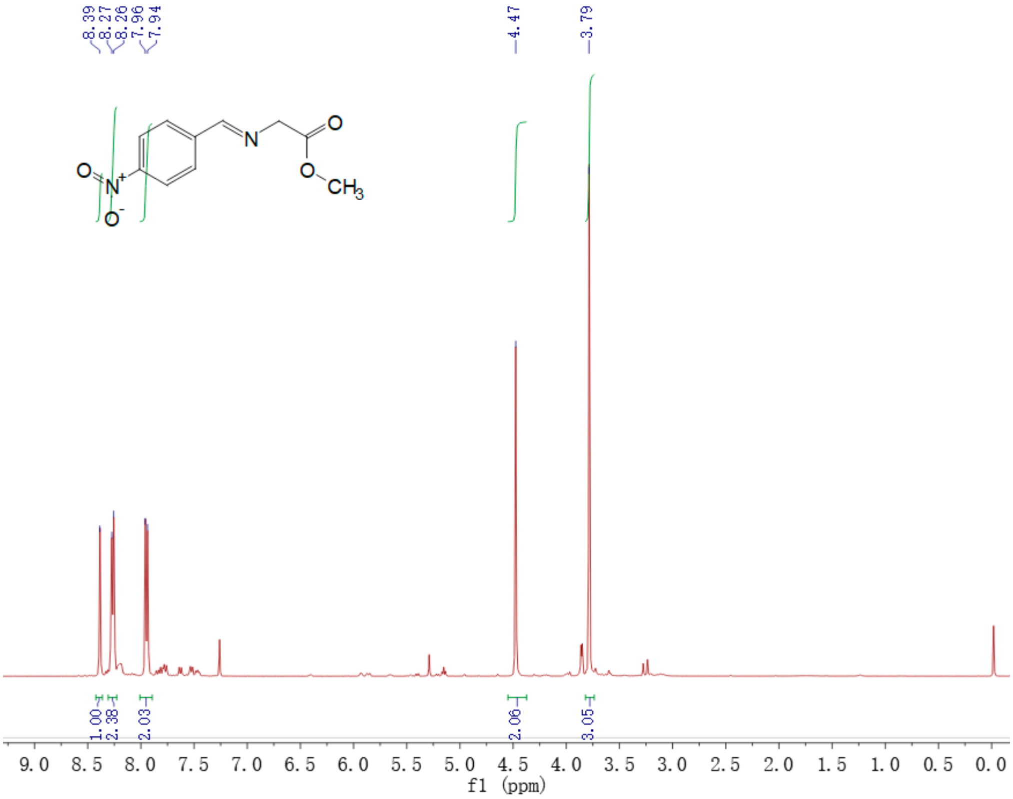

Fig. S31 The ^{1}H NMR (400 MHz, CDCl3) of **1p**

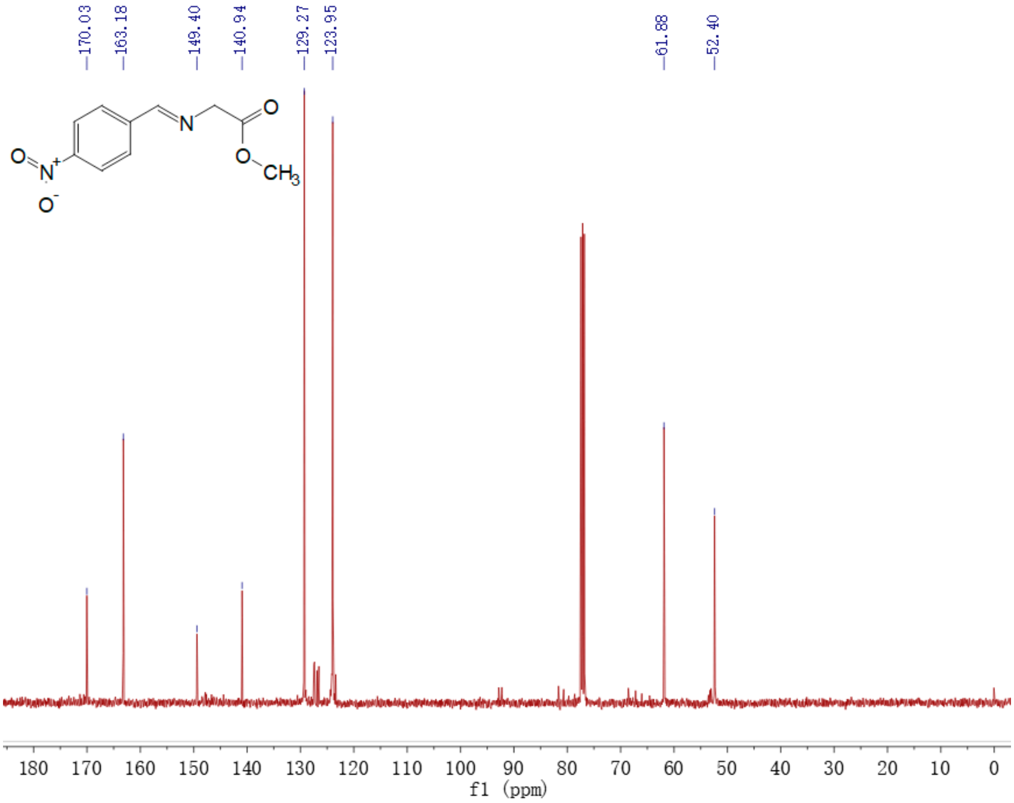

Fig. S32 The ^{13}C NMR (101 MHz, CDCl3) of **1p**

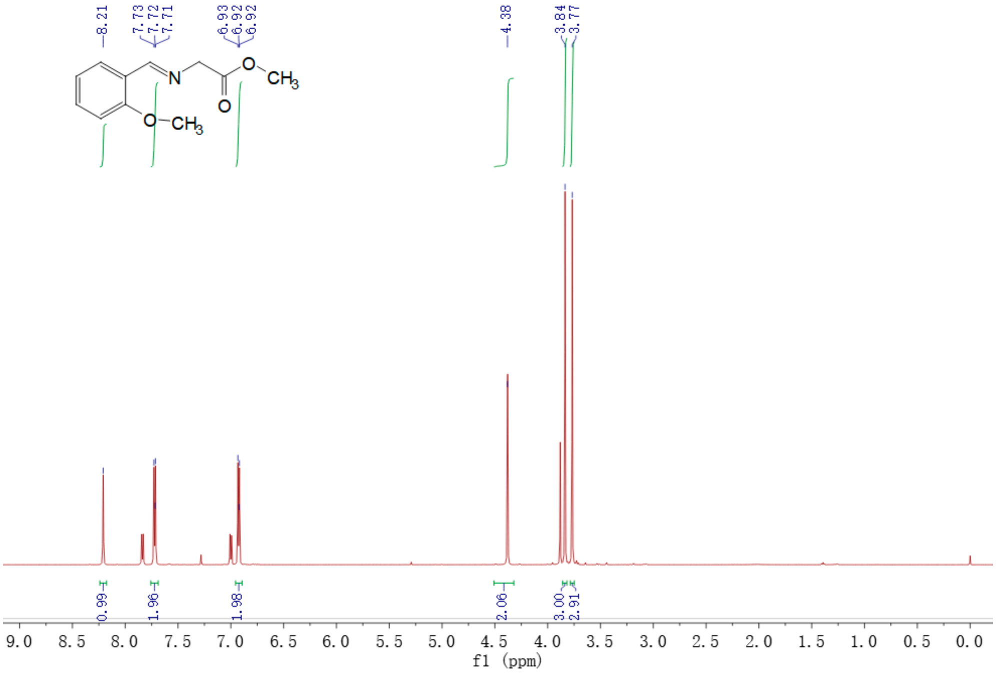

Fig. S33 The ^{1}H NMR (400 MHz, CDCl3) of **1q**

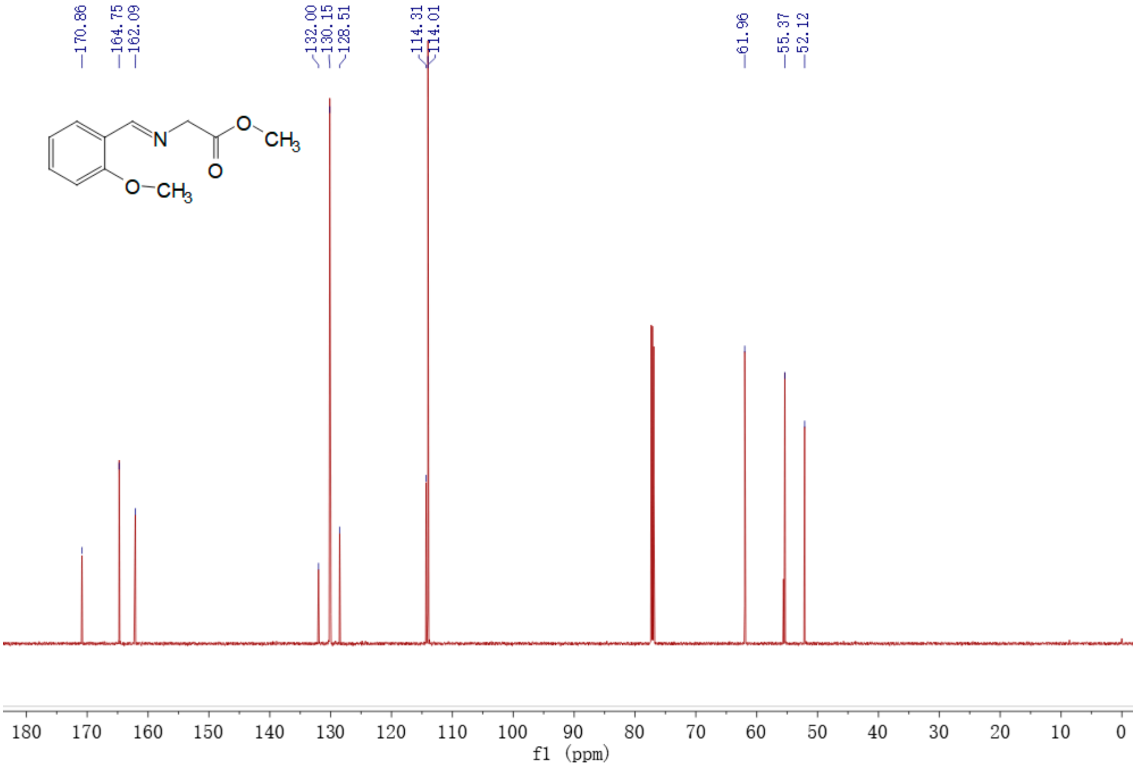

Fig. S34 The ^{13}C NMR (151 MHz, CDCl3) of **1q**

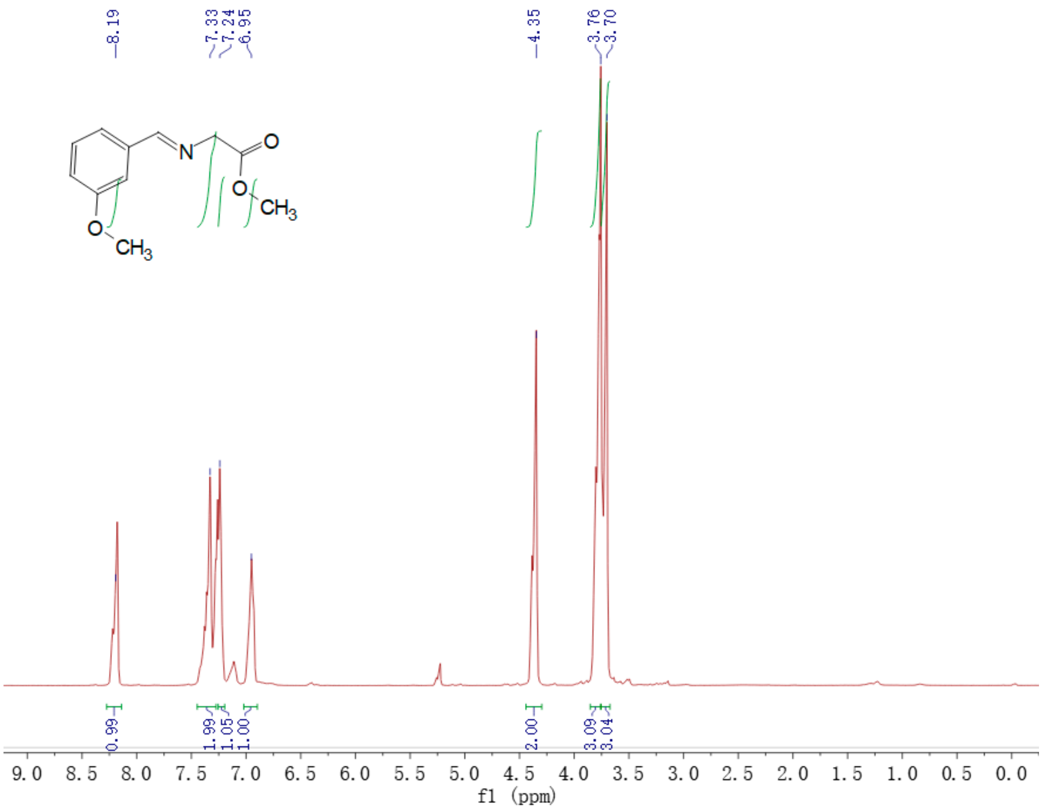

Fig. S35 The ^{1}H NMR (400 MHz, $CDCl_3$) of **1r**

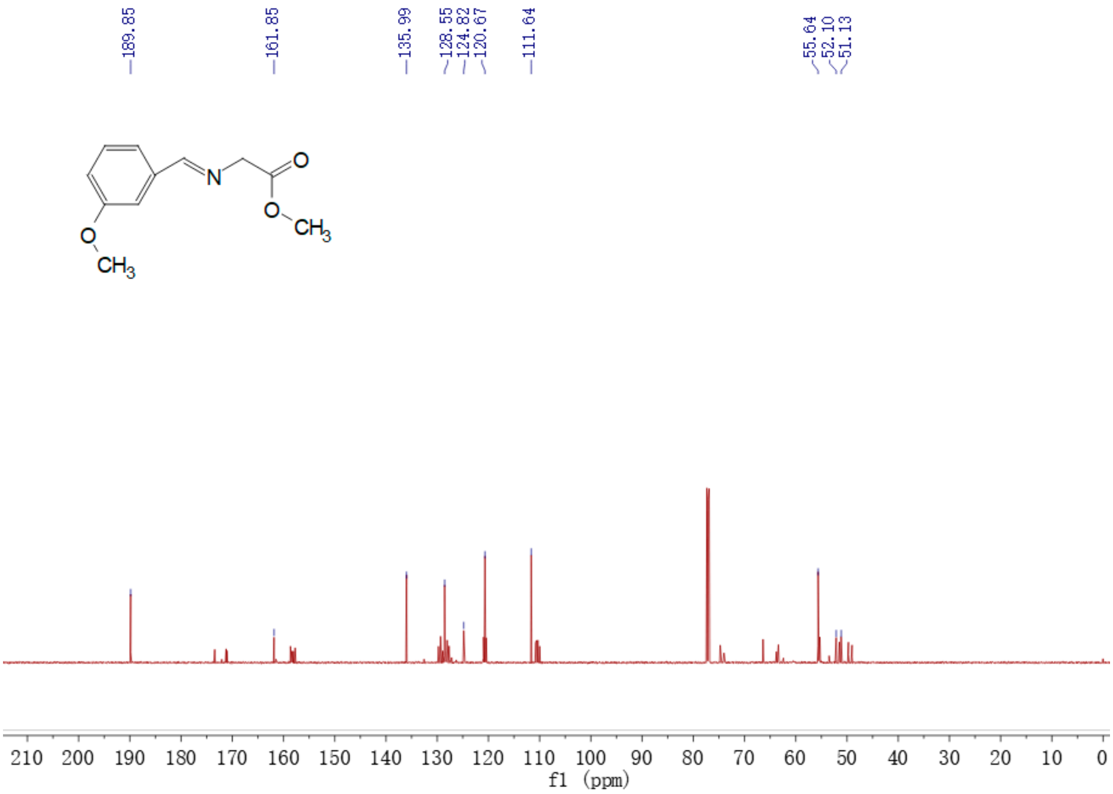

Fig. S36 The ^{13}C NMR (151 MHz, $CDCl_3$) of **1r**

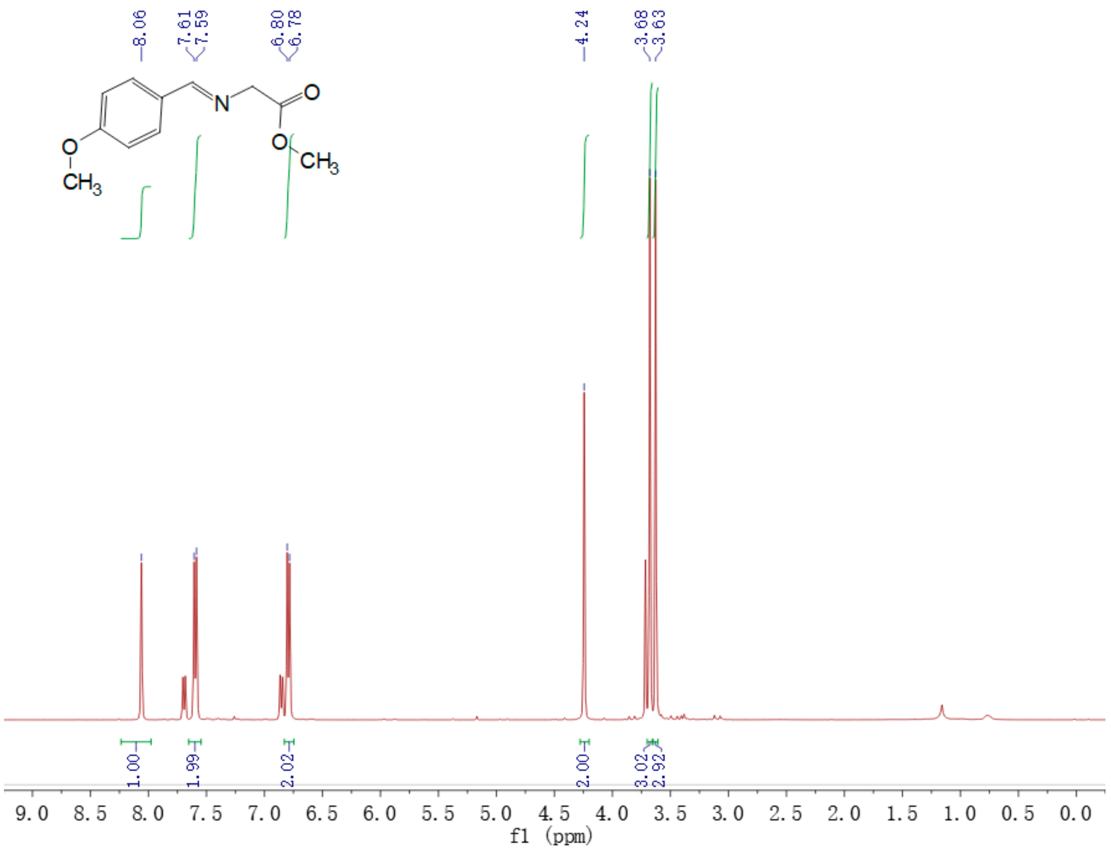

Fig. S37 The ^{1}H NMR (400 MHz, $CDCl_3$) of **1s**

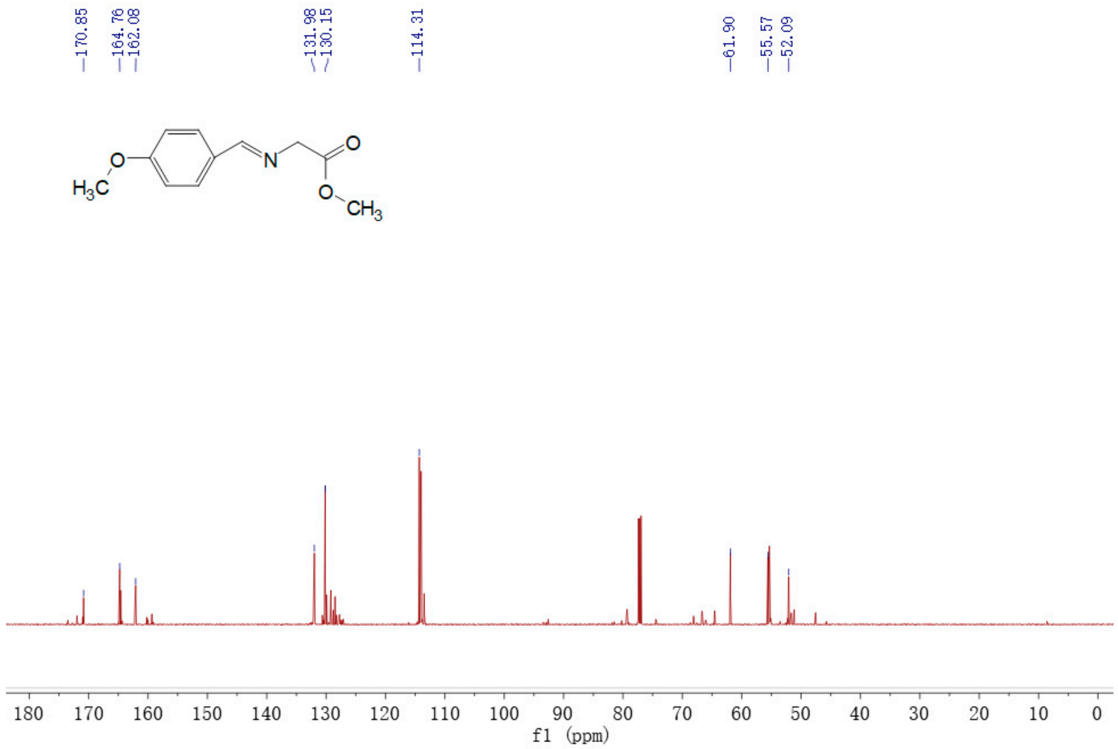

Fig. S38 The ^{13}C NMR (151 MHz, $CDCl_3$) of **1s**

5. Spectrums of 2-oxoindolin-3-ylidene bodies 2a-2g

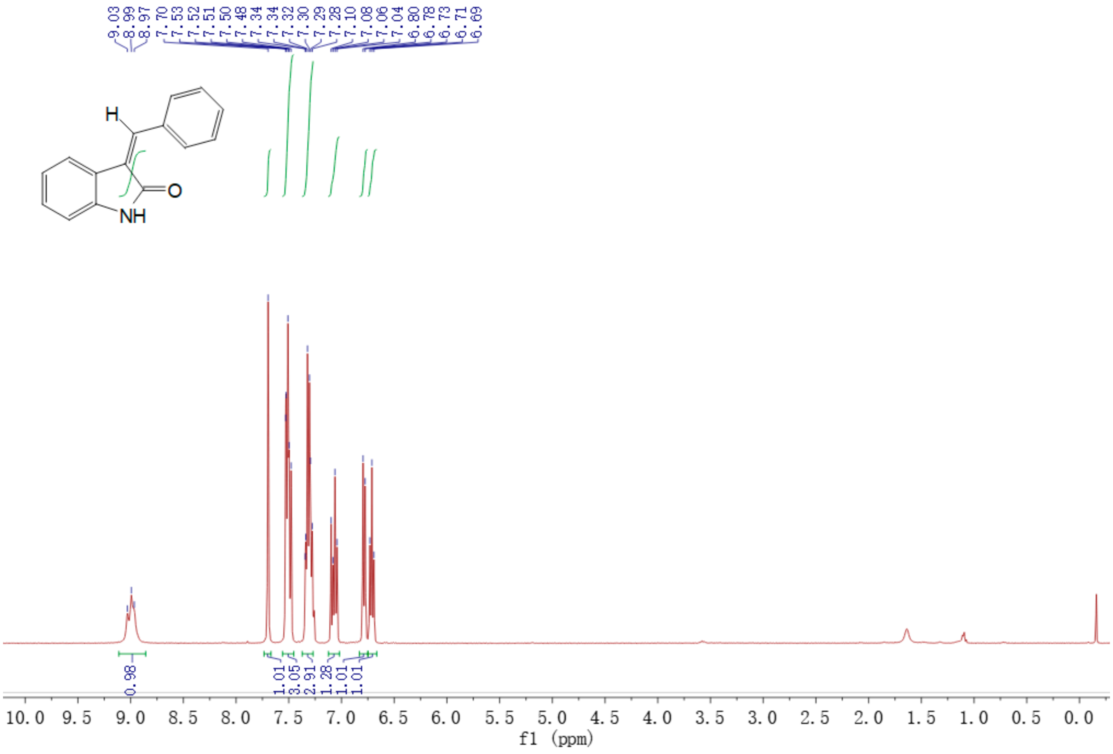

Fig. S39 The ^{1}H NMR (400 MHz, CDCl3) of **2a**

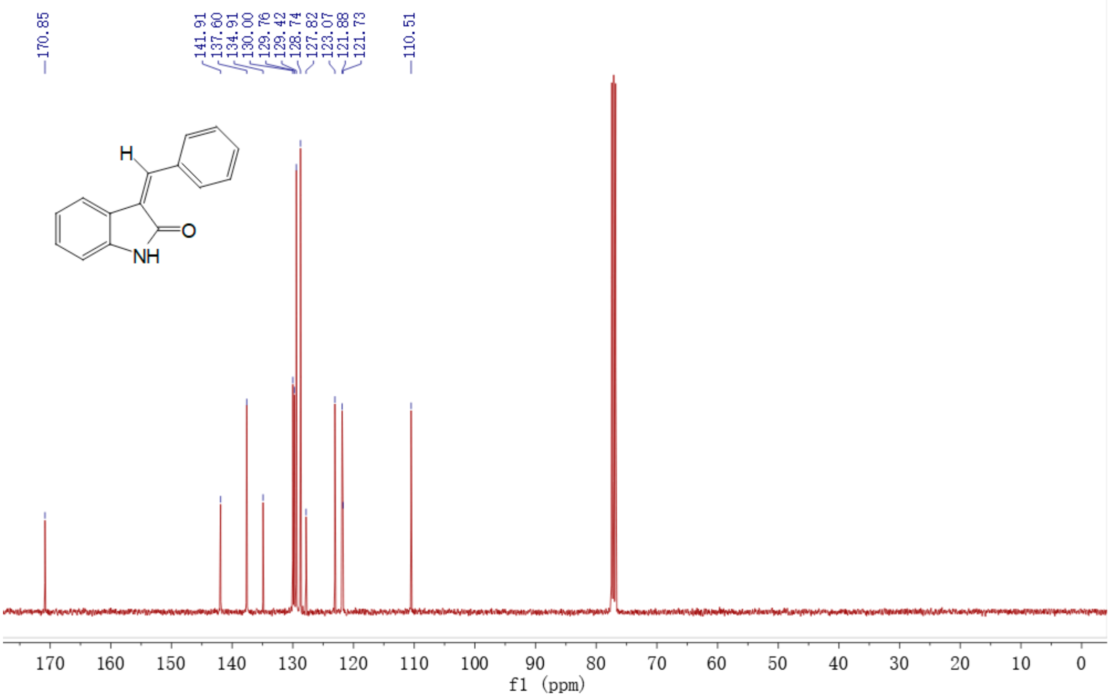

Fig. S40 The ^{13}C NMR (101 MHz, CDCl3) of **2a**

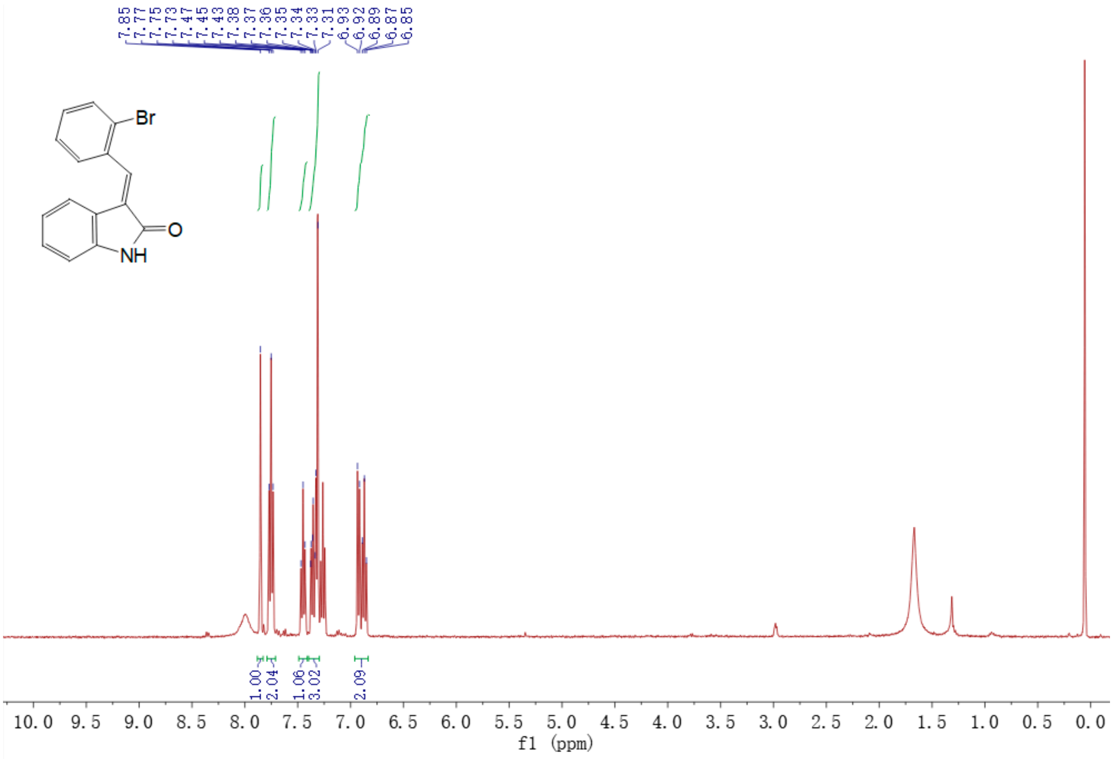

Fig. S41 The ^{1}H NMR (400 MHz, $CDCl_3$) of **2b**

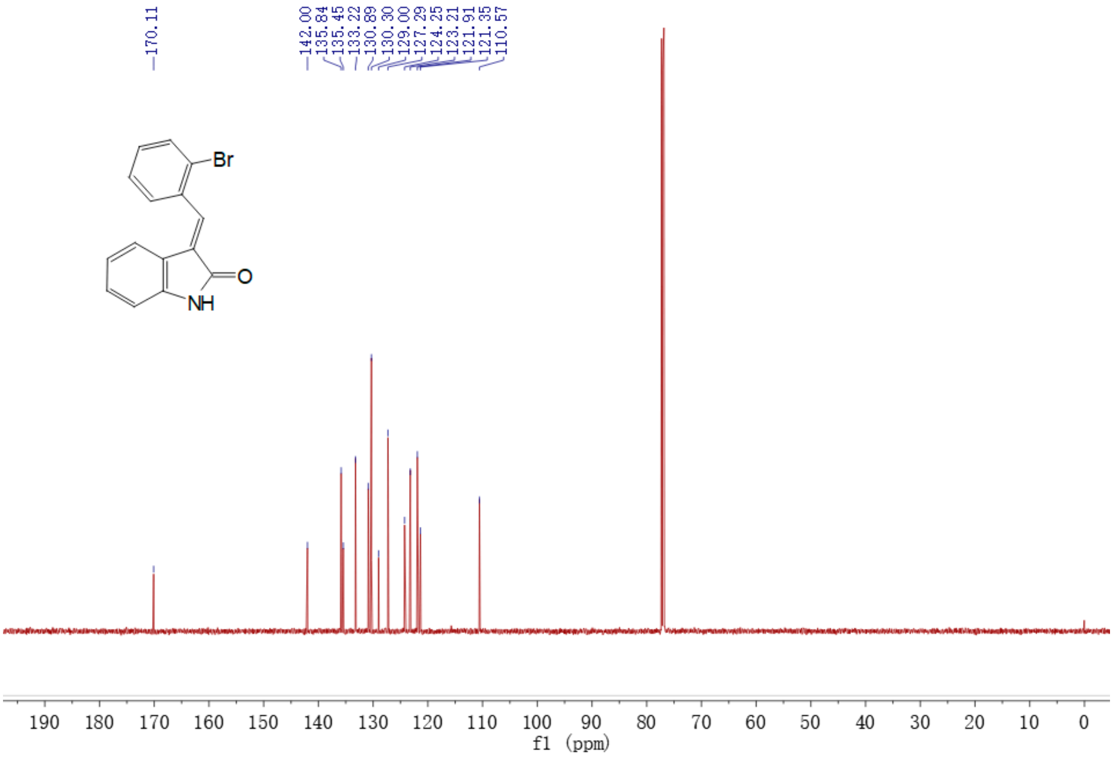

Fig. S42 The ^{13}C NMR (151 MHz, $CDCl_3$) of **2b**

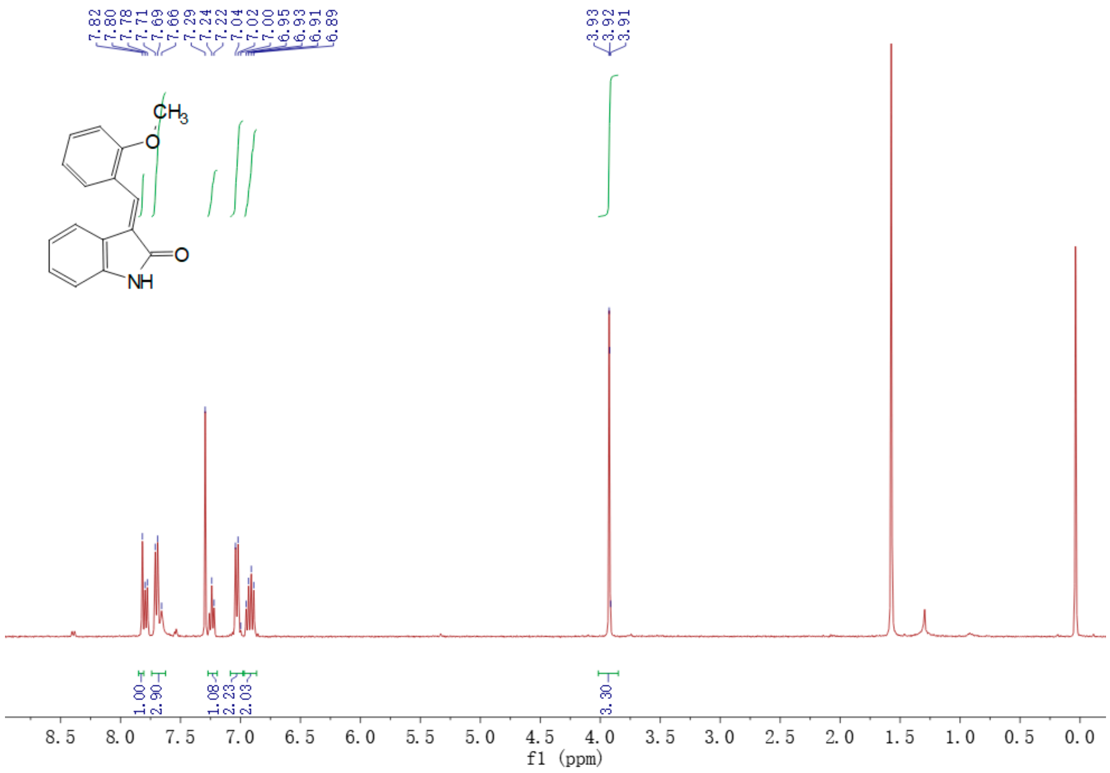

Fig. S43 The ^{1}H NMR (400 MHz, CDCl$_3$) of **2c**

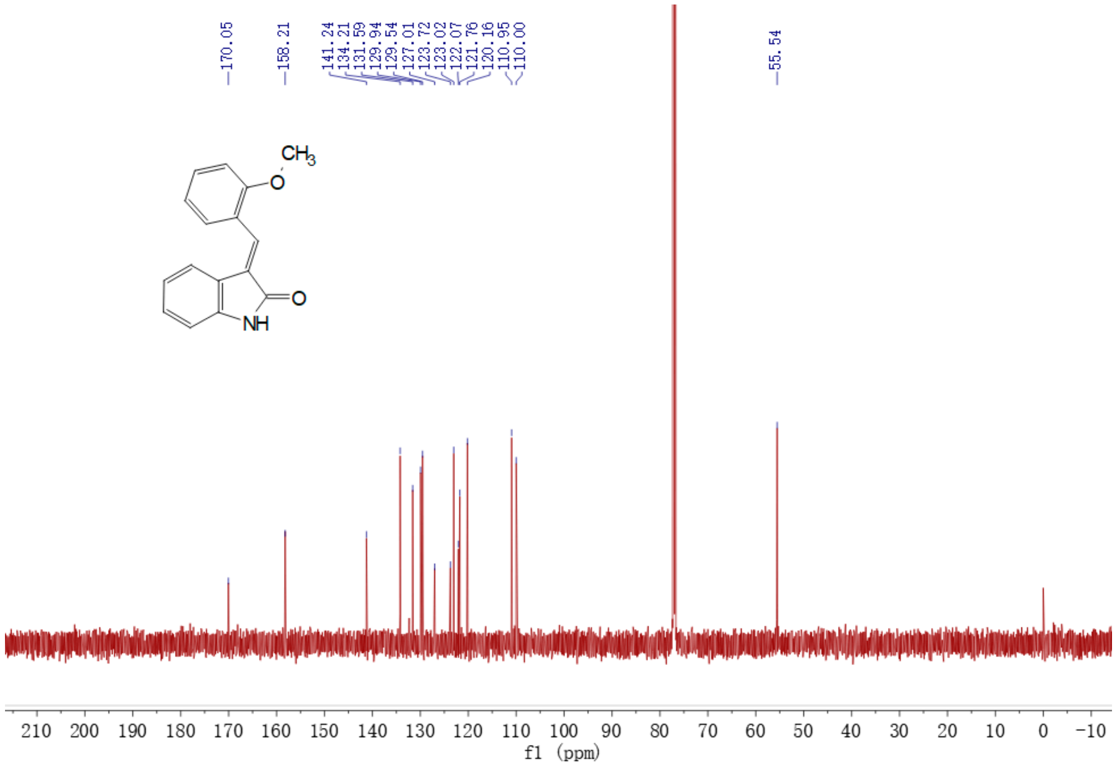

Fig. S44 The ^{13}C NMR (151 MHz, CDCl$_3$) of **2c**

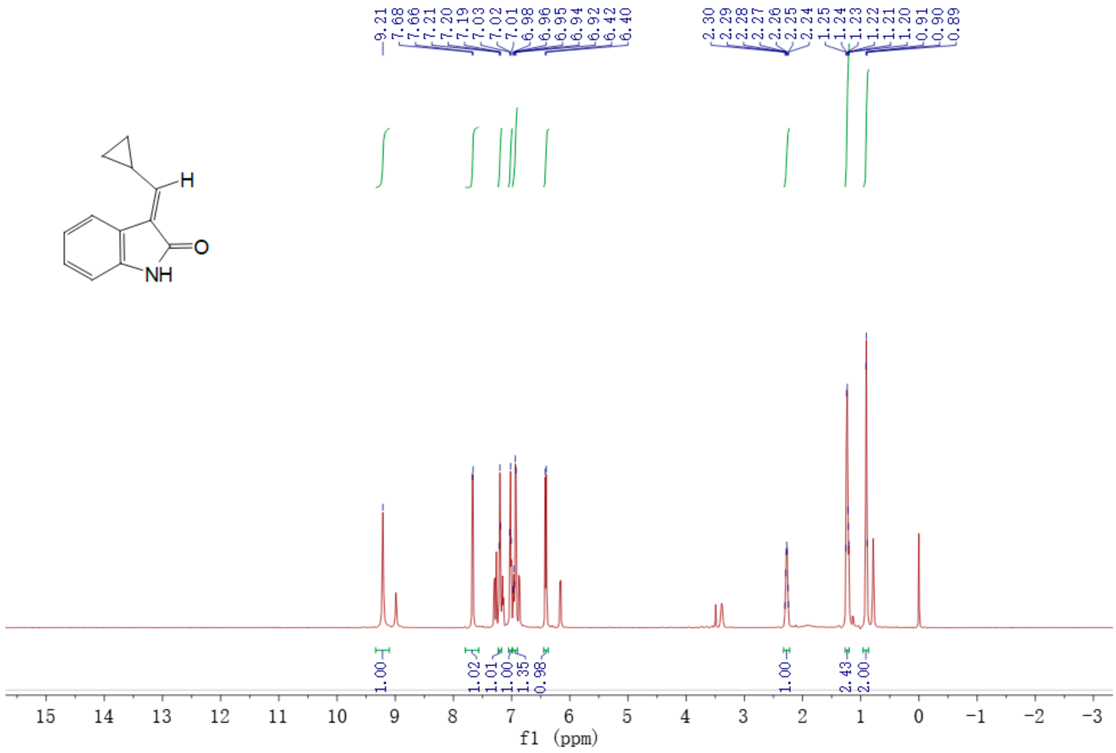

Fig. S45 The ^{1}H NMR (600 MHz, CDCl3) of **2d**

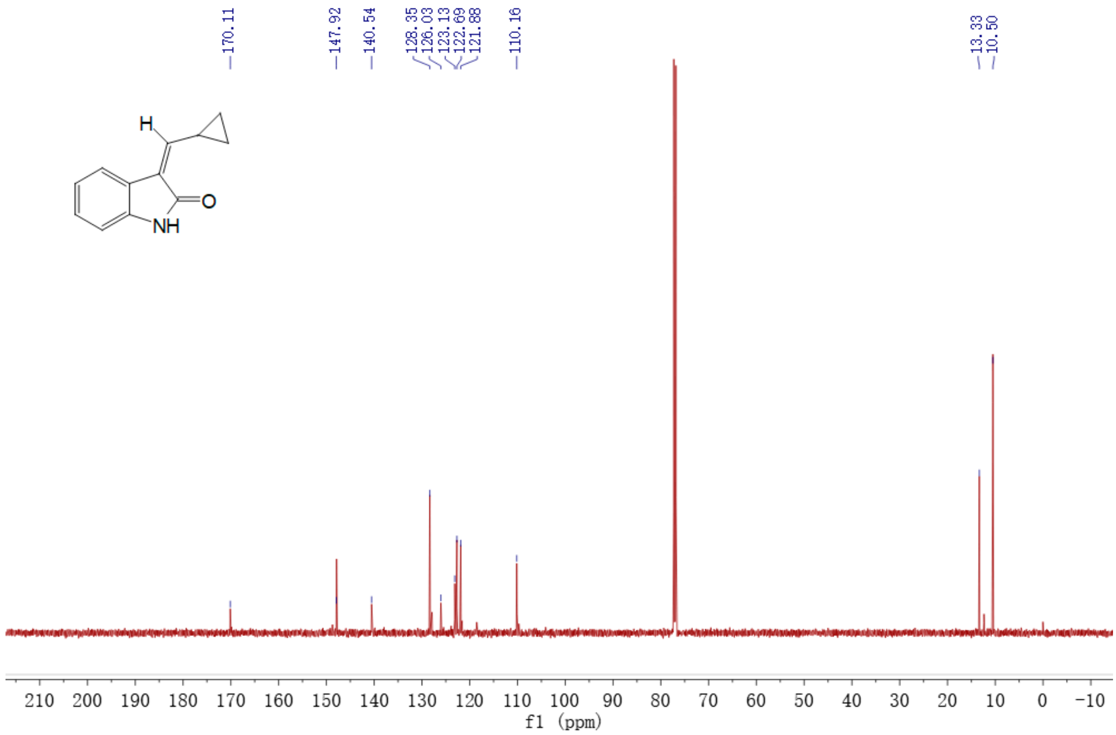

Fig. S46 The ^{13}C NMR (151 MHz, CDCl3) of **2d**

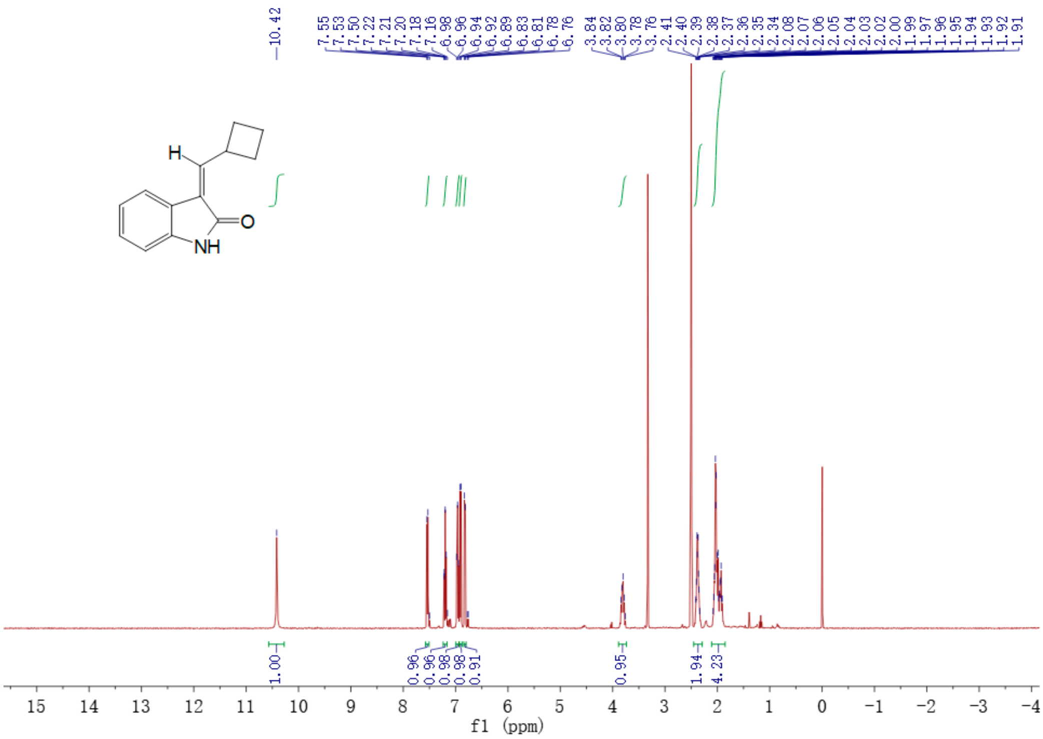

Fig. S47 The ^{1}H NMR (400 MHz, CDCl3) of **2e**

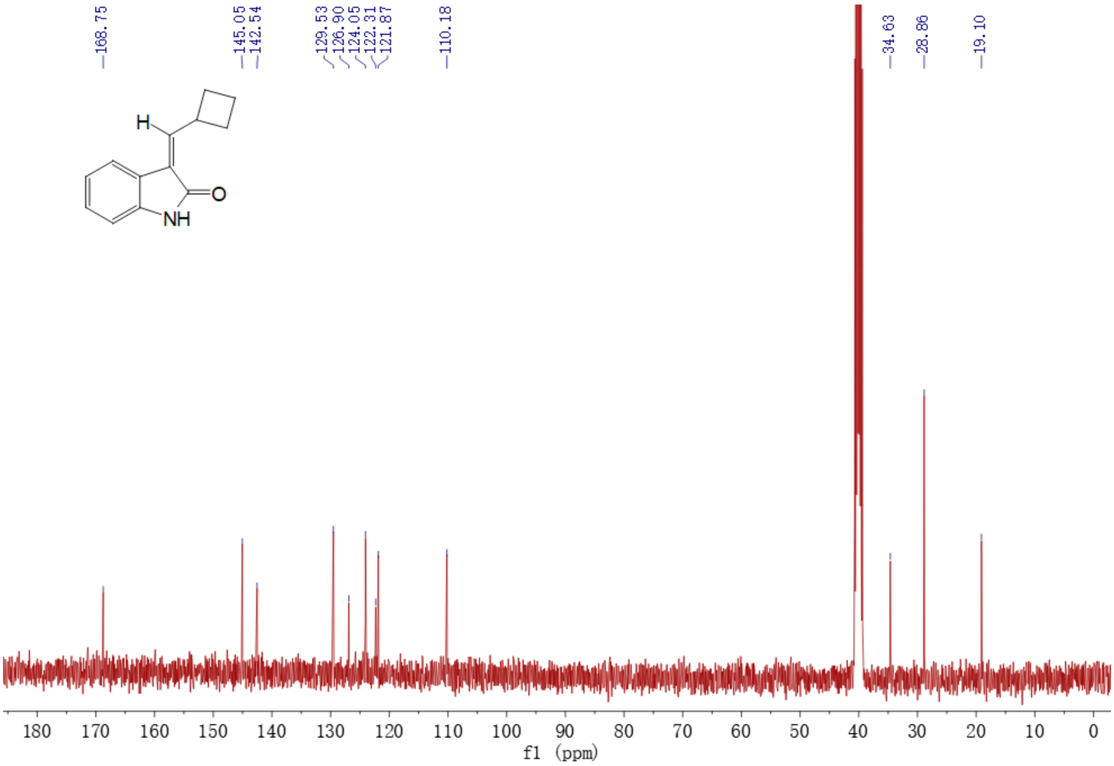

Fig. S48 The ^{13}C NMR (101 MHz, CDCl3) of **2e**

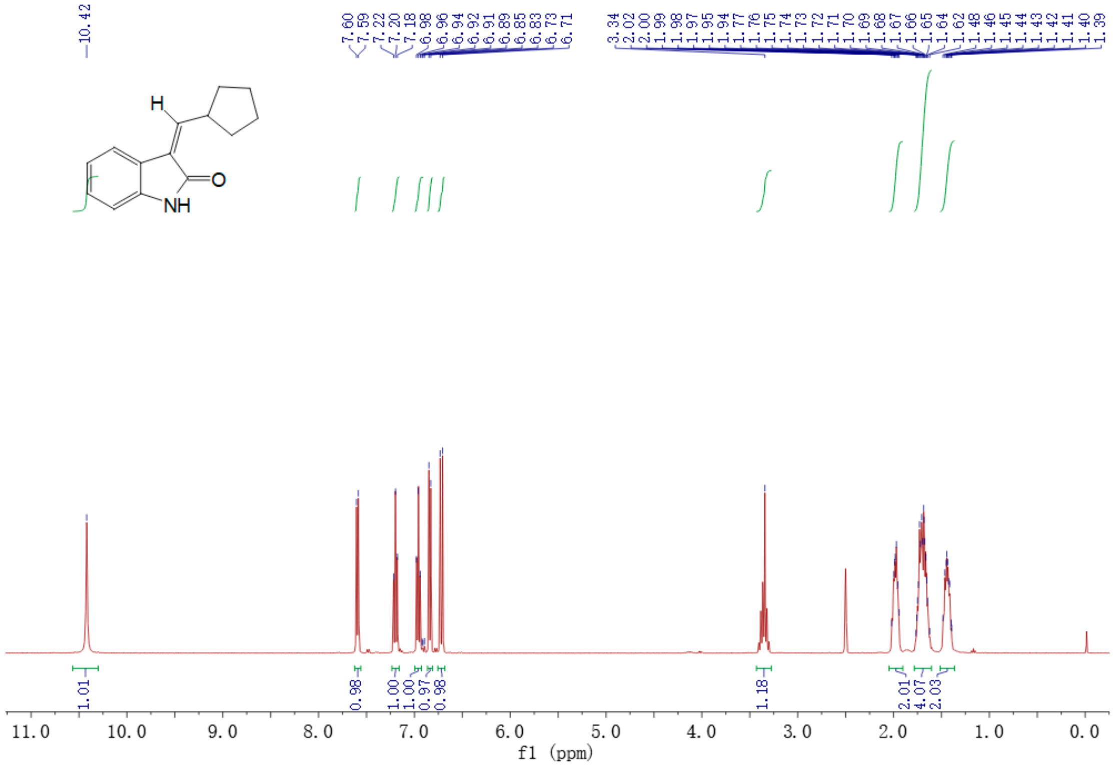

Fig. S49 The ^{1}H NMR (400 MHz, CDCl3) of **2f**

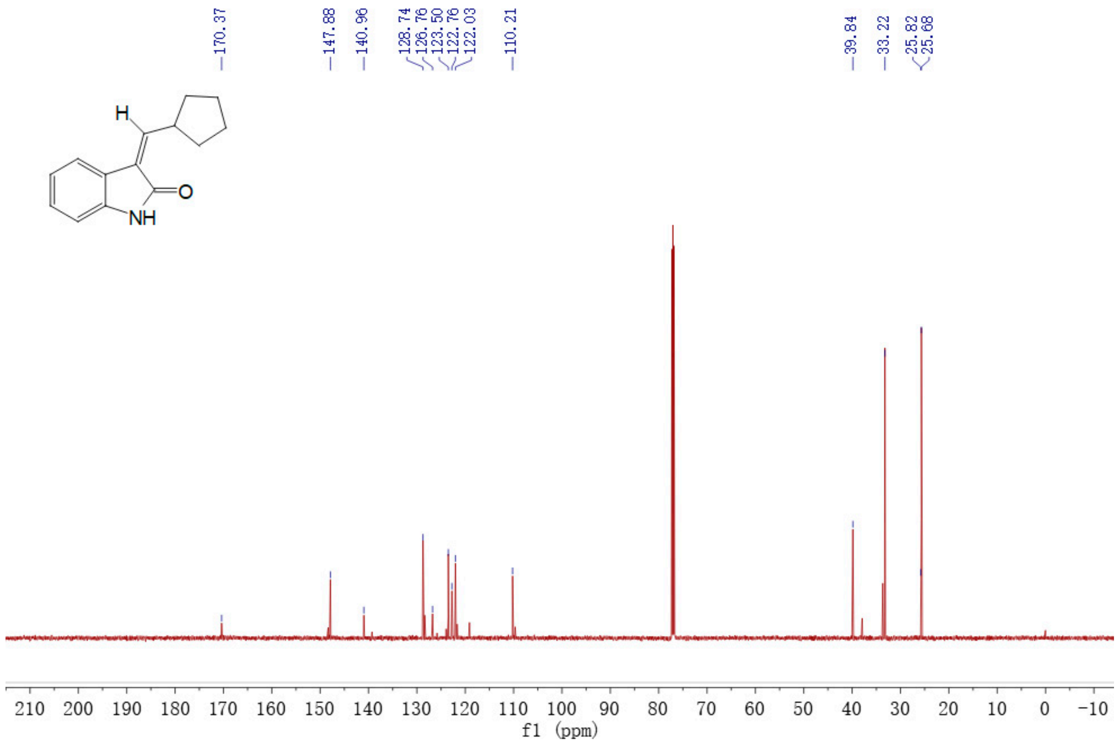

Fig. S50 The ^{13}C NMR (151 MHz, CDCl3) of **2f**

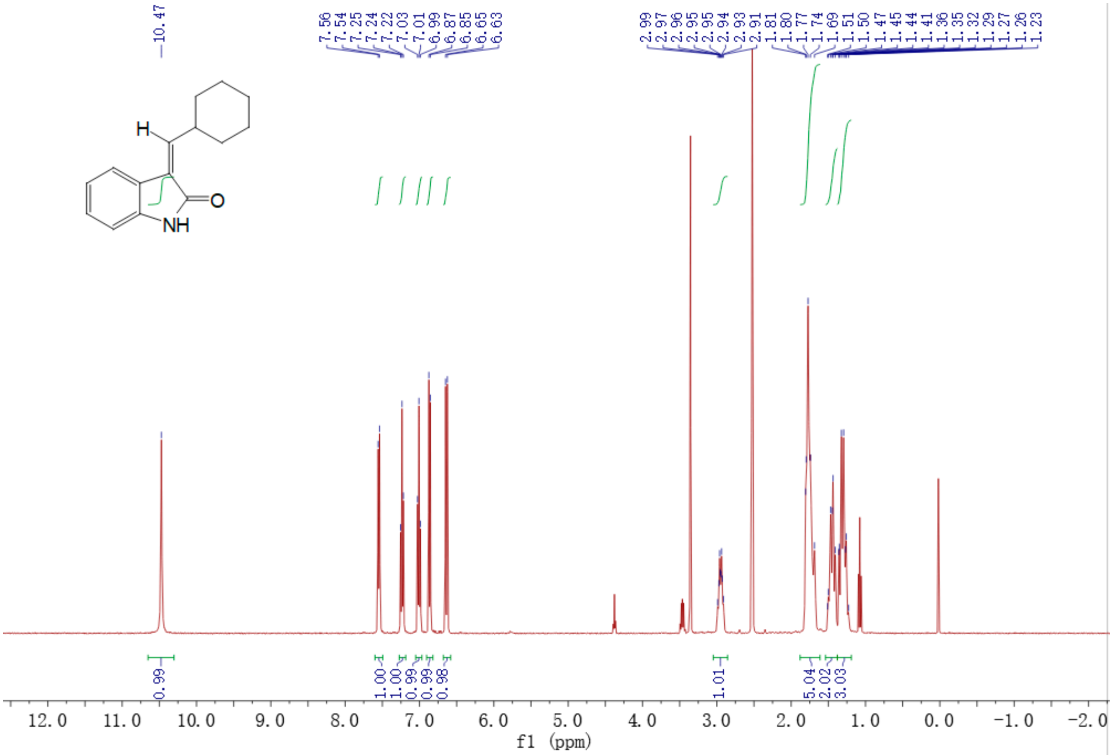

Fig. S51 The ^{1}H NMR (400 MHz, $CDCl_3$) of **2g**

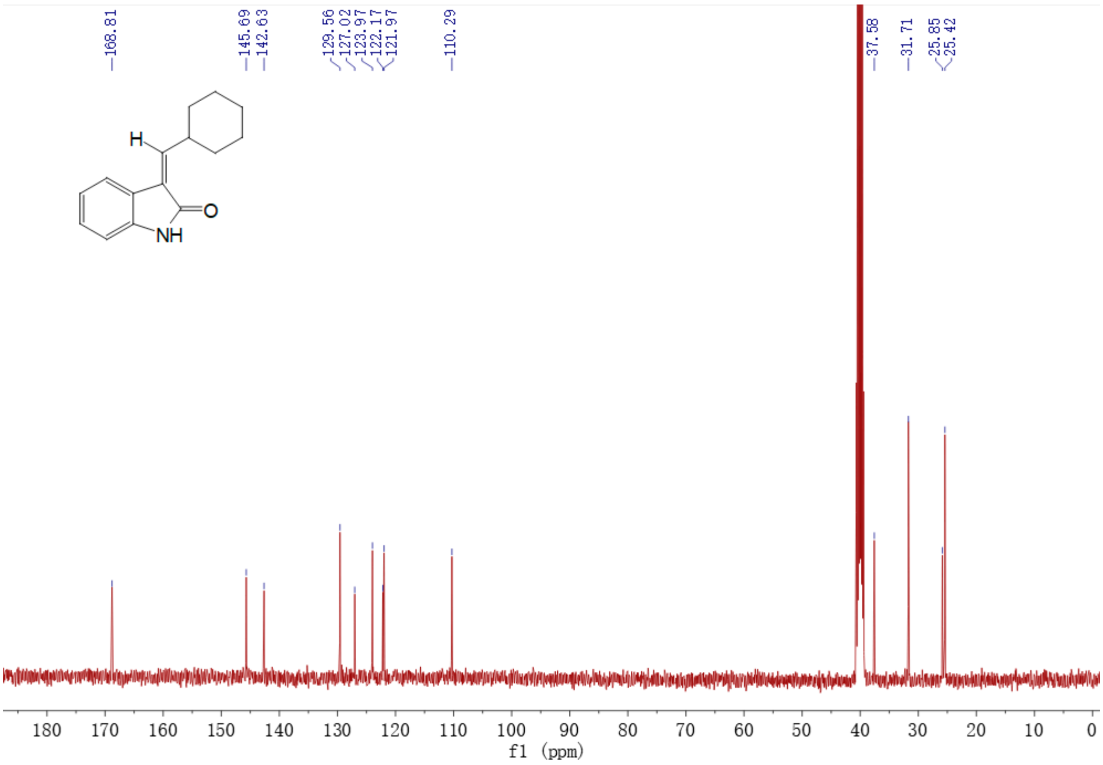

Fig. S52 The ^{13}C NMR (101 MHz, $CDCl_3$) of **2g**

6. Spectrums of spirooxindole intermediates 3a-3x

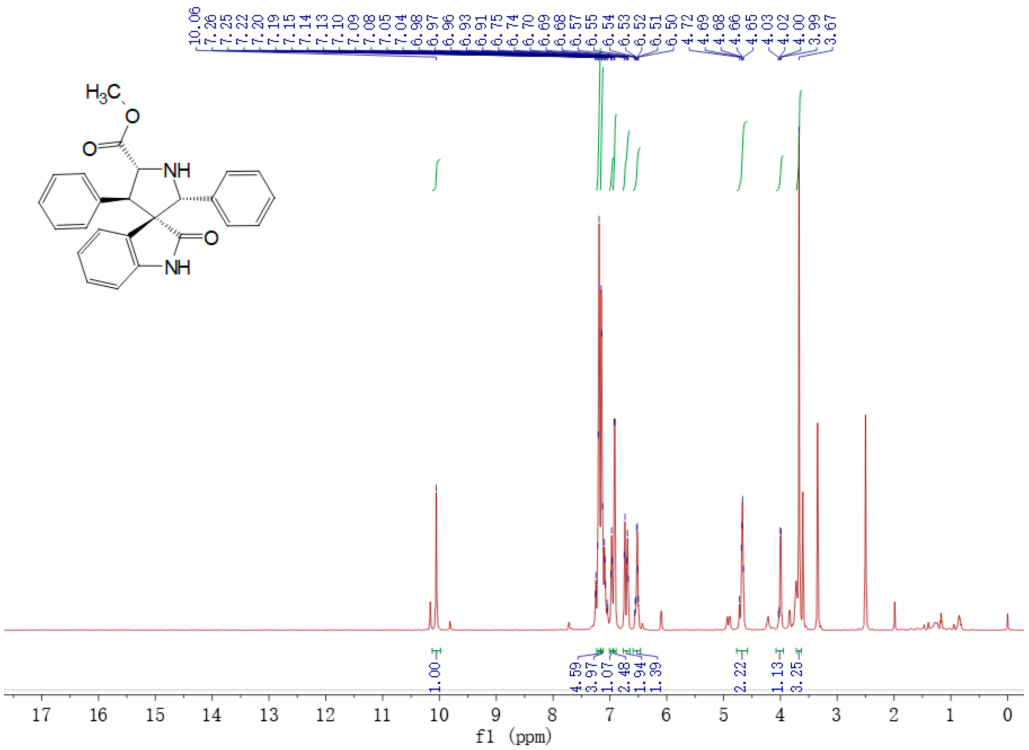

Fig. S53 The ^{1}H NMR (600 MHz, DMSO-d$_6$) of **3a**

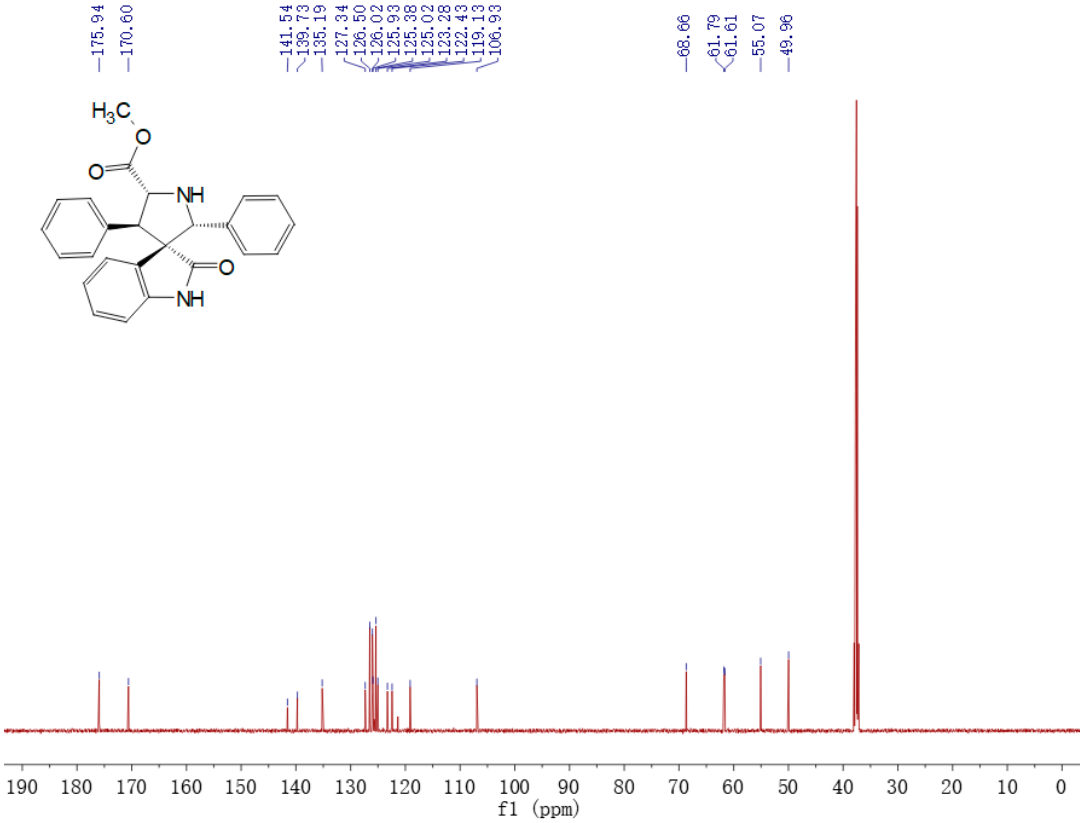

Fig. S54 The ^{13}C NMR (151 MHz, DMSO-d$_6$) of **3a**

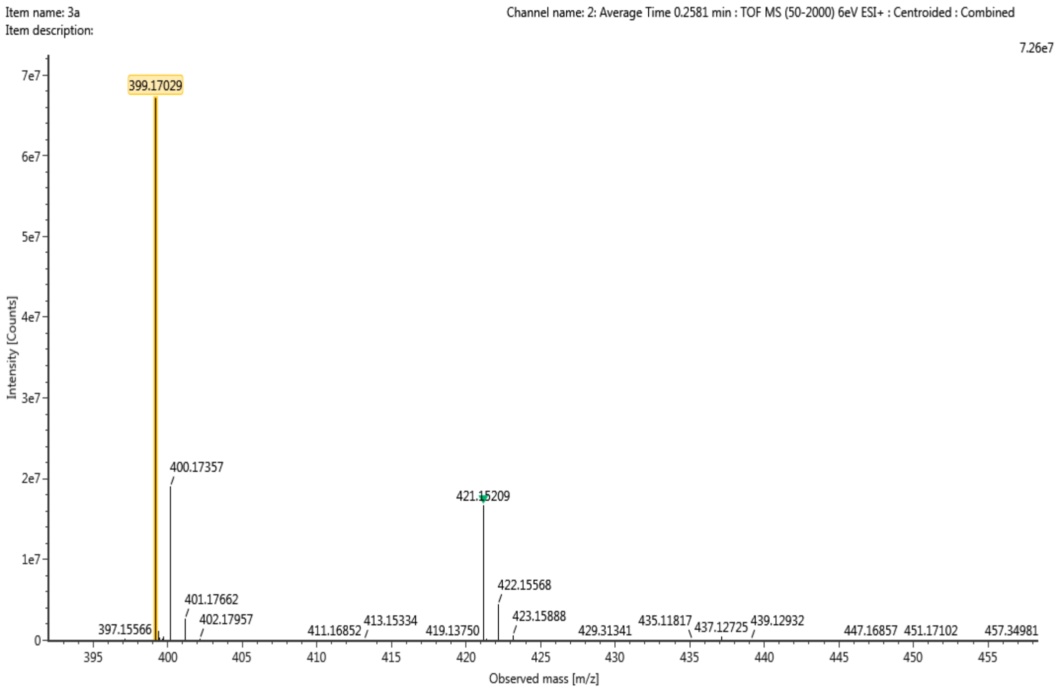

Fig. S55 The Mass spectrogram of **3a**

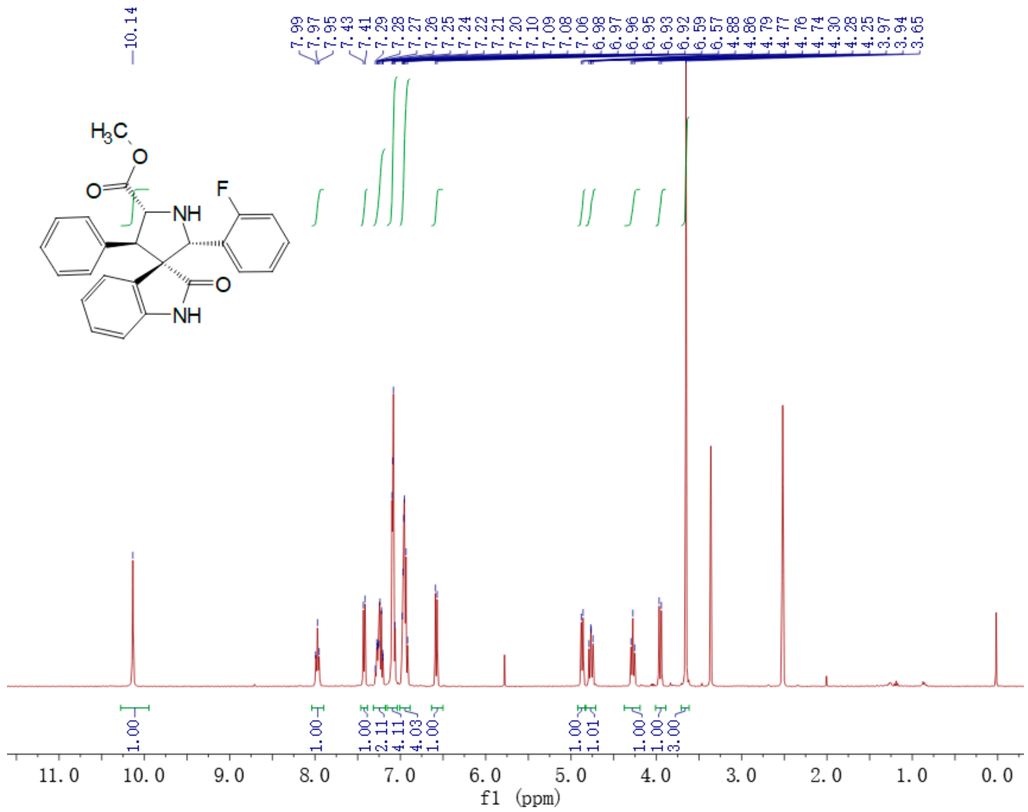

Fig. S56 The ^{1}H NMR (400 MHz, DMSO-d_6) of **3b**

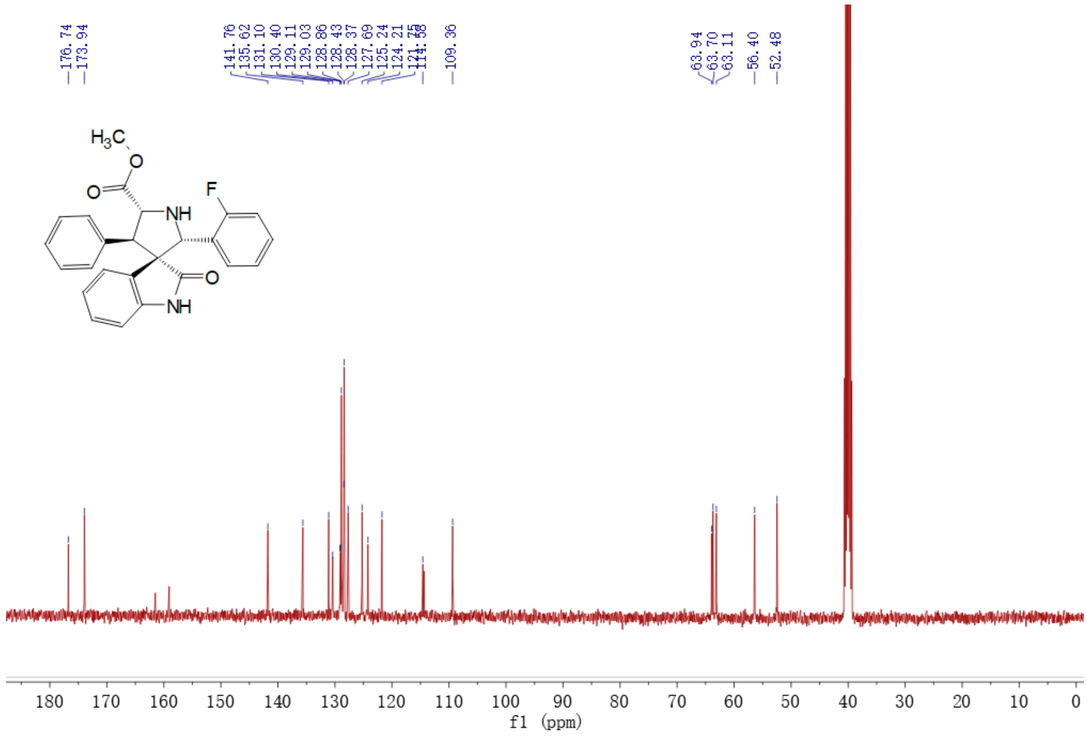

Fig. S57 The ^{13}C NMR (101 MHz, DMSO-d_6) of **3b**

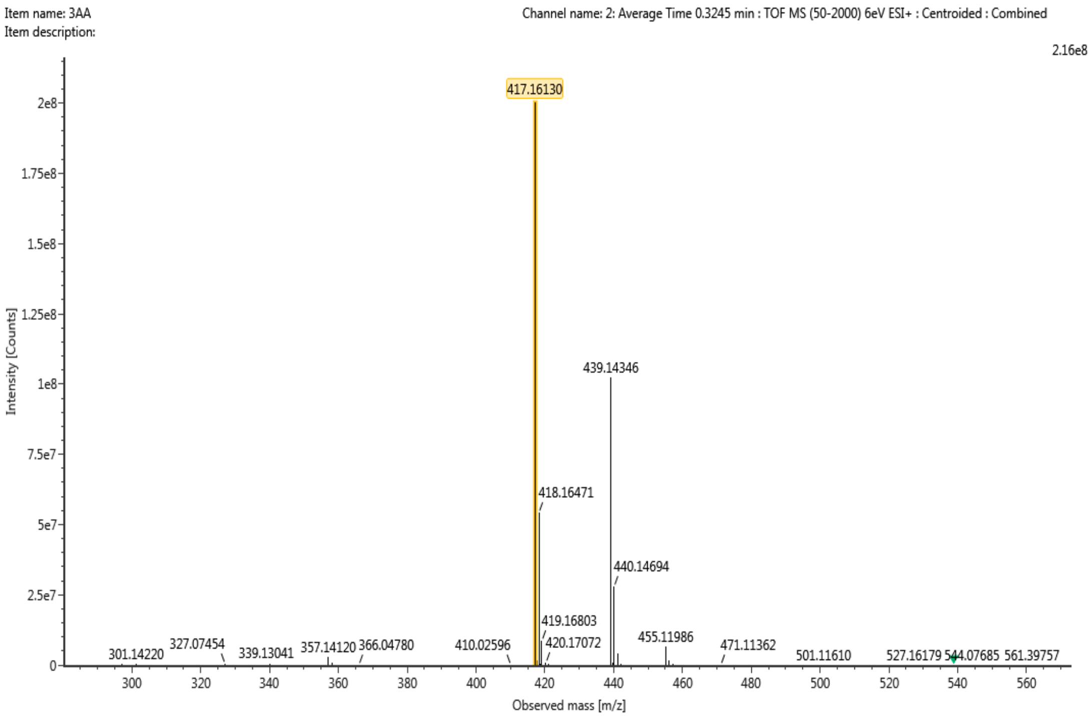

Fig. S58 The Mass spectrogram of **3b**

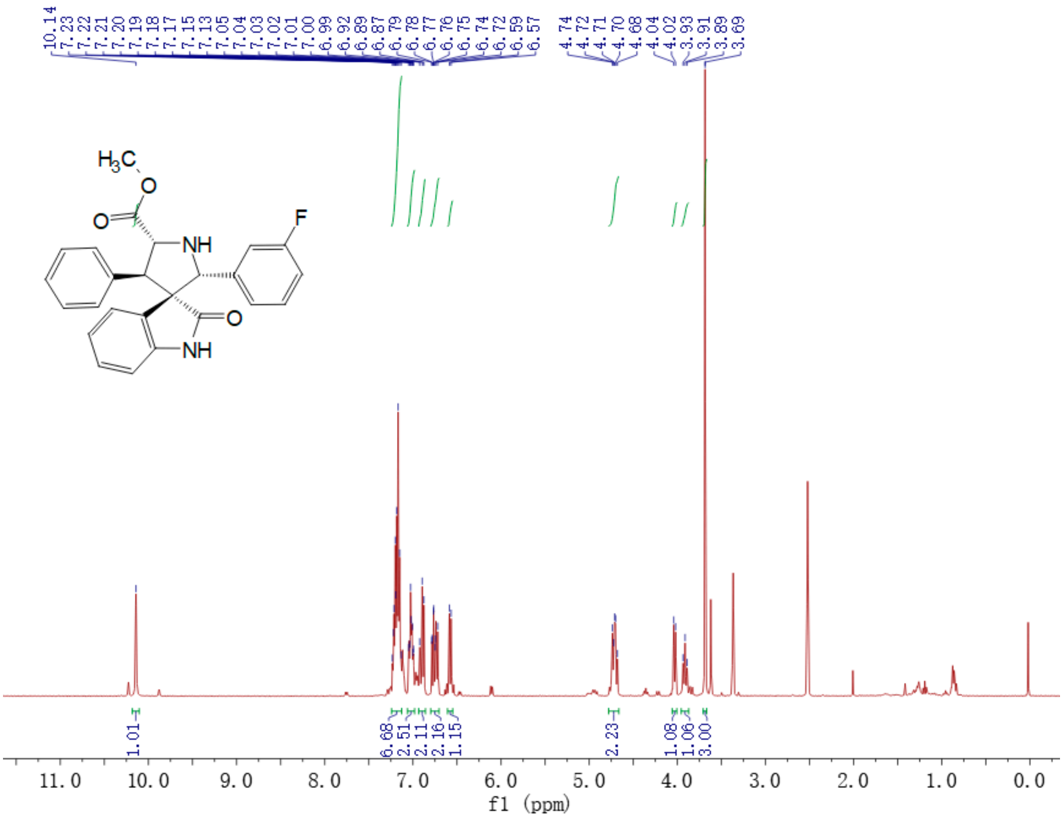

Fig. S59 The ^{1}H NMR (400 MHz, DMSO-d$_6$) of **3c**

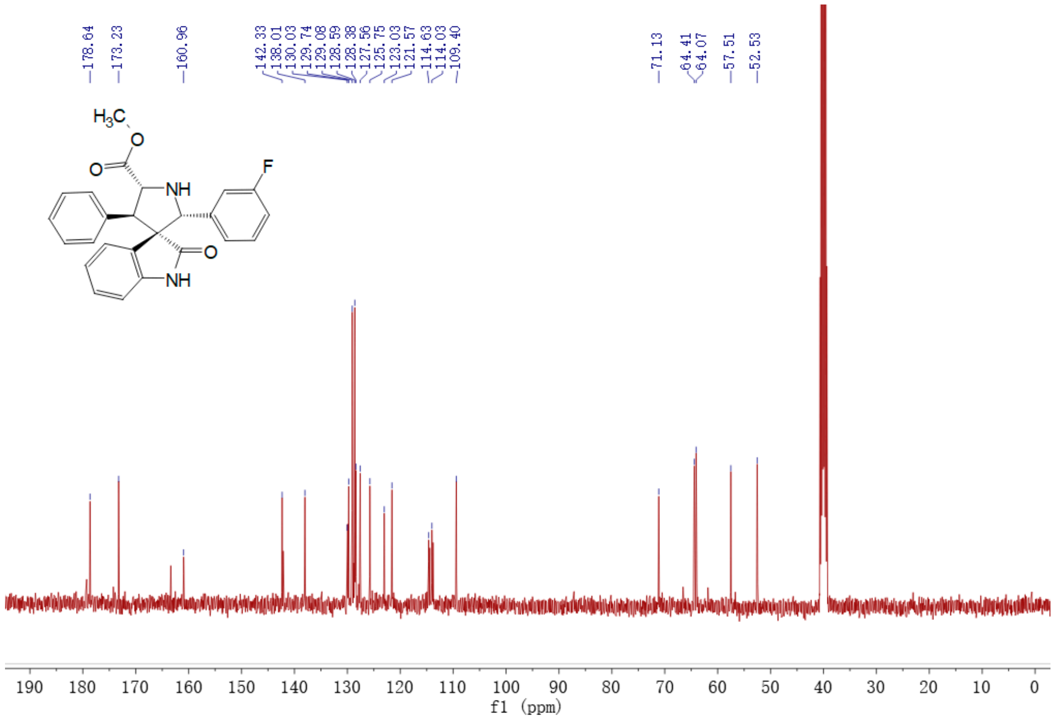

Fig. S60 The ^{13}C NMR (101 MHz, DMSO-d$_6$) of **3c**

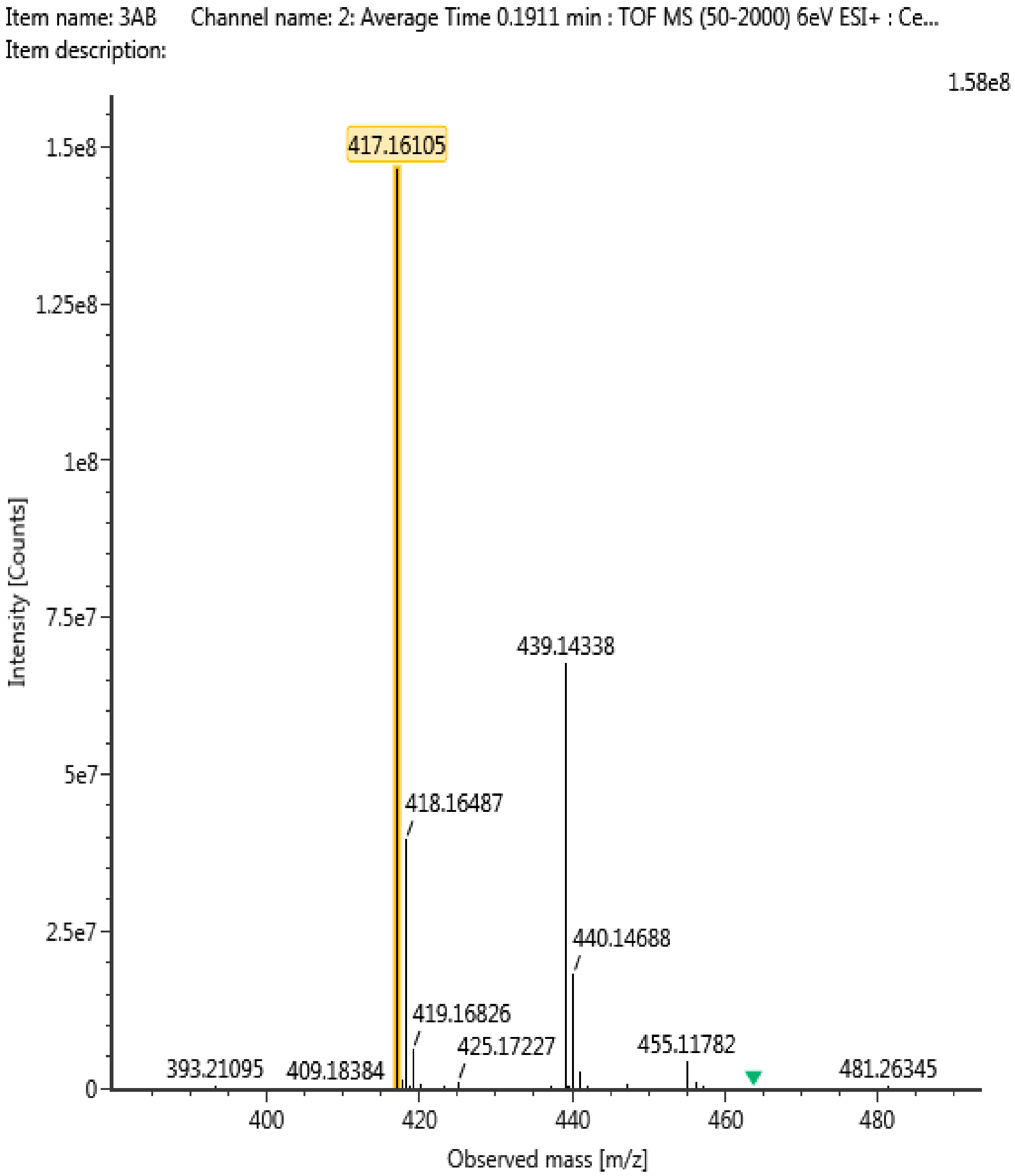

Fig. S61 The Mass spectrogram of **3c**

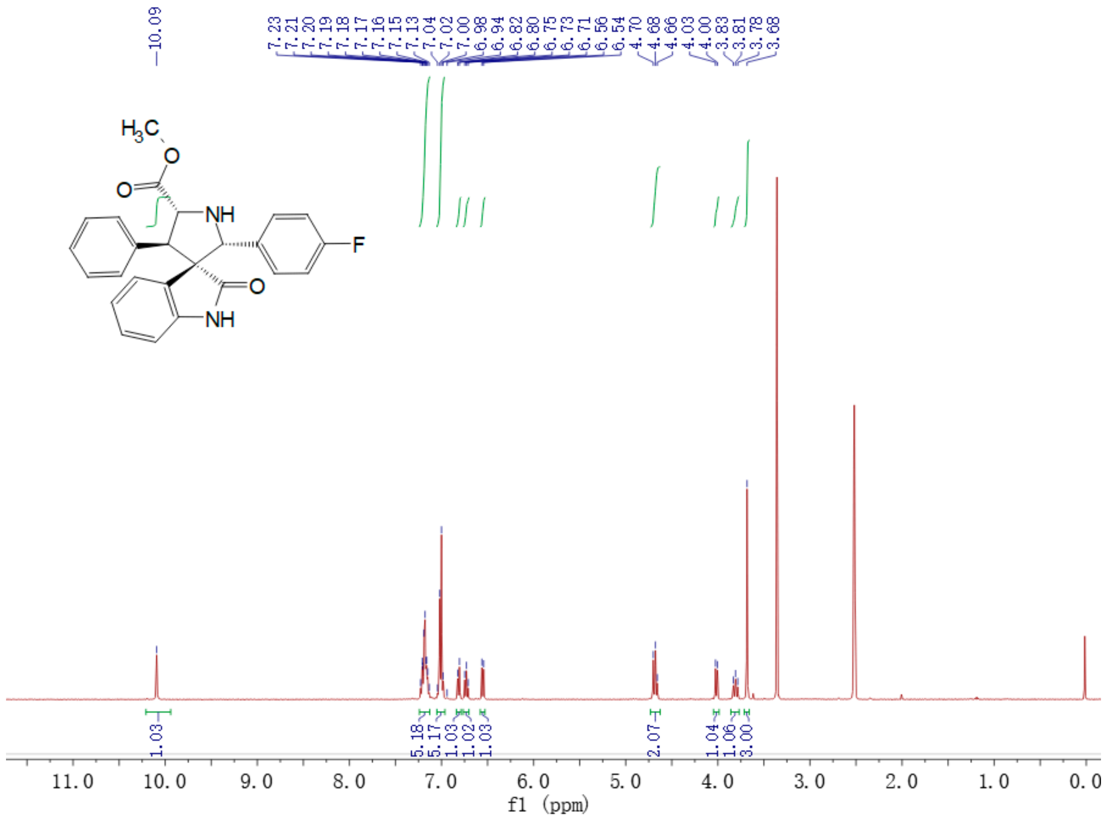

Fig. S62 The ^{1}H NMR (400 MHz, DMSO-d6) of **3d**

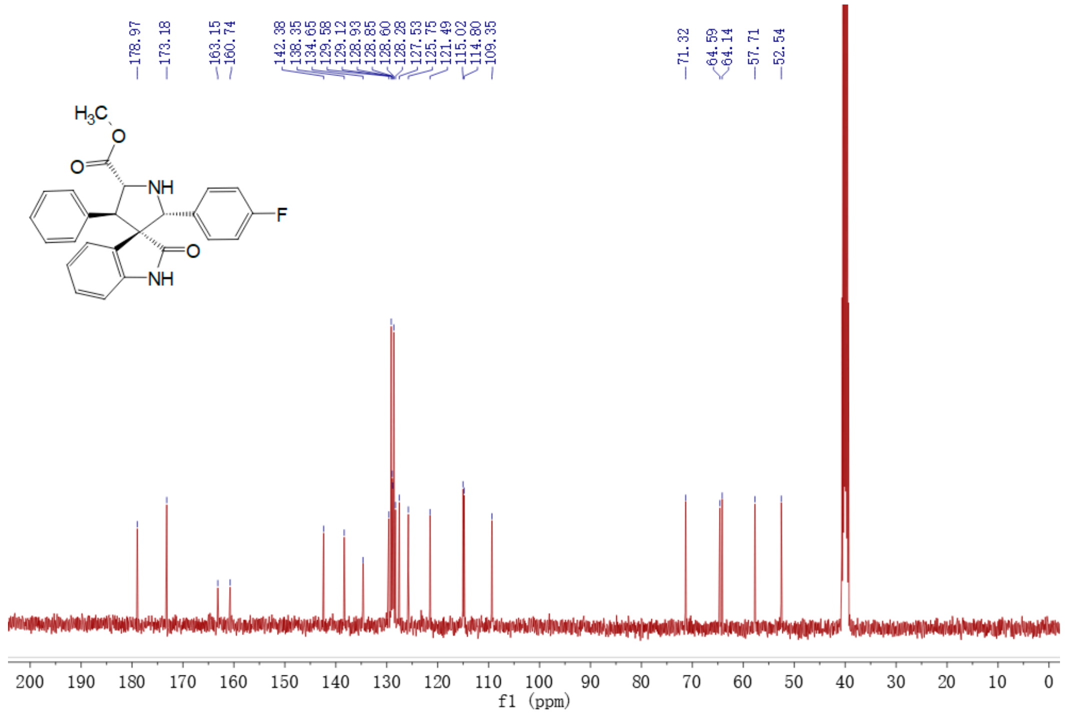

Fig. S63 The ^{13}C NMR (101 MHz, DMSO-d6) of **3d**

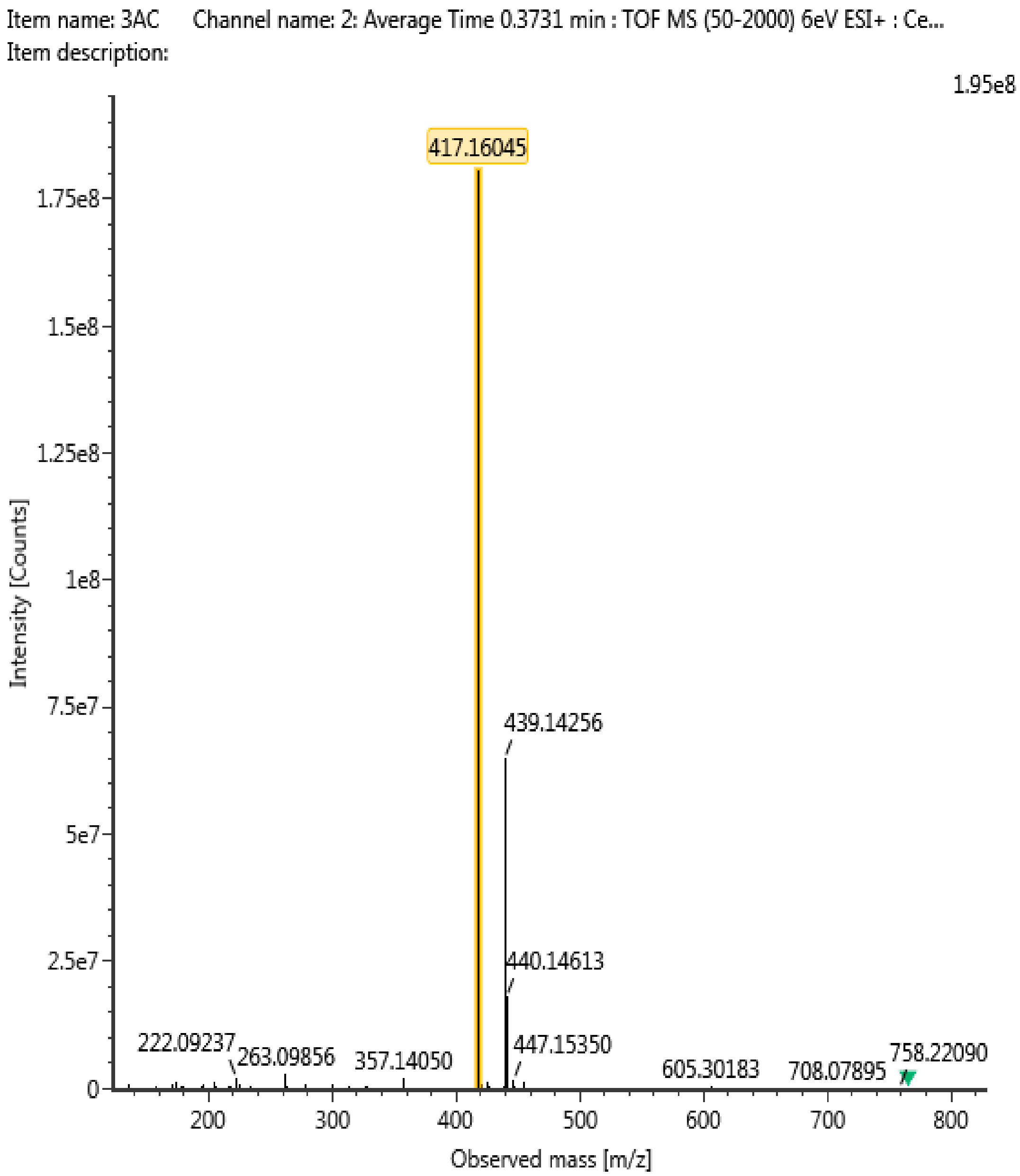

Fig. S64 The Mass spectrogram of **3d**

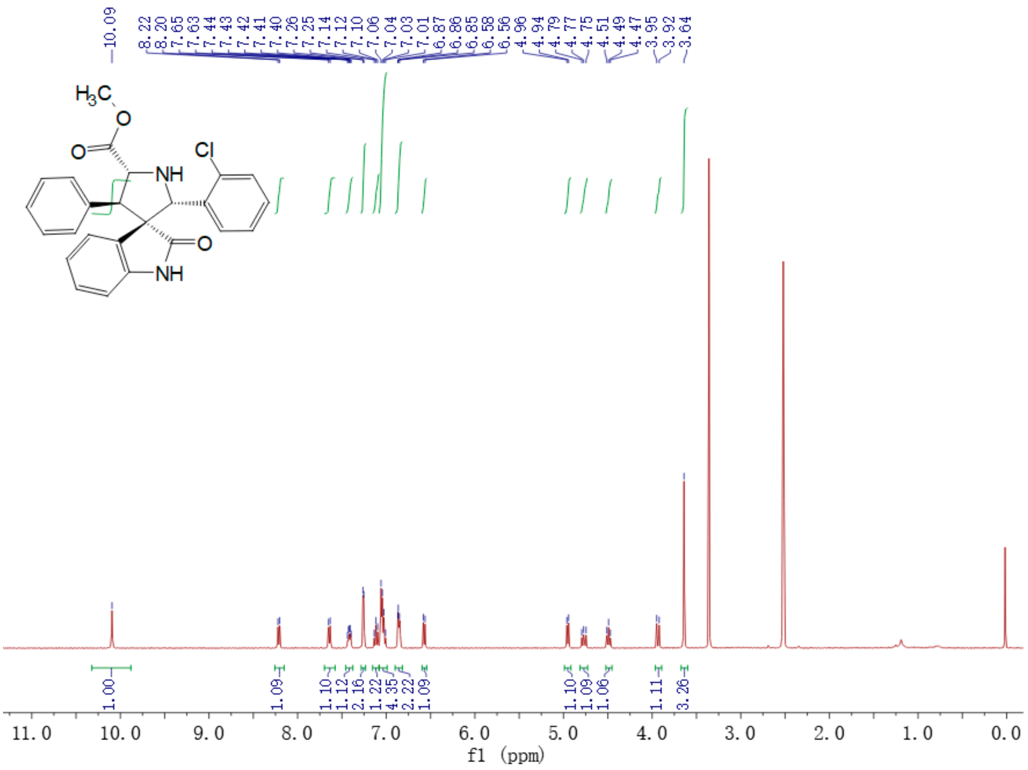

Fig. S65 The ^{1}H NMR (400 MHz, DMSO-d$_6$) of **3e**

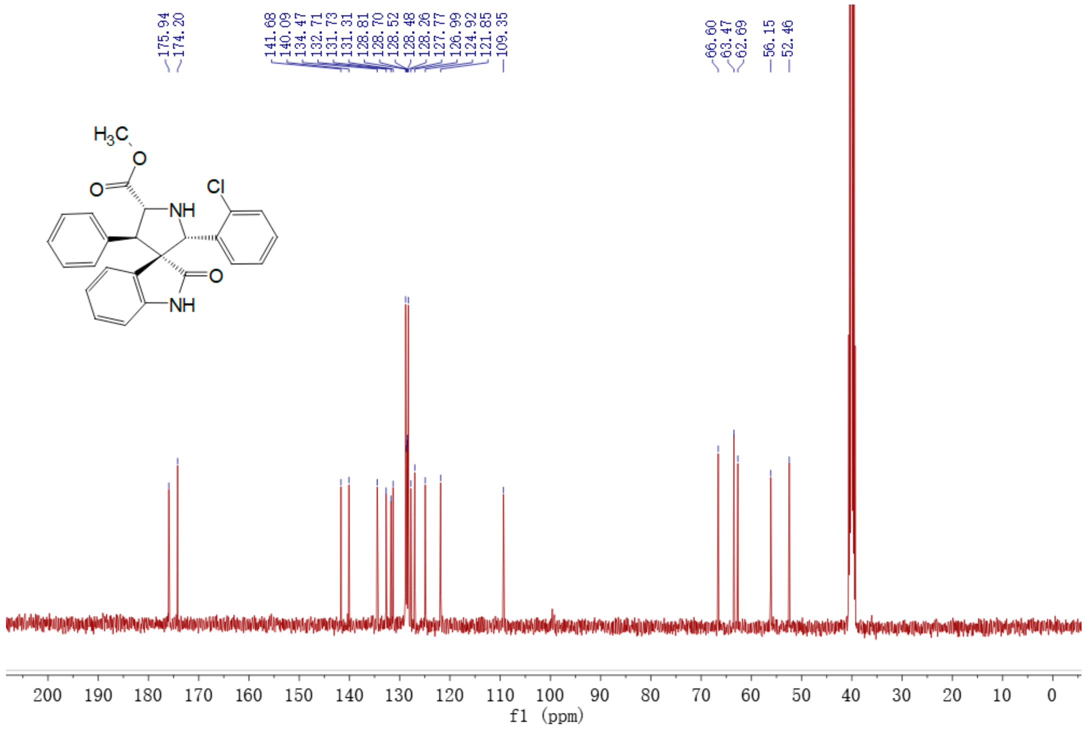

Fig. S66 The ^{13}C NMR (101 MHz, DMSO-d$_6$) of **3e**

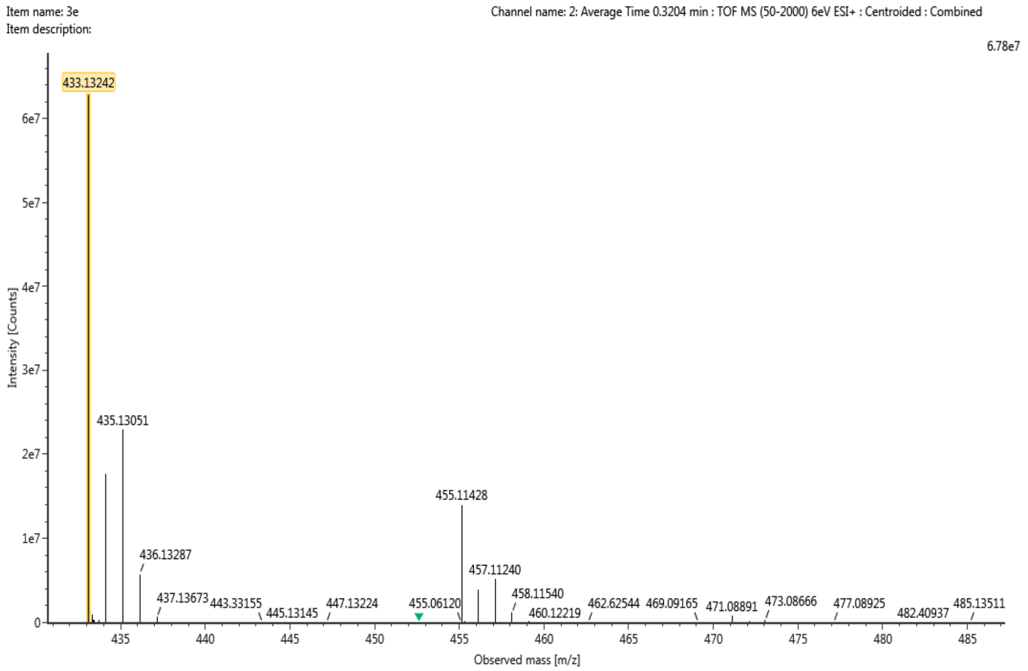

Fig. S67 The Mass spectrogram of **3e**

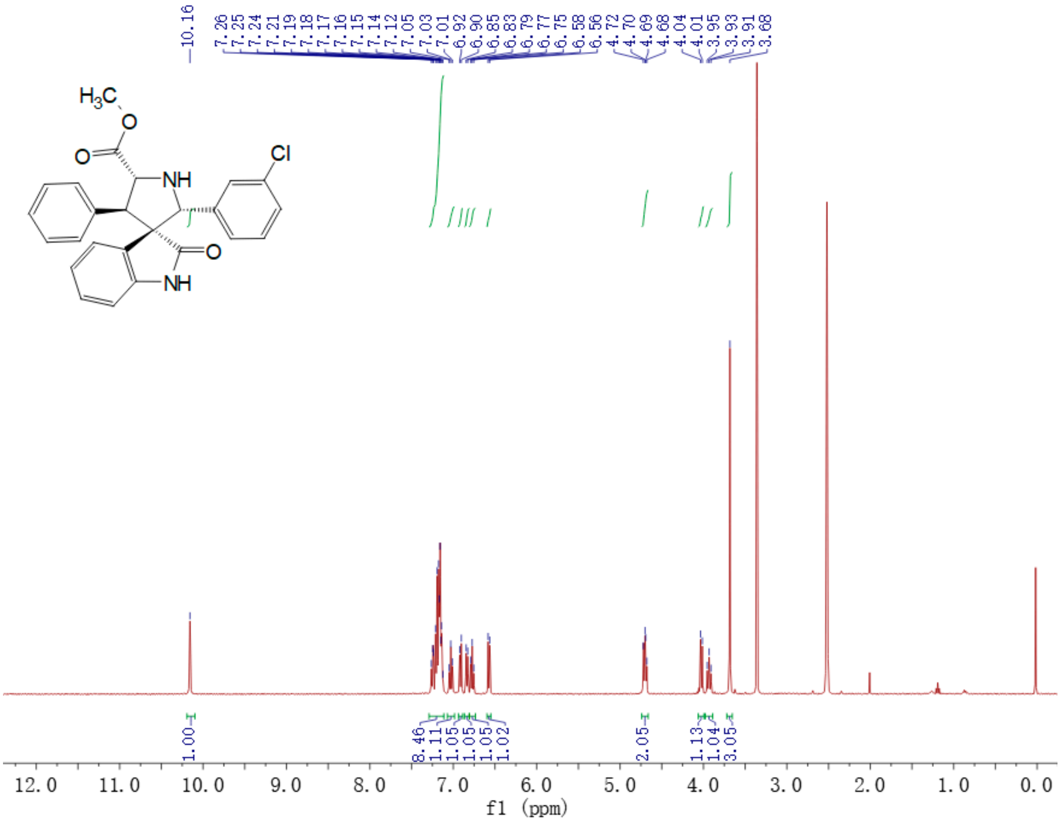

Fig. S68 The ^{1}H NMR (400 MHz, DMSO-d$_6$) of **3f**

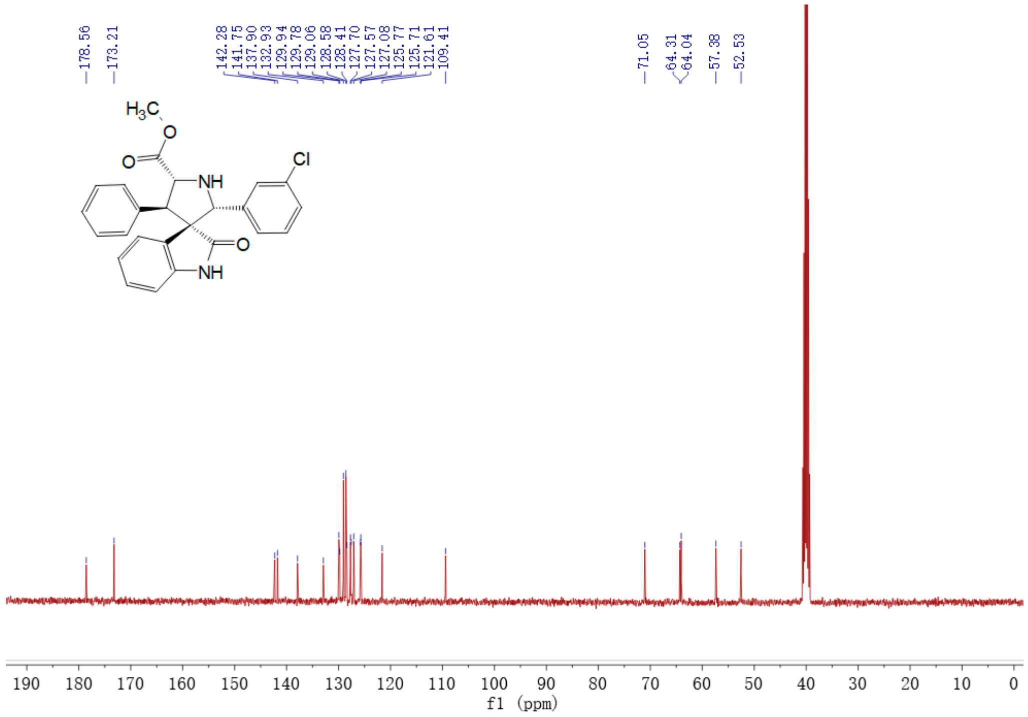

Fig. S69 The ^{13}C NMR (101 MHz, DMSO-d$_6$) of **3f**

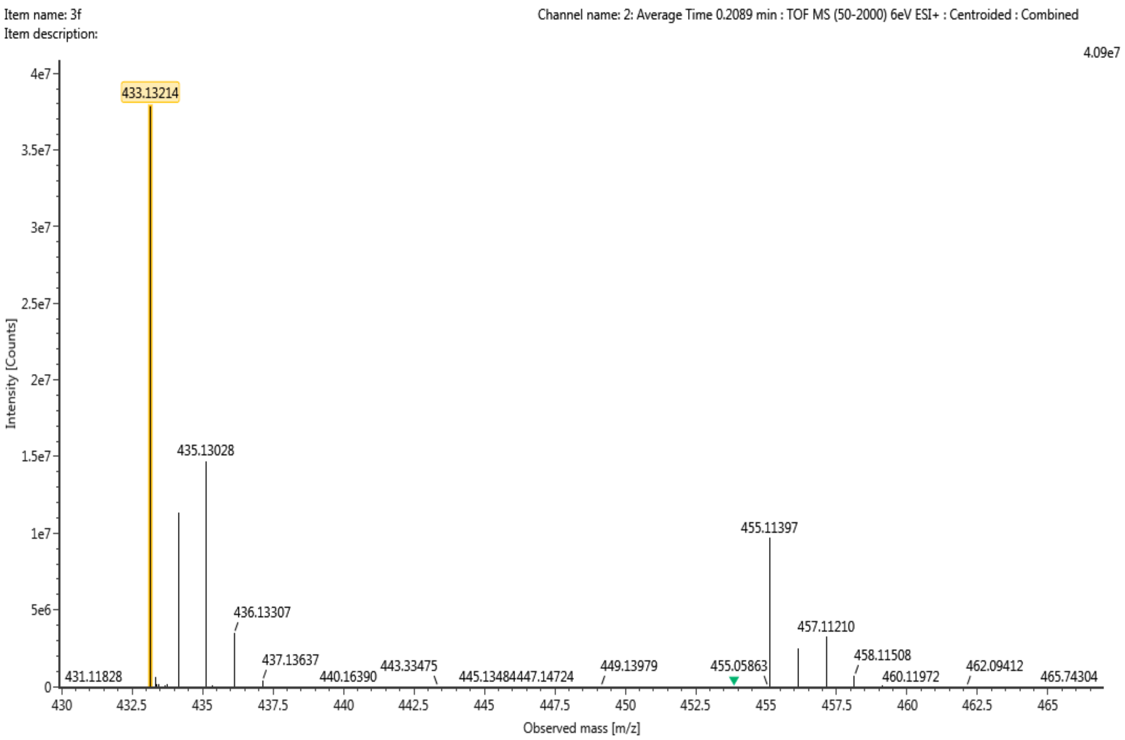

Fig. S70 The Mass spectrogram of **3f**

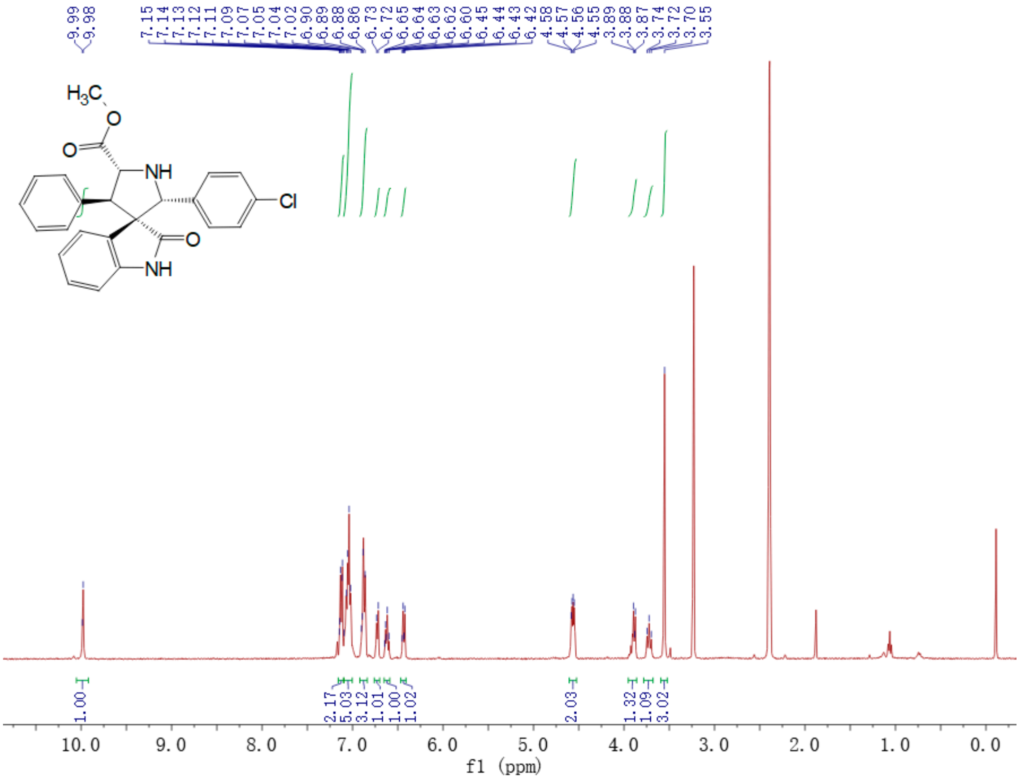

Fig. S71 The ^{1}H NMR (400 MHz, DMSO-d6) of **3g**

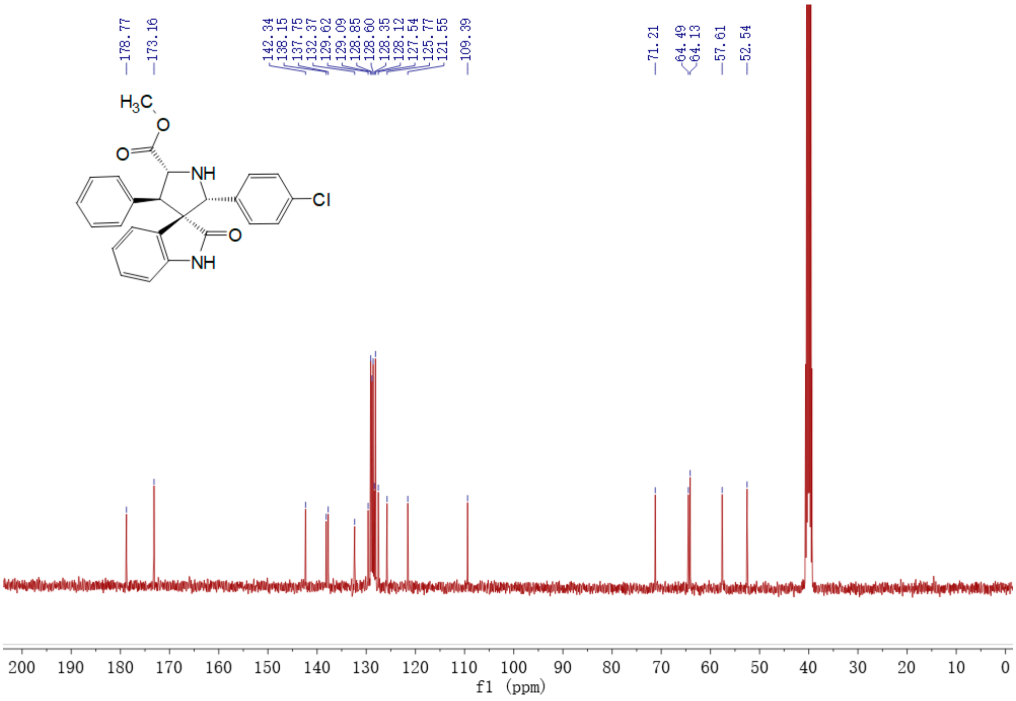

Fig. S72 The ^{13}C NMR (101 MHz, DMSO-d6) of **3g**

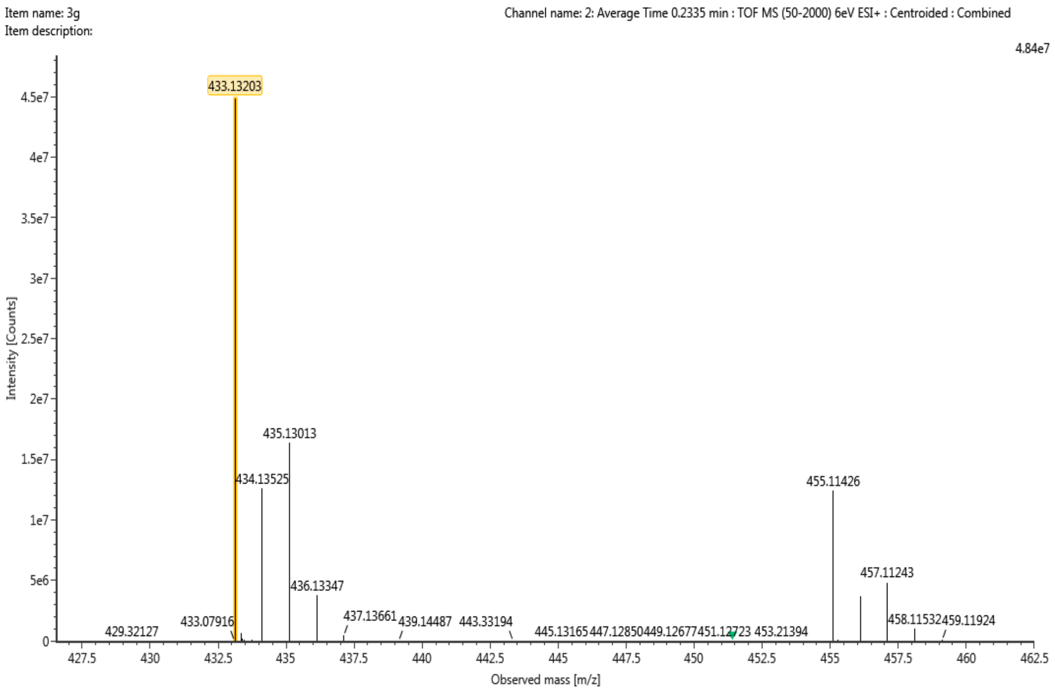

Fig. S73 The Mass spectrogram of **3g**

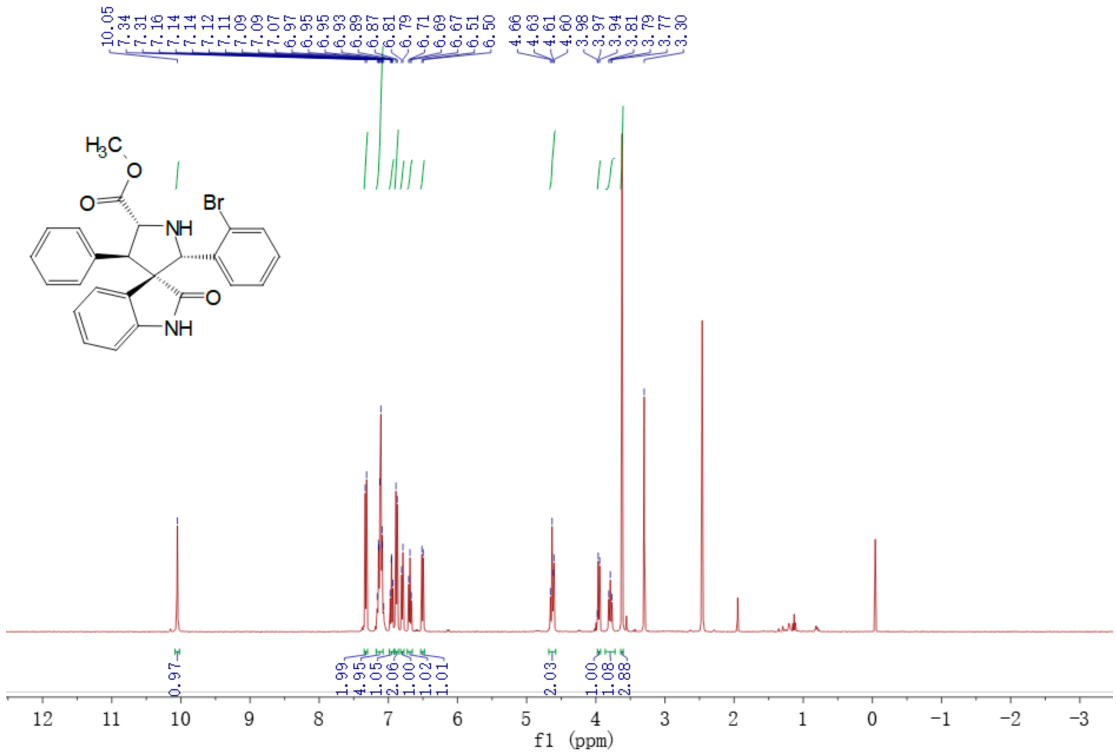

Fig. S74 The ^{1}H NMR (400 MHz, DMSO-d_6) of **3h**

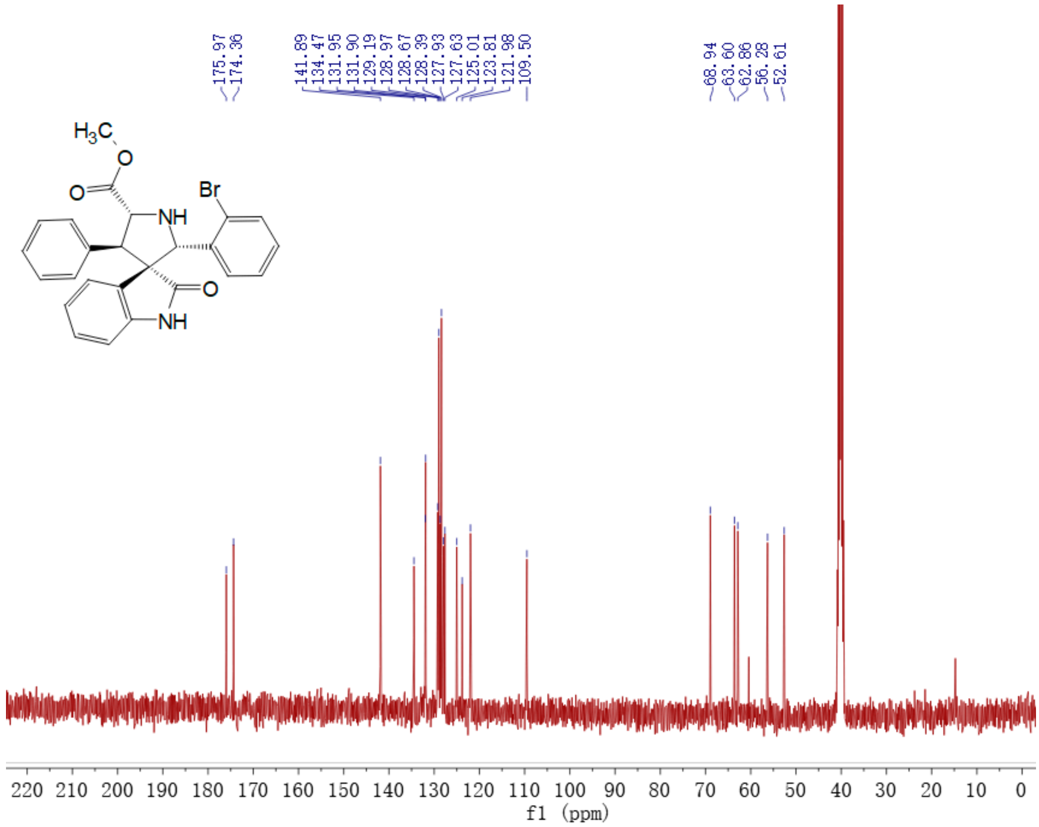

Fig. S75 The ^{13}C NMR (101 MHz, DMSO-d_6) of **3h**

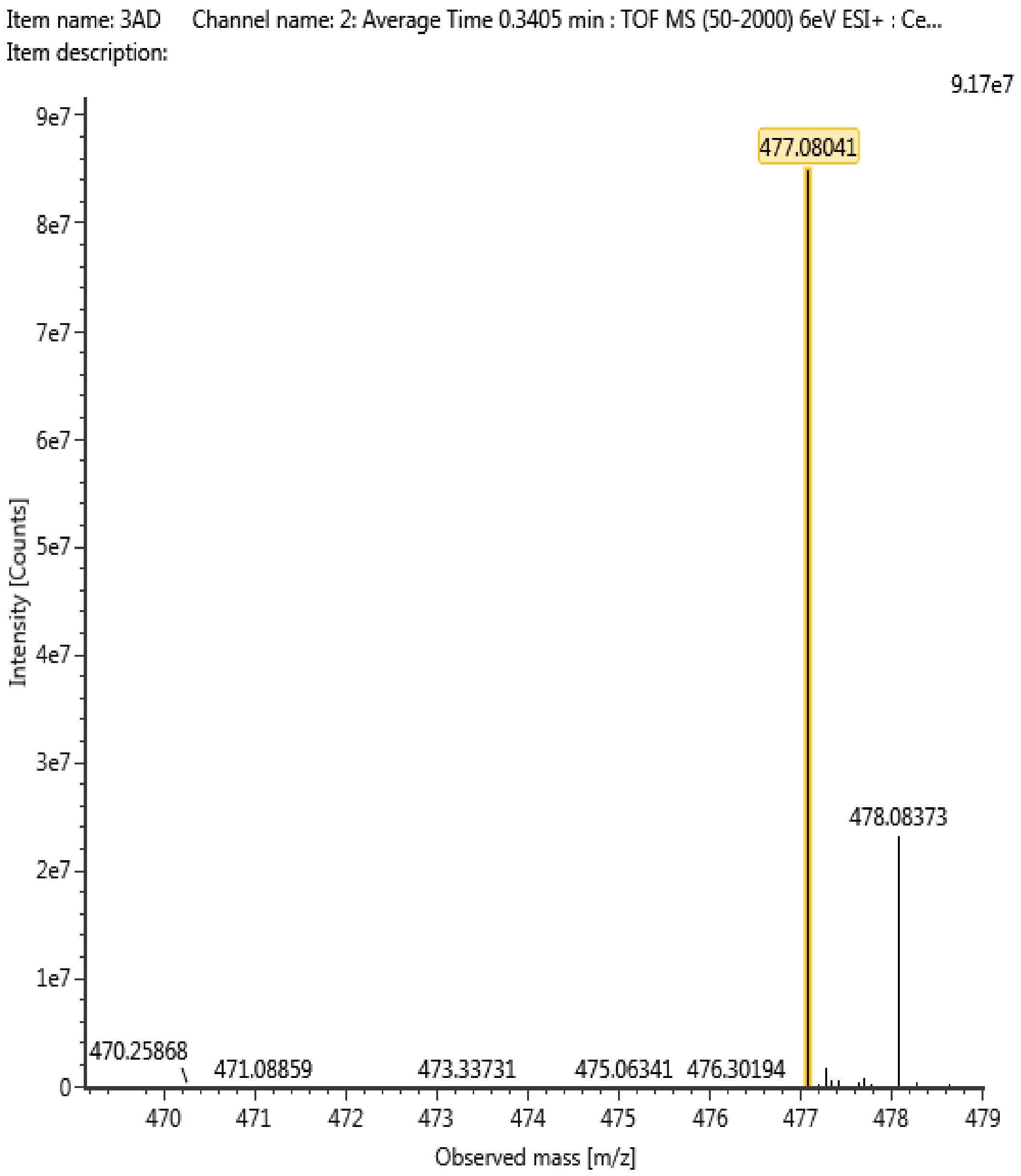

Fig. S76 The Mass spectrogram of **3h**

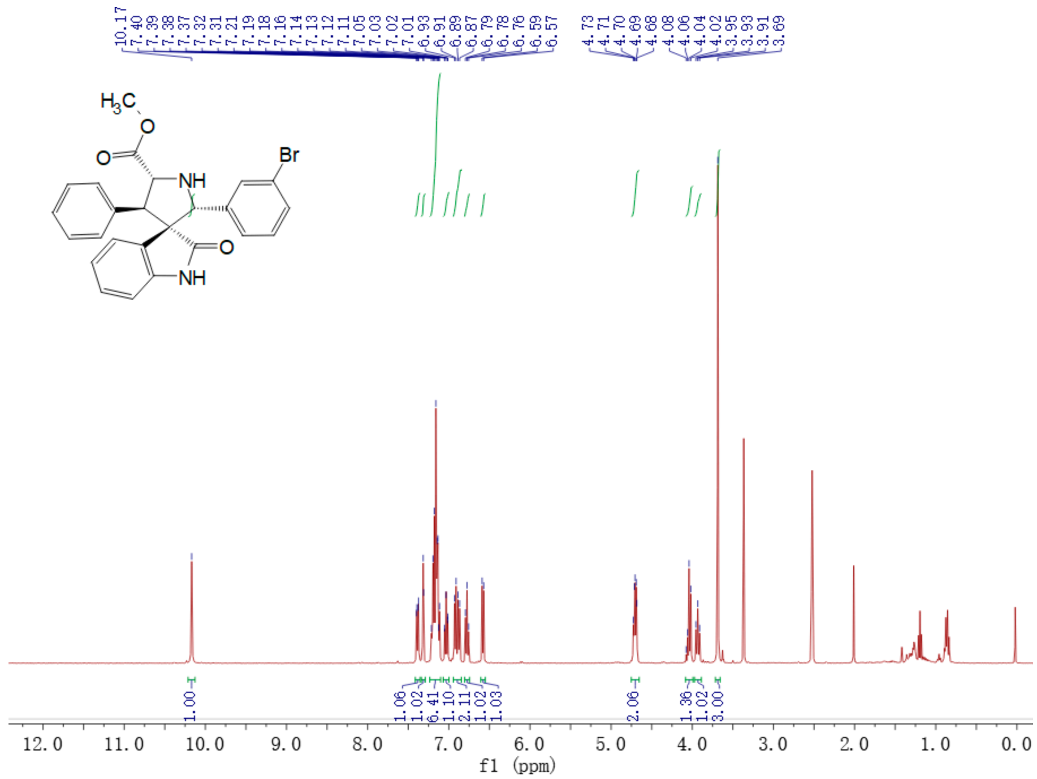

Fig. S77 The ^{1}H NMR (400 MHz, DMSO-d$_6$) of **3i**

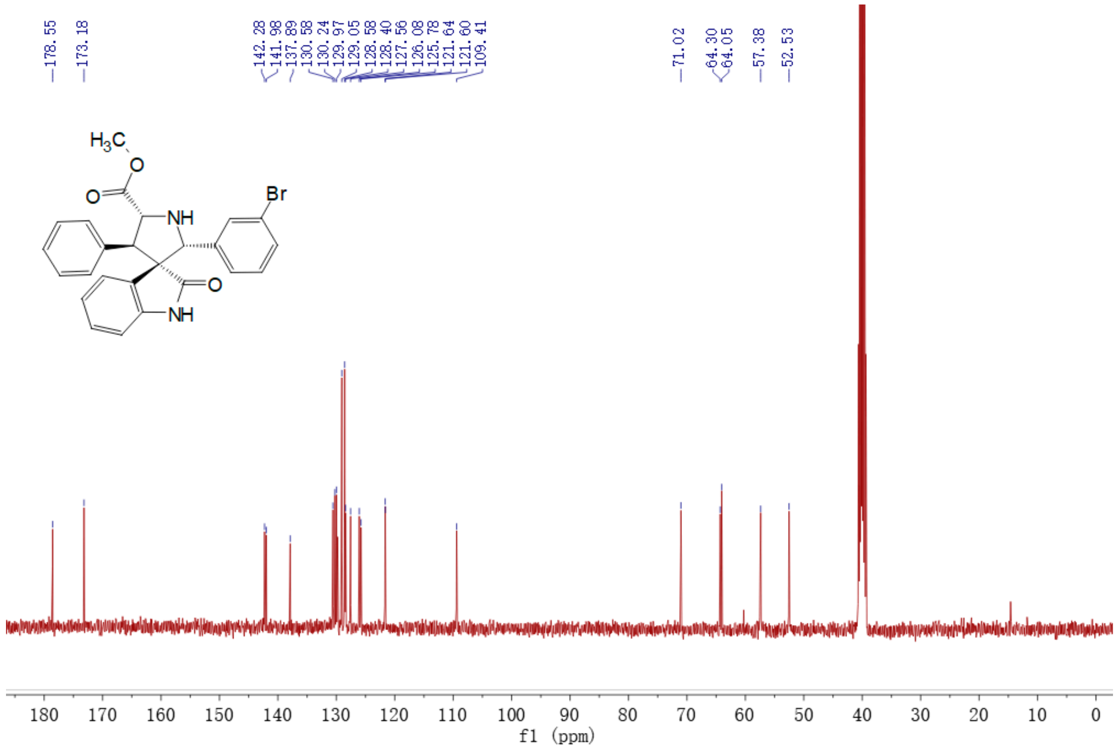

Fig. S78 The ^{13}C NMR (101 MHz, DMSO-d$_6$) of **3i**

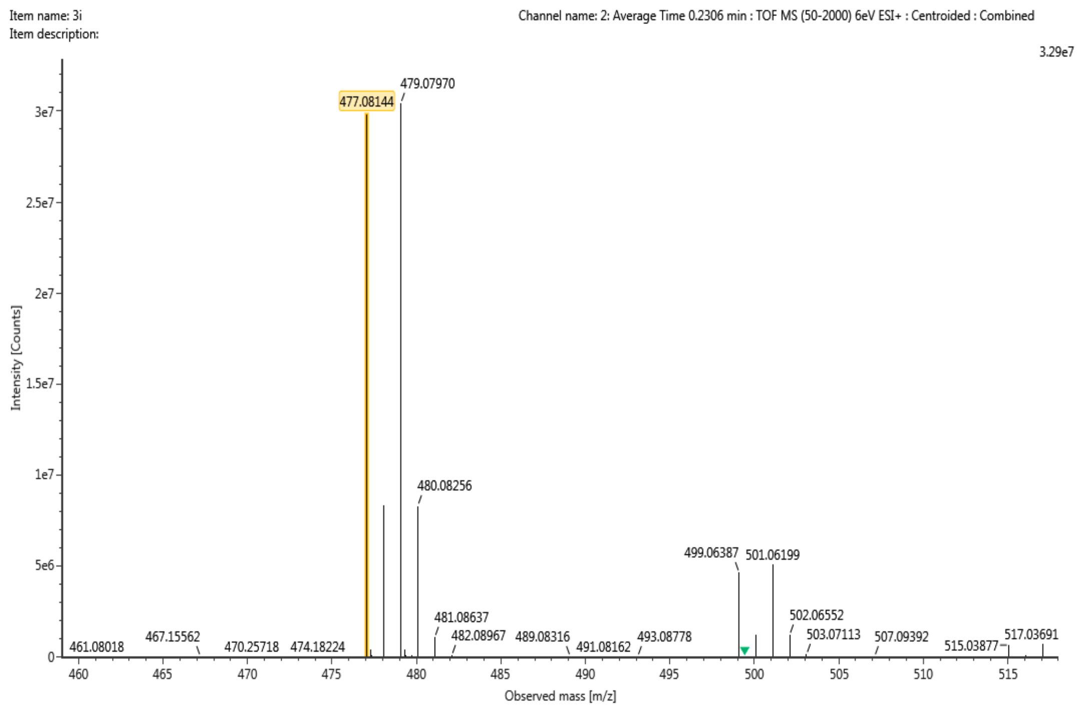

Fig. S79 The Mass spectrogram of **3i**

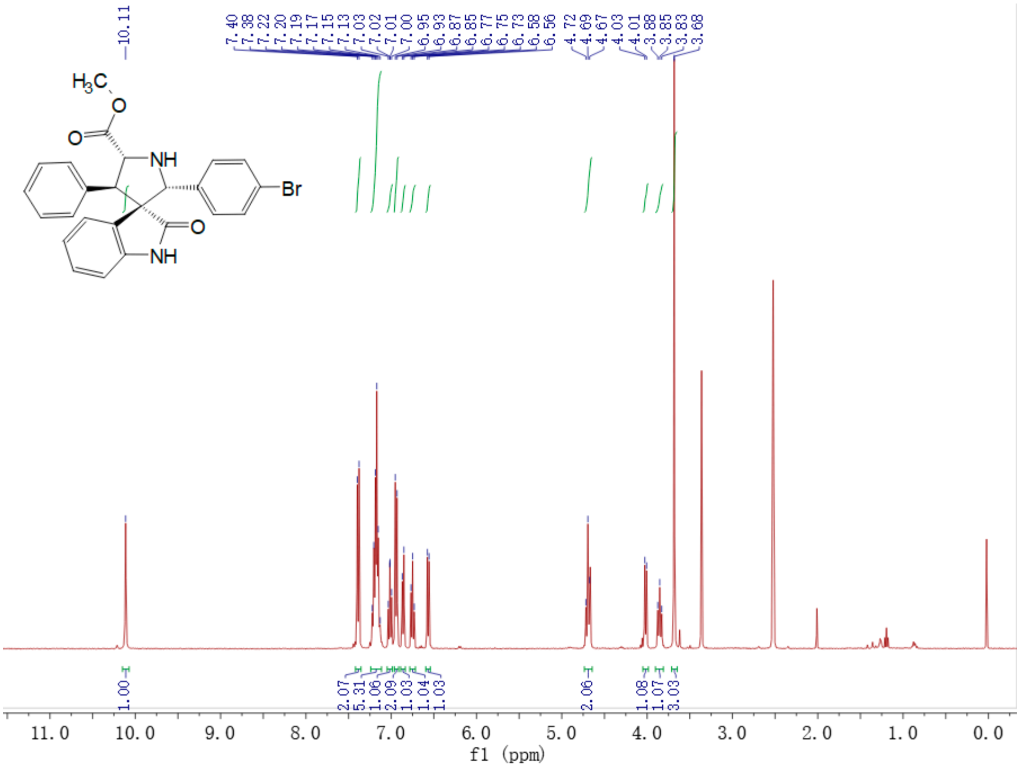

Fig. S80 The ^{1}H NMR (400 MHz, DMSO-d6) of **3j**

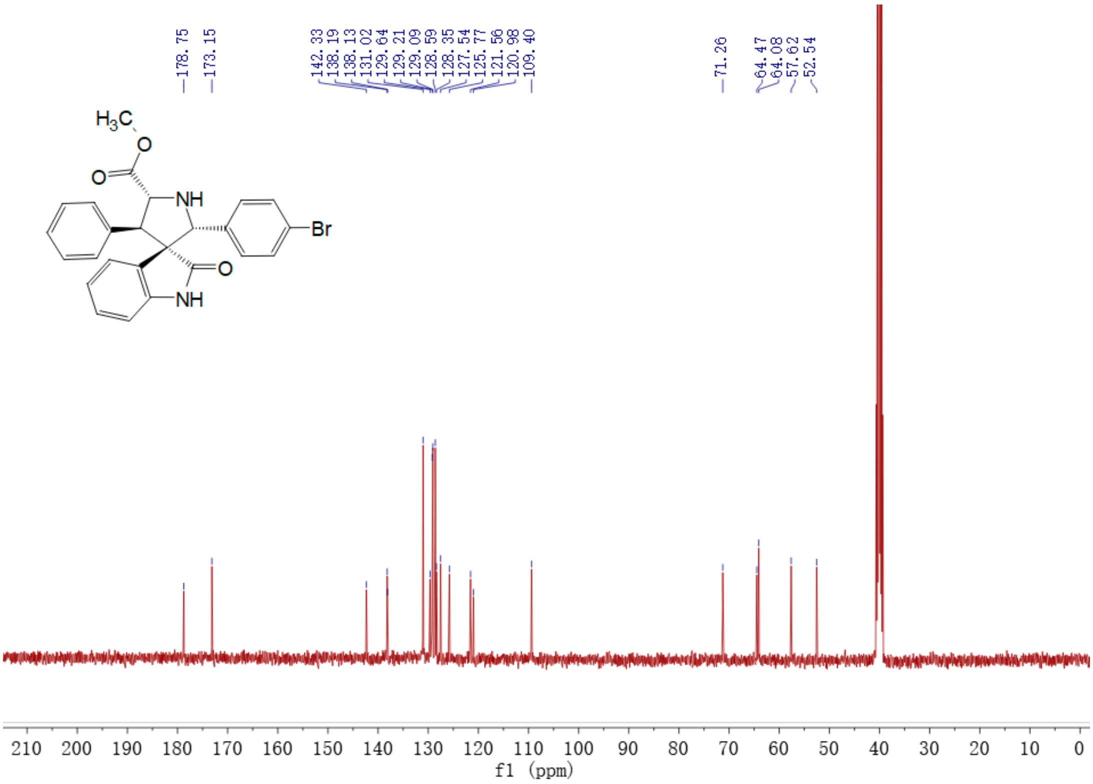

Fig. S81 The ^{13}C NMR (101 MHz, DMSO-d6) of **3j**

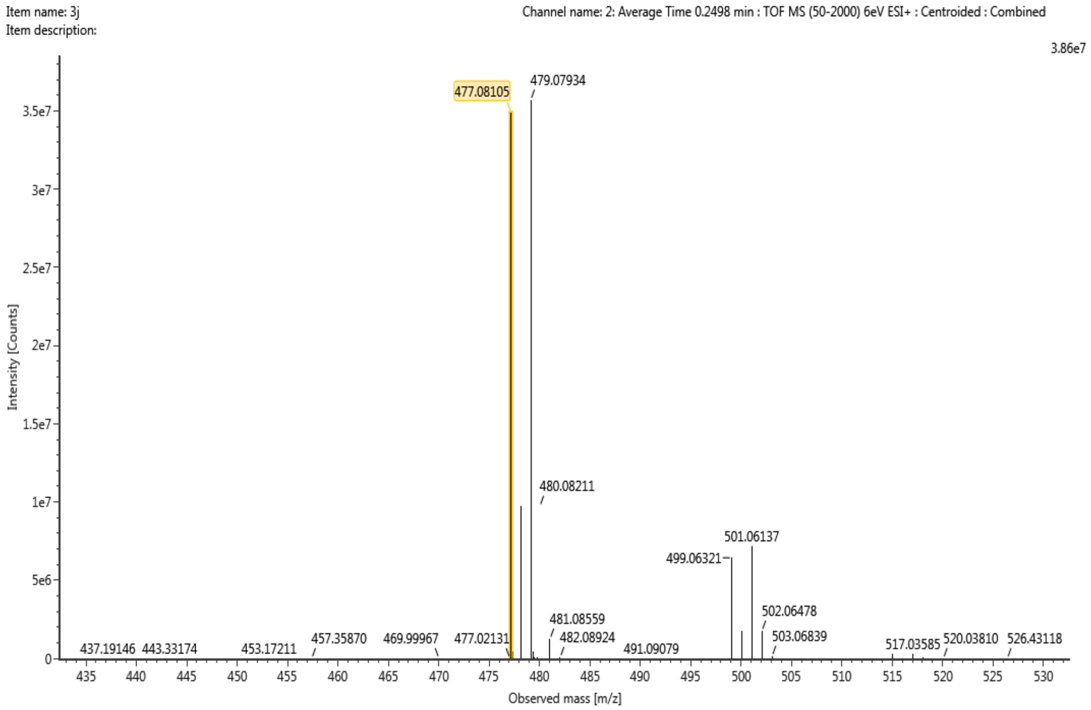

Fig. S82 The Mass spectrogram of **3j**

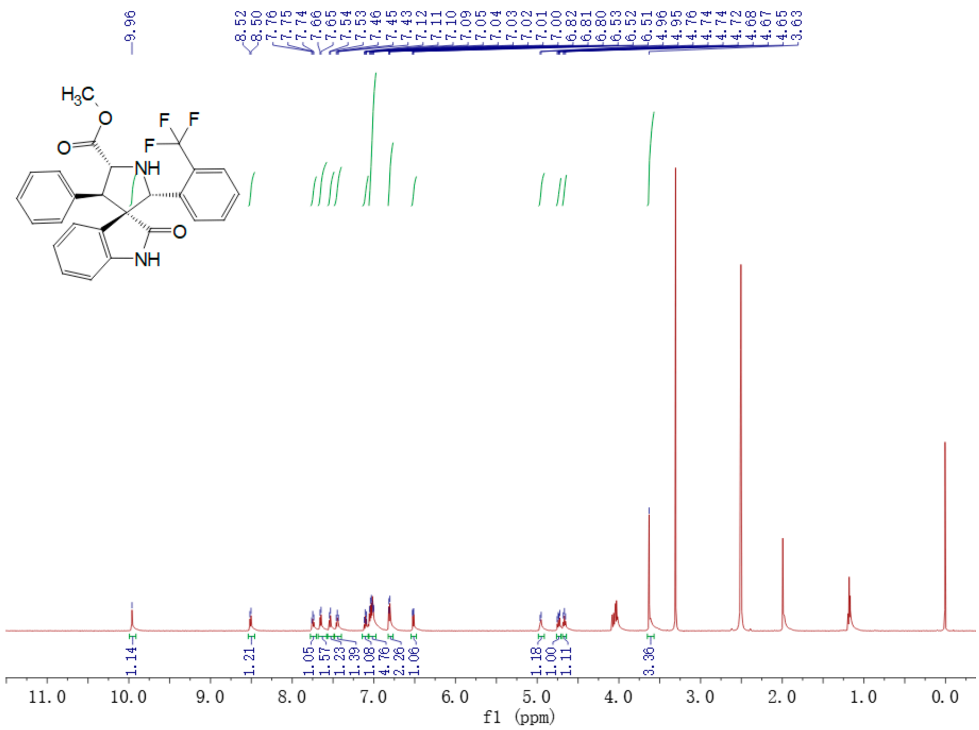

Fig. S83 The ^{1}H NMR (600 MHz, DMSO-d$_6$) of **3k**

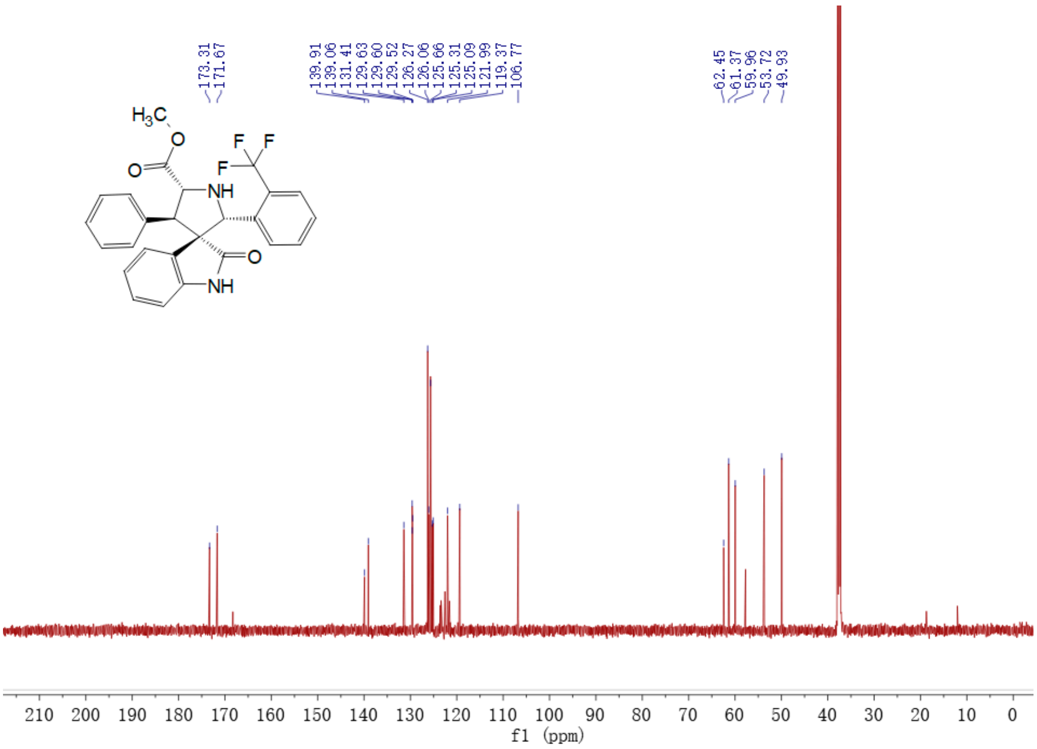

Fig. S84 The ^{13}C NMR (151 MHz, DMSO-d$_6$) of **3k**

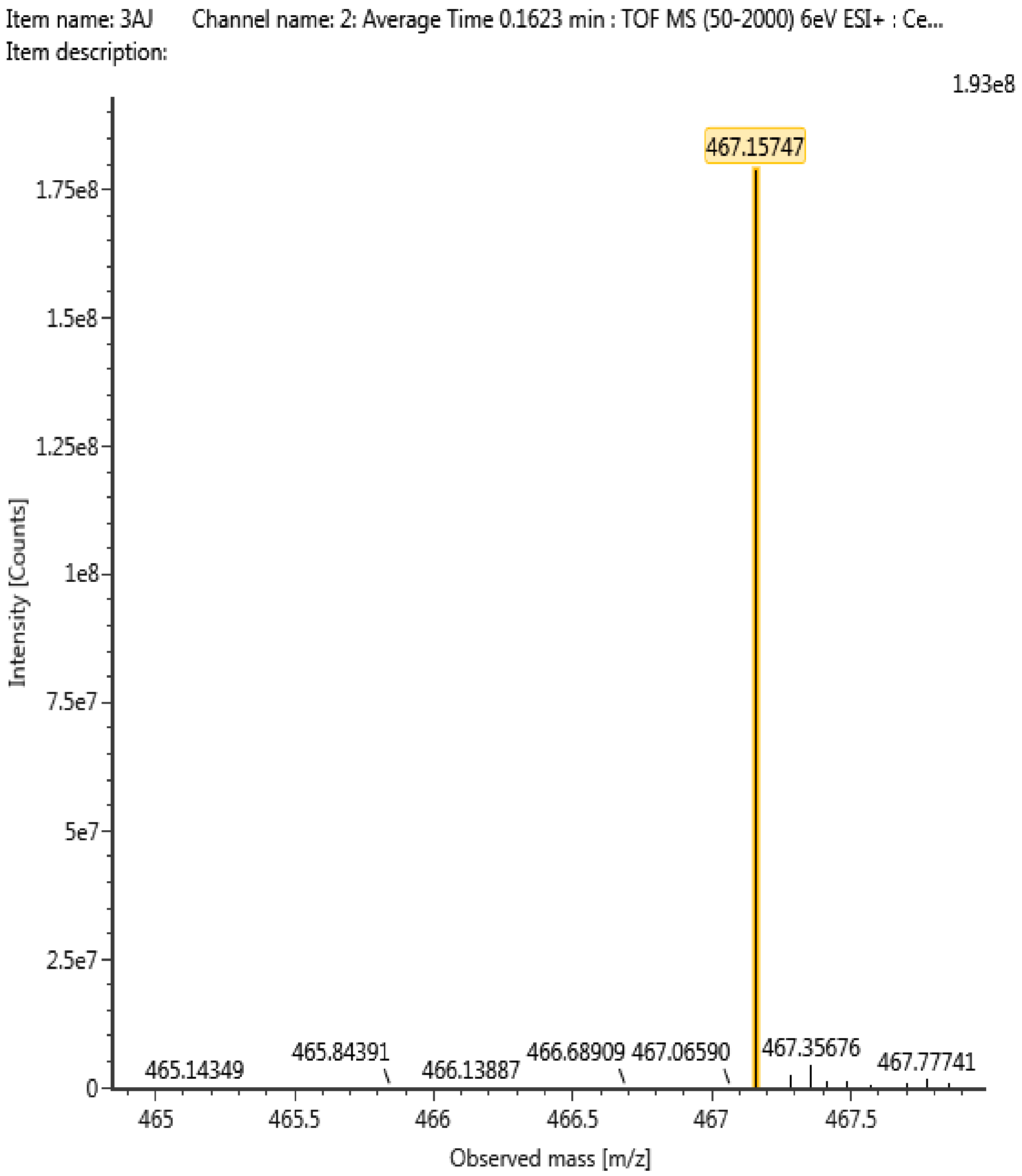

Fig. S85 The Mass spectrogram of **3k**

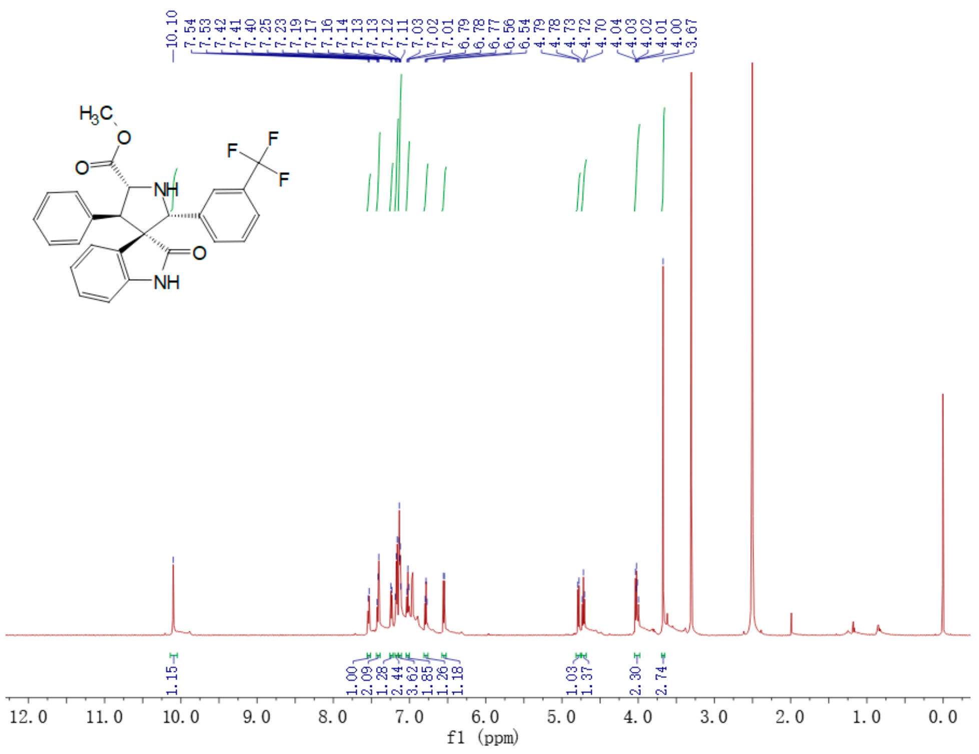

Fig. S86 The ^{1}H NMR (600 MHz, DMSO-d6) of **3l**

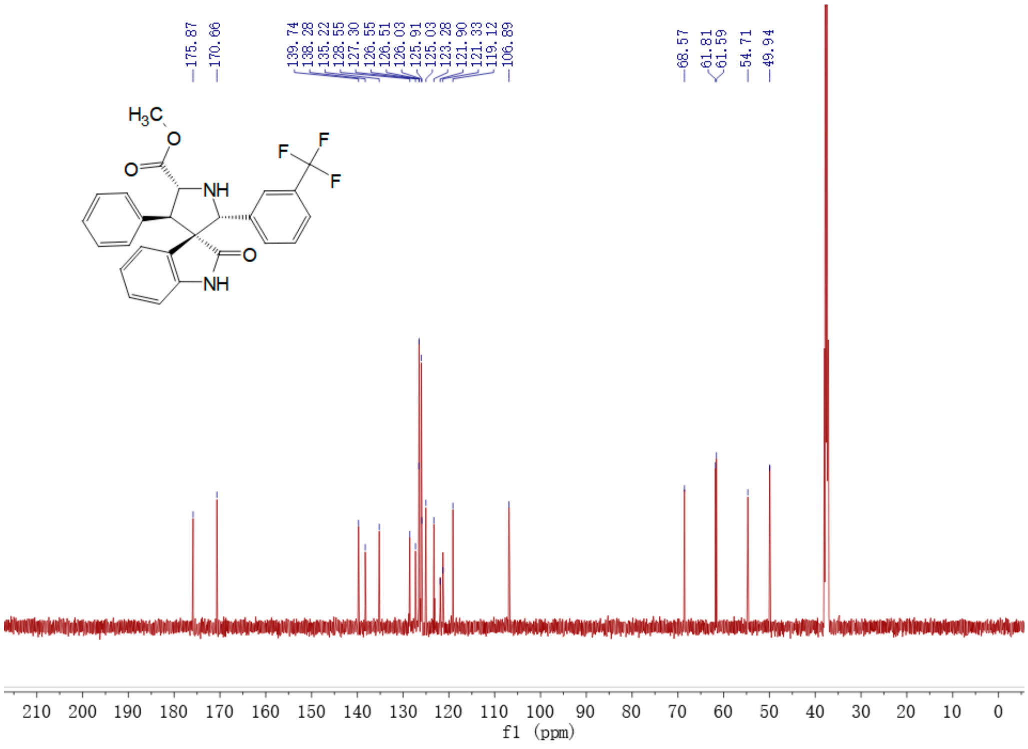

Fig. S87 The ^{13}C NMR (151 MHz, DMSO-d6) of **3l**

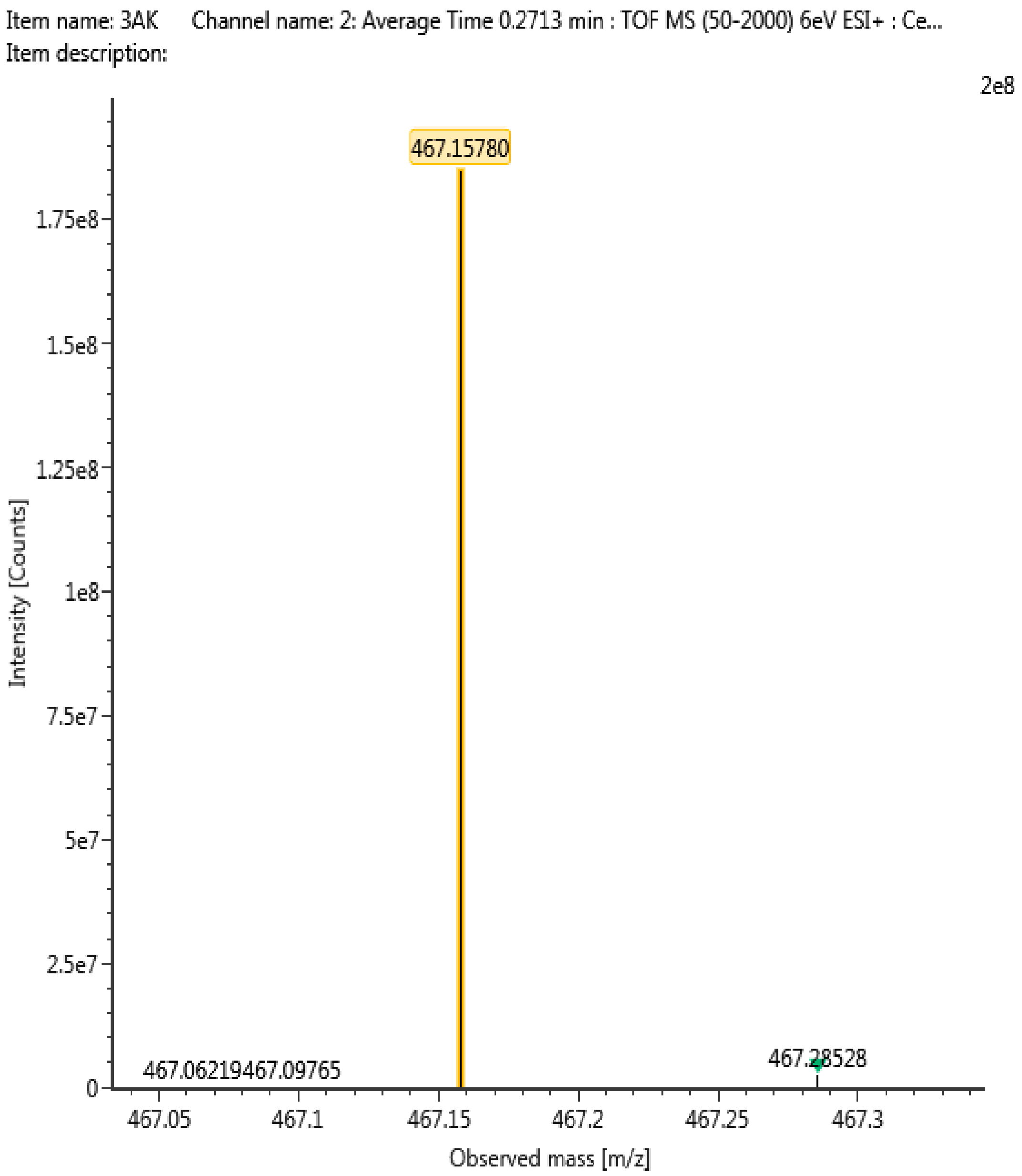

Fig. S88 The Mass spectrogram of **3l**

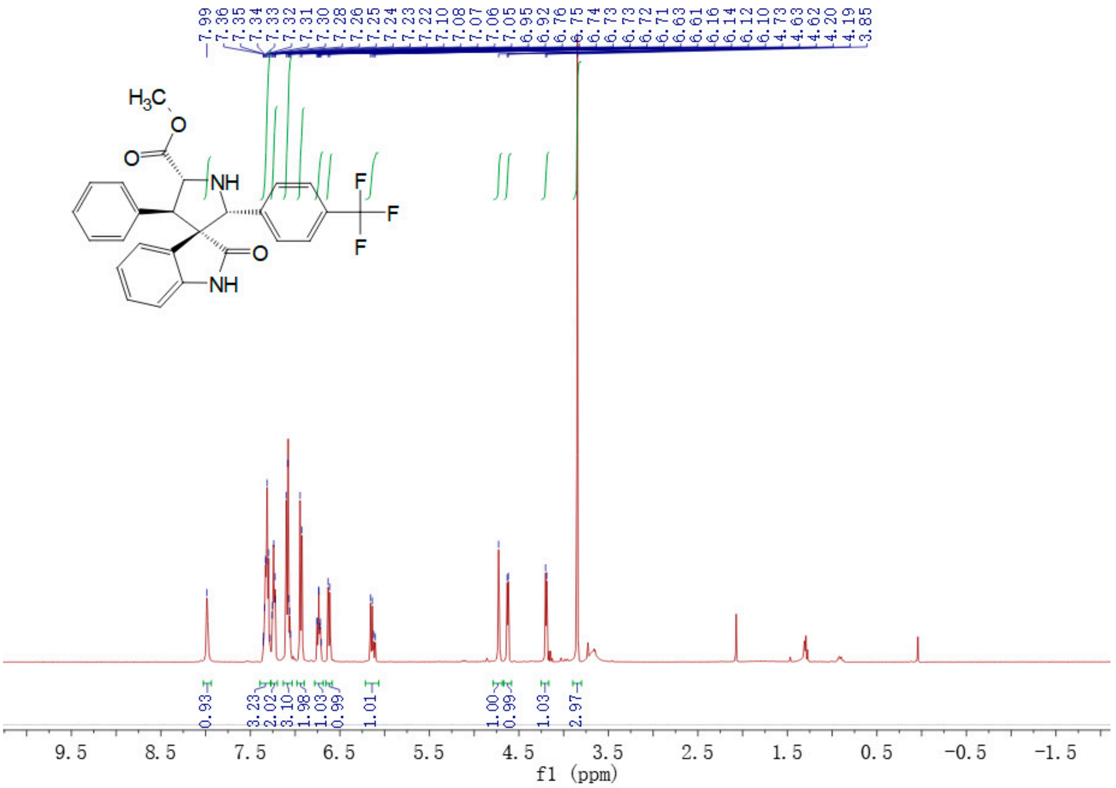

Fig. S89 The ^{1}H NMR (400 MHz, $CDCl_3$) of **3m**

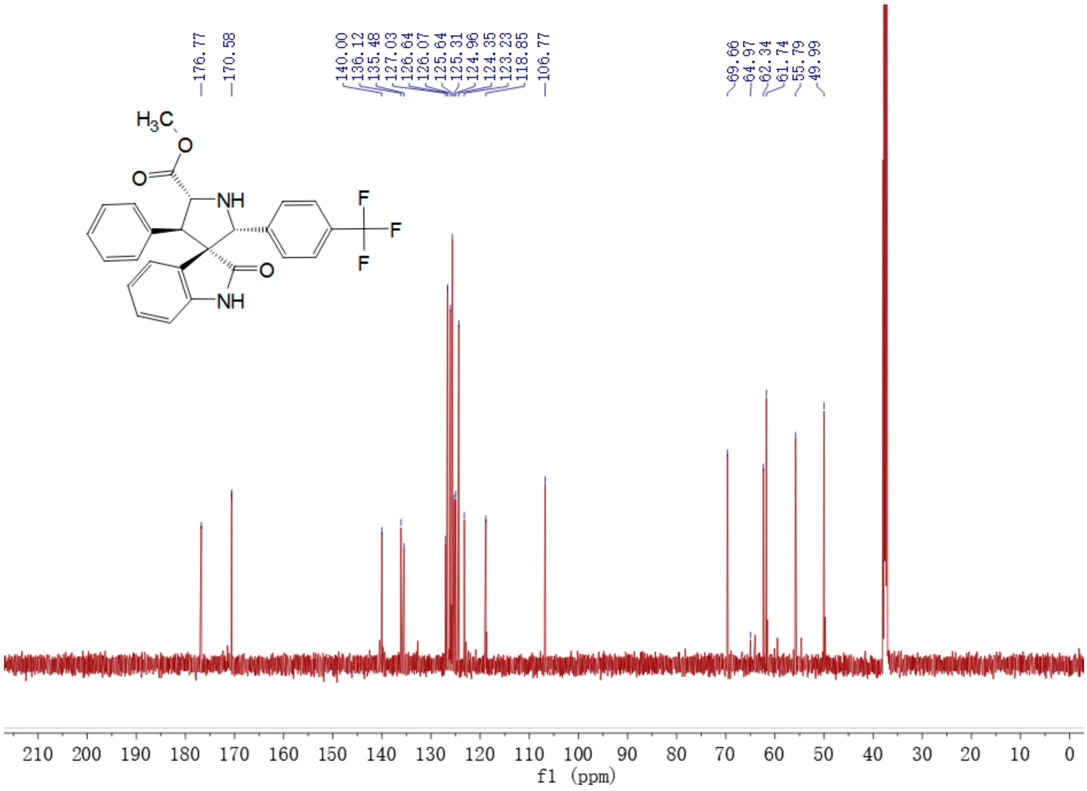

Fig. S90 The ^{13}C NMR (151 MHz, DMSO-d_6) of **3m**

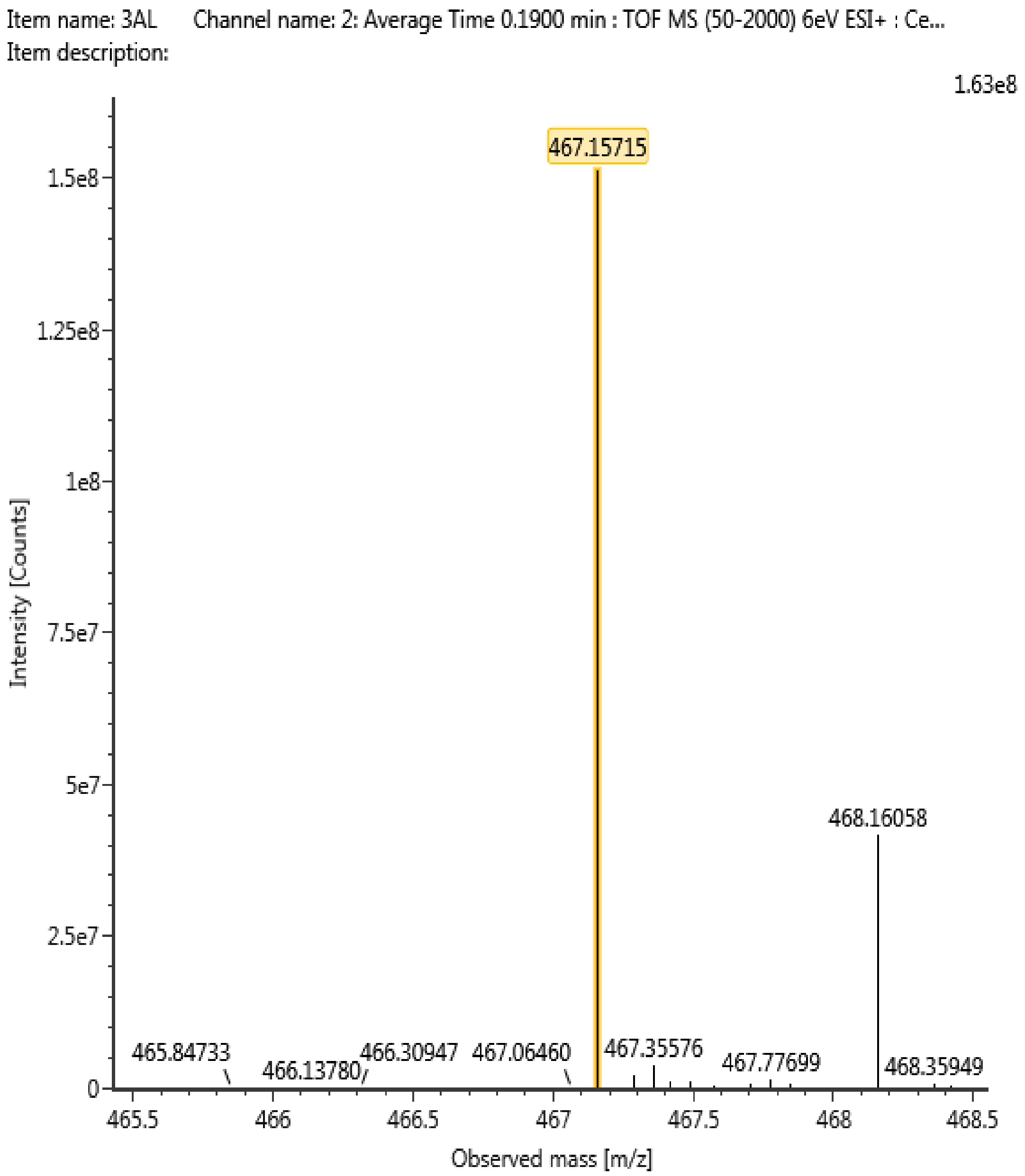

Fig. S91 The Mass spectrogram of **3m**

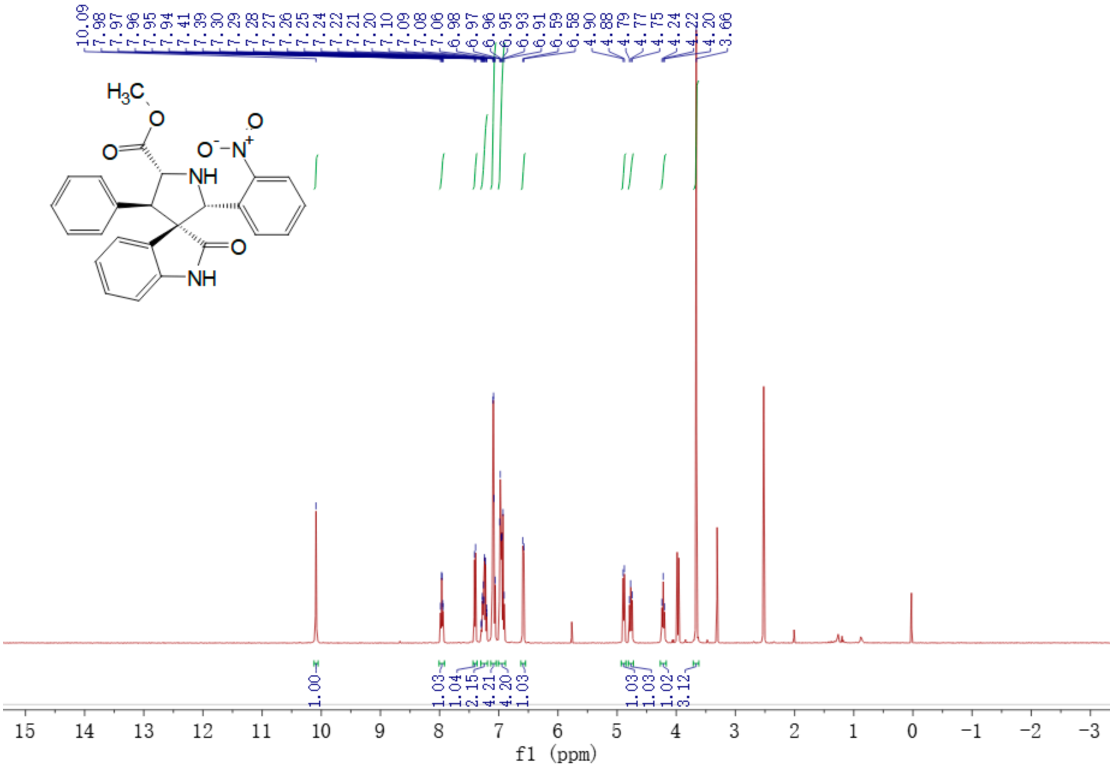

Fig. S92 The ^{1}H NMR (400 MHz, DMSO-d$_6$) of **3n**

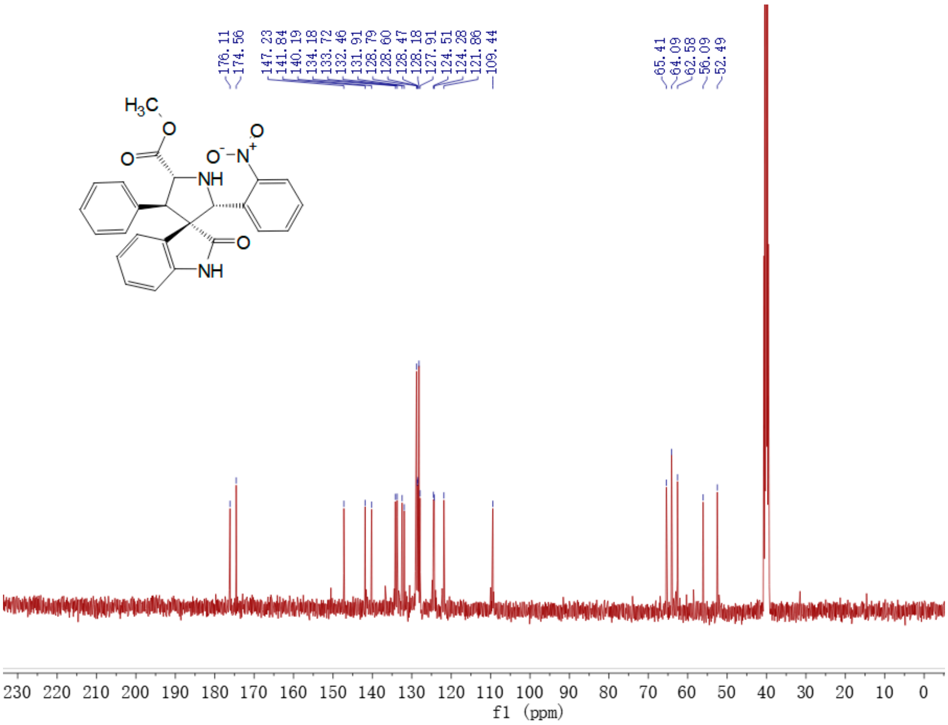

Fig. S93 The ^{13}C NMR (101 MHz, DMSO-d$_6$) of **3n**

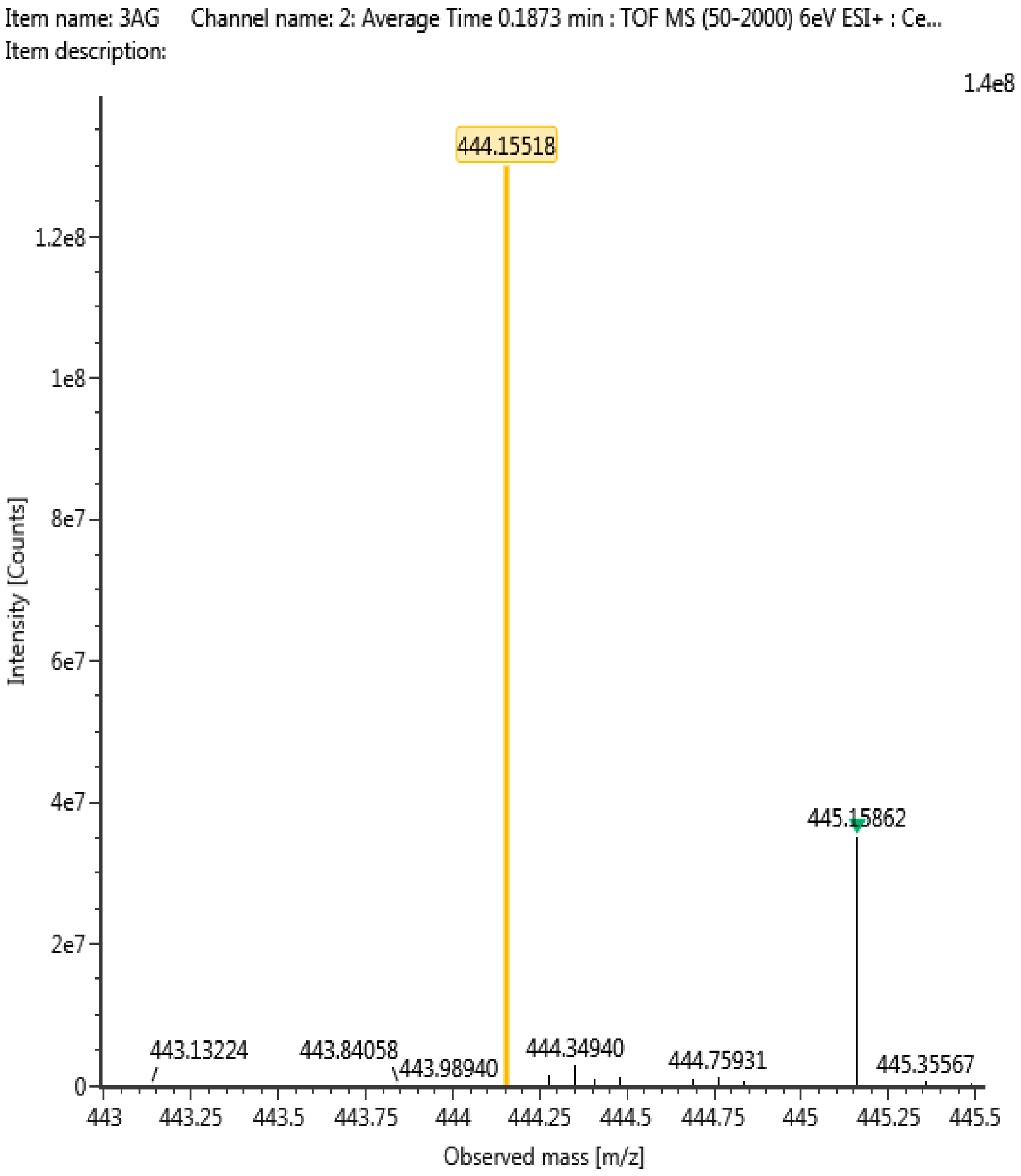

Fig. S94 The Mass spectrogram of **3n**

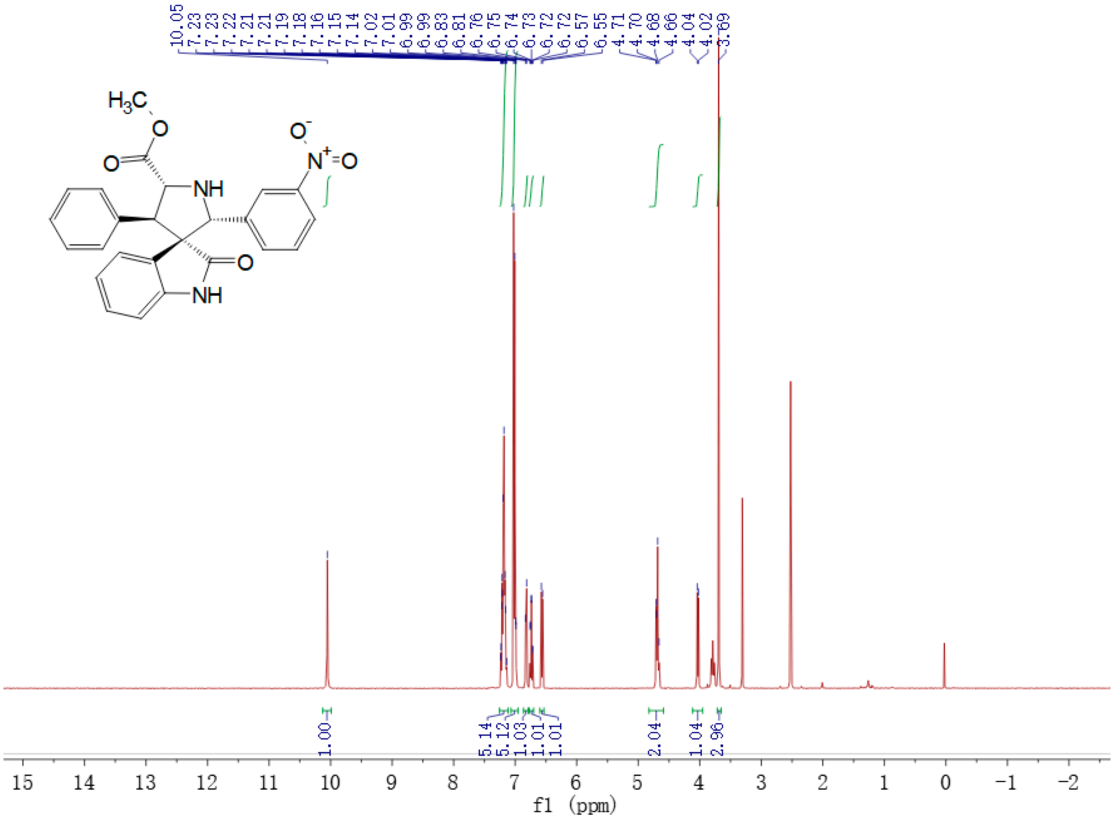

Fig. S95 The ^{1}H NMR (400 MHz, DMSO-d$_6$) of **3o**

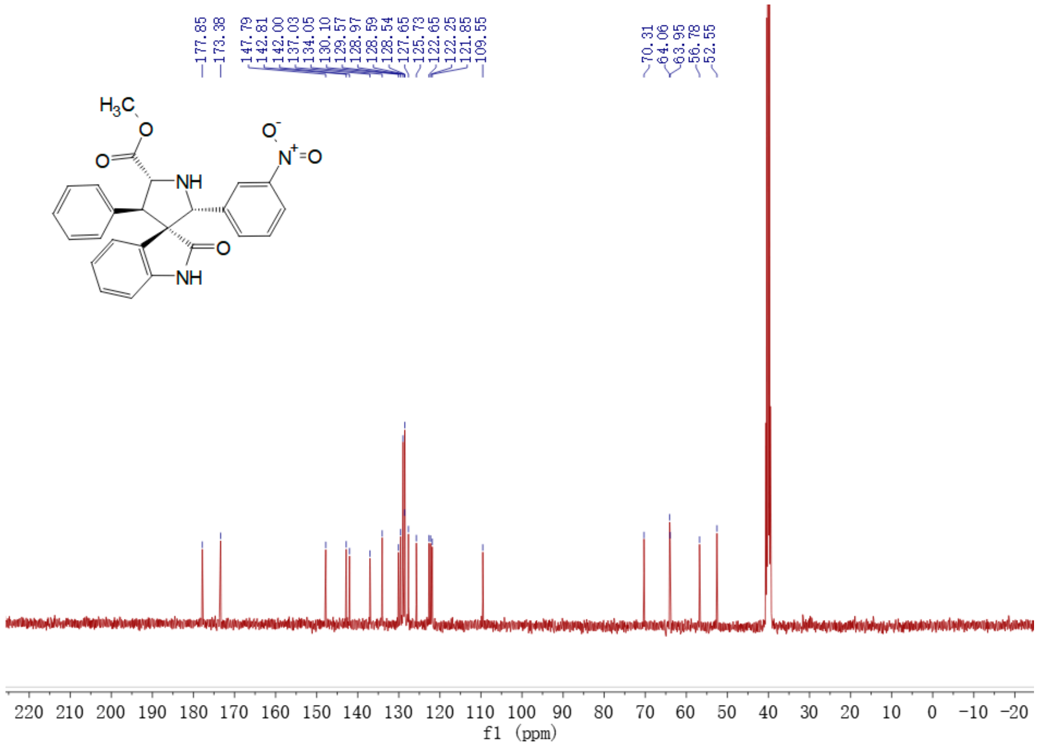

Fig. S96 The ^{13}C NMR (101 MHz, DMSO-d$_6$) of **3o**

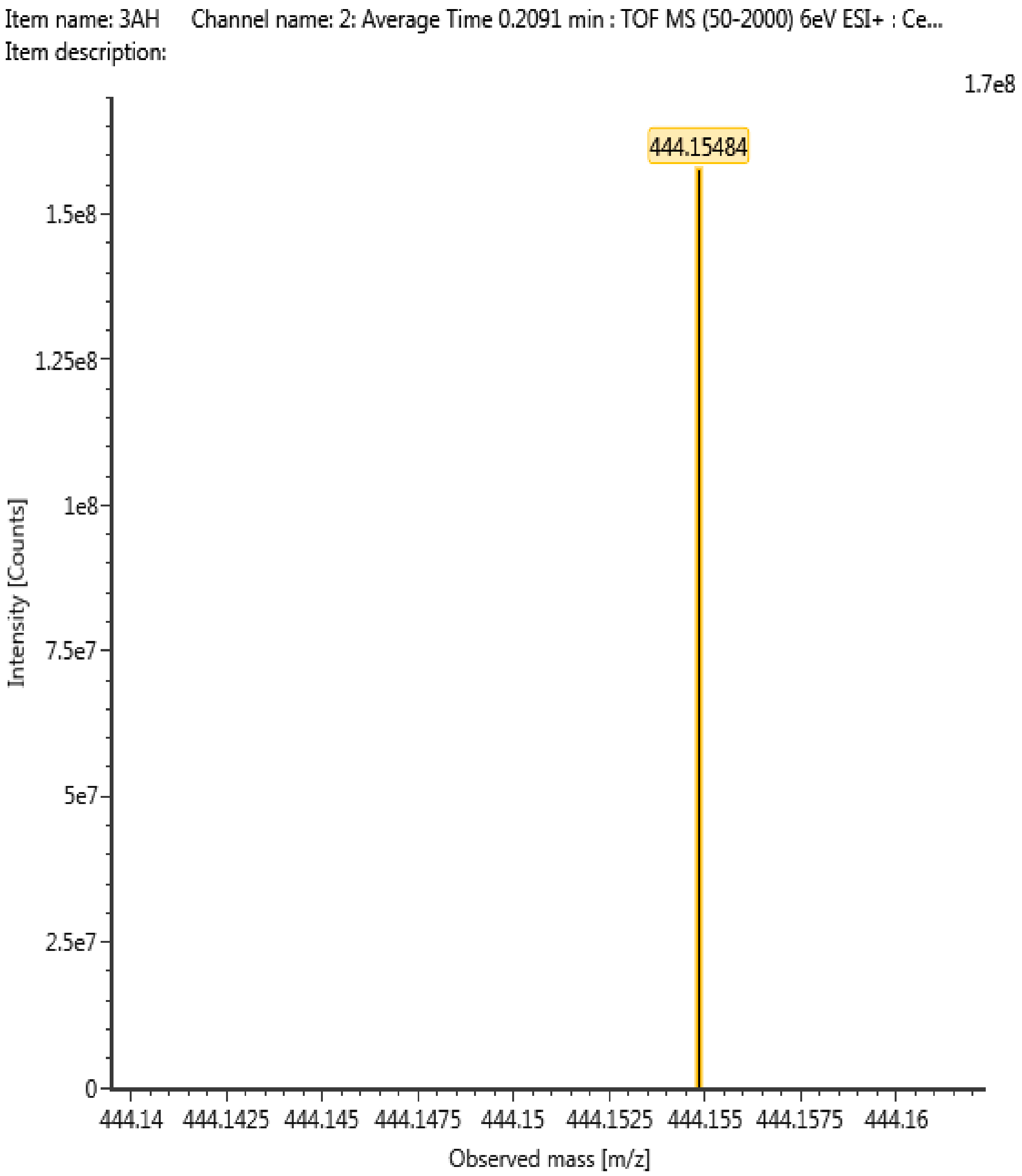

Fig. S97 The Mass spectrogram of **3o**

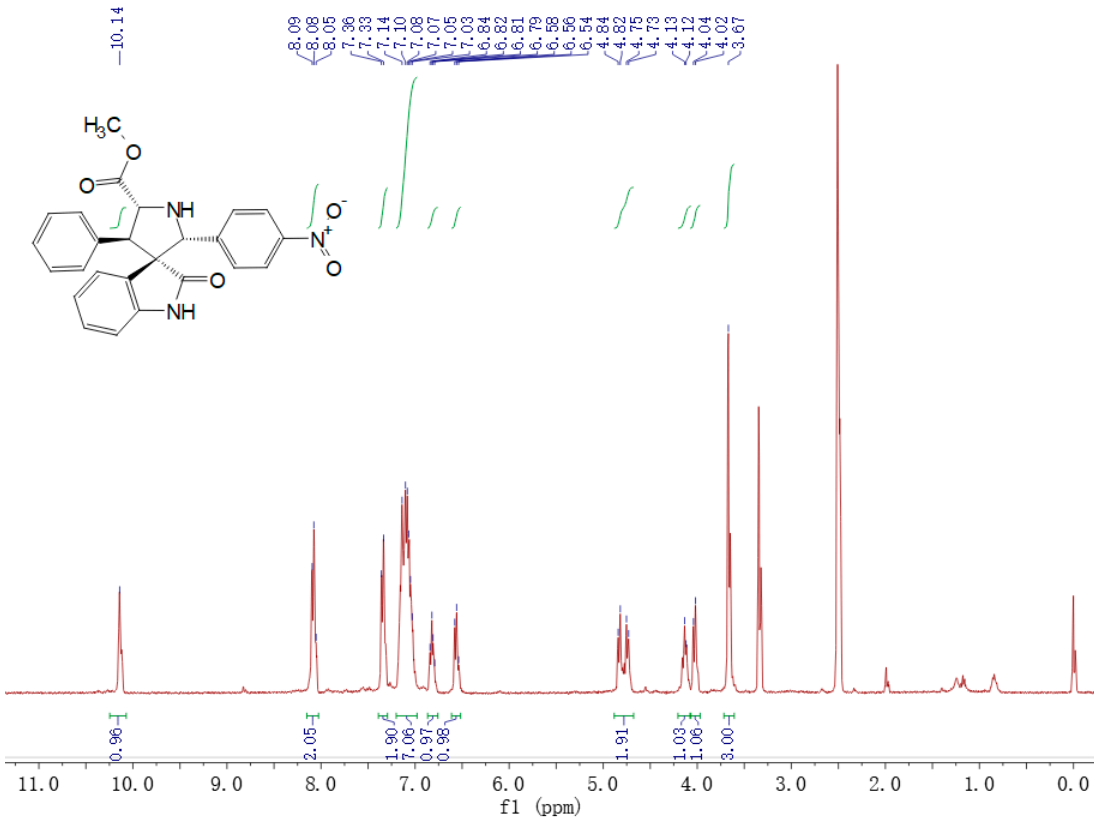

Fig. S98 The ^{1}H NMR (400 MHz, DMSO-d$_6$) of **3p**

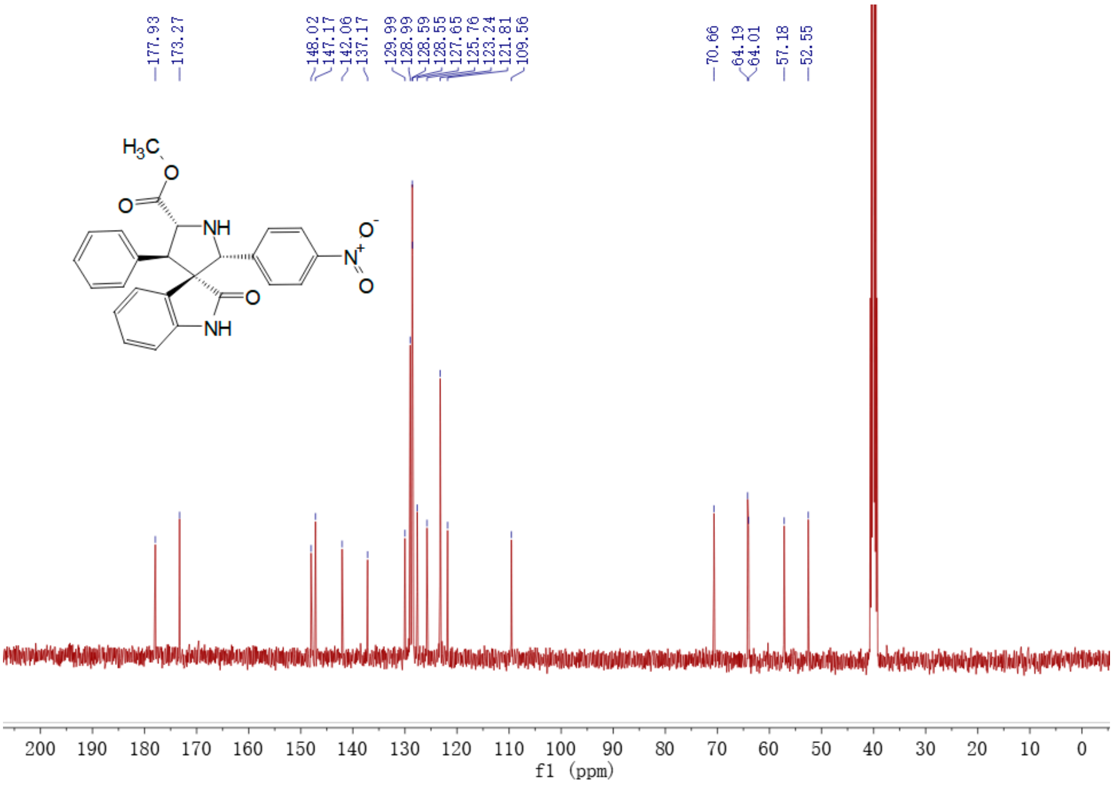

Fig. S99 The ^{13}C NMR (101 MHz, DMSO-d$_6$) of **3p**

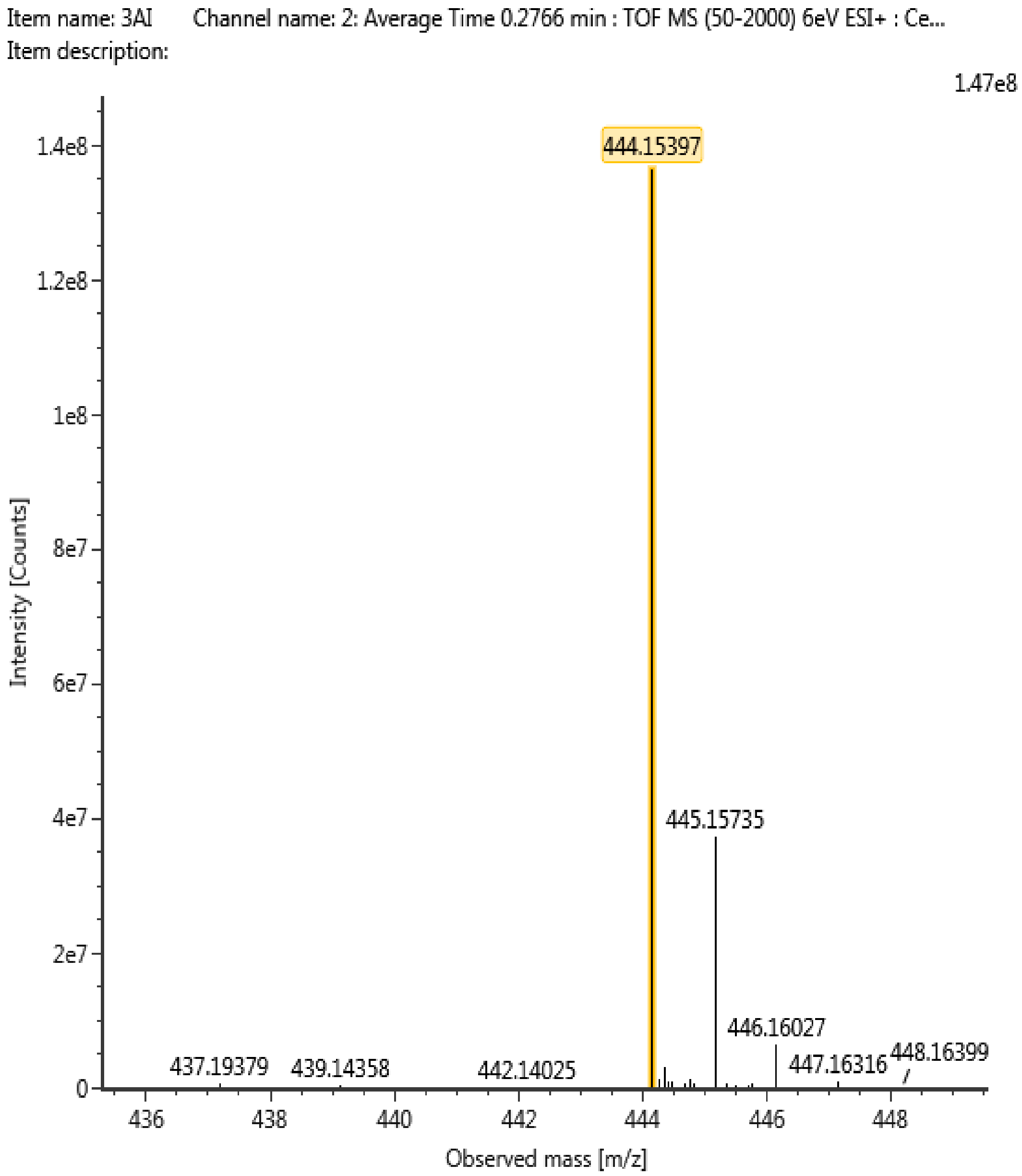

Fig. S100 The Mass spectrogram of **3p**

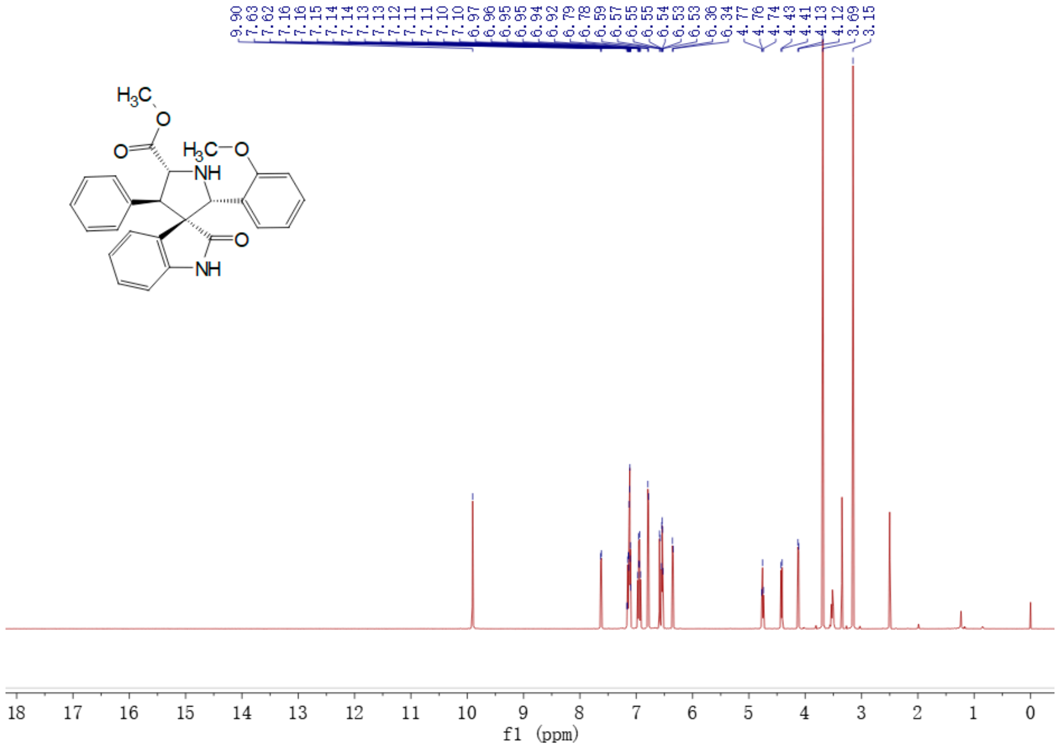

Fig. S101 The ^{1}H NMR (600 MHz, DMSO-d$_6$) of **3q**

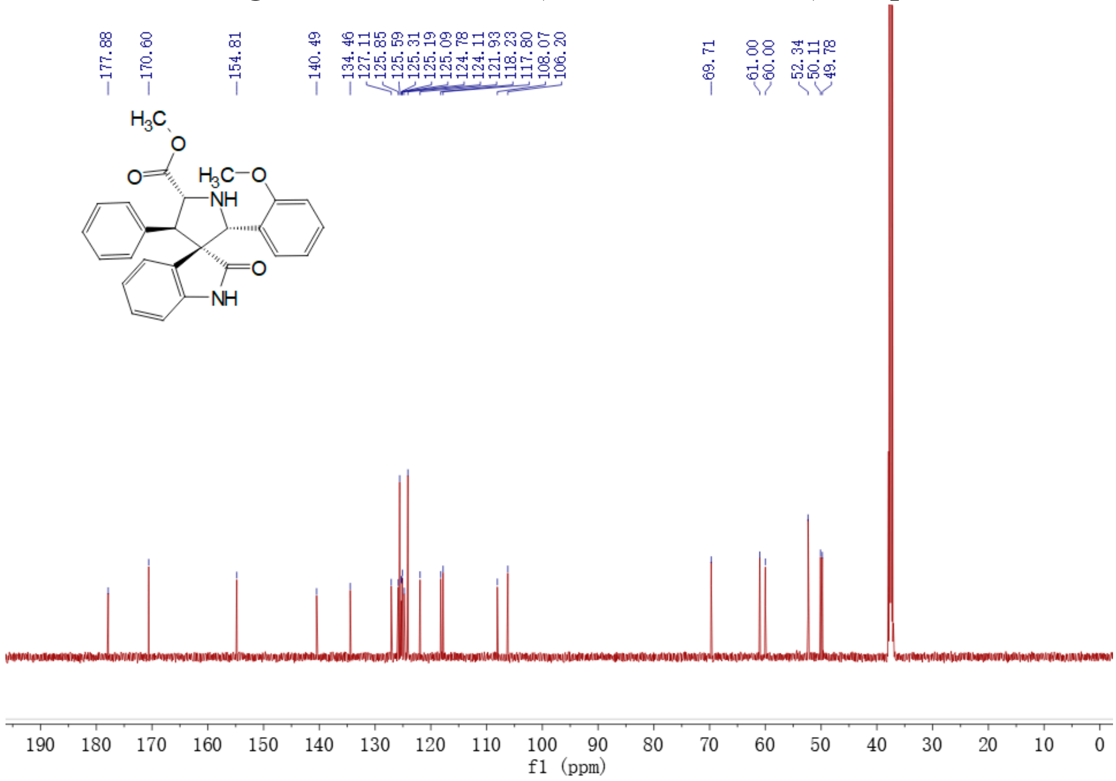

Fig. S102 The ^{13}C NMR (151 MHz, DMSO-d$_6$) of **3q**

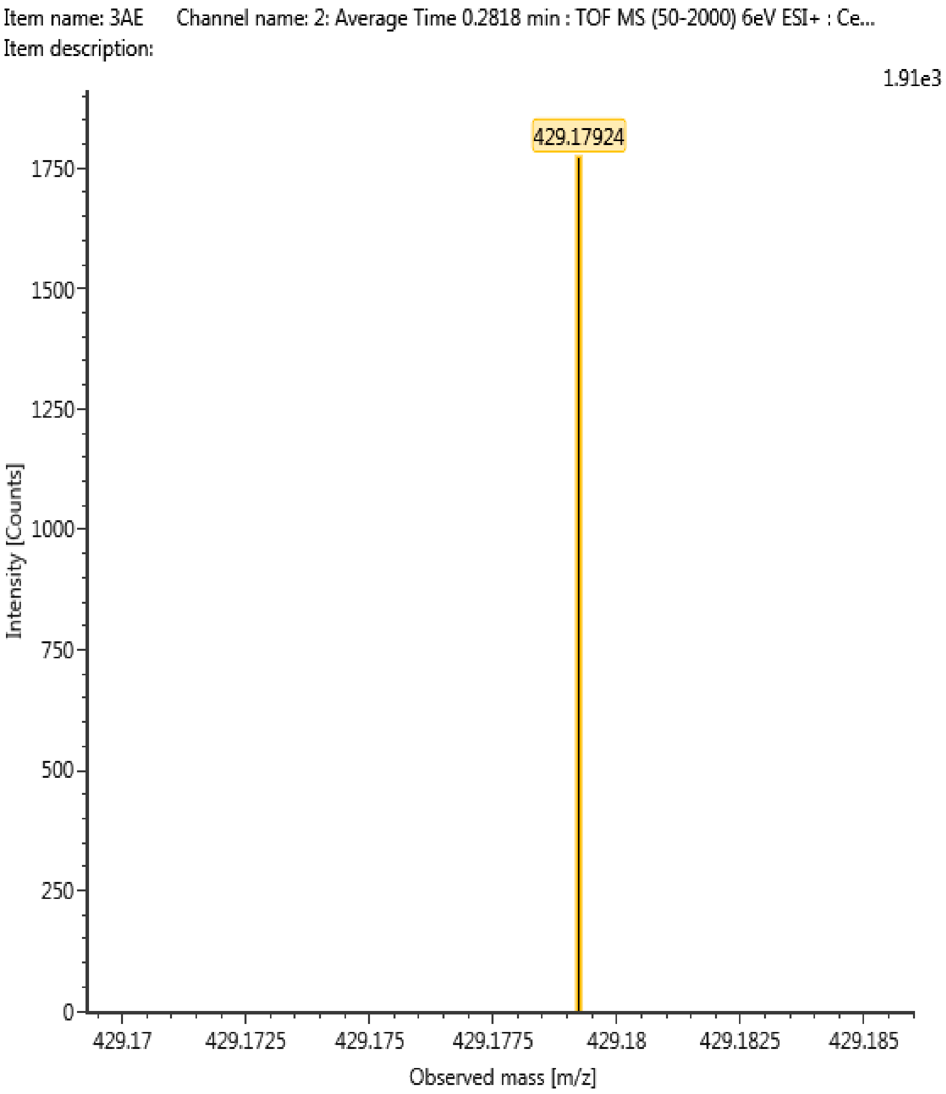

Fig. S103 The Mass spectrogram of **3q**

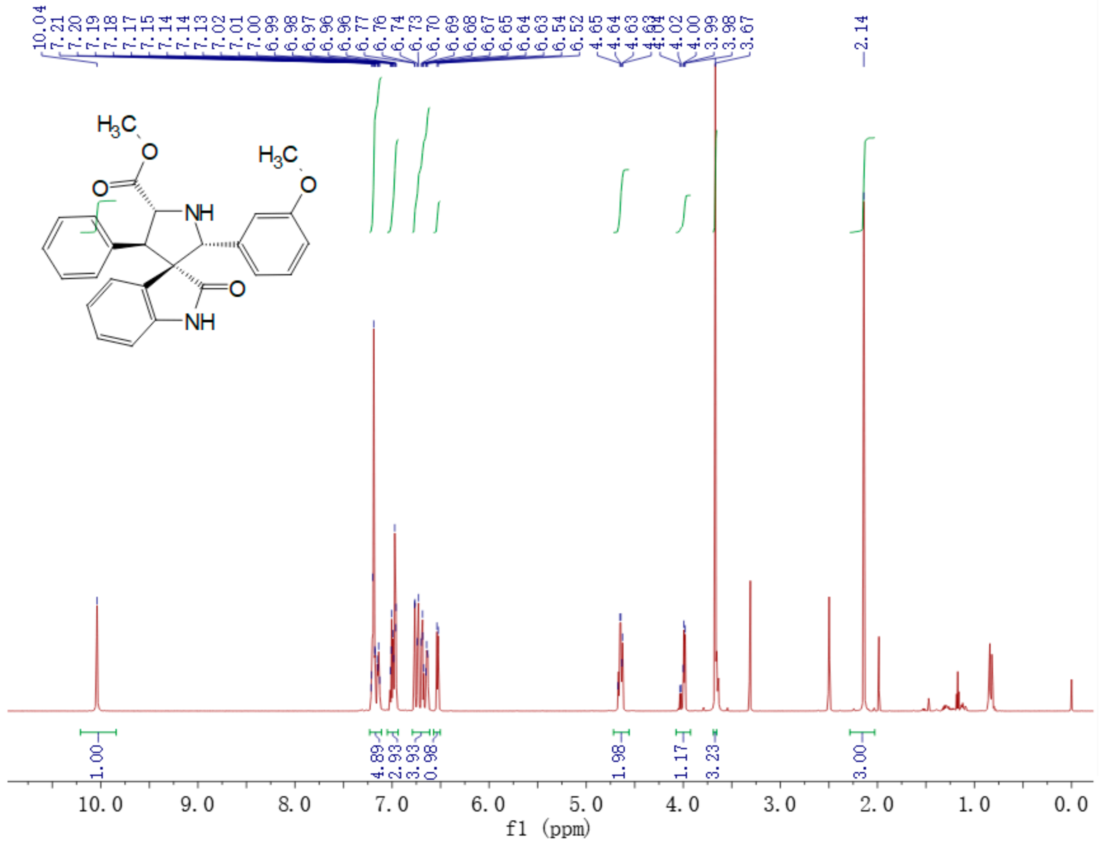

Fig. S104 The ^{1}H NMR (600 MHz, DMSO-d$_6$) of **3r**

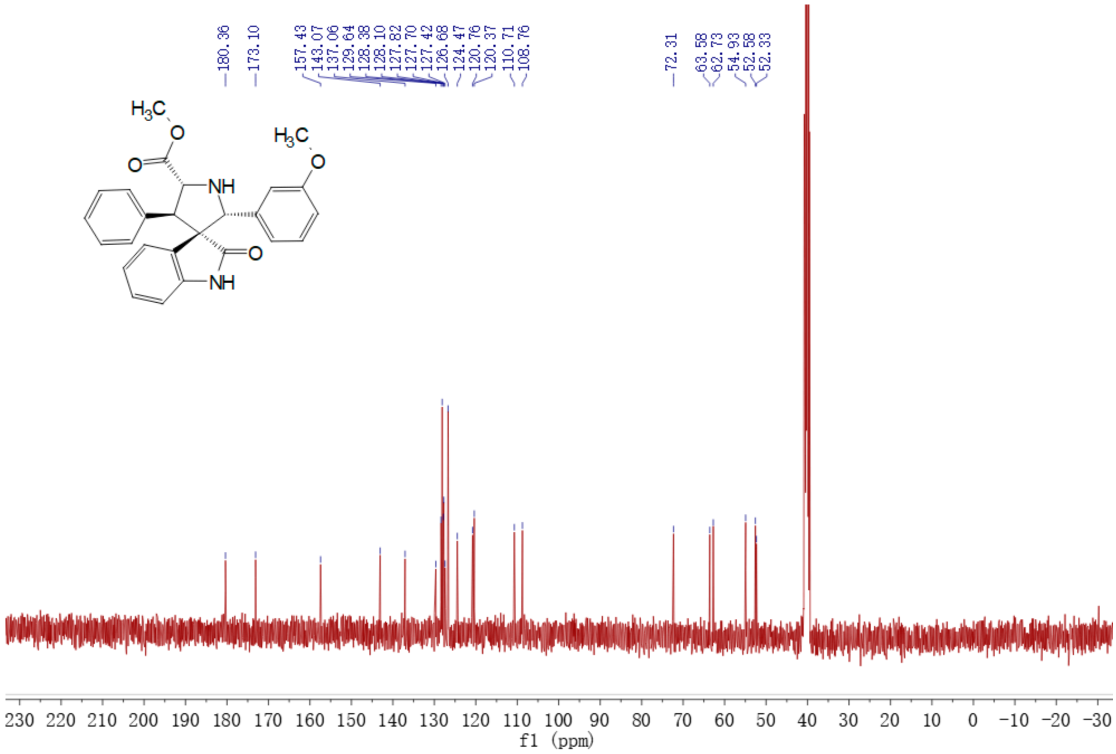

Fig. S105 The ^{13}C NMR (101 MHz, DMSO-d$_6$) of **3r**

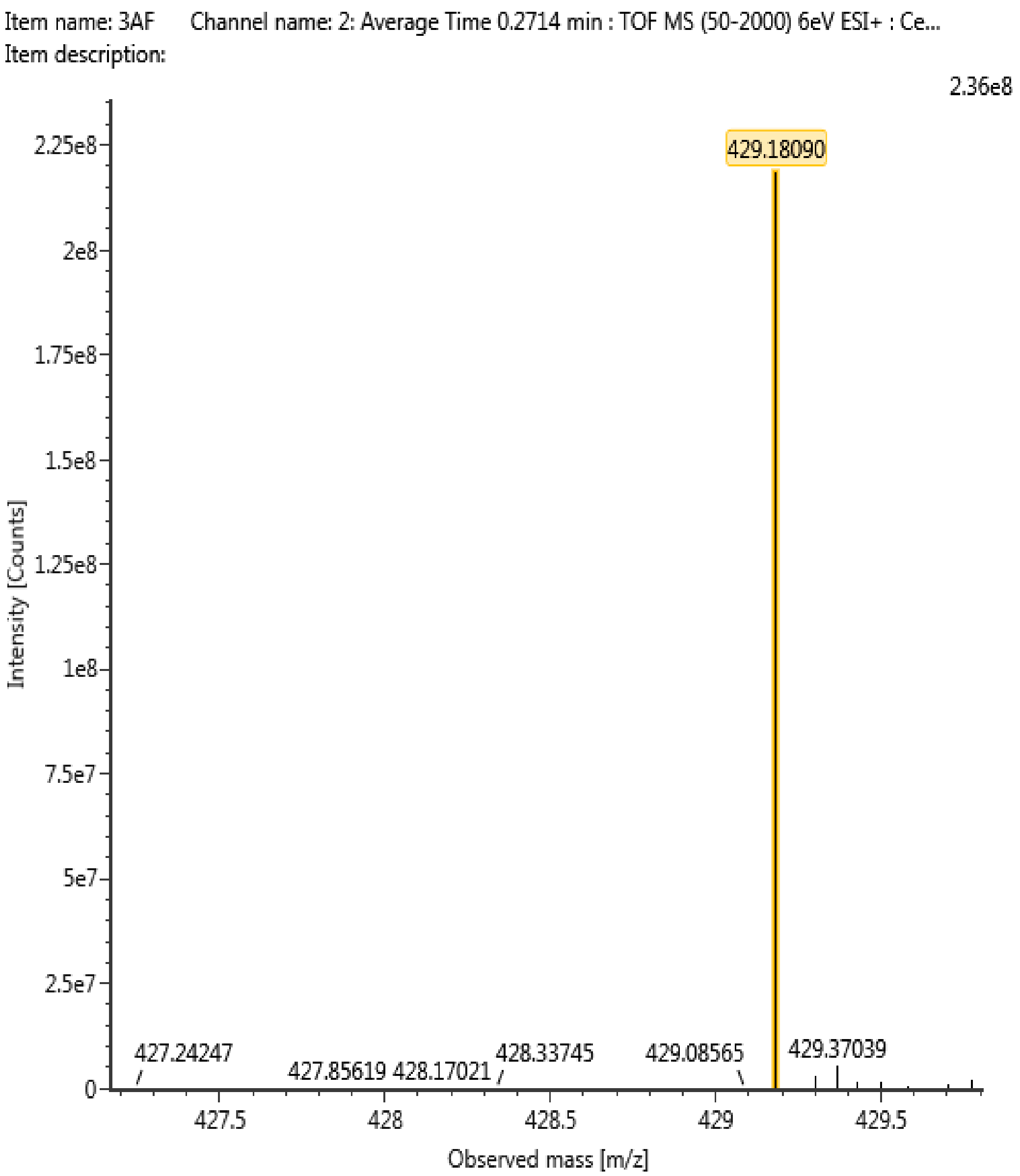

Fig. S106 The Mass spectrogram of **3r**

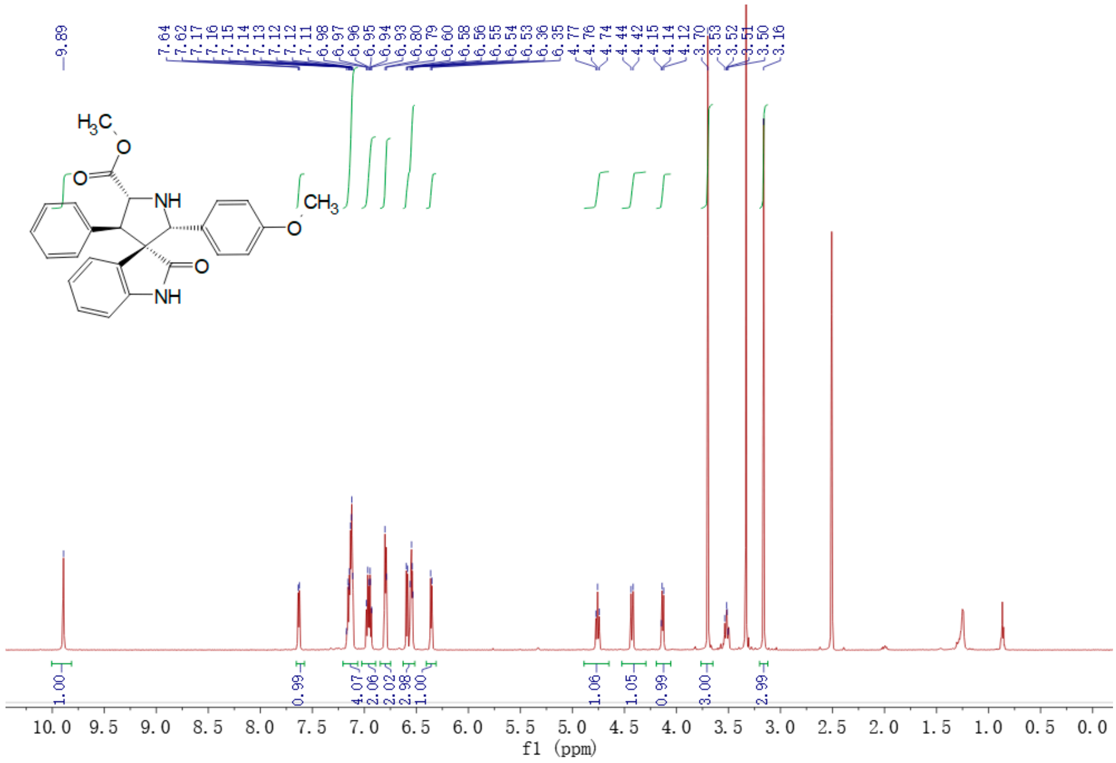

Fig. S107 The ^{1}H NMR (600 MHz, DMSO-d6) of **3s**

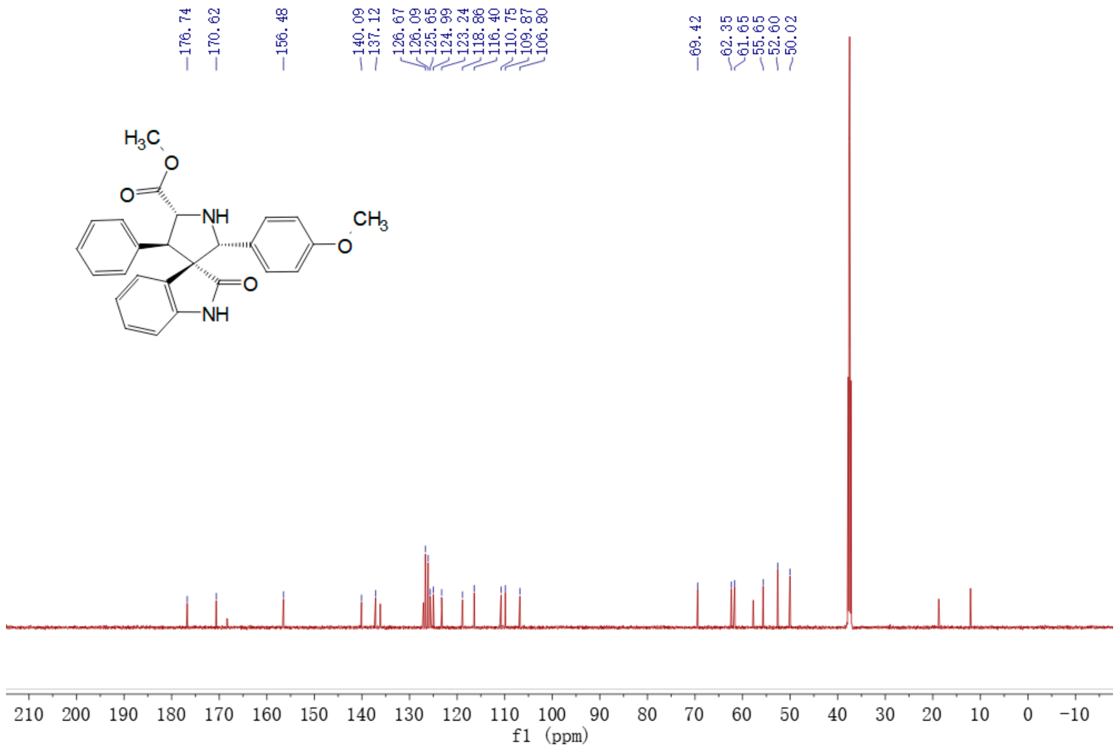

Fig. S108 The ^{13}C NMR (151 MHz, DMSO-d6) of **3s**

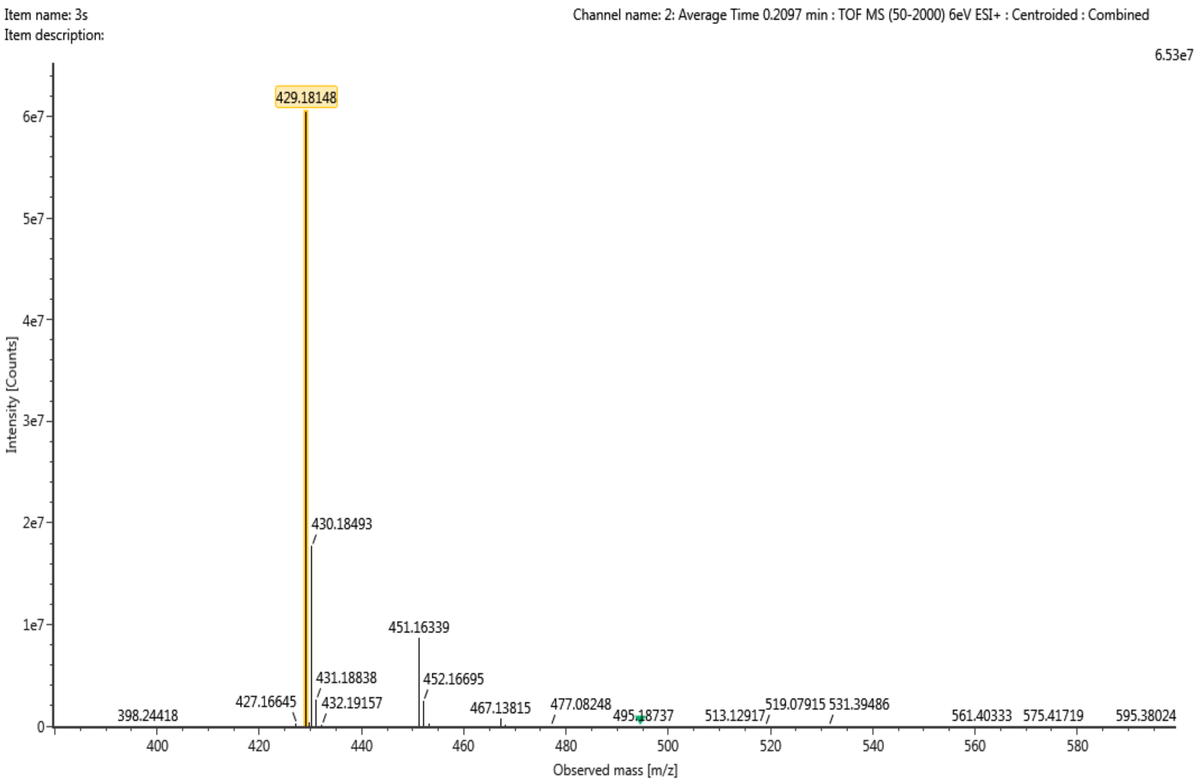

Fig. S109 The Mass spectrogram of **3s**

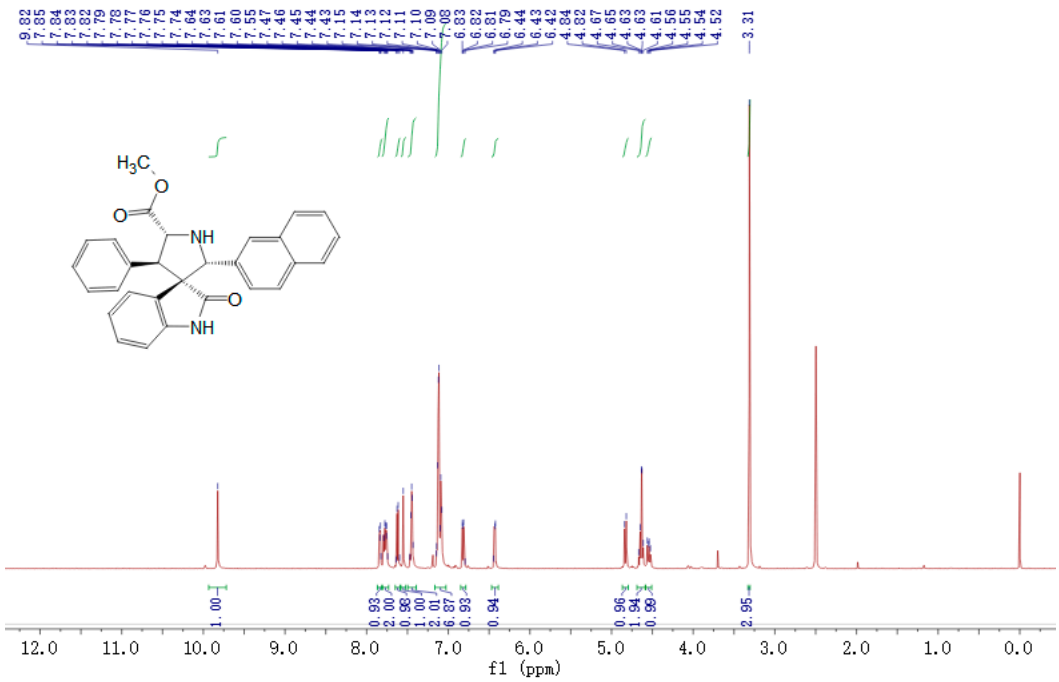

Fig. S110 The ^{1}H NMR (600 MHz, DMSO-d$_6$) of **3t**

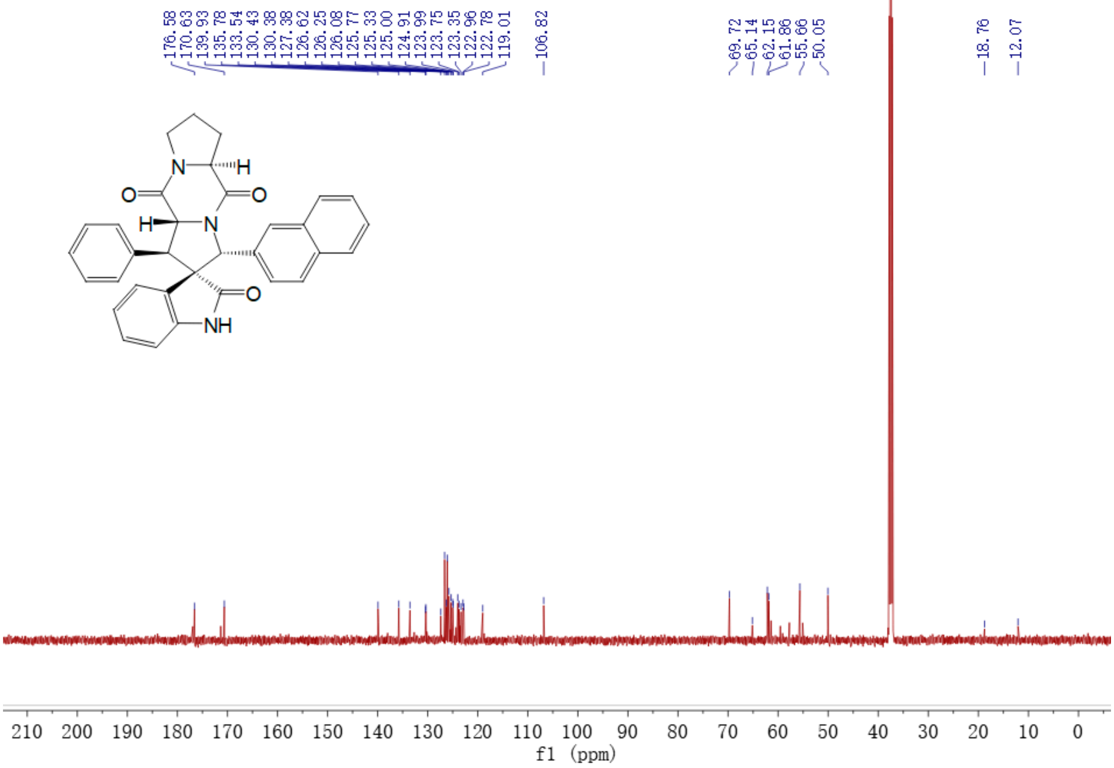

Fig. S111 The ^{13}C NMR (151 MHz, DMSO-d$_6$) of **3t**

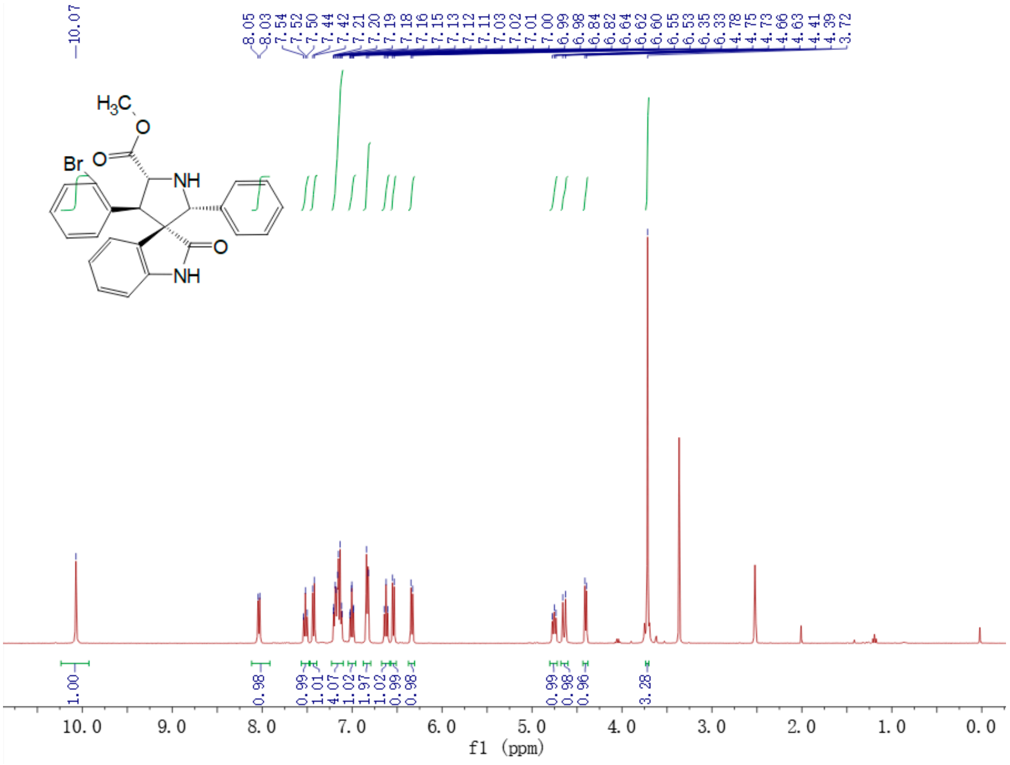

Fig. S112 The ^{1}H NMR (400 MHz, DMSO-d_6) of **3u**

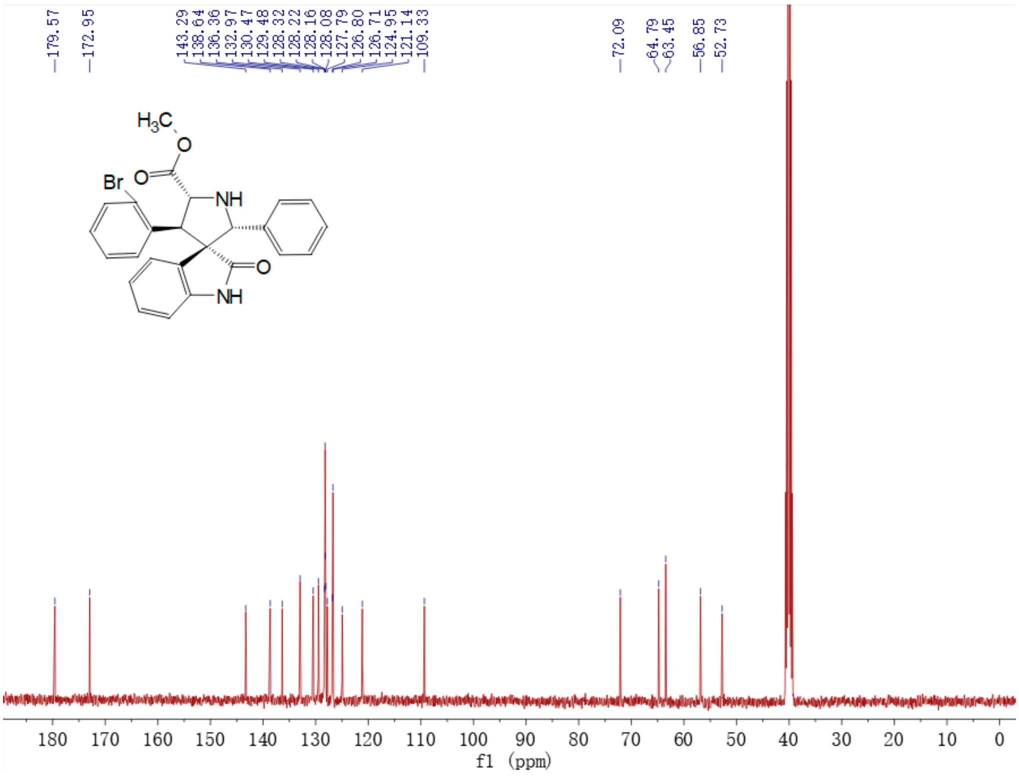

Fig. S113 The ^{13}C NMR (101 MHz, DMSO-d_6) of **3u**

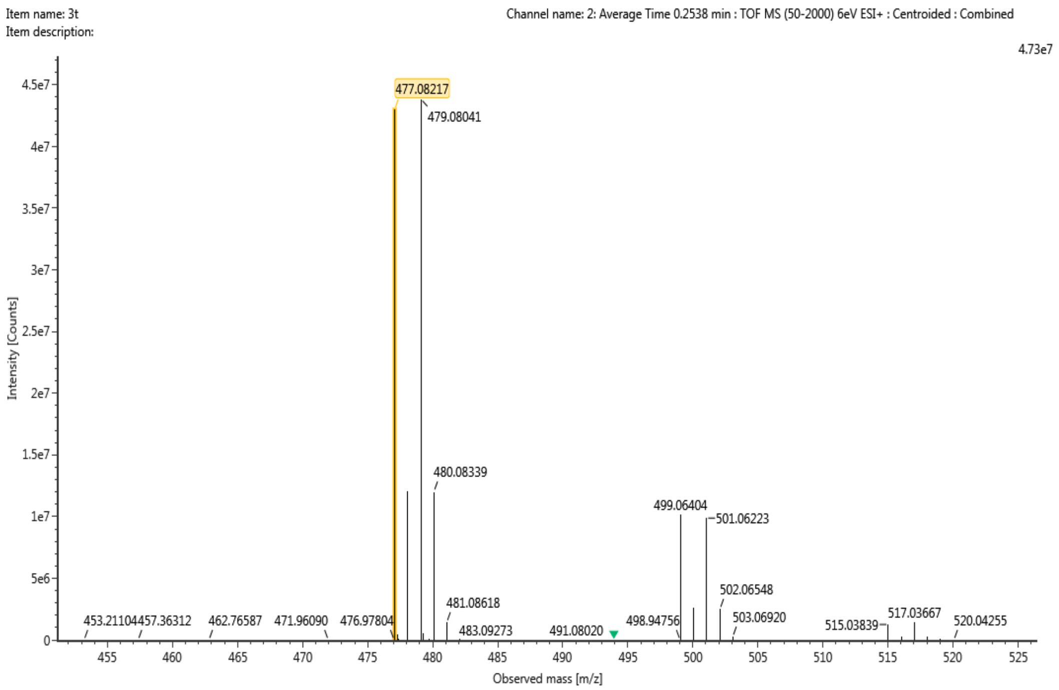

Fig. S114 The Mass spectrogram of **3u**

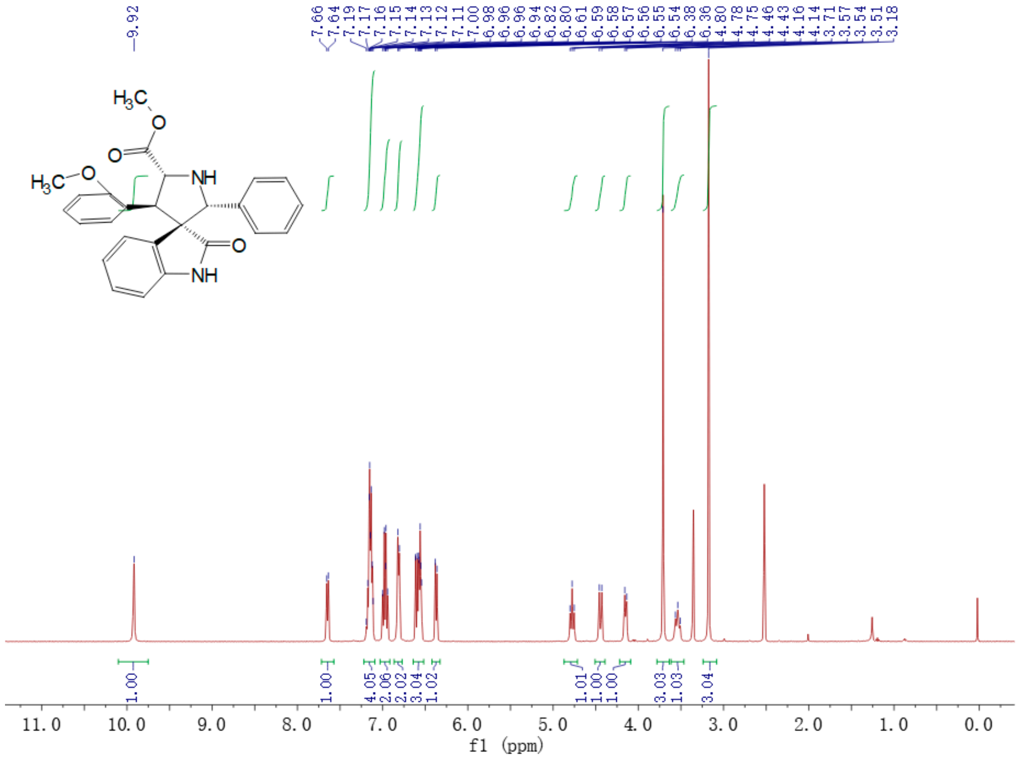

Fig. S115 The ^{1}H NMR (400 MHz, DMSO-d$_6$) of **3v**

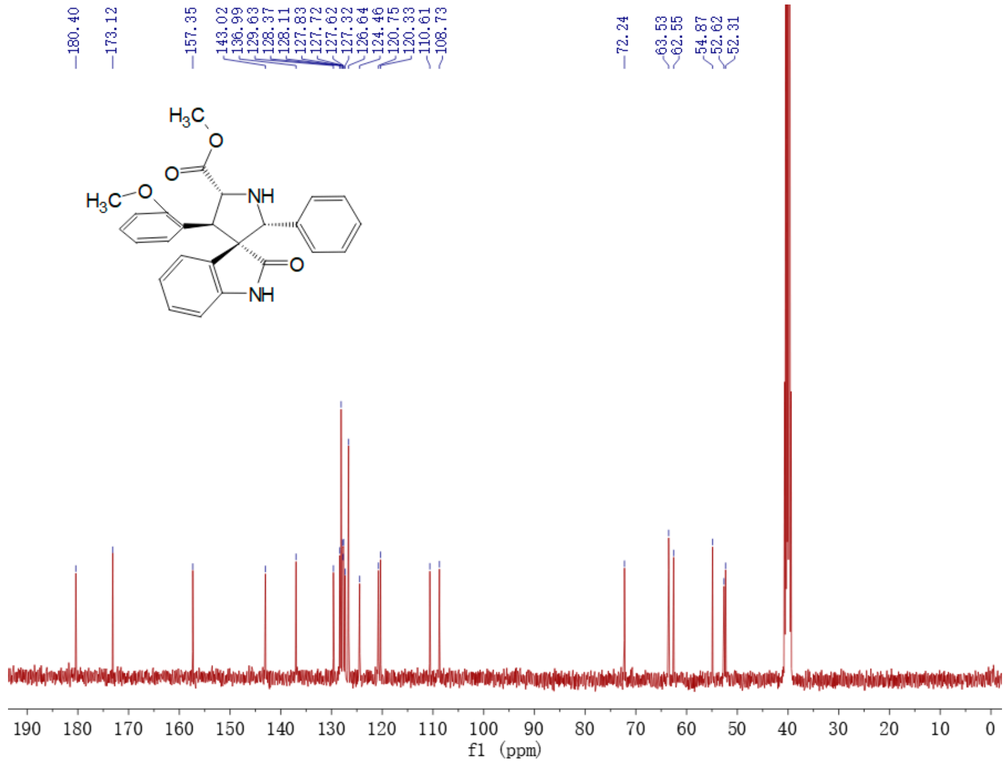

Fig. S116 The ^{13}C NMR (101 MHz, DMSO-d$_6$) of **3v**

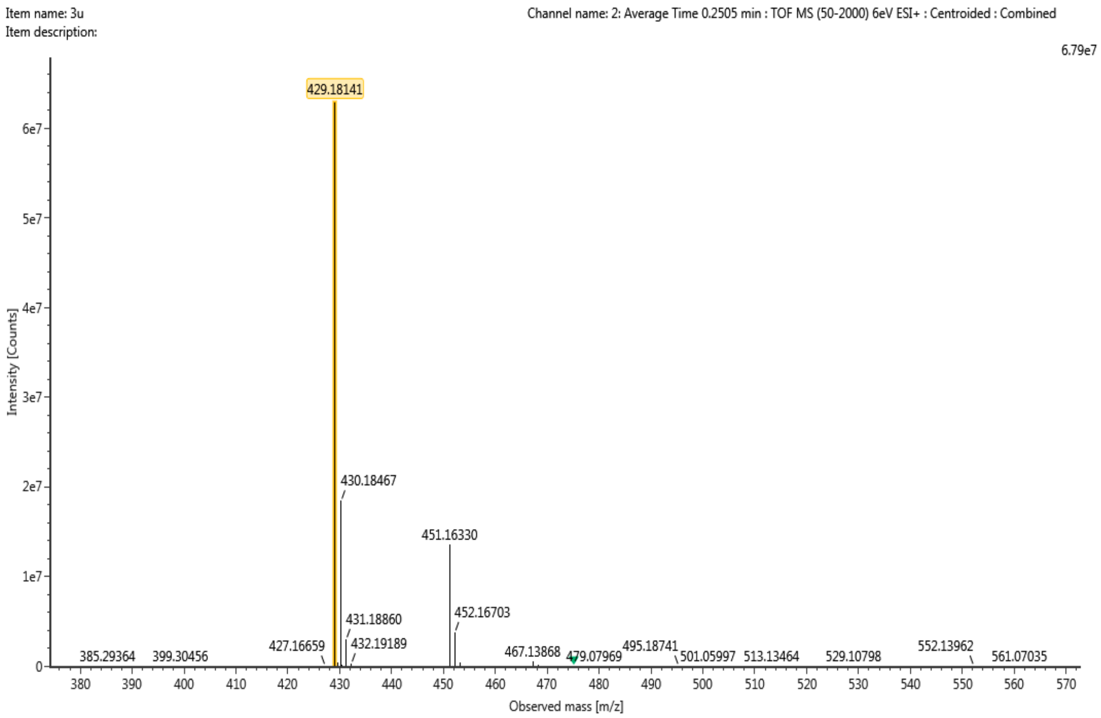

Fig. S117 The Mass spectrogram of **3v**

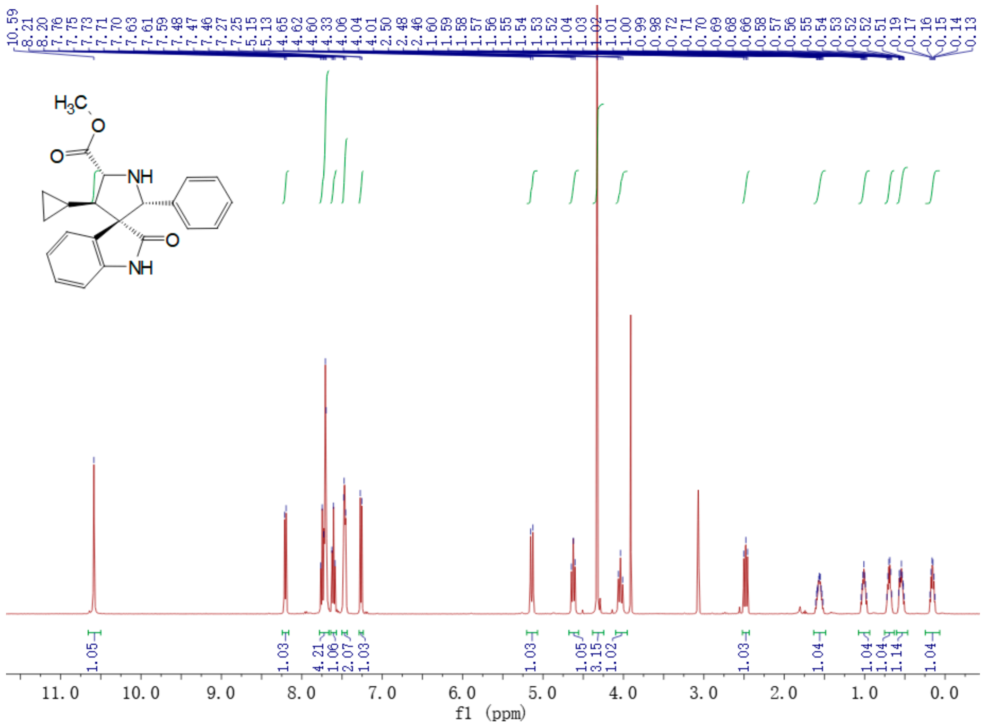

Fig. S118 The ^{1}H NMR (400 MHz, DMSO-d6) of **3w**

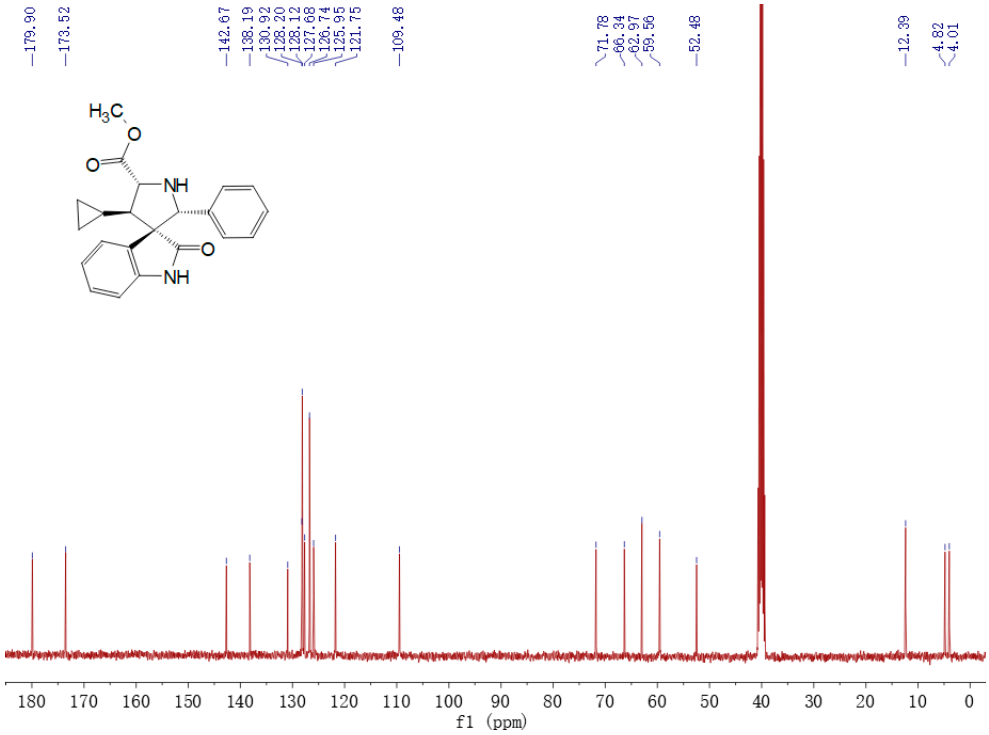

Fig. S119 The ^{13}C NMR (101 MHz, DMSO-d6) of **3w**

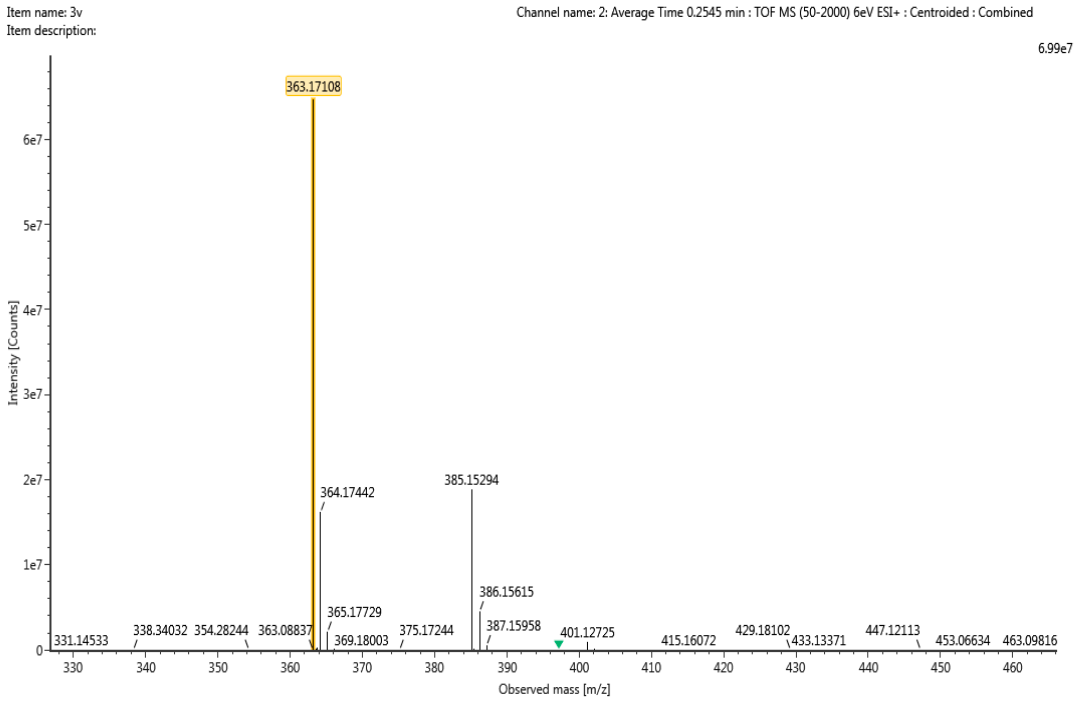

Fig. S120 The Mass spectrogram of **3w**

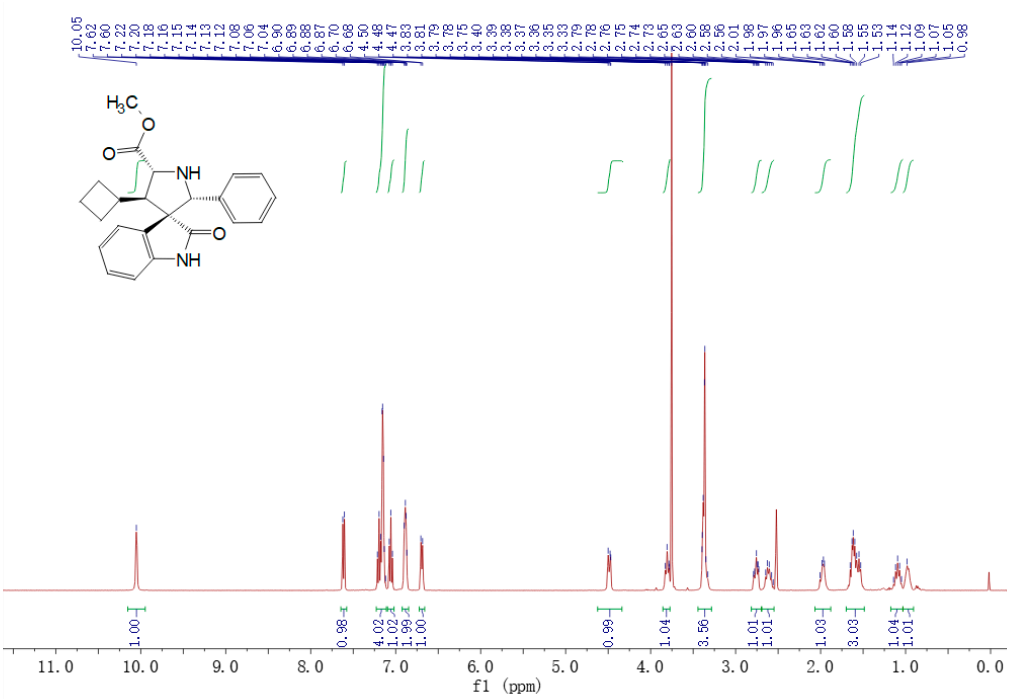

Fig. S121 The ^{1}H NMR (400 MHz, DMSO-d$_6$) of **3x**

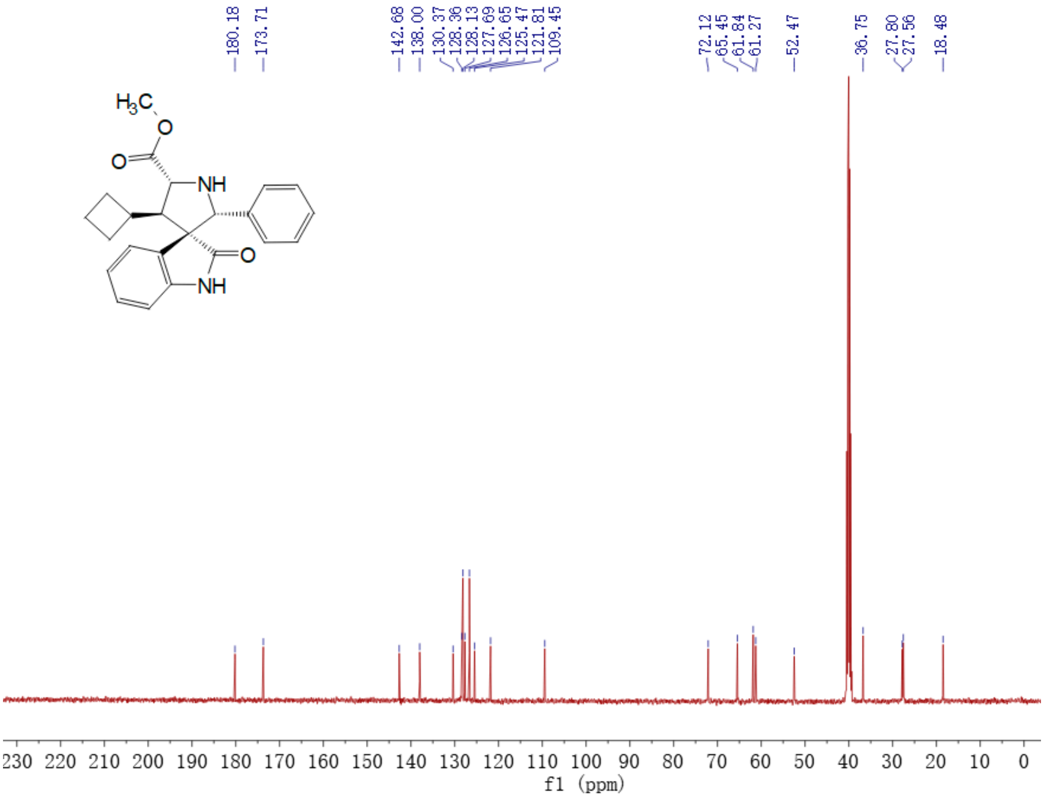

Fig. S122 The ^{13}C NMR (101 MHz, DMSO-d$_6$) of **3x**

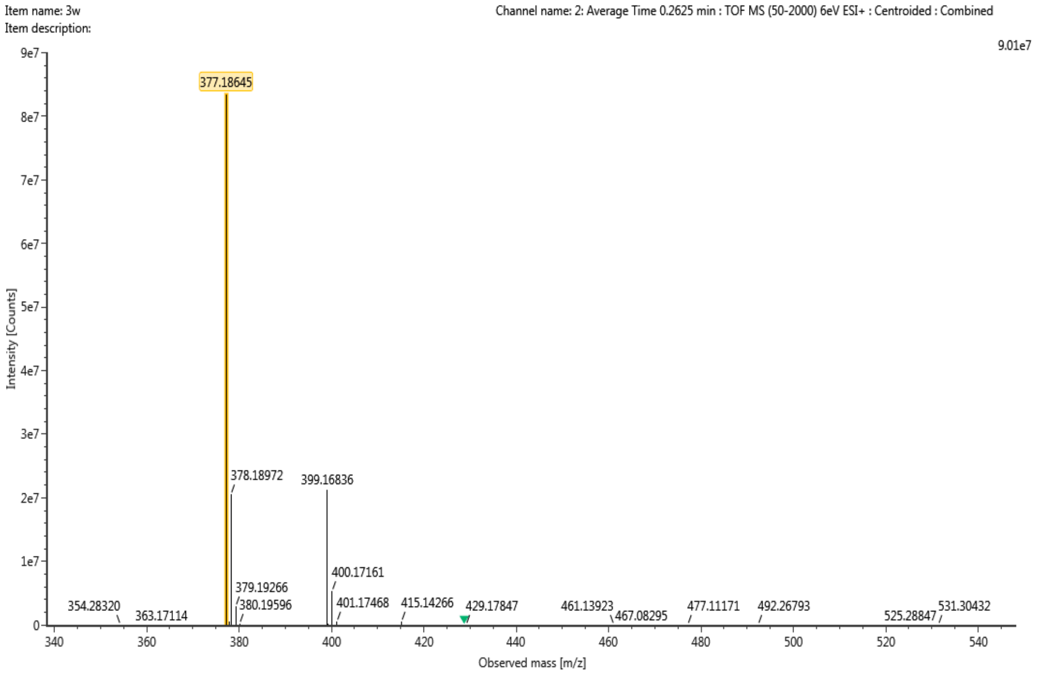

Fig. S123 The Mass spectrogram of **3x**

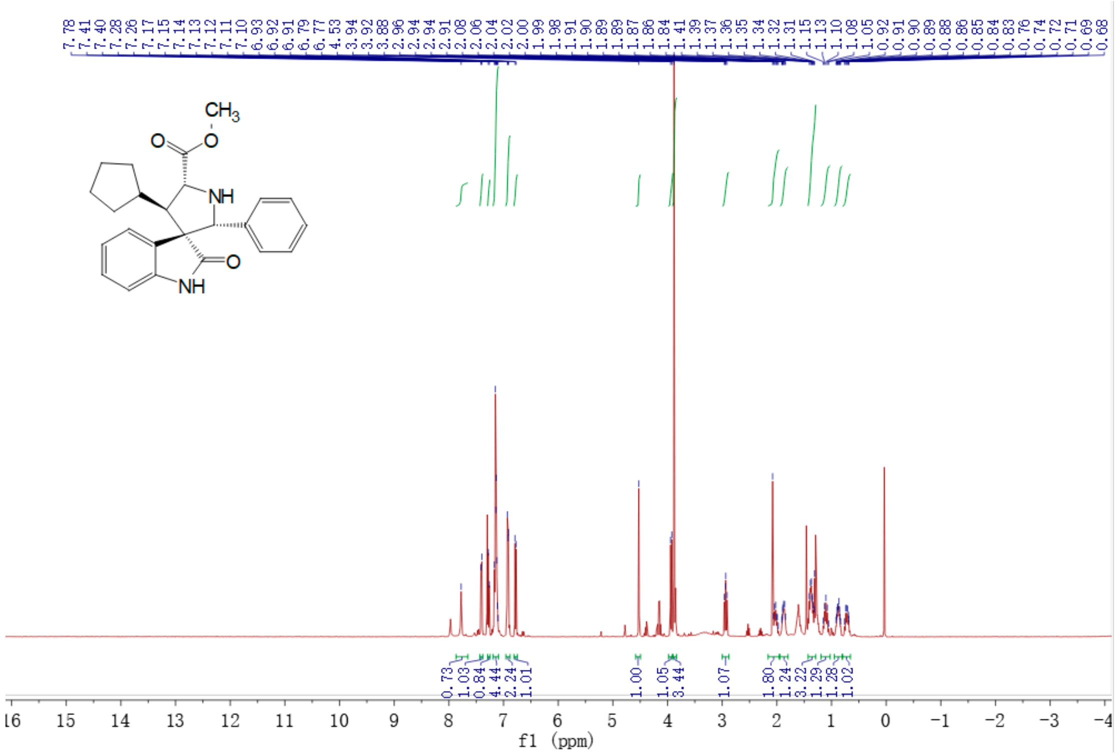

Fig. S124 The ^{1}H NMR (400 MHz, CDCl3) of **3y**

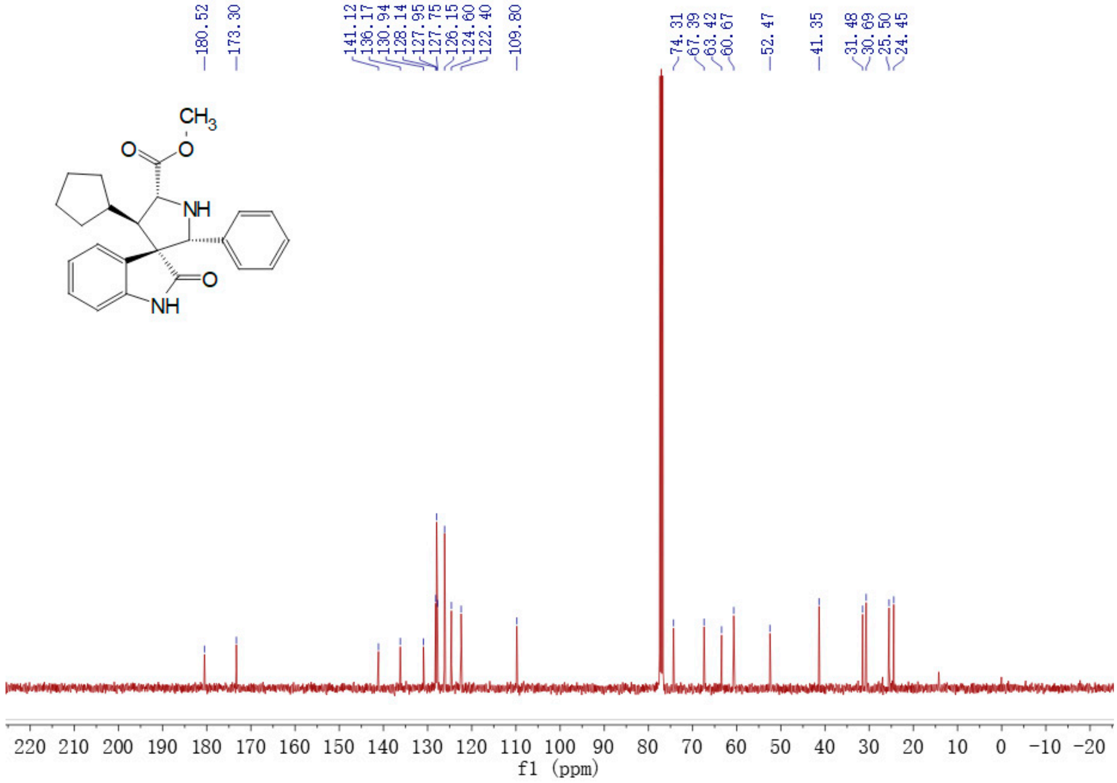

Fig. S125 The ^{13}C NMR (101 MHz, CDCl3) of **3y**

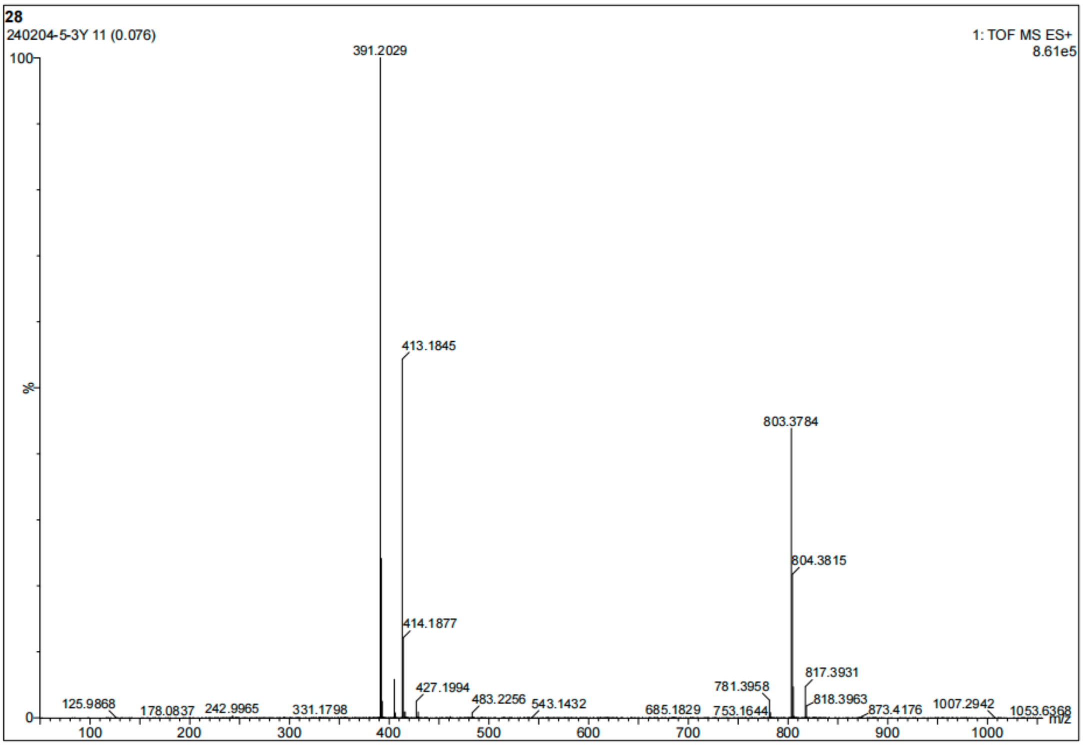

Fig. S126 The Mass spectrogram of **3y**

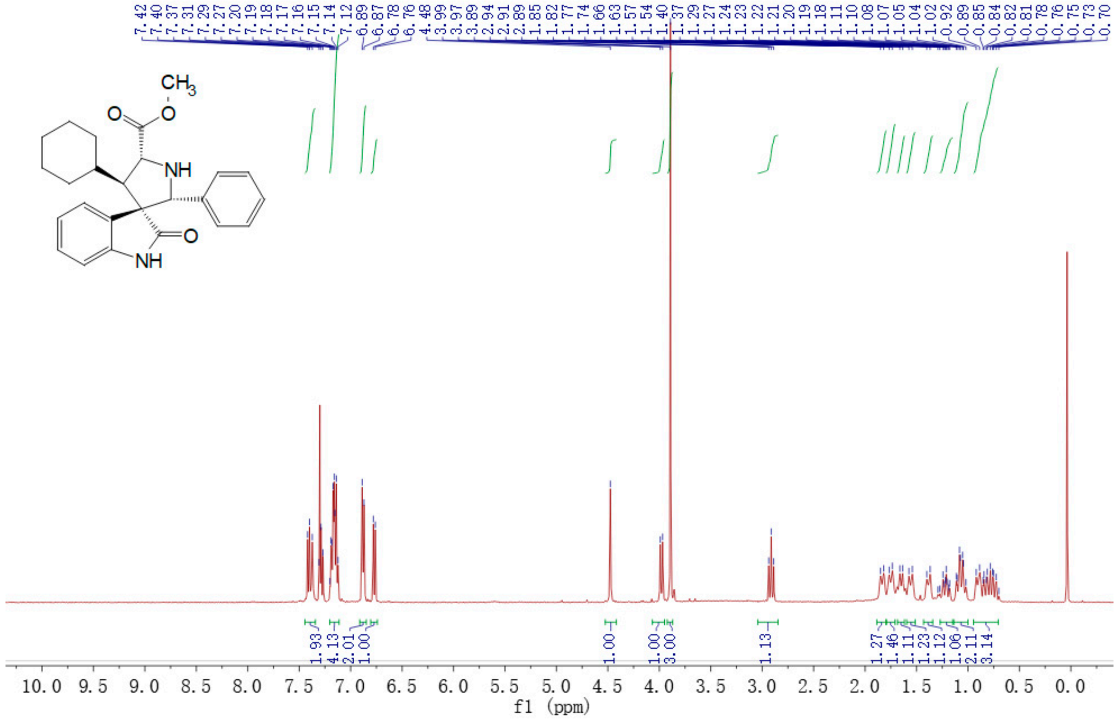

Fig. S127 The ^{1}H NMR (400 MHz, $CDCl_3$) of **3z**

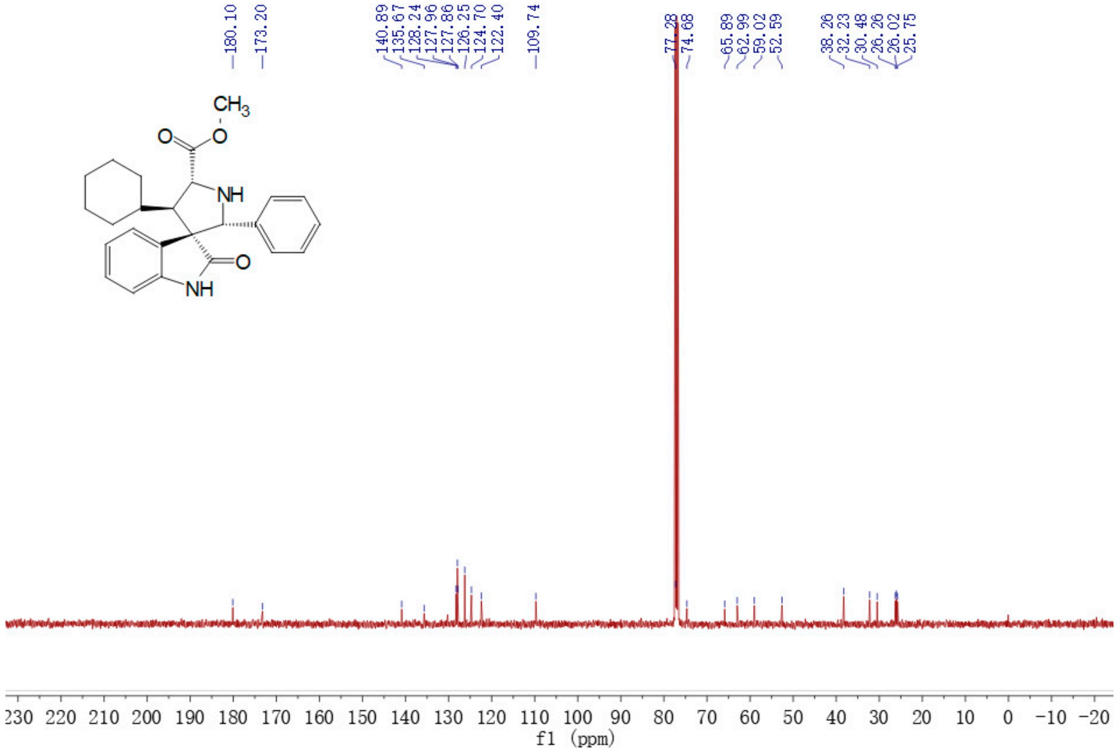

Fig. S128 The ^{13}C NMR (101 MHz, $CDCl_3$) of **3z**

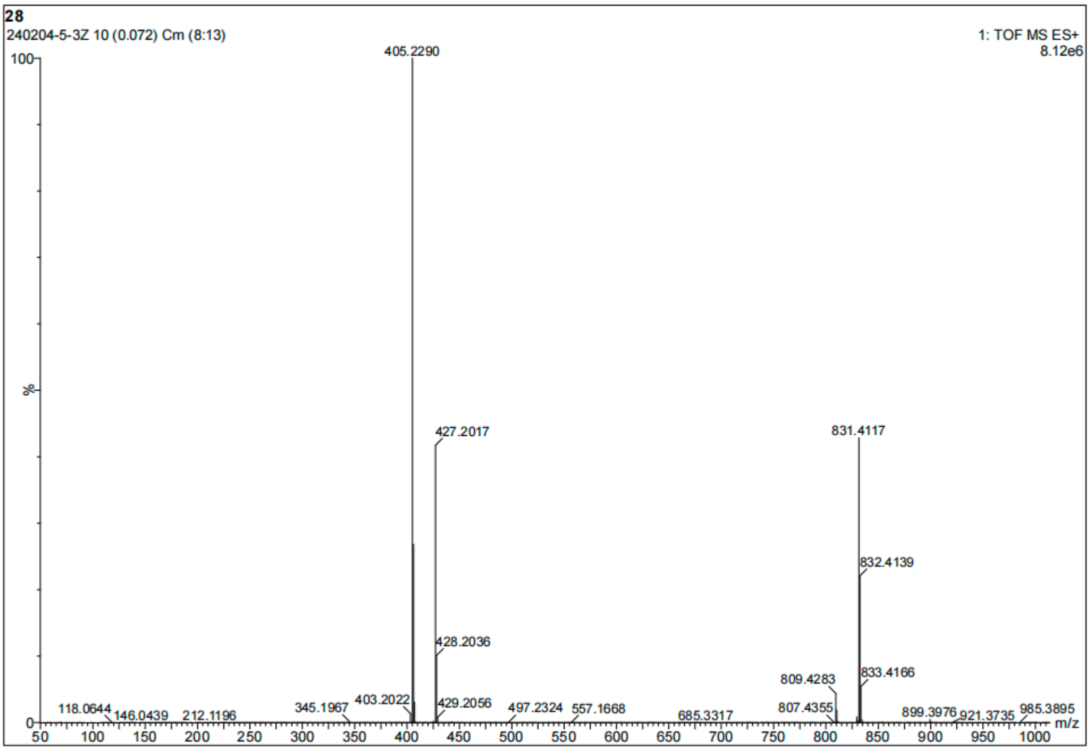

Fig. S129 The Mass spectrogram of **3z**

7. Spectrums of target compounds 4a-4s

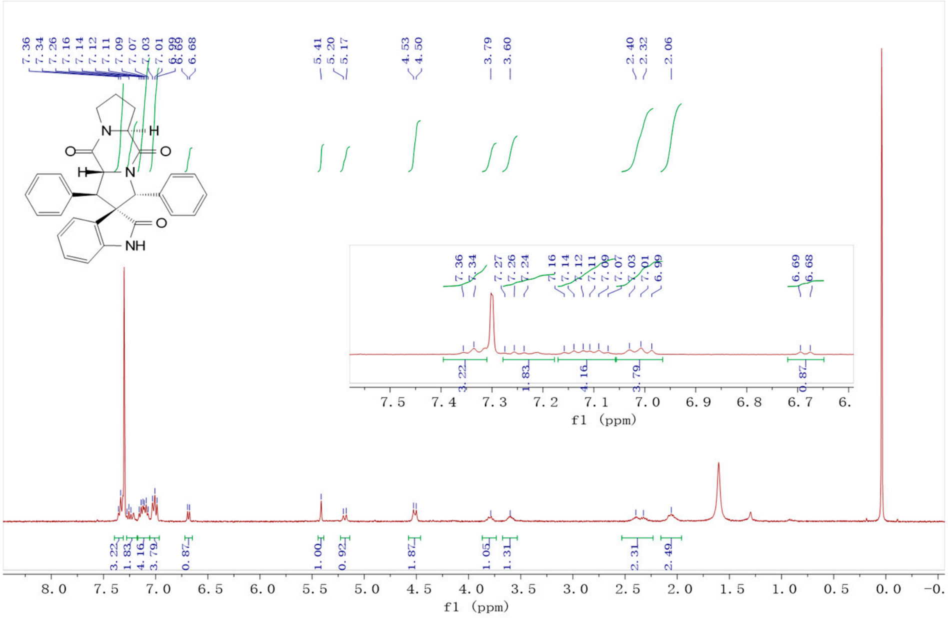

Fig. S130 The ^{1}H NMR (400 MHz, $CDCl_3$) of **4a**

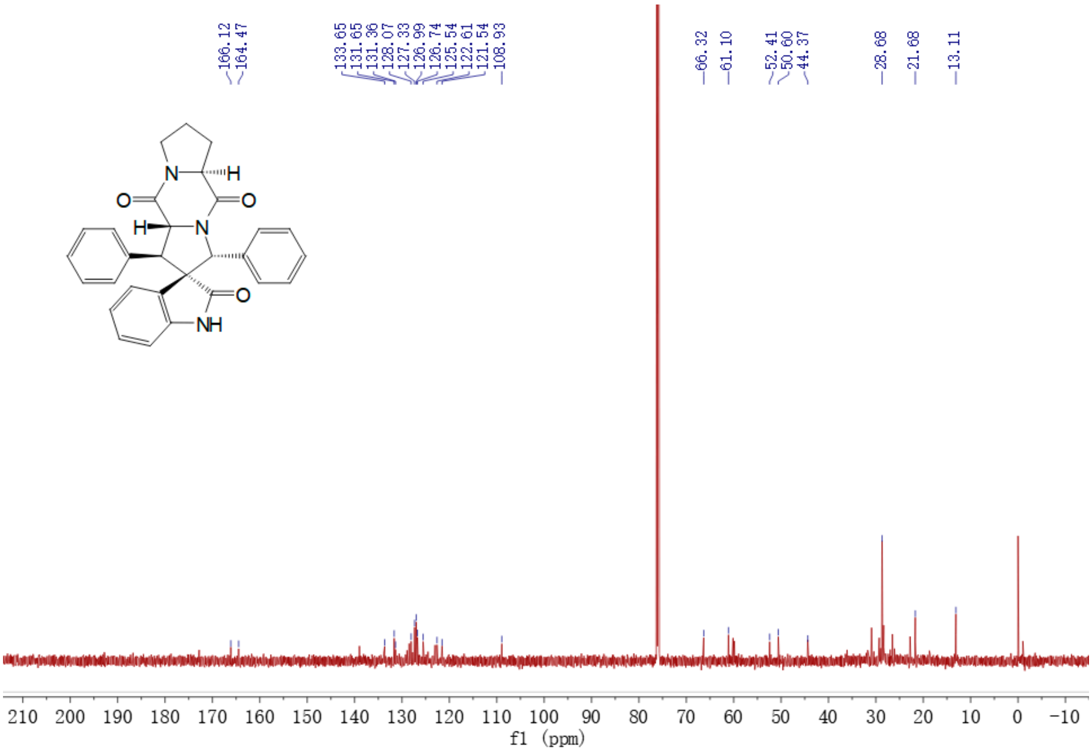

Fig. S131 The ^{13}CNMR (151 MHz, $CDCl_3$) of **4a**

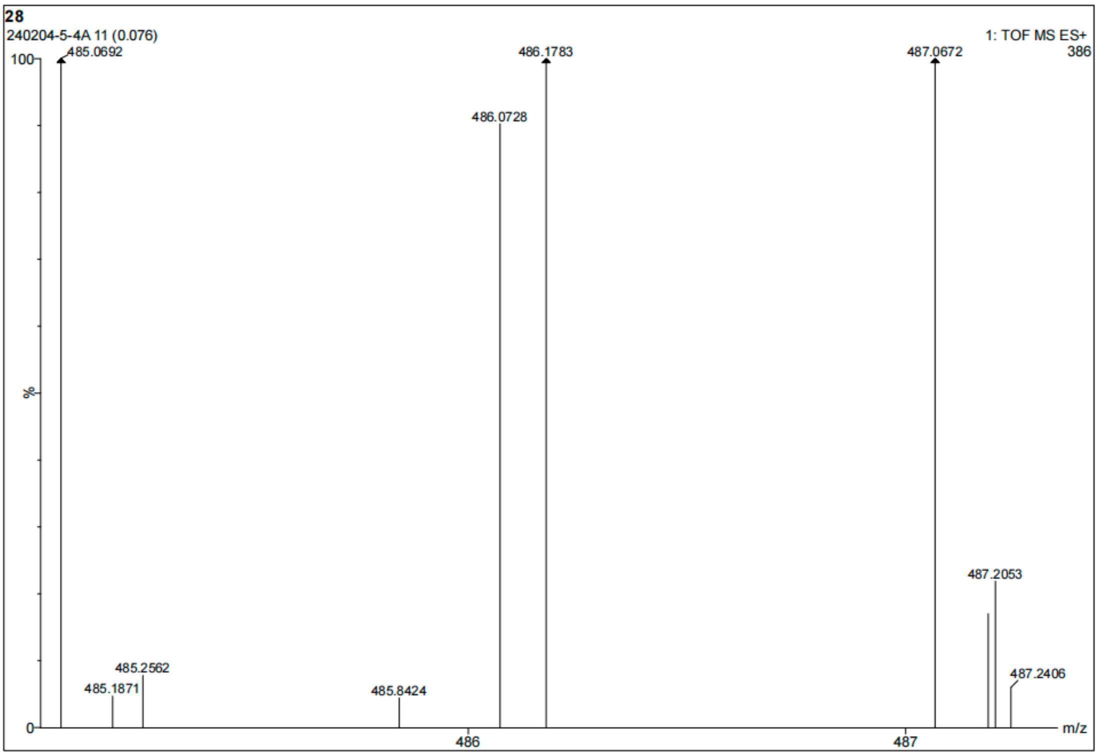

Fig. S132 The Mass spectrogram of **4a**

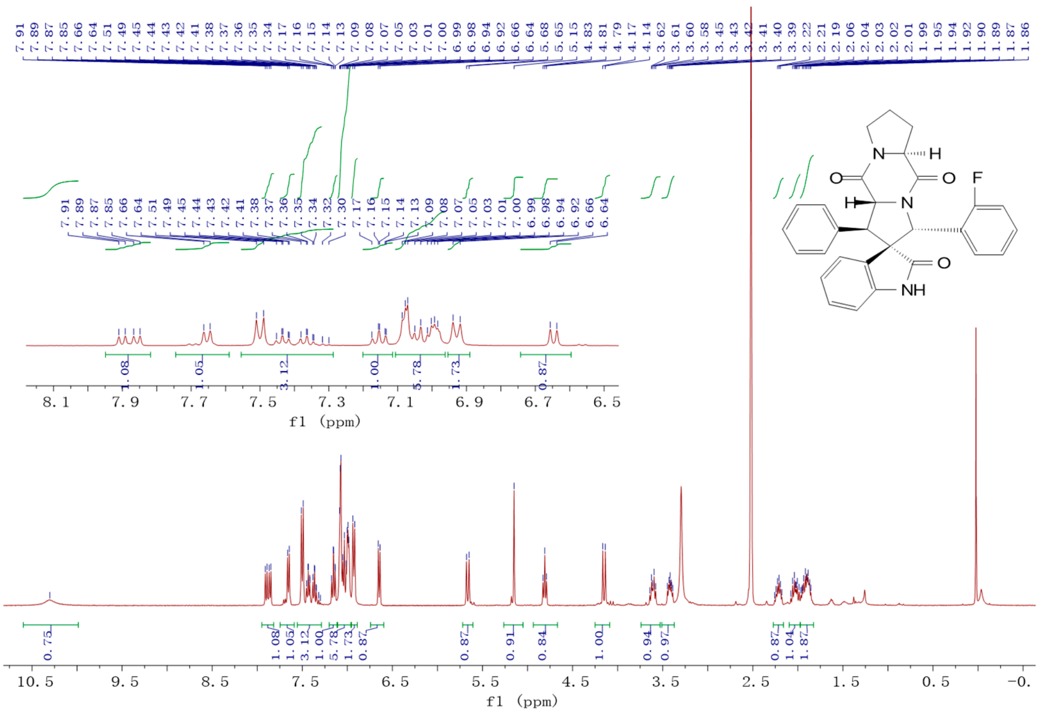

Fig. S133 The ^{1}H NMR (400 MHz, DMSO-d$_6$) of **4b**

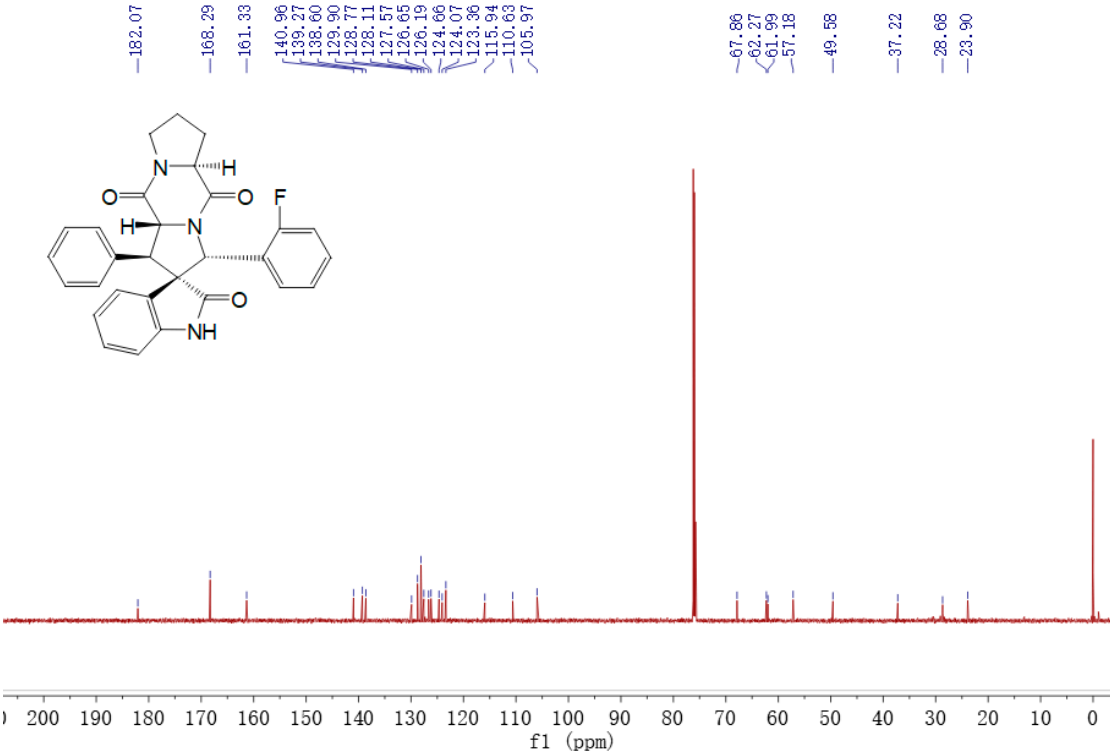

Fig. S134 The ^{13}C NMR (151 MHz, CDCl$_3$) of **4b**

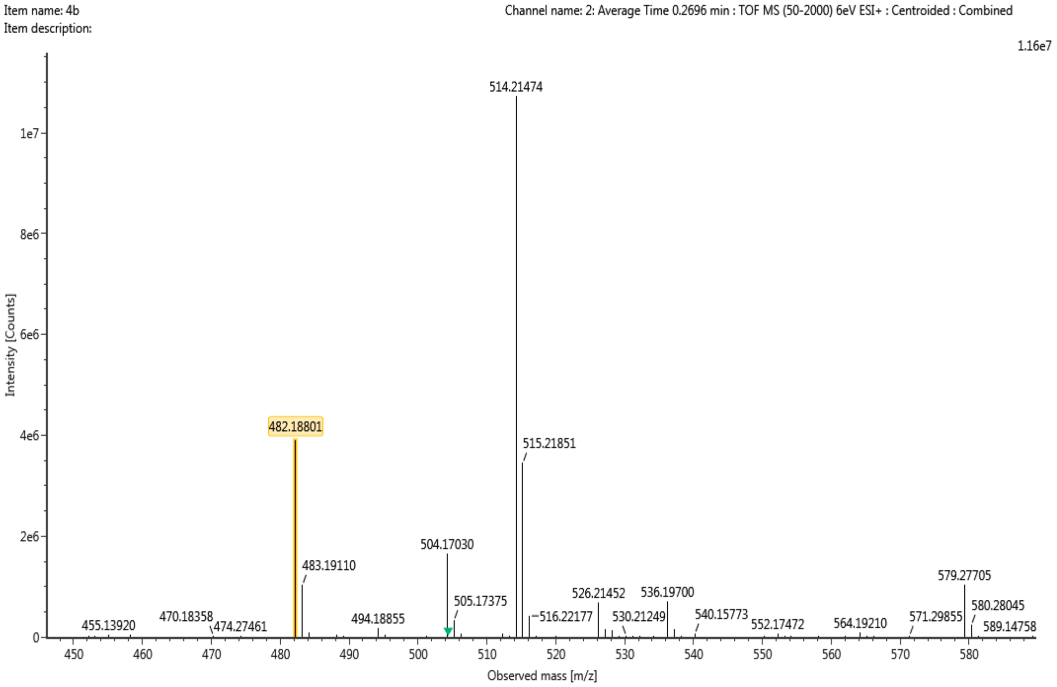

Fig. S135 The Mass spectrogram of **4b**

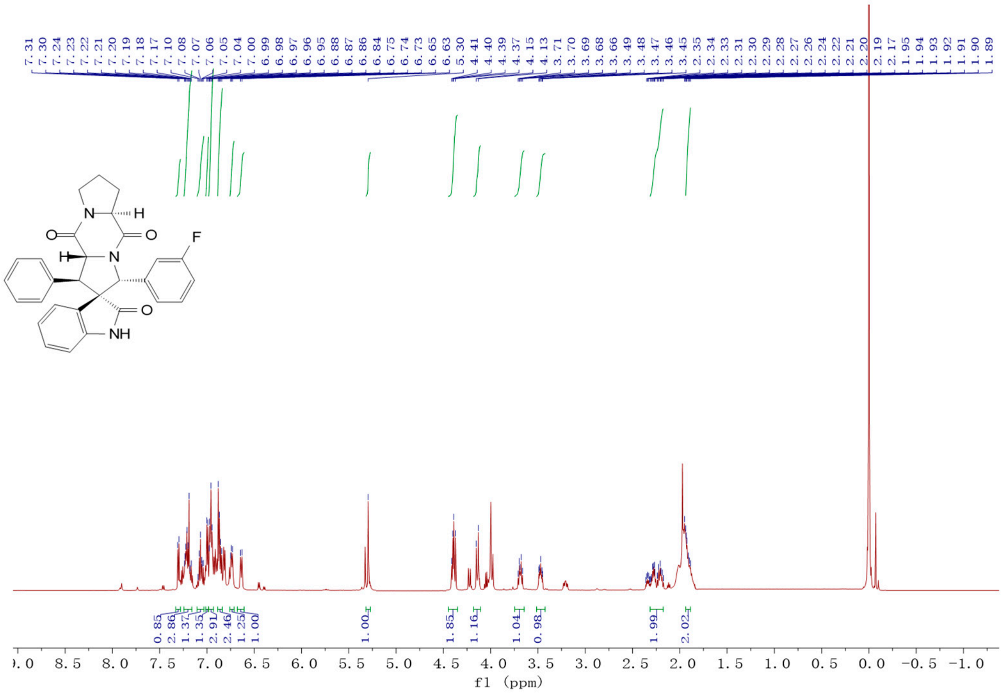

Fig. S136 The ^{1}H NMR (600 MHz, CDCl3) of **4c**

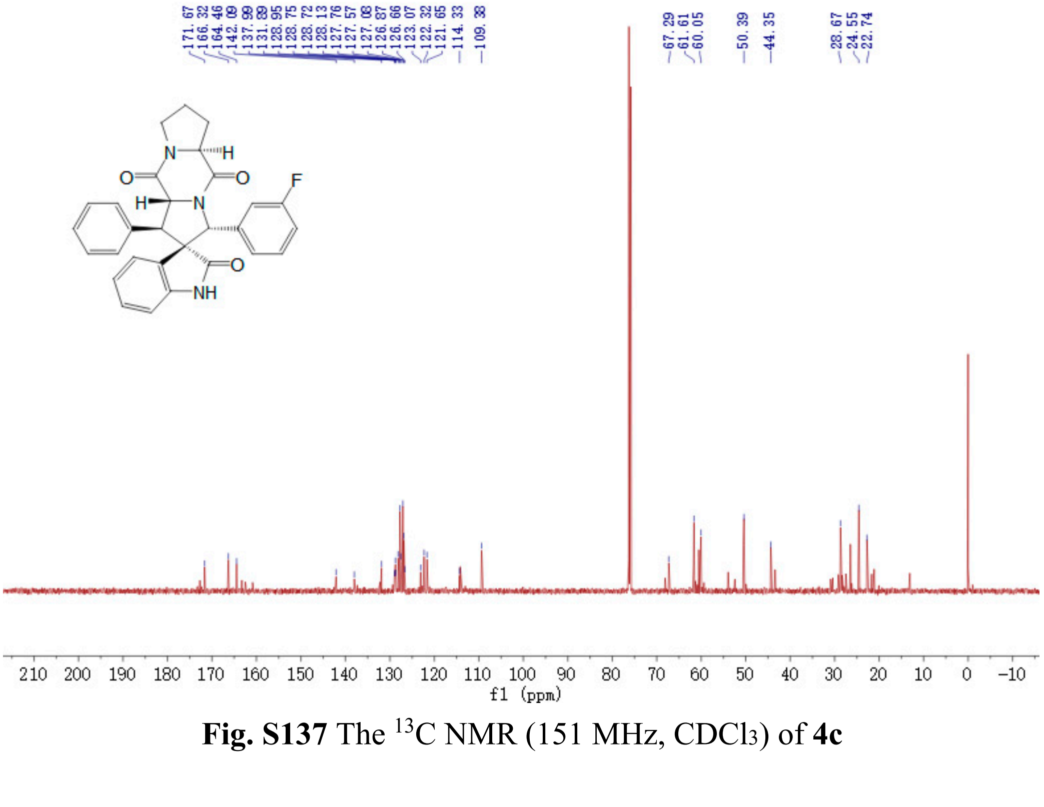

Fig. S137 The ^{13}C NMR (151 MHz, CDCl3) of **4c**

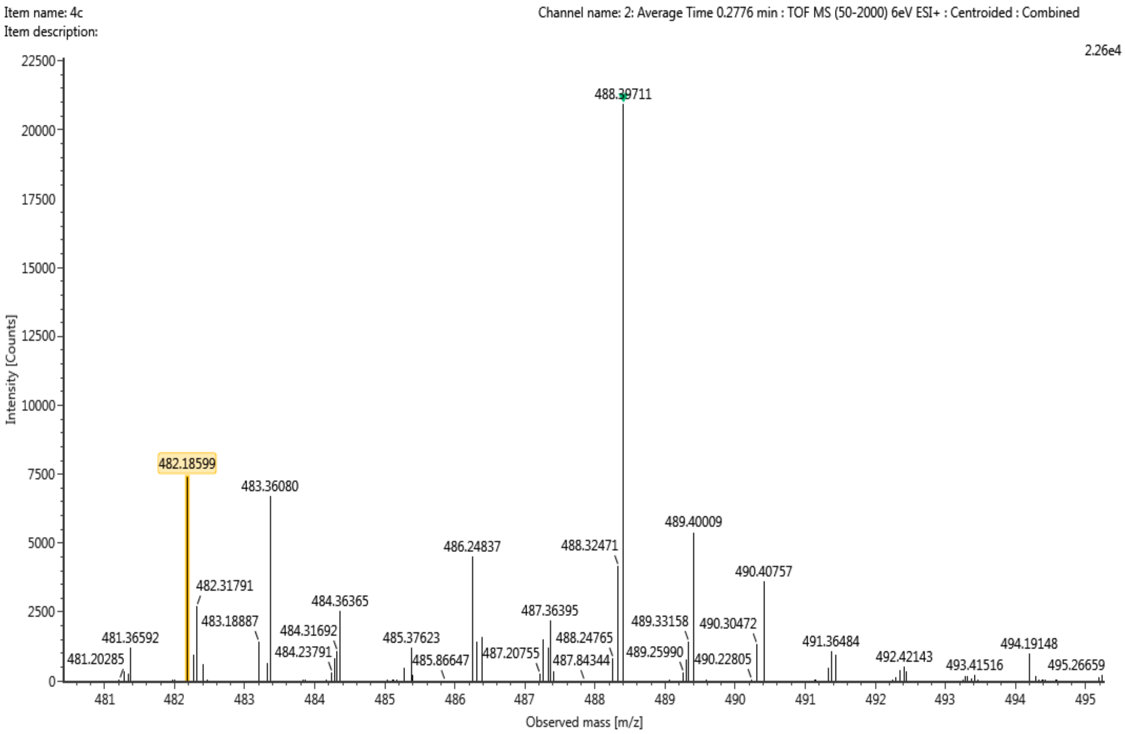

Fig. S138 The Mass spectrogram of **4c**

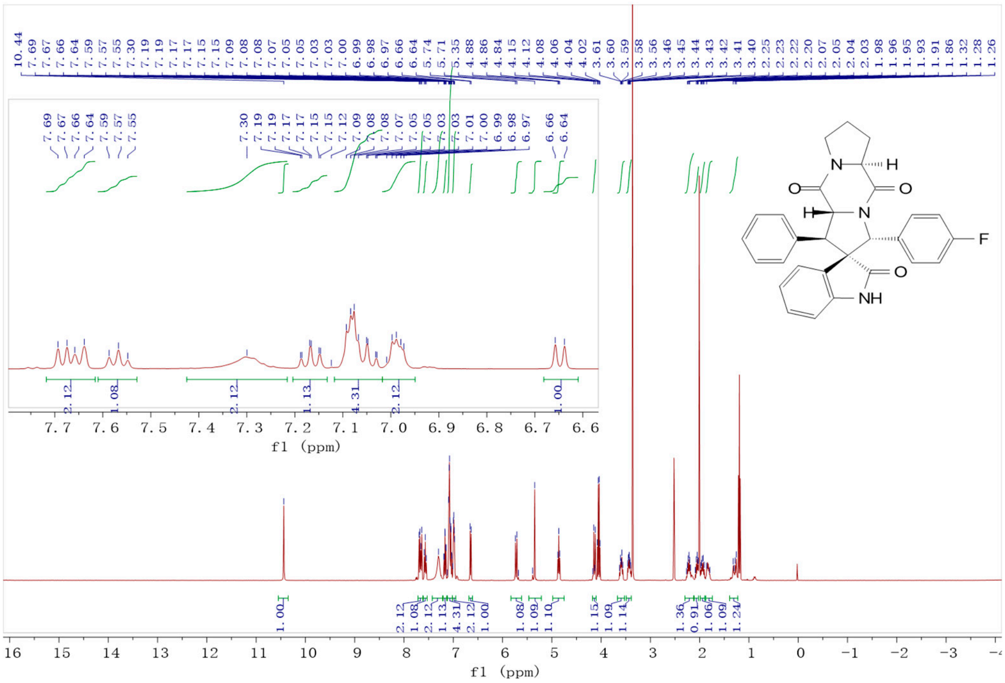

Fig. S139 The ^{1}H NMR (400 MHz, DMSO-d$_6$) of **4d**

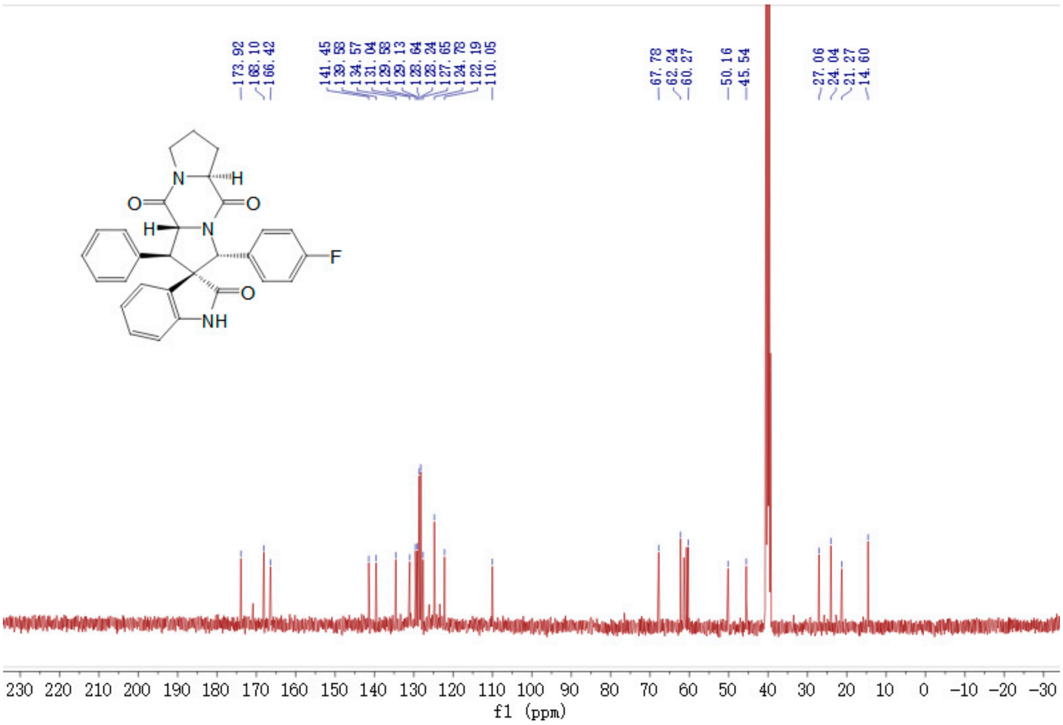

Fig. S140 The ^{13}C NMR (151 MHz, DMSO-d$_6$) of **4d**

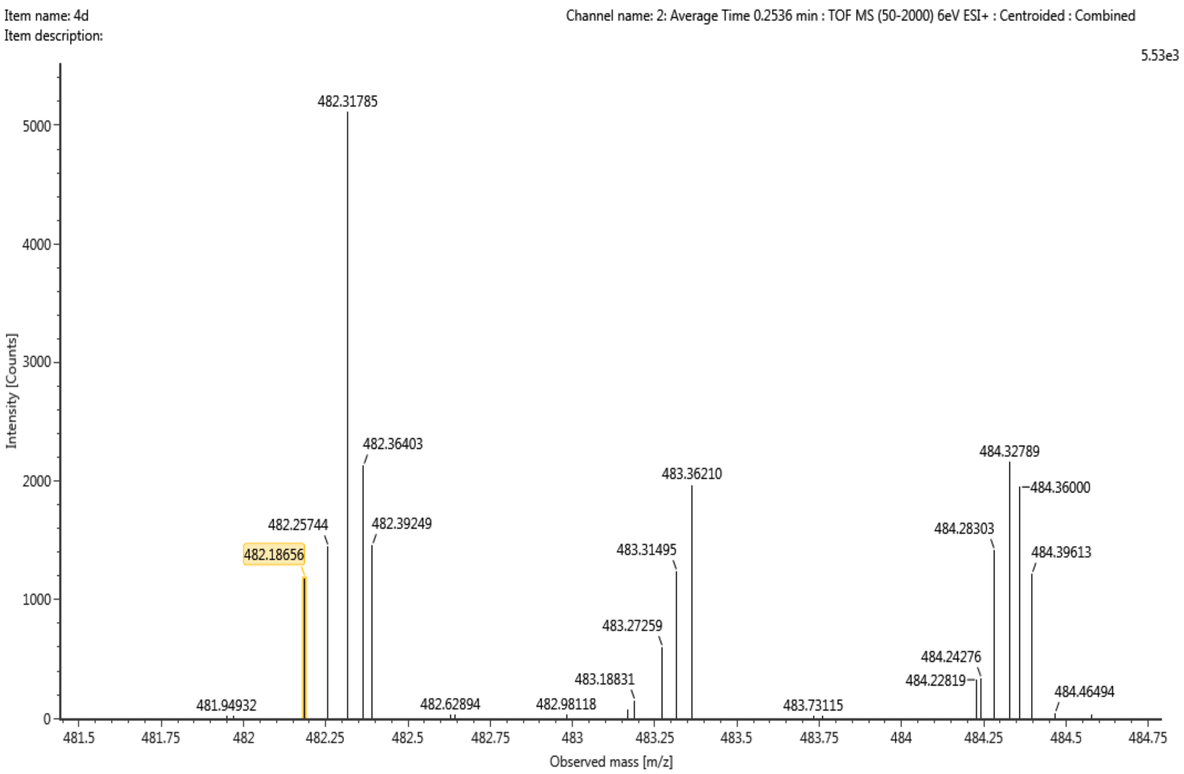

Fig. S141 The Mass spectrogram of **4d**

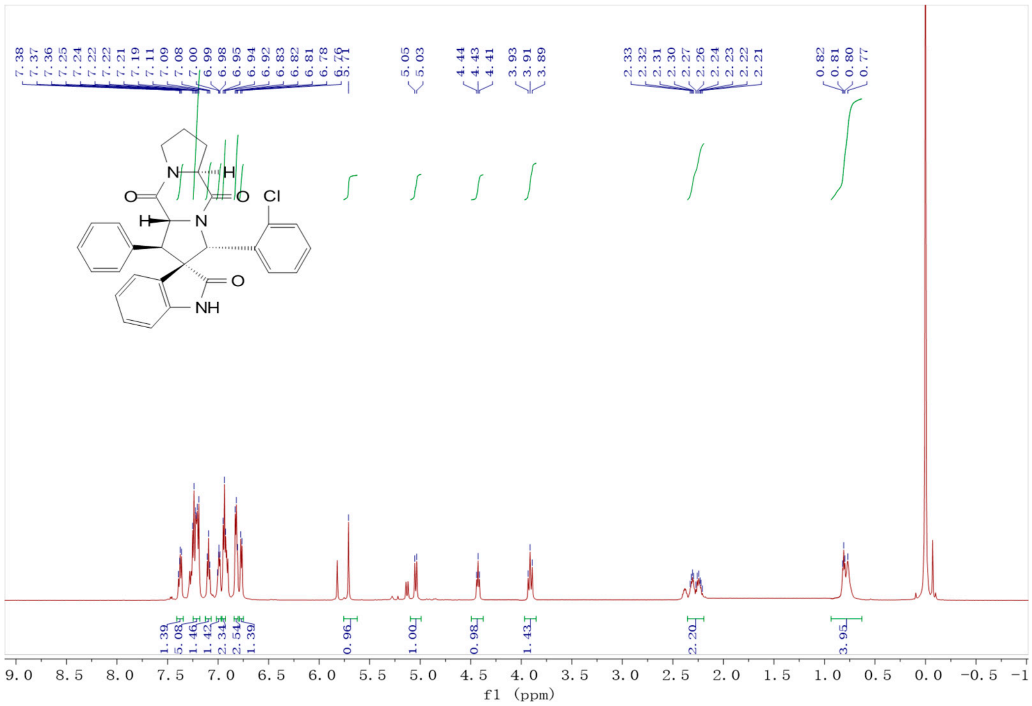

Fig. S142 The ^{1}H NMR (600 MHz, $CDCl_3$) of **4e**

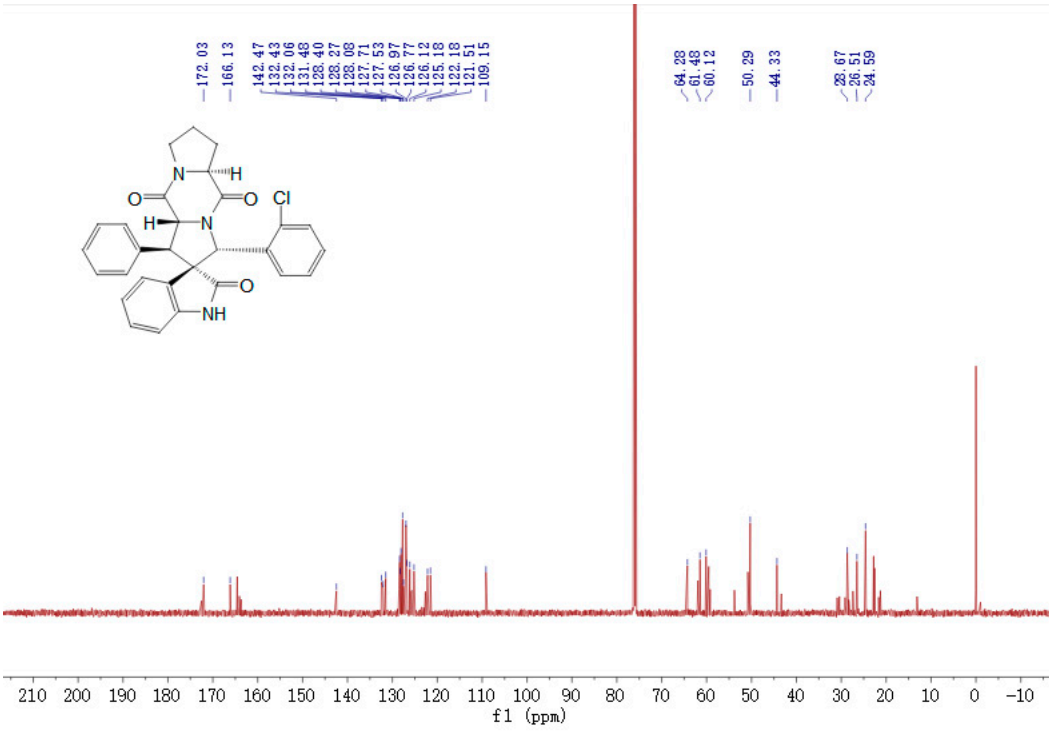

Fig. S143 The ^{13}C NMR (151 MHz, $CDCl_3$) of **4e**

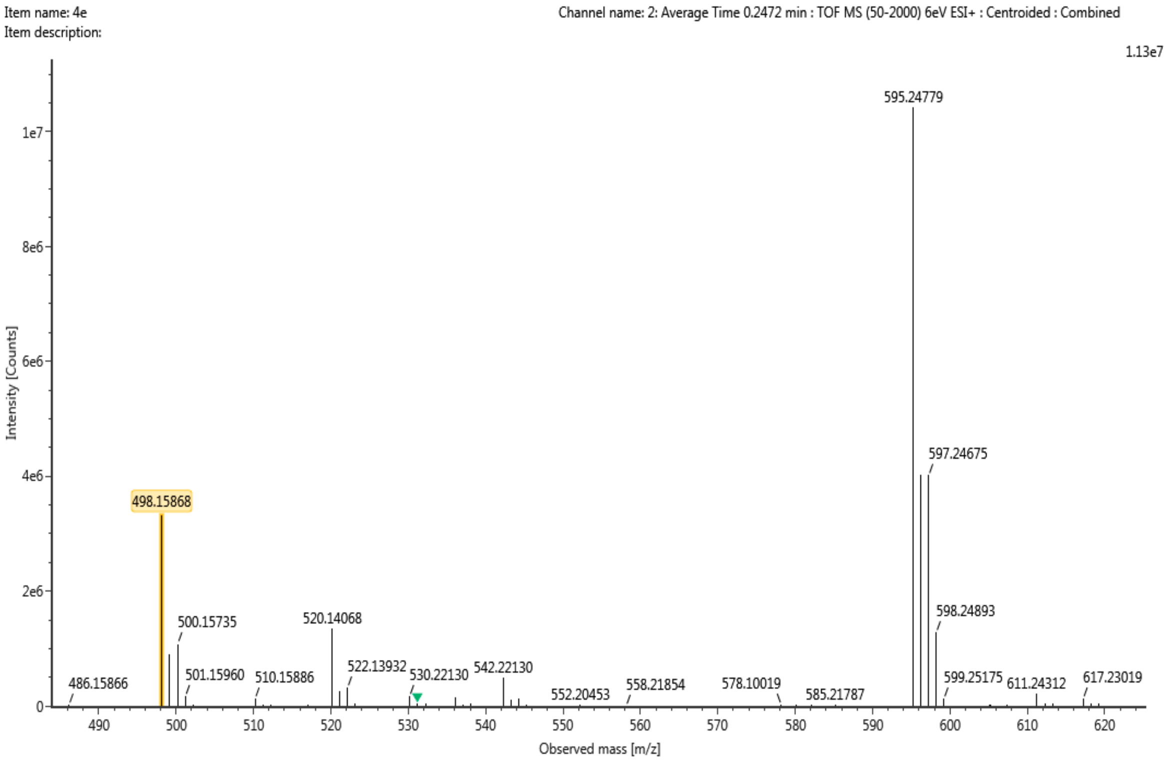

Fig. S144 The Mass spectrogram of **4e**

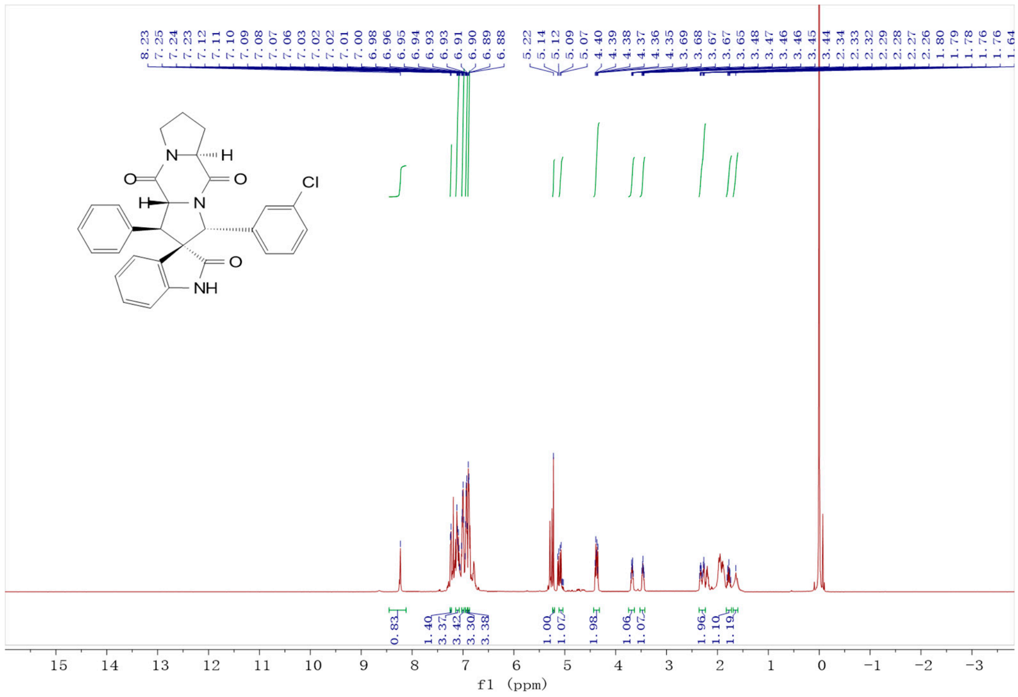

Fig. S145 The ^{1}H NMR (600 MHz, CDCl3) of **4f**

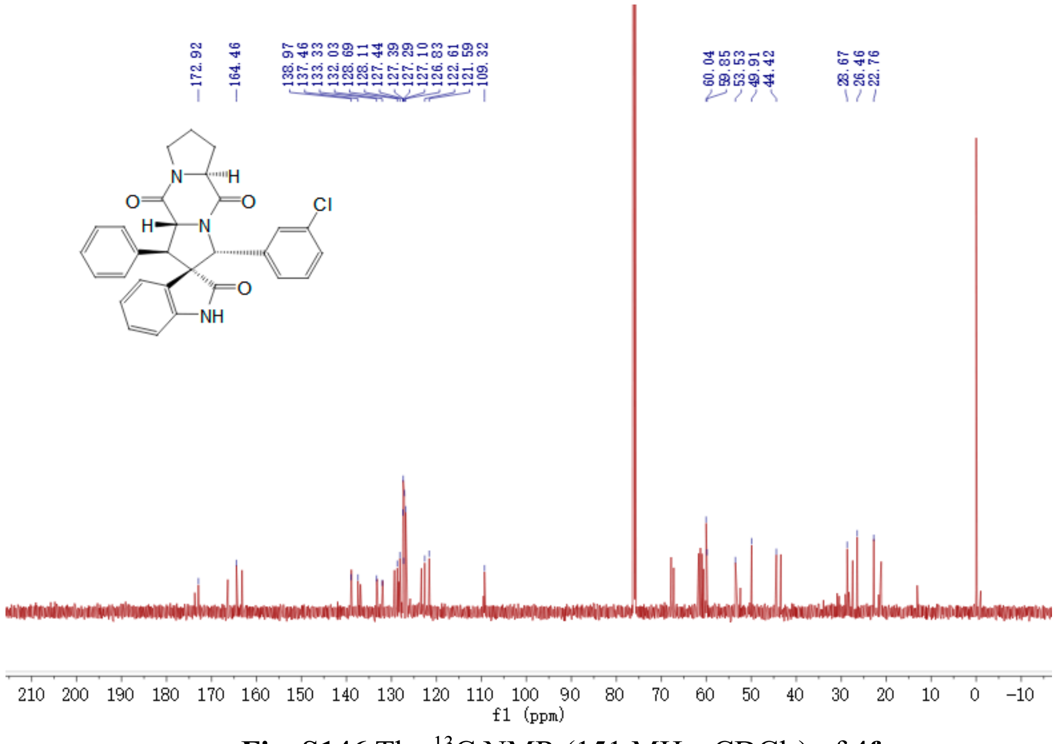

Fig. S146 The ^{13}C NMR (151 MHz, CDCl3) of **4f**

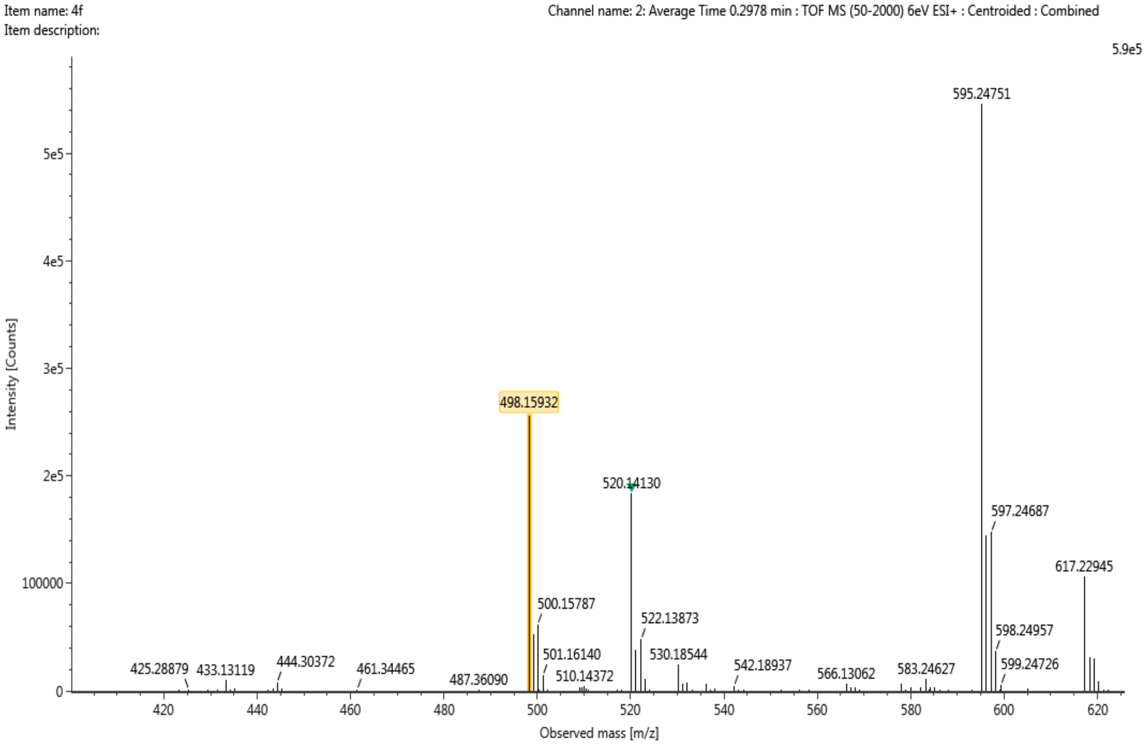

Fig. S147 The Mass spectrogram of **4f**

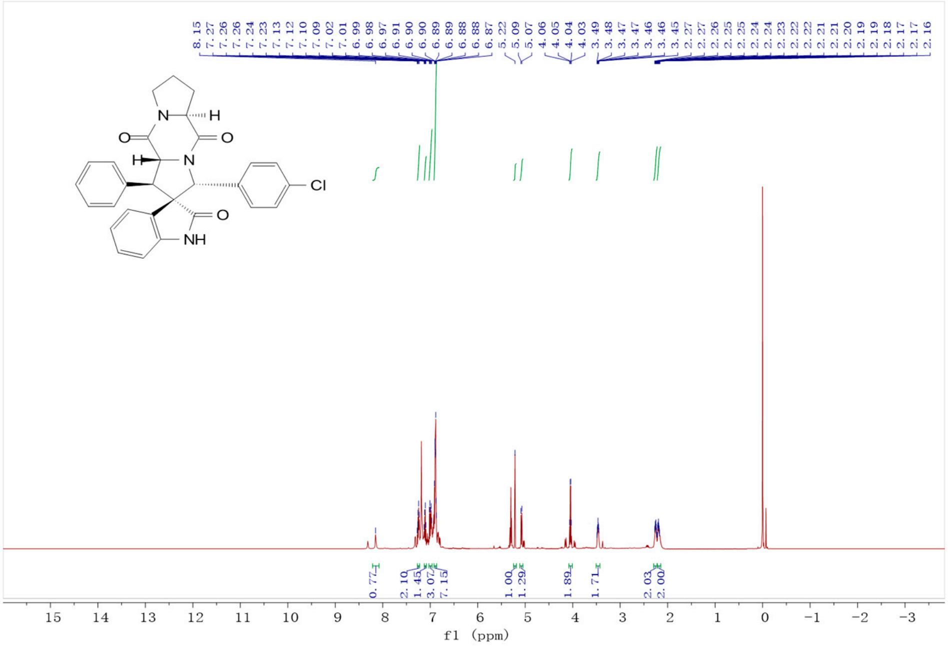

Fig. S148 The ^{1}H NMR (600 MHz, CDCl3) of **4g**

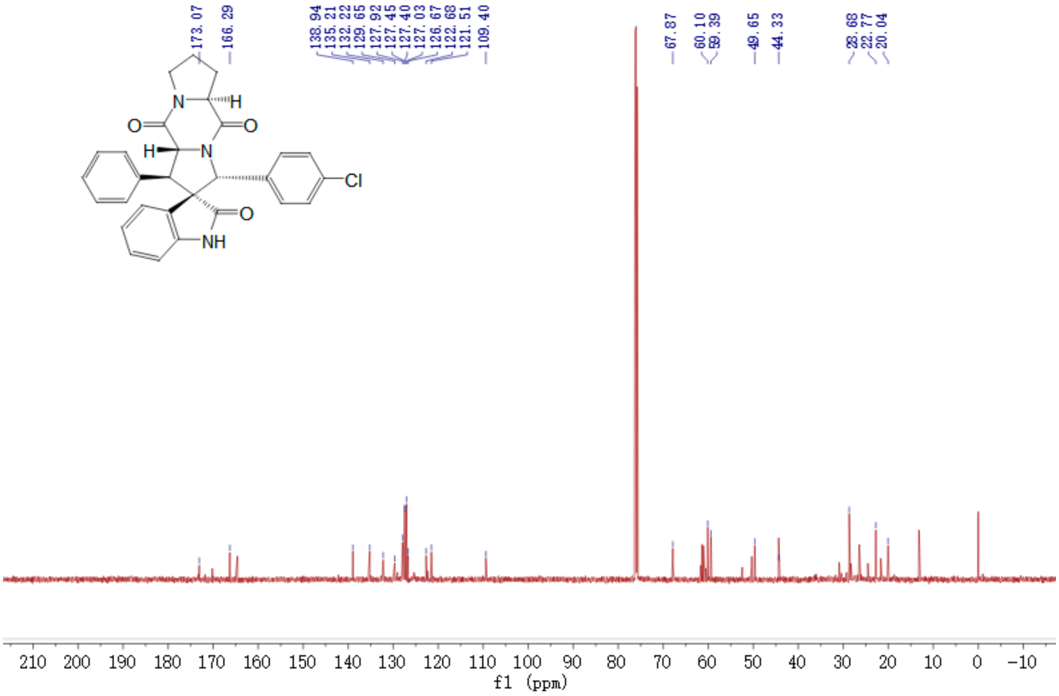

Fig. S149 The ^{13}C NMR (151 MHz, CDCl3) of **4g**

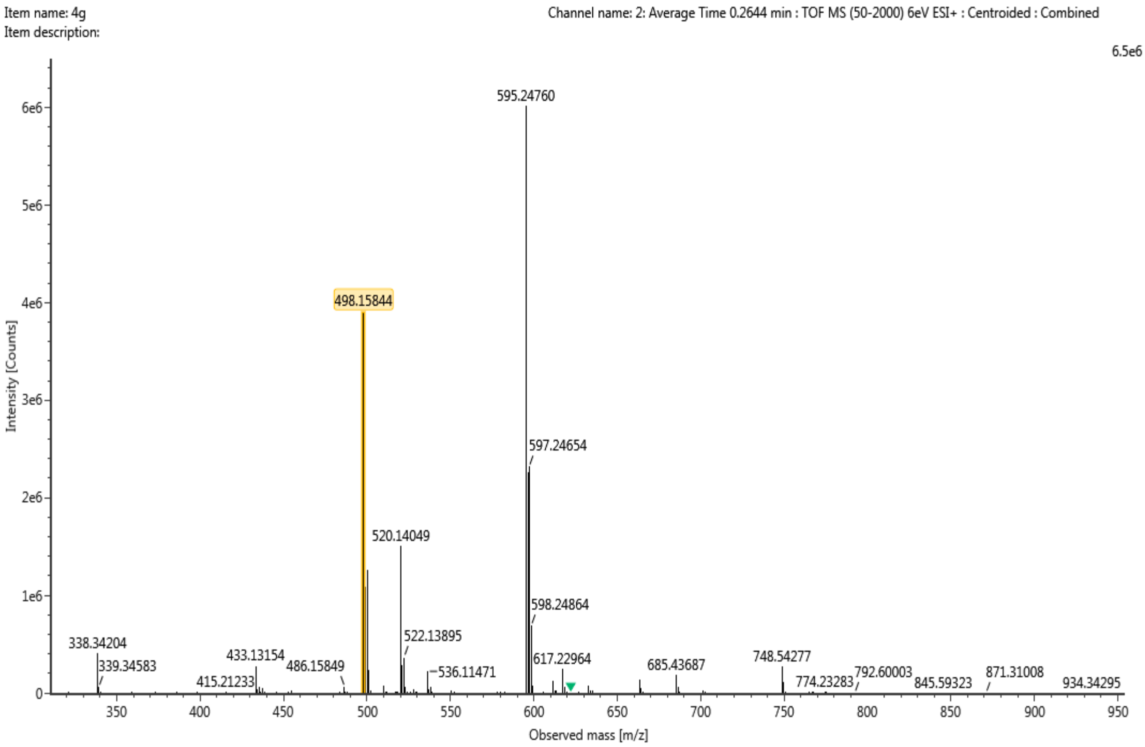

Fig. S150 The Mass spectrogram of **4g**

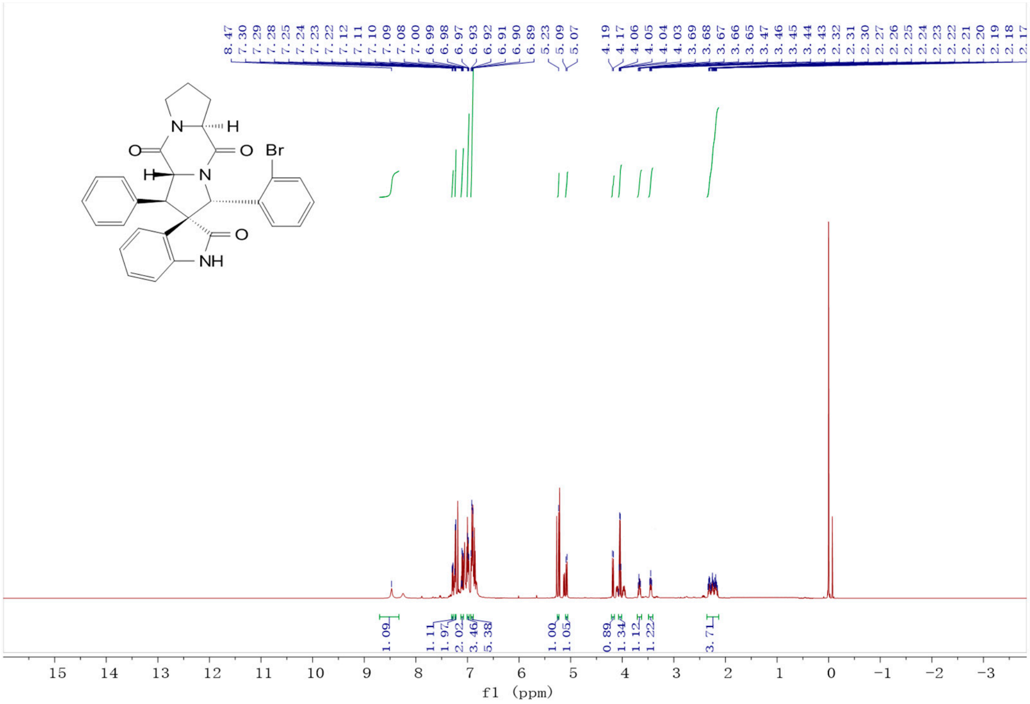

Fig. S151 The ^{1}H NMR (600 MHz, CDCl3) of **4h**

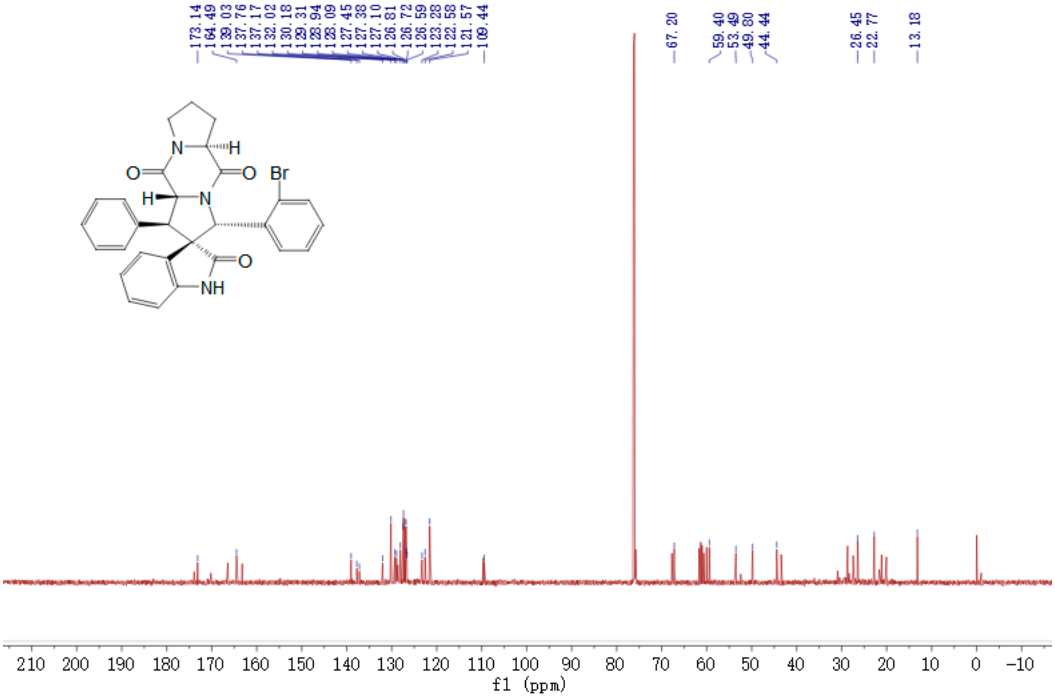

Fig. S152 The ^{13}C NMR (151 MHz, CDCl3) of **4h**

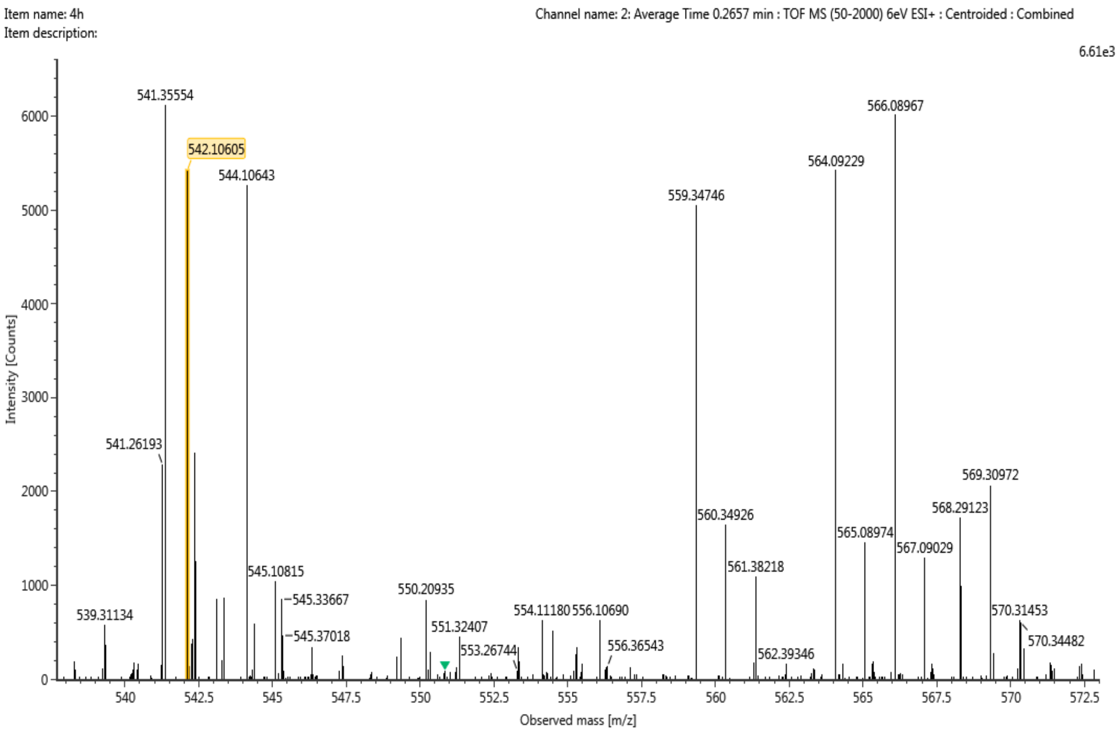

Fig. S153 The Mass spectrogram of **4h**

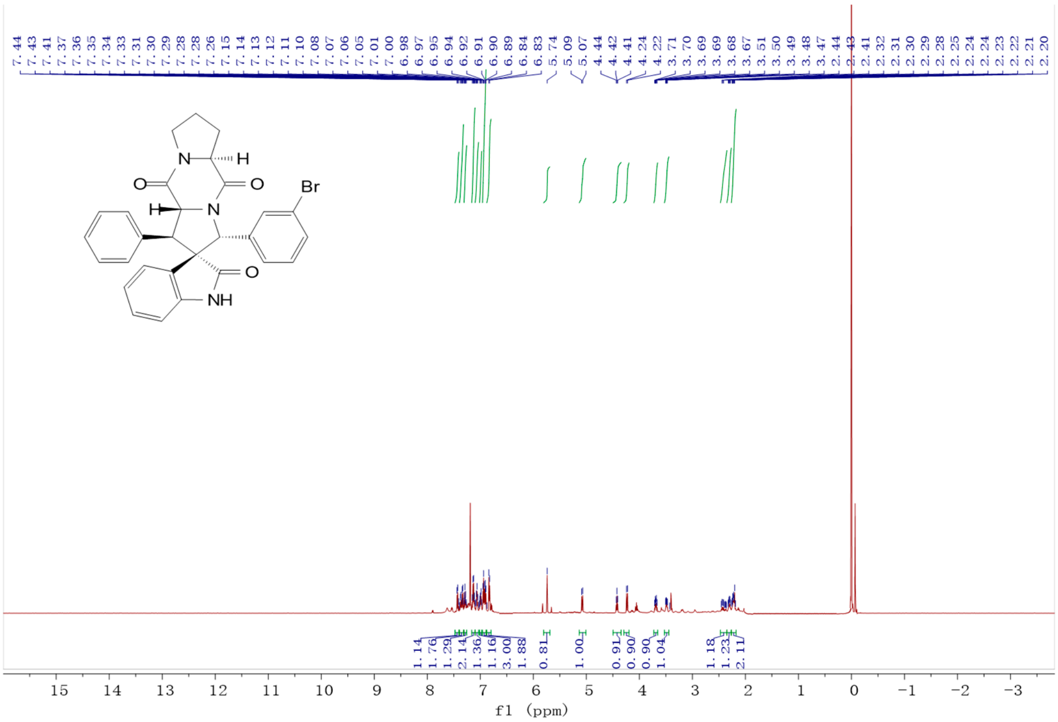

Fig. S154 The ^{1}H NMR (600 MHz, $CDCl_3$) of **4i**

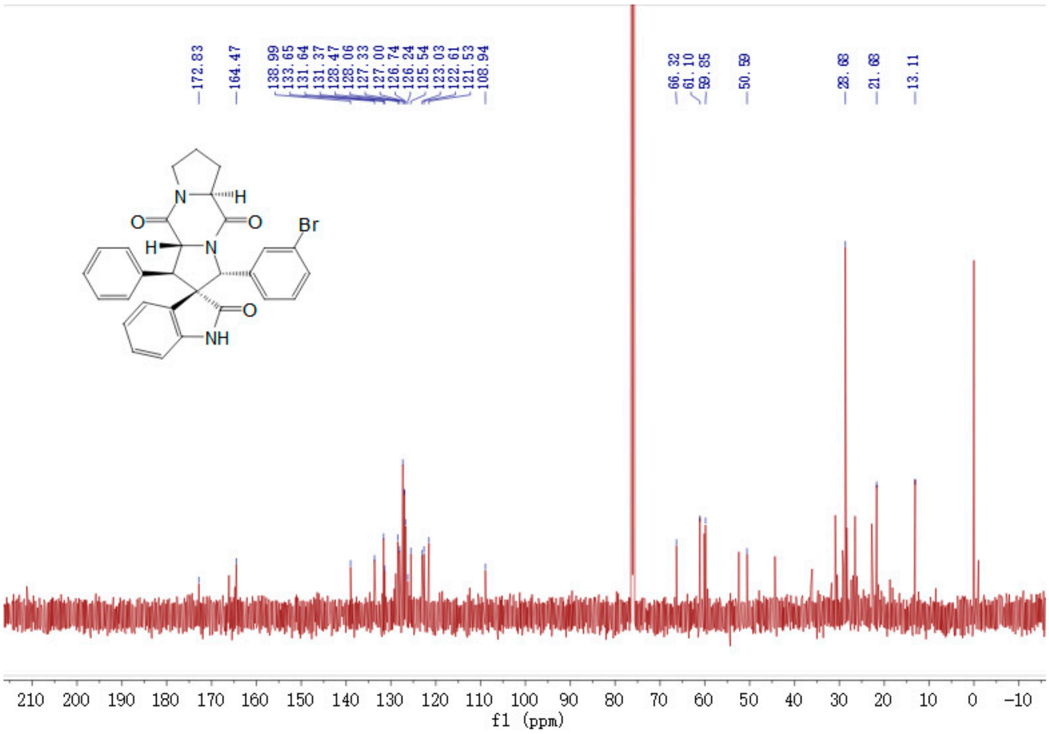

Fig. S155 The ^{13}C NMR (151 MHz, $CDCl_3$) of **4i**

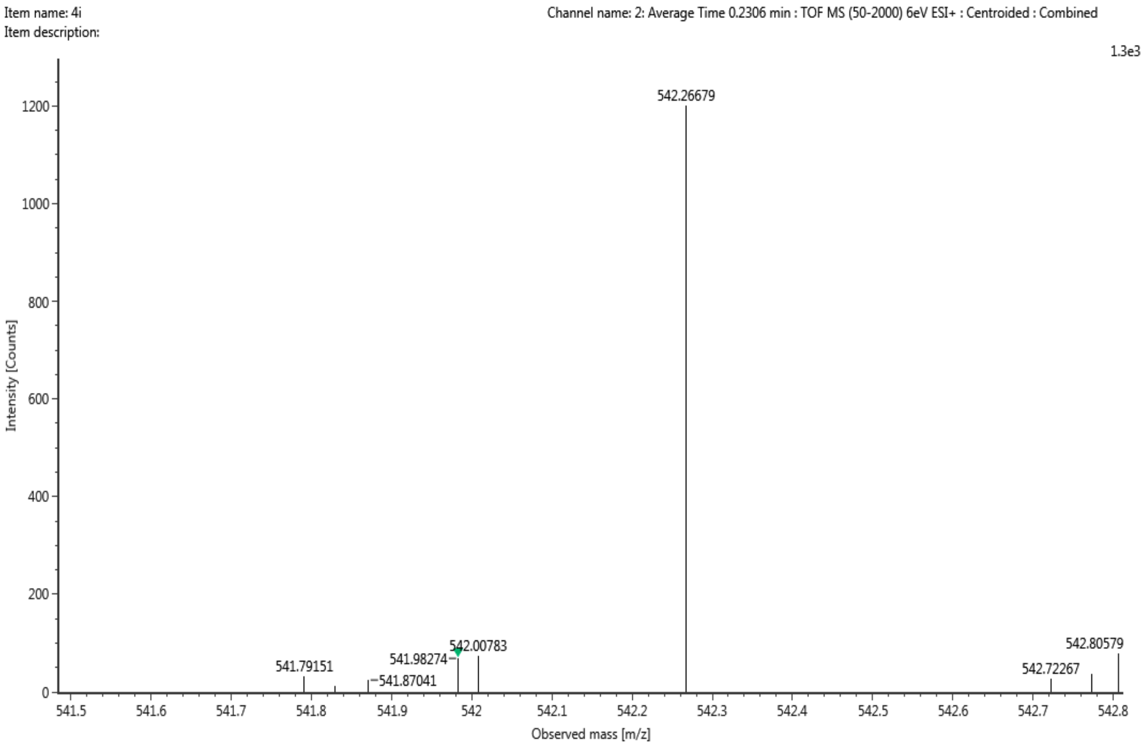

Fig. S156 The Mass spectrogram of **4i**

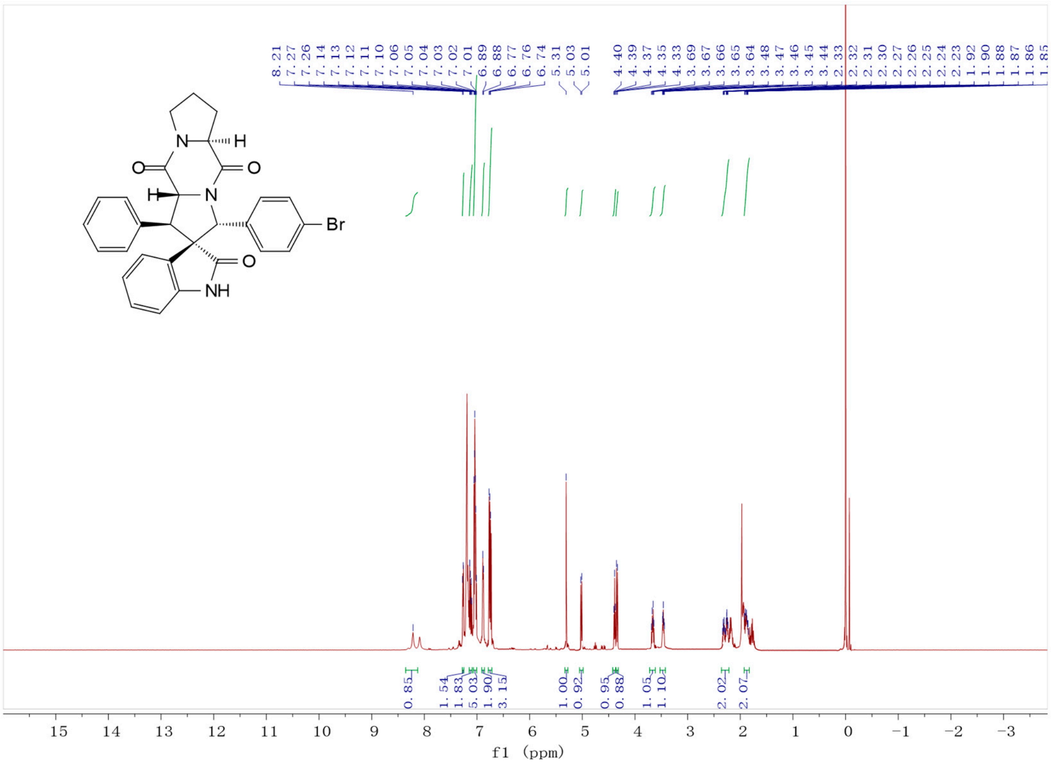

Fig. S157 The ^{1}H NMR (600 MHz, $CDCl_3$) of **4j**

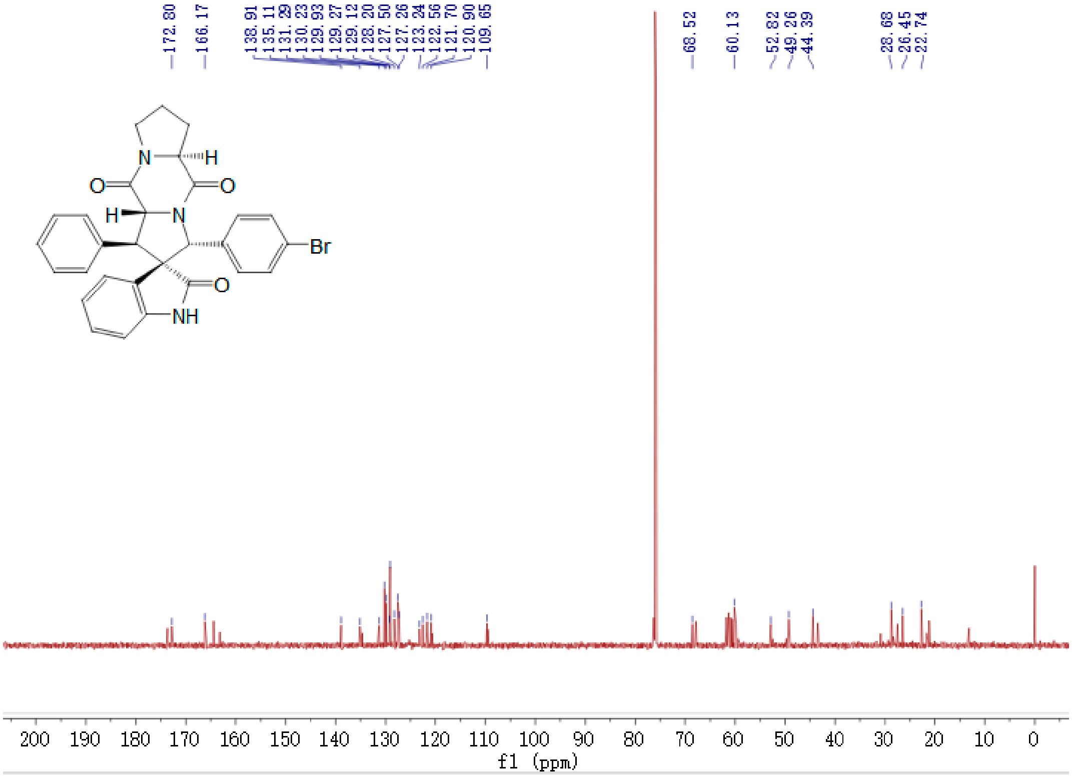

Fig. S158 The ^{13}C NMR (151 MHz, $CDCl_3$) of **4j**

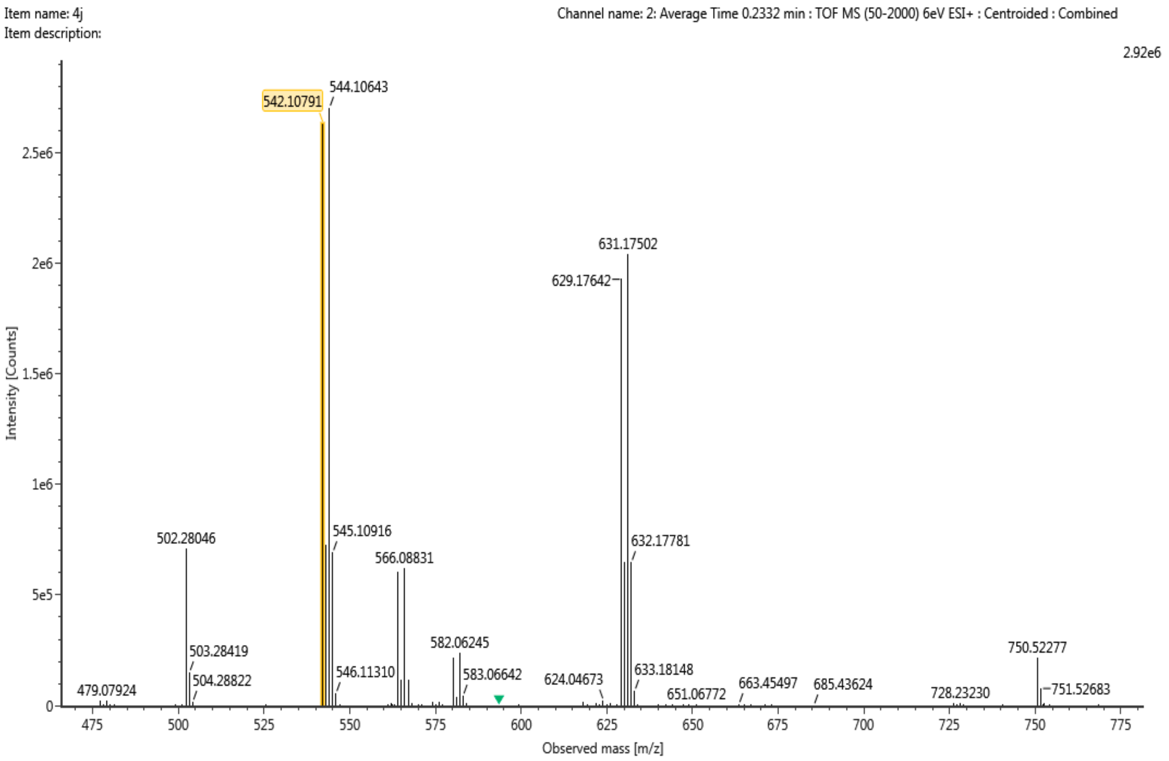

Fig. S159 The Mass spectrogram of **4j**

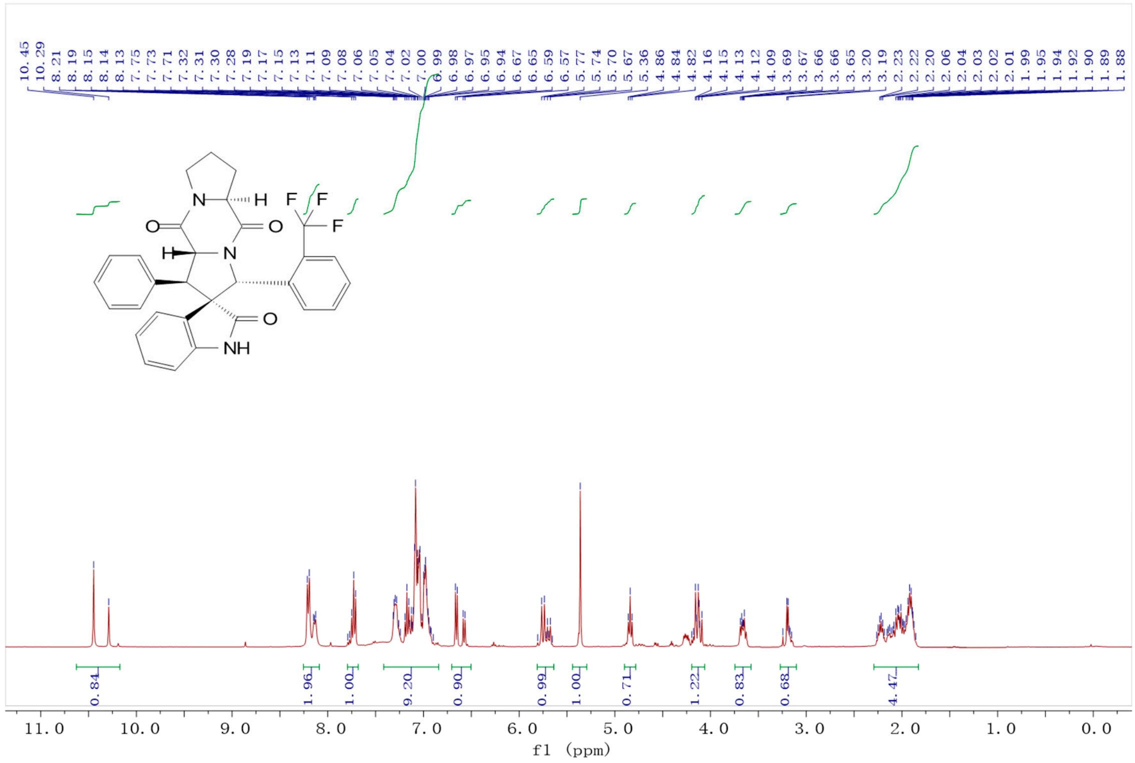

Fig. S160 The ^{1}H NMR (400 MHz, DMSO-d$_6$) of **4k**

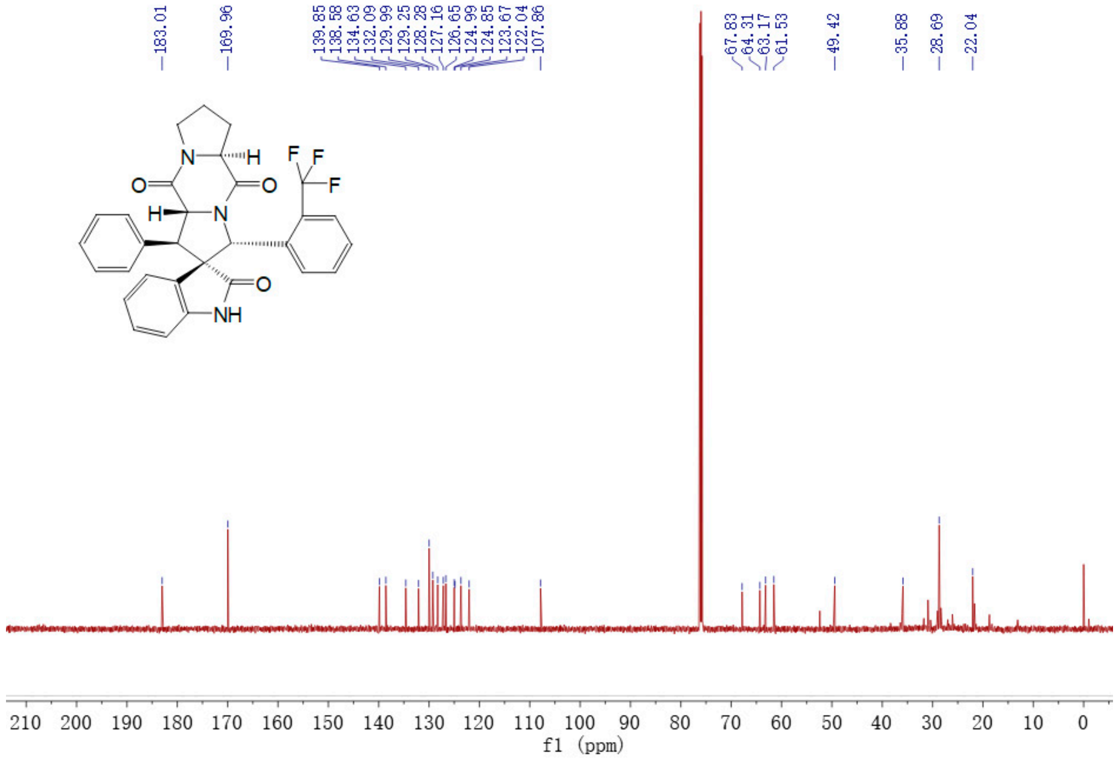

Fig. S161 The ^{13}C NMR (151 MHz, CDCl$_3$) of **4k**

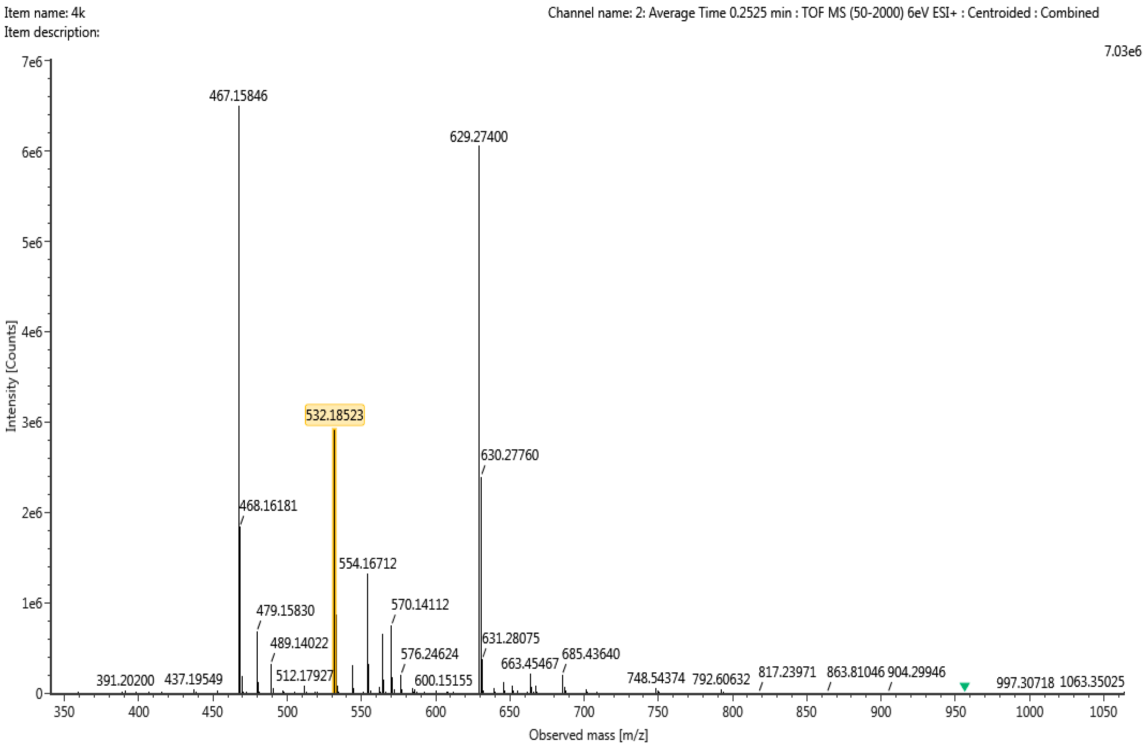

Fig. S162 The Mass spectrogram of **4k**

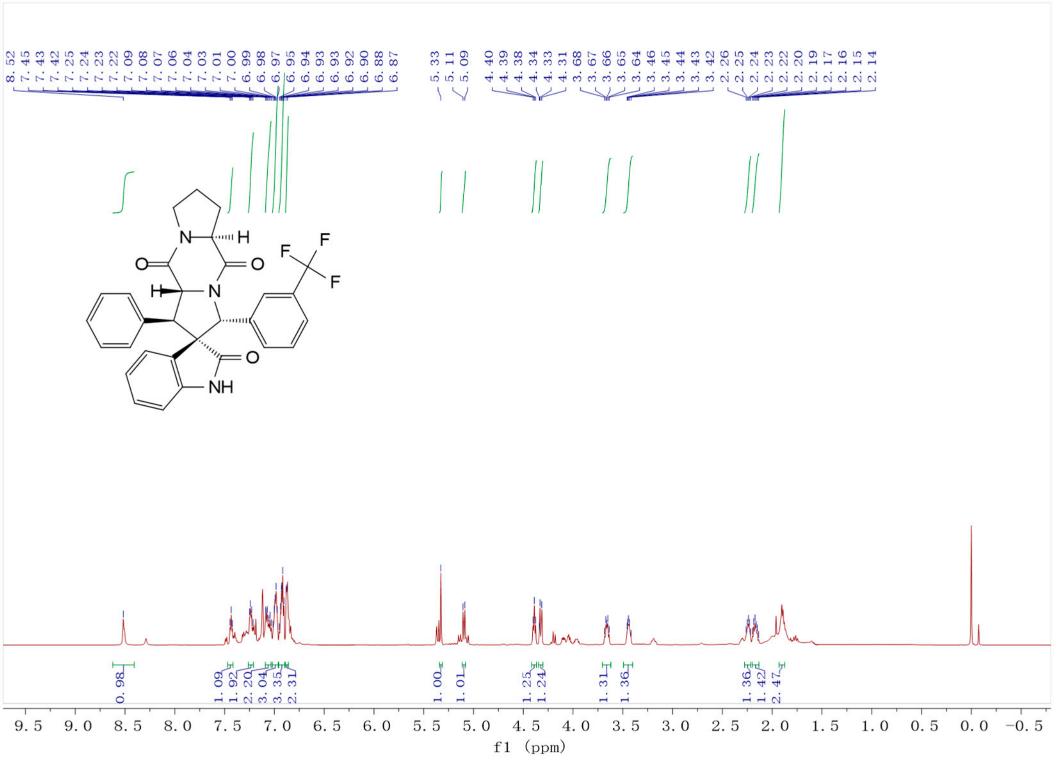

Fig. S163 The ^{1}H NMR (600 MHz, CDCl$_3$) of **4l**

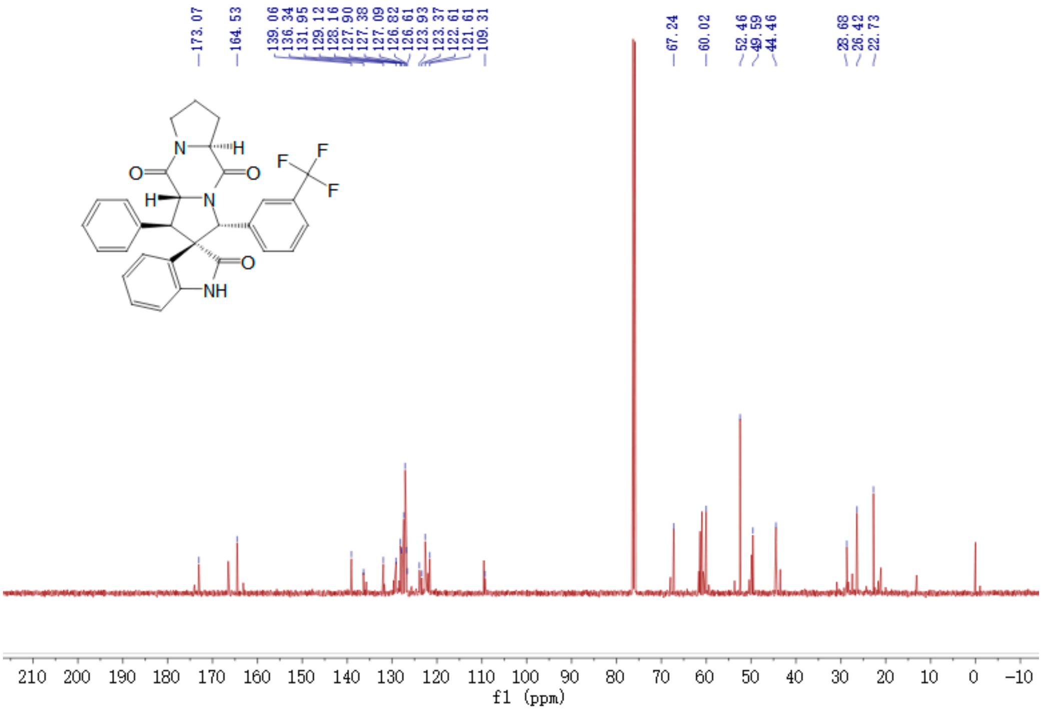

Fig. S164 The ^{13}C NMR (151 MHz, CDCl$_3$) of **4l**

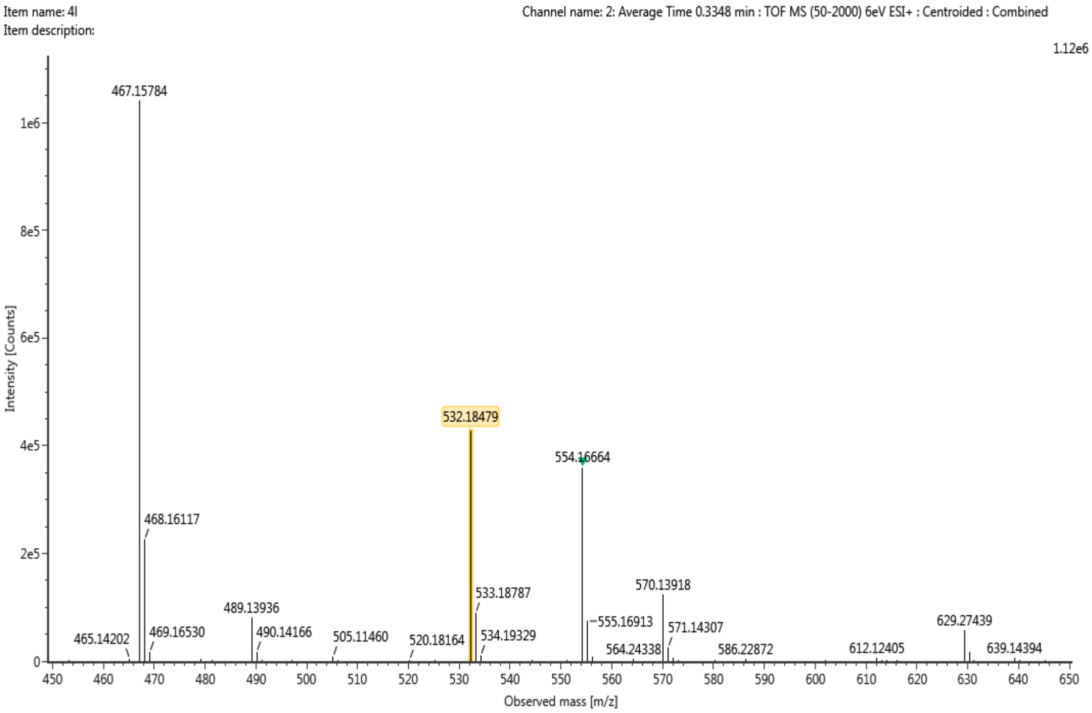

Fig. S165 The Mass spectrogram of **4l**

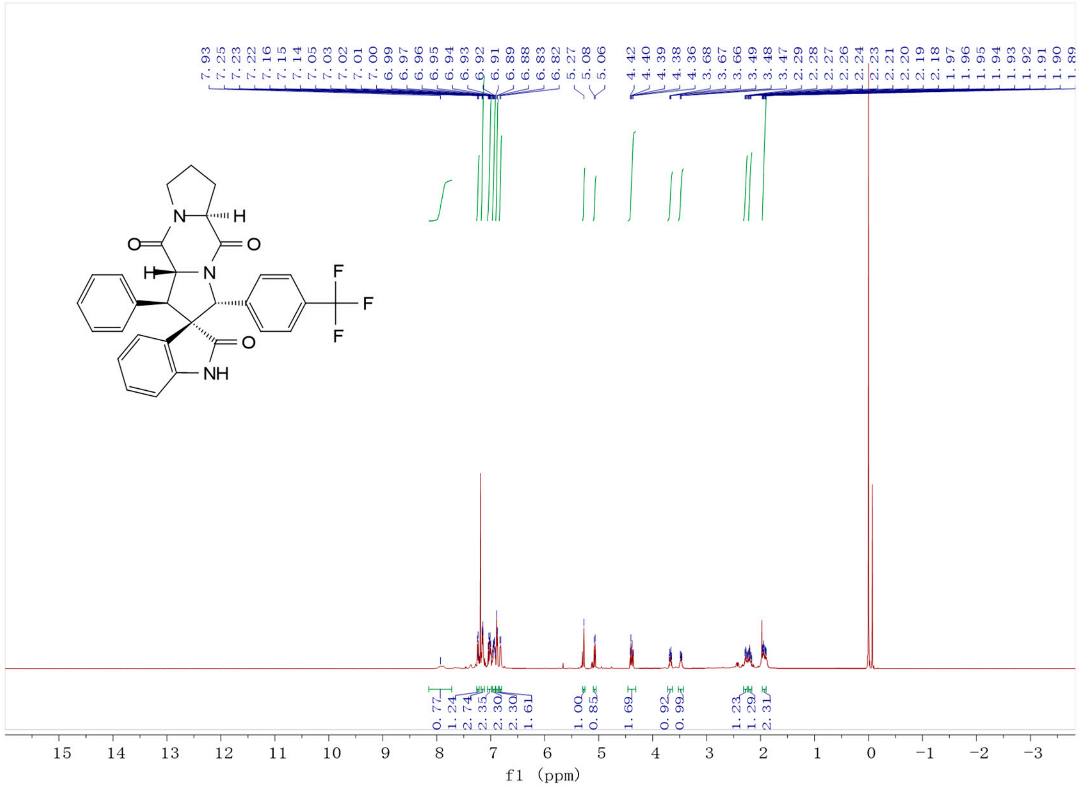

Fig. S166 The ^{1}H NMR (600 MHz, $CDCl_3$) of **4m**

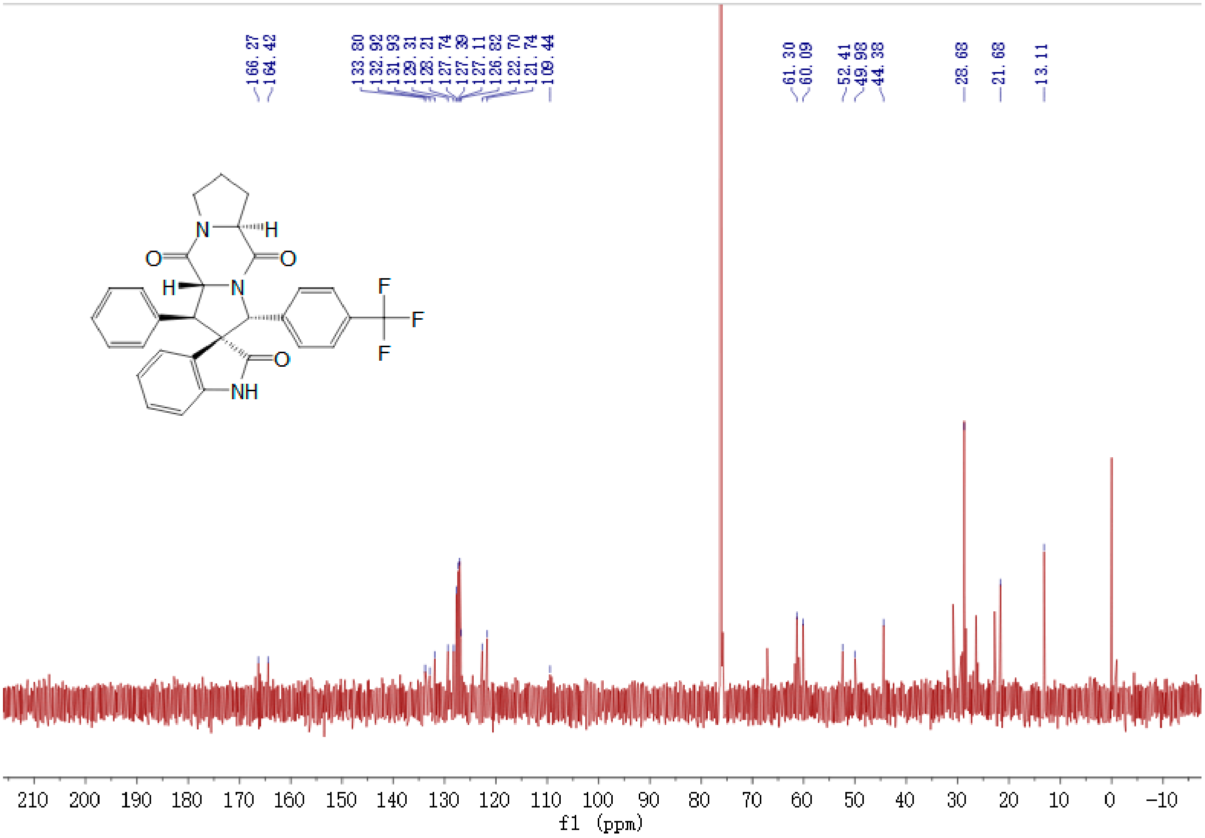

Fig. S167 The ^{13}C NMR (151 MHz, $CDCl_3$) of **4m**

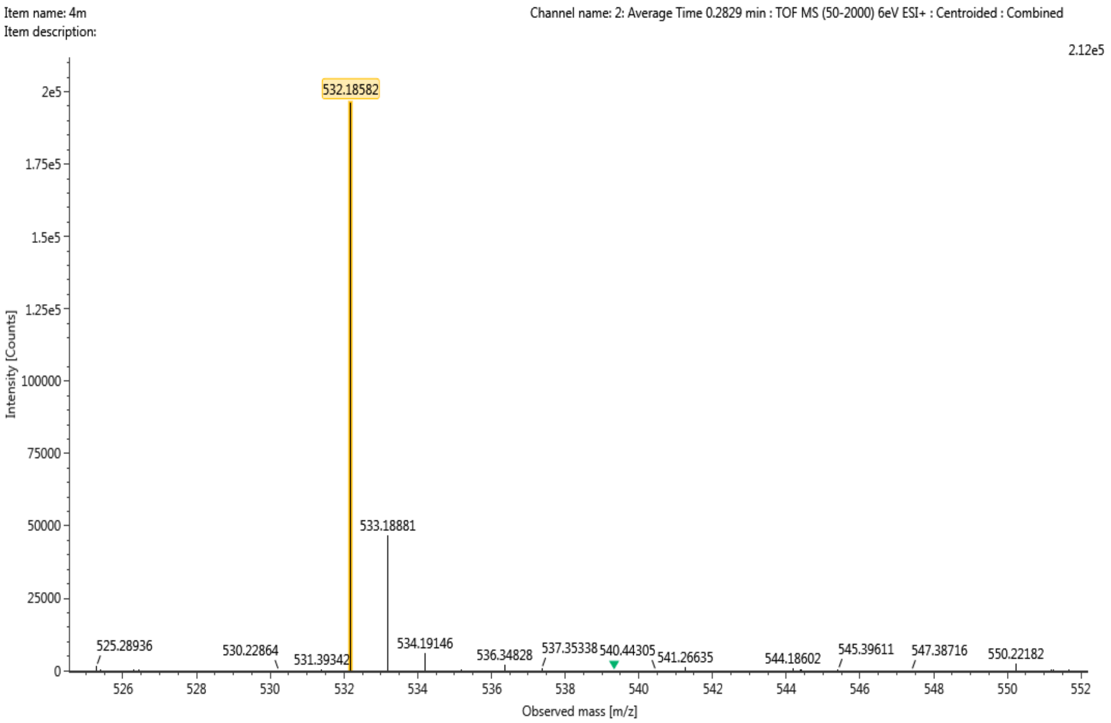

Fig. S168 The Mass spectrogram of **4m**

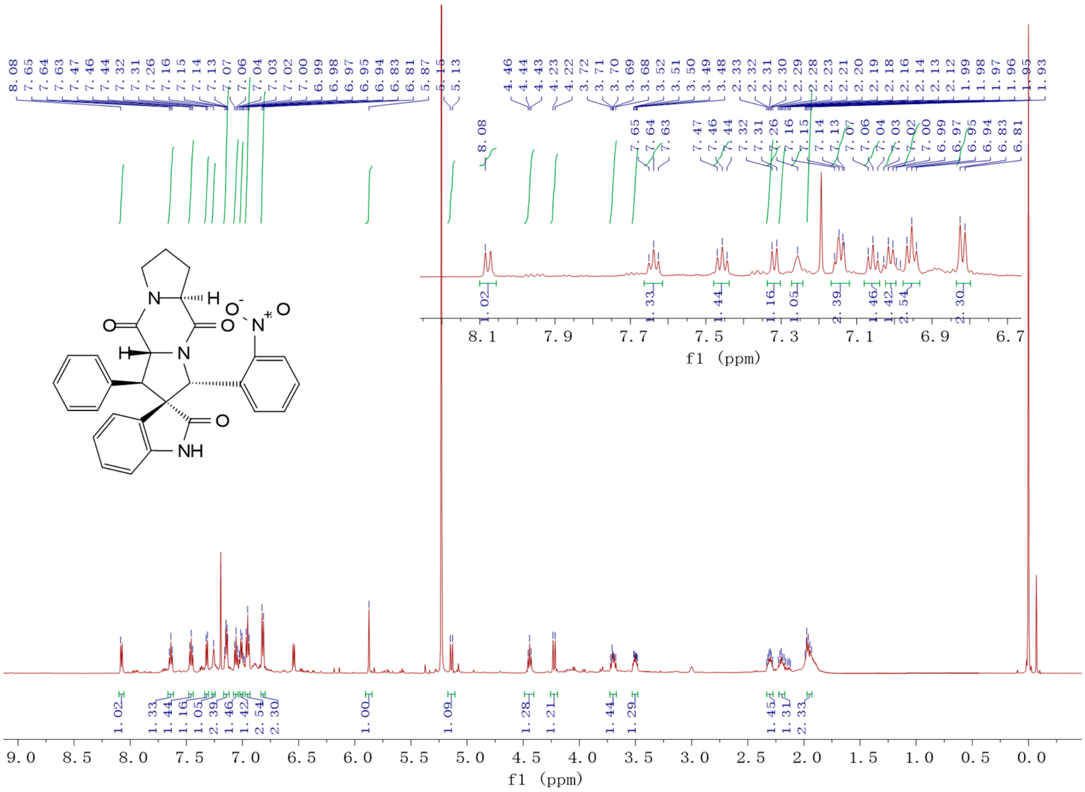

Fig. S169 The ^{1}H NMR (600 MHz, $CDCl_3$) of **4n**

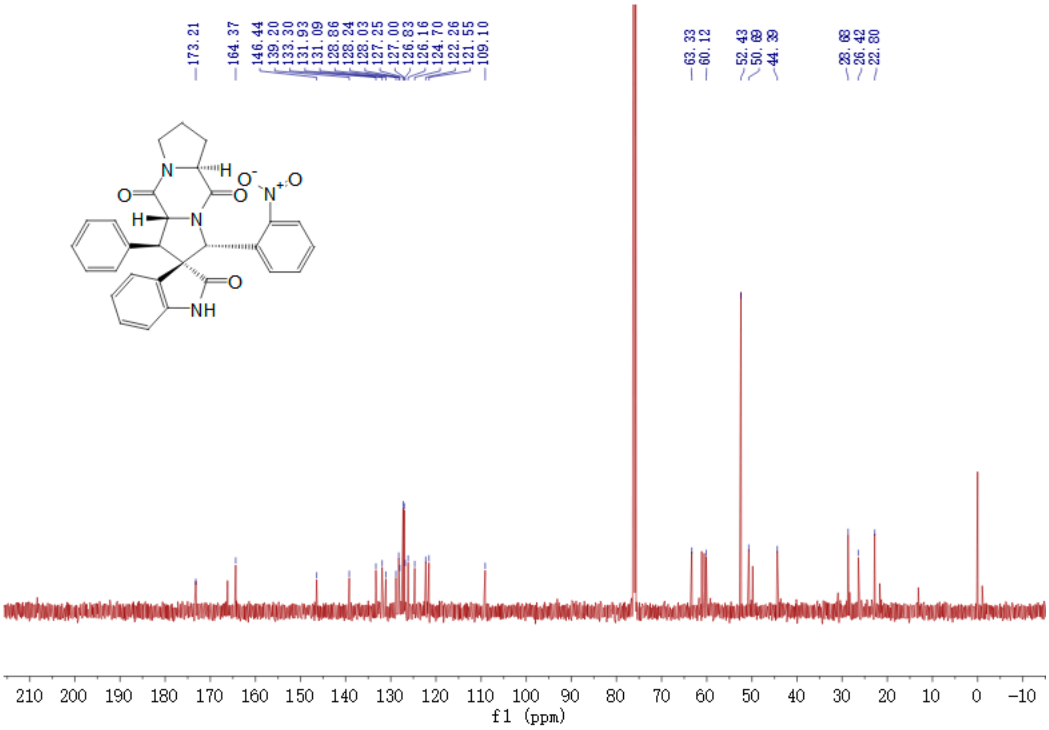

Fig. S170 The ^{13}C NMR (151 MHz, $CDCl_3$) of **4n**

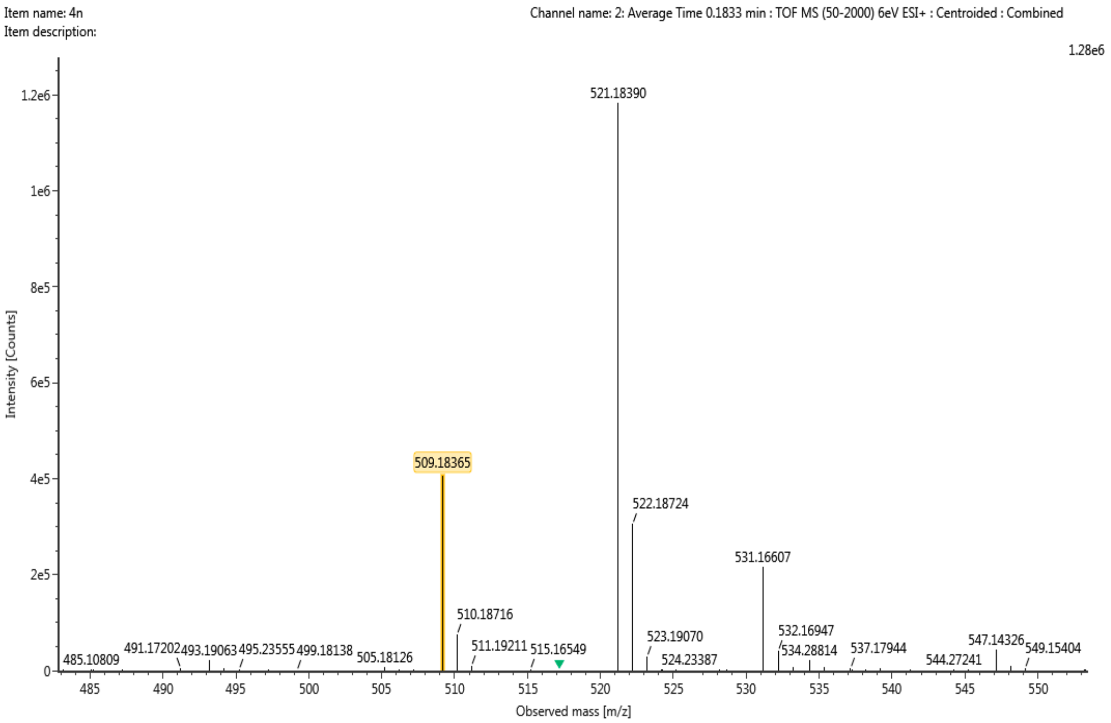

Fig. S171 The Mass spectrogram of **4n**

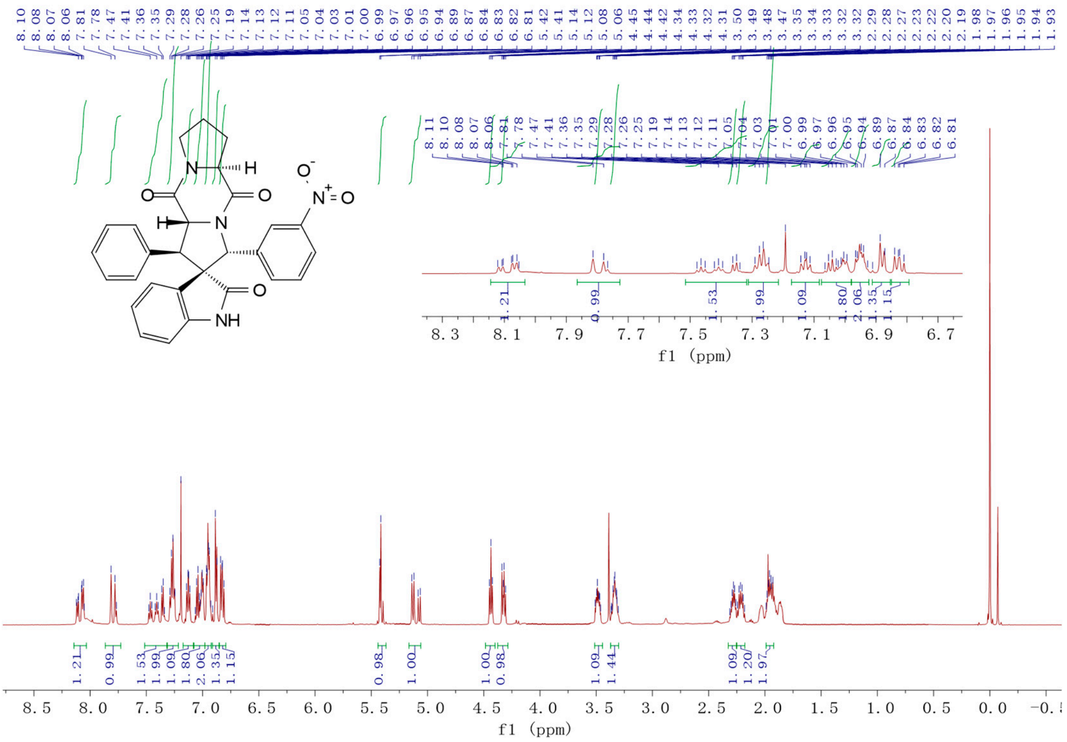

Fig. S172 The ^{1}H NMR (600 MHz, CDCl3) of **4o**

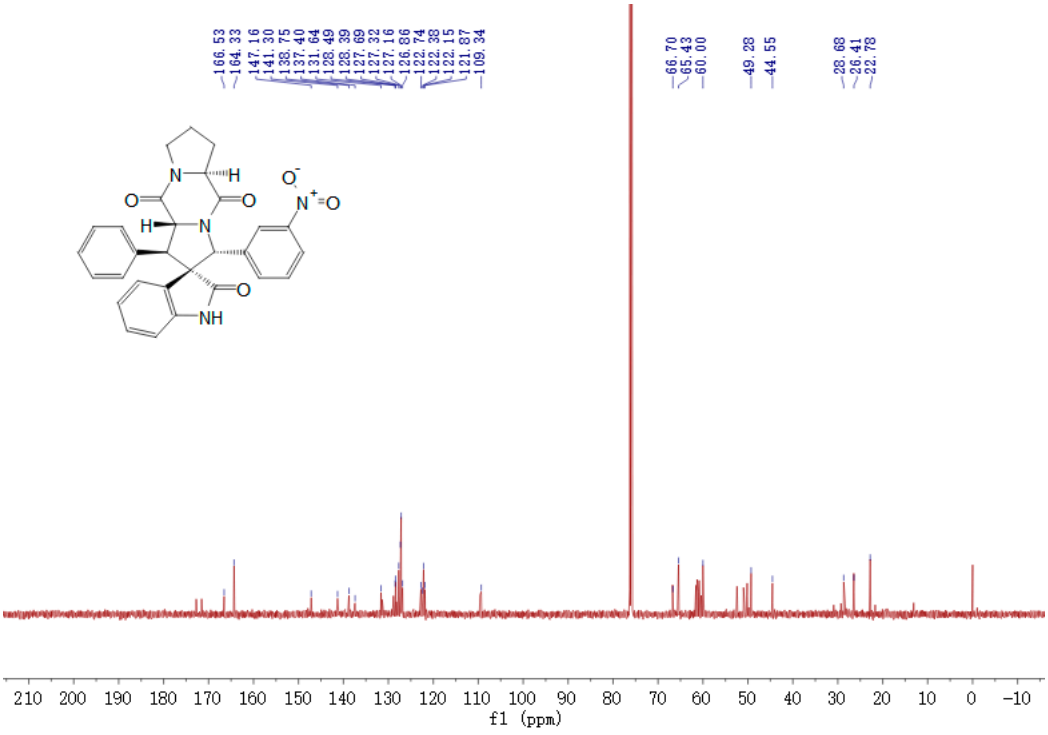

Fig. S173 The ^{13}C NMR (151 MHz, CDCl3) of **4o**

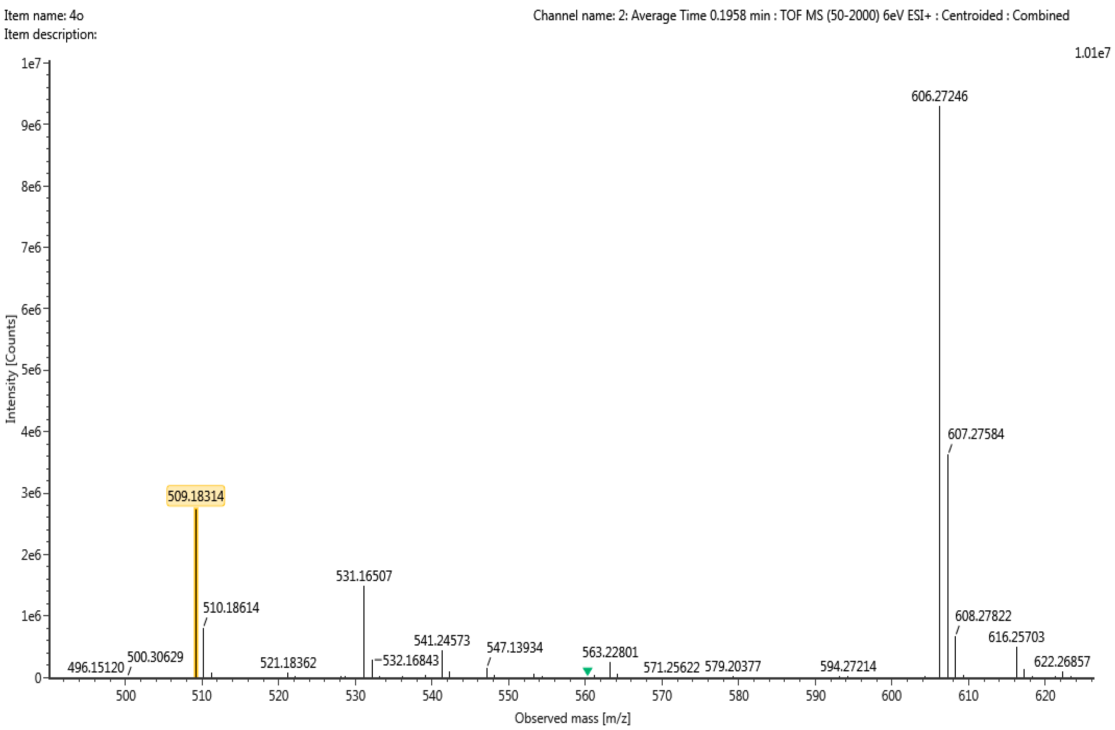

Fig. S174 The Mass spectrogram of **4o**

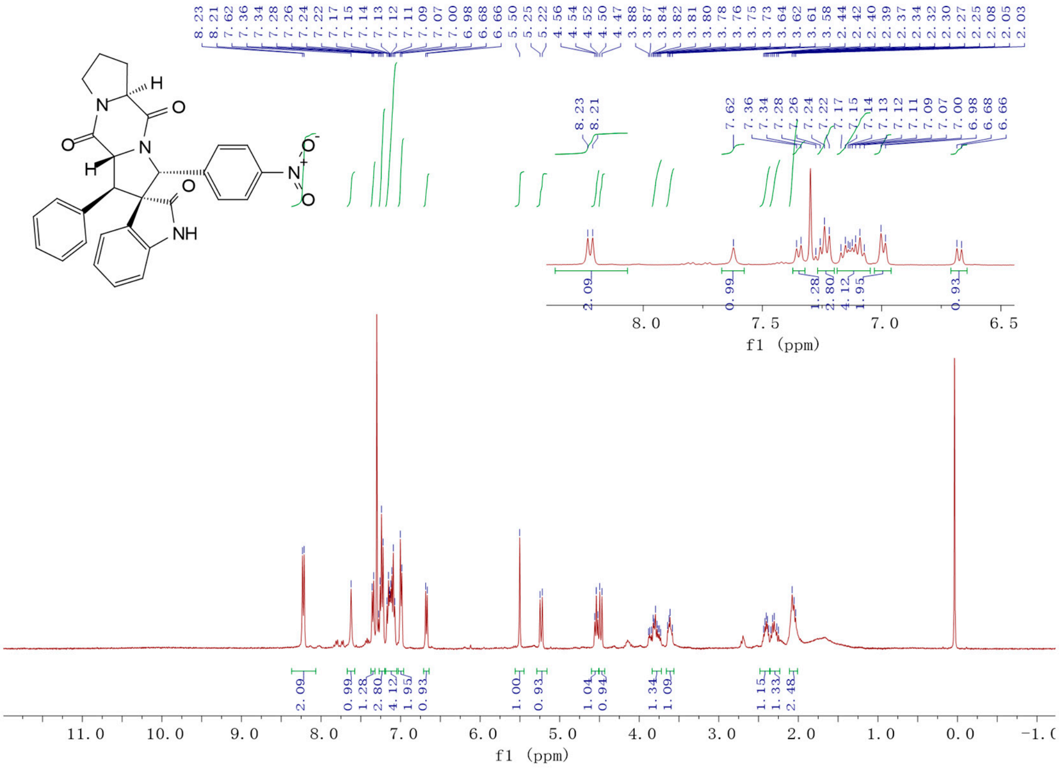

Fig. S175 The ^{1}H NMR (400 MHz, $CDCl_3$) of **4p**

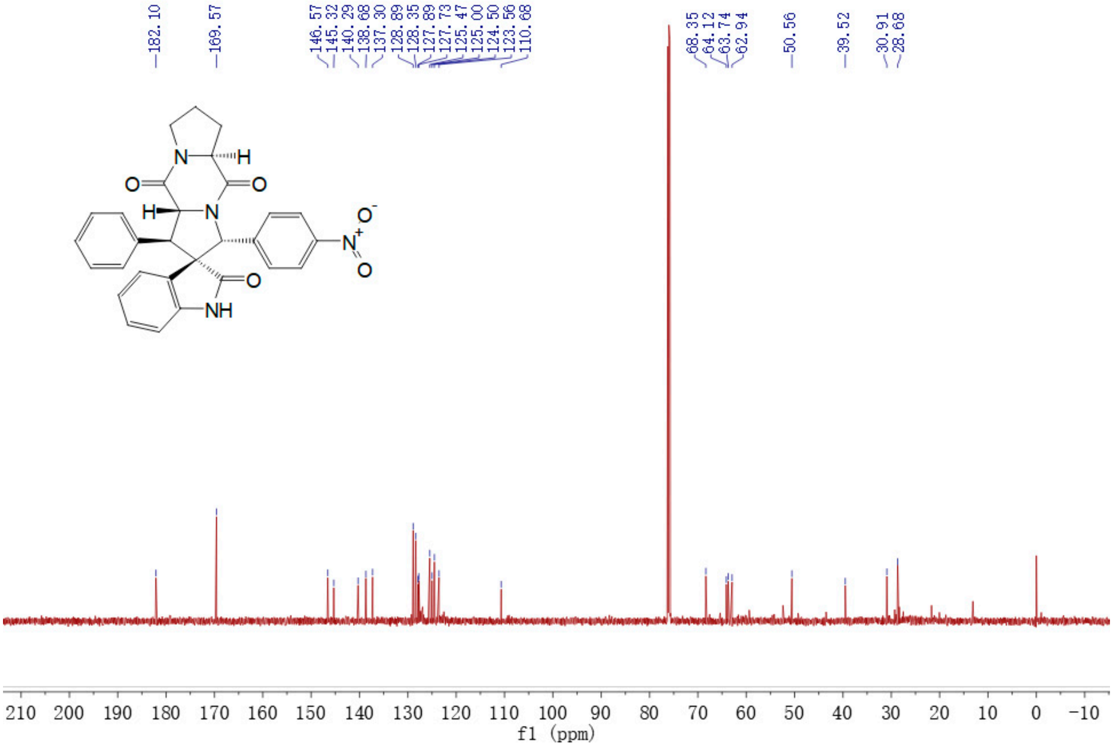

Fig. S176 The ^{13}C NMR (151 MHz, $CDCl_3$) of **4p**

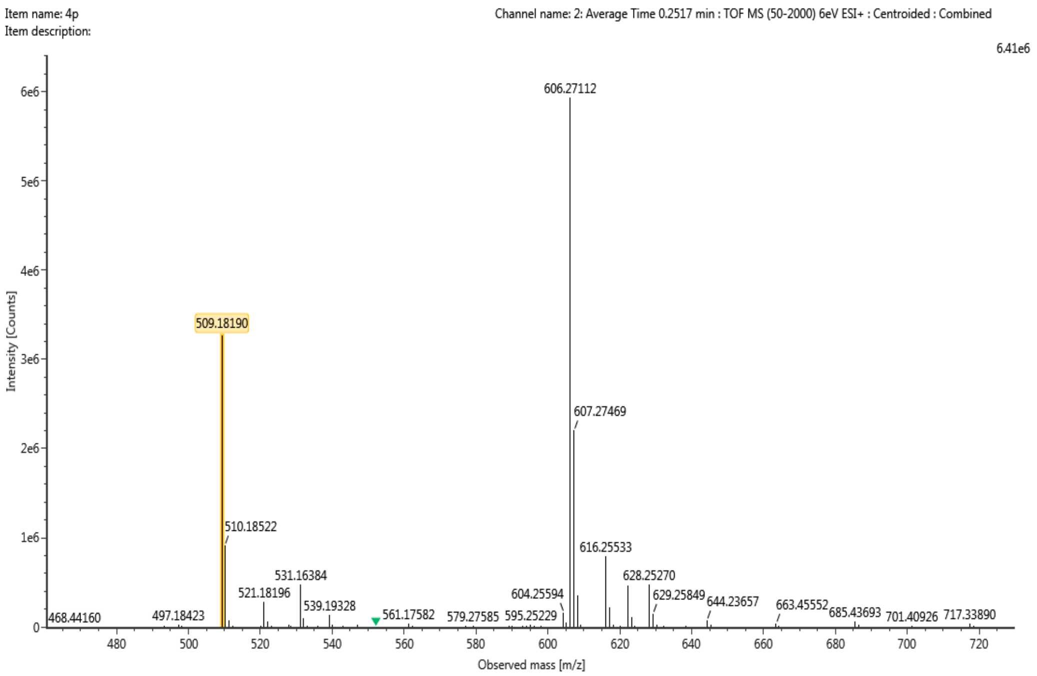

Fig. S177 The Mass spectrogram of **4p**

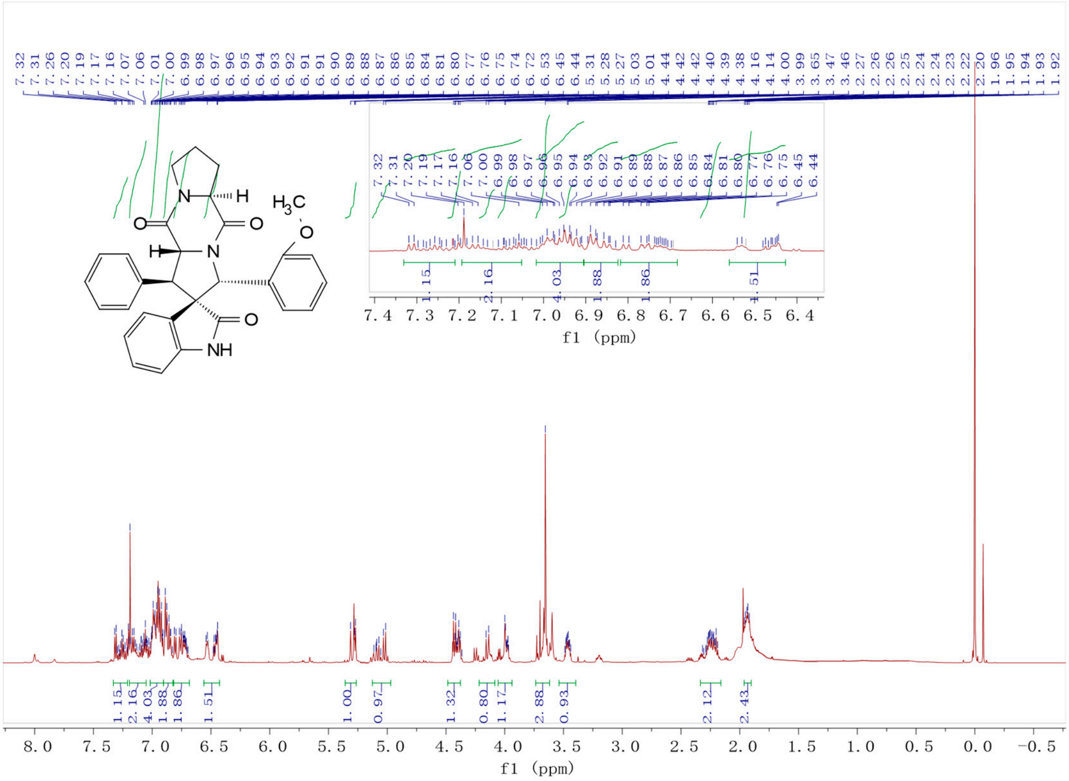

Fig. S178 The ^{1}H NMR (600 MHz, $CDCl_3$) of **4q**

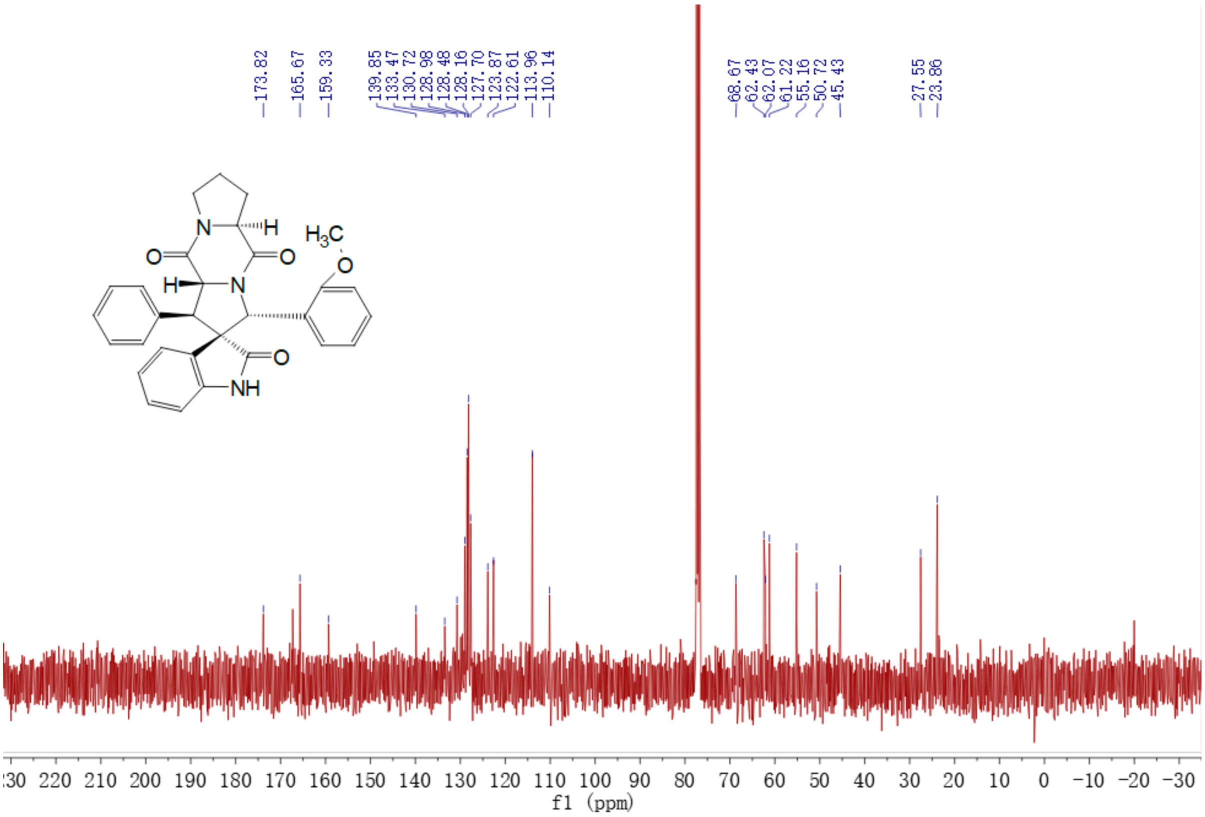

Fig. S179 The ^{13}C NMR (101 MHz, $CDCl_3$) of **4q**

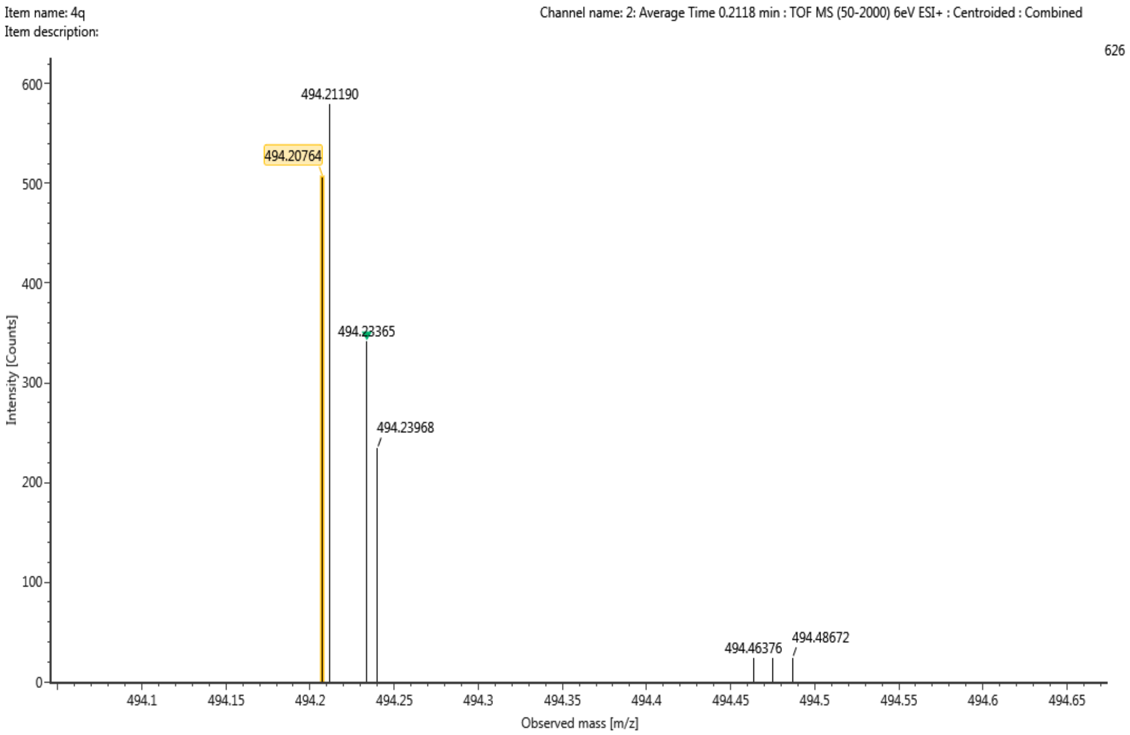

Fig. S180 The Mass spectrogram of **4q**

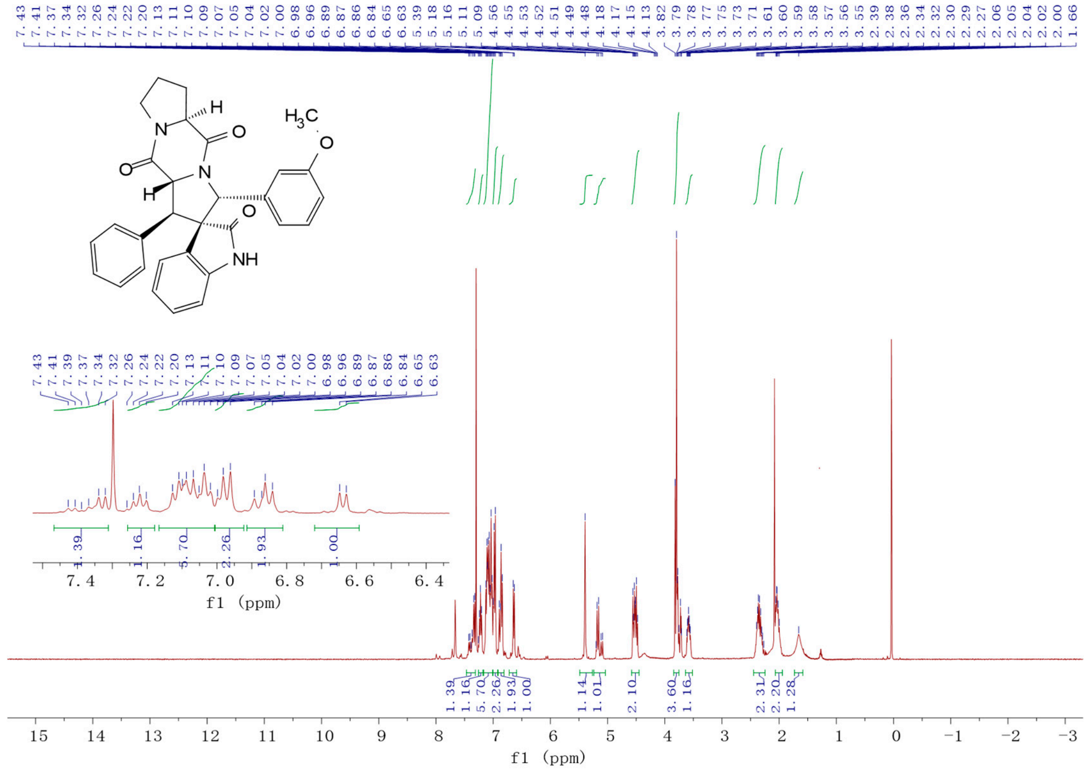

Fig. S181 The ^{1}H NMR (400 MHz, CDCl3) of **4r**

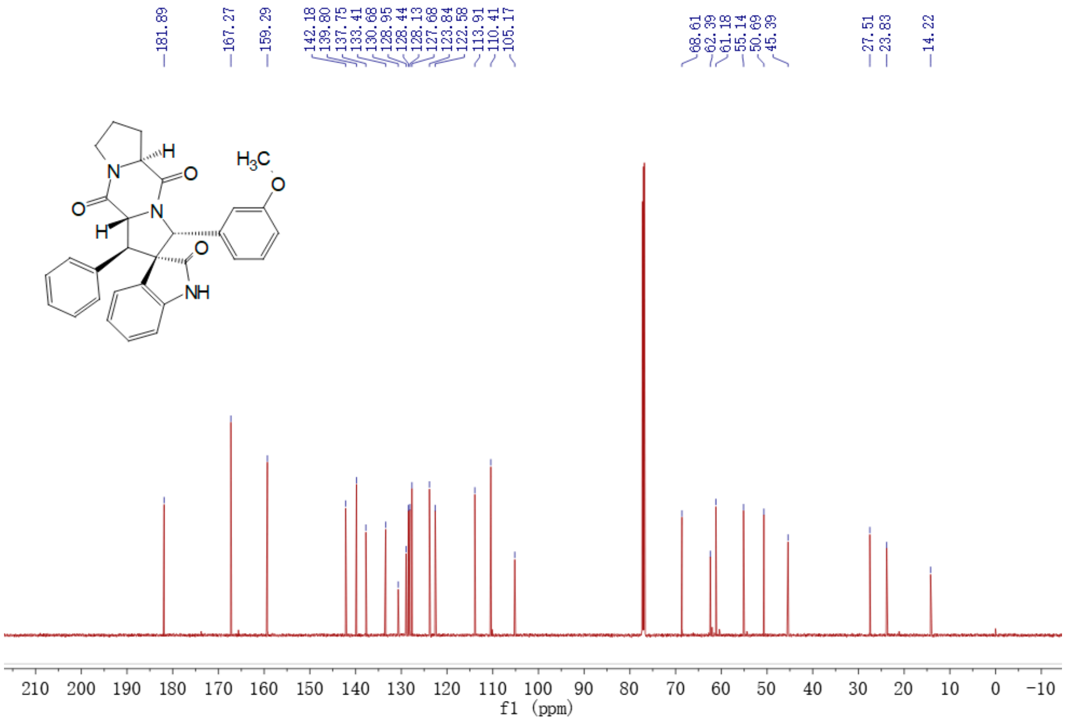

Fig. S182 The ^{13}C NMR (151 MHz, CDCl3) of **4r**

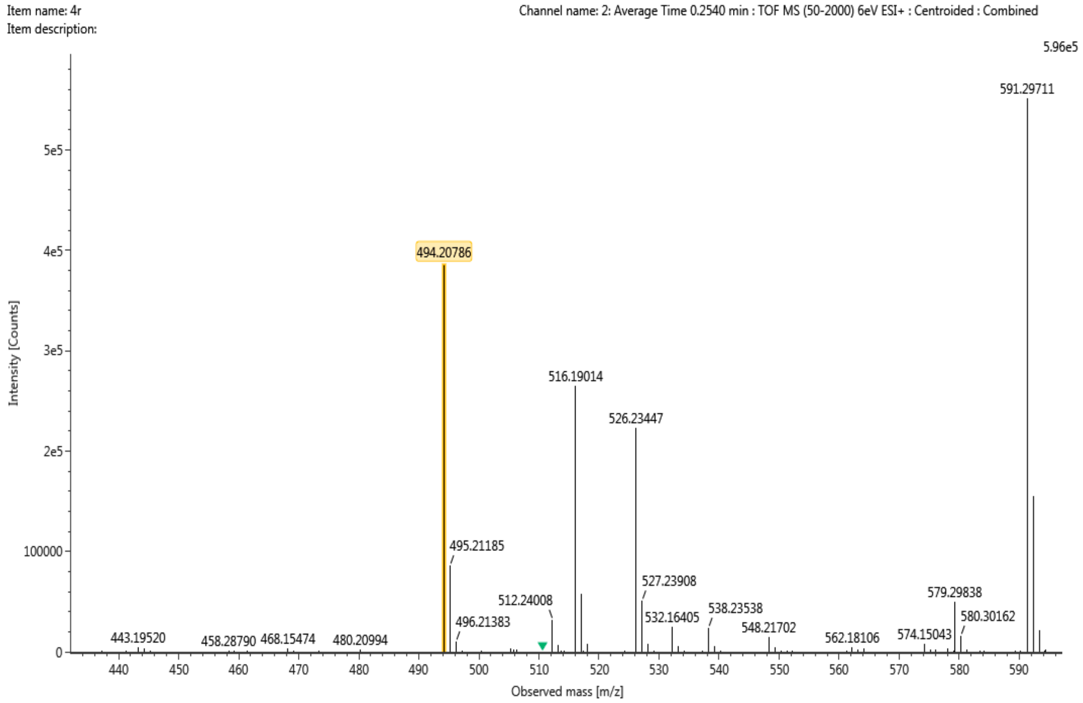

Fig. S183 The Mass spectrogram of **4r**

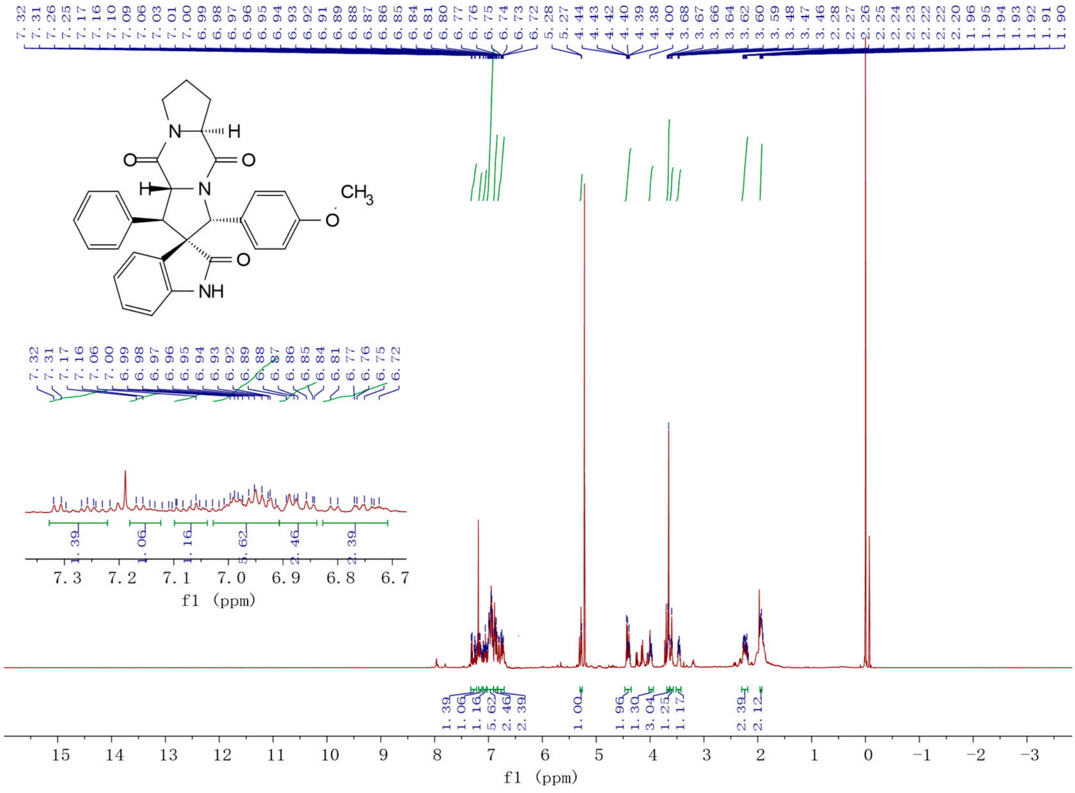

Fig. S184 The ^{1}H NMR (600 MHz, CDCl3) of **4s**

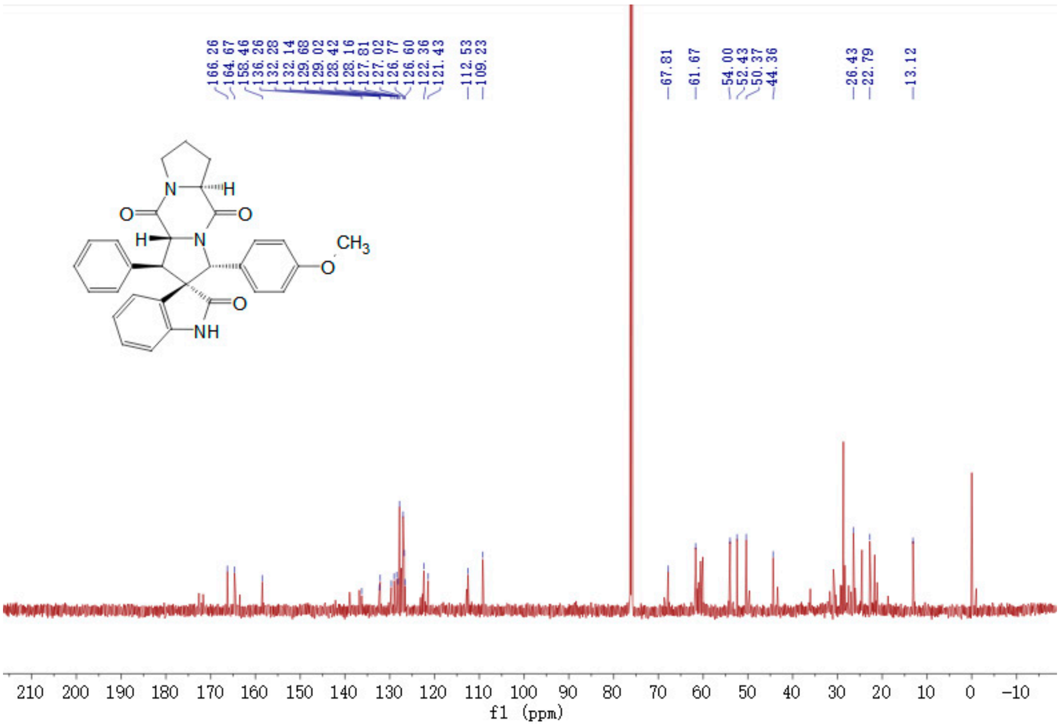

Fig. S185 The ^{13}C NMR (151 MHz, CDCl3) of **4s**

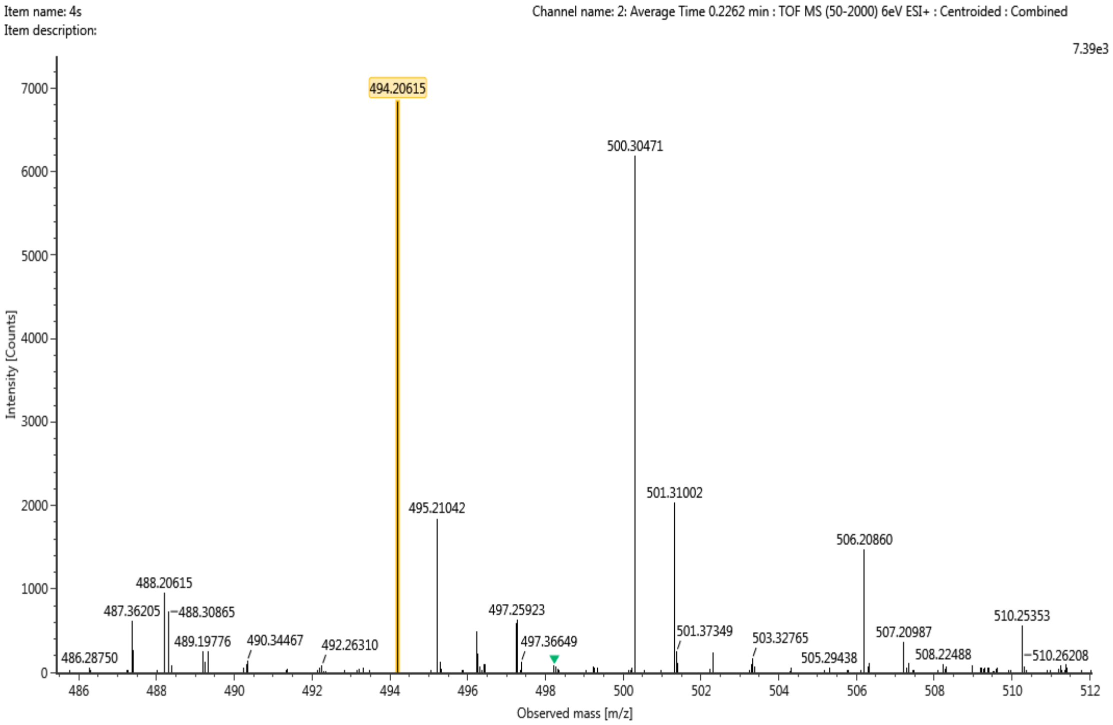

Fig. S186 The Mass spectrogram of **4s**

8. Crystallographic data

8.1 Crystallographic data for compound **3d**

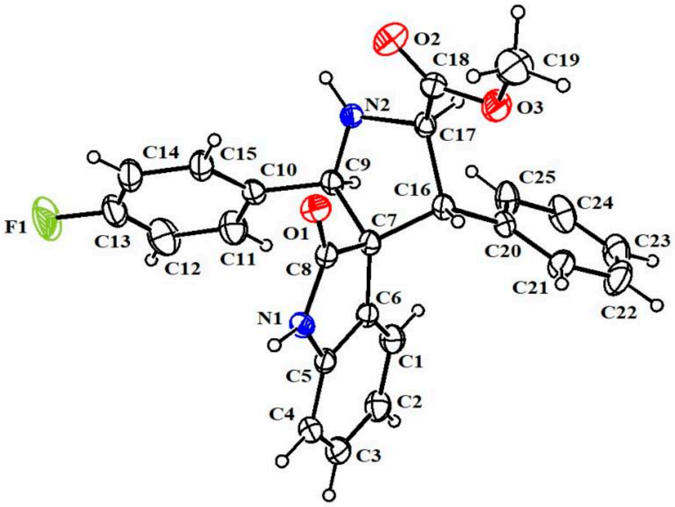

Table S6 Crystal data and structure refinement for **3d**

Identification code	3d
Empirical formula	$C_{25}H_{21}FN_2O_3$
Formula weight	416.44
Temperature	276(2) K
Wavelength	0.71073 A
Crystal system, space group	Monoclinic, P2(1)/c
Unit cell dimensions	a = 9.7134(3) A alpha = 90 deg.<br>b = 12.9290(4) A beta = 96.7990(10) deg.<br>c = 16.9669(6) A gamma = 90 deg.
Volume	2115.80(12) A^3
Z, Calculated density	4, 1.307 Mg/m^3
Absorption coefficient	0.092 mm^-1
F(000)	872
Crystal size	0.130 x 0.120 x 0.110 mm
Theta range for data collection	2.635 to 27.528 deg.
Limiting indices	-12<=h<=12, -16<=k<=16, -22<=l<=22
Reflections collected / unique	53863 / 4865 [R(int) = 0.1153]
Completeness to theta = 25.242	99.90%
Absorption correction	None
Refinement method	Full-matrix least-squares on F^2
Data / restraints / parameters	4865 / 0 / 281
Goodness-of-fit on F^2	1.049
Final R indices [I>2sigma(I)]	R1 = 0.0522, wR2 = 0.1347
R indices (all data)	R1 = 0.0921, wR2 = 0.1539
Extinction coefficient	n/a
Largest diff. peak and hole	0.504 and -0.442 e.A^-3

8.2 Crystallographic data for compound **3x**

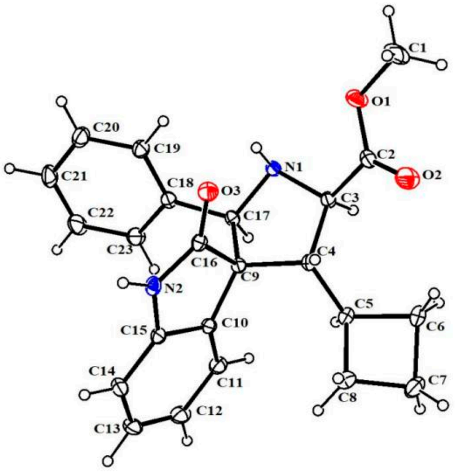

Table S7 Crystal data and structure refinement for **3x**.

Identification code	3x
Empirical formula	$C_{23}H_{24}N_2O_3$
Formula weight	376.44
Temperature/K	193
Crystal system	monoclinic
Space group	Cc
a/Å	15.8616(7)
b/Å	11.7953(7)
c/Å	12.2833(6)
α/°	90
β/°	116.743(2)
γ/°	90
Volume/Å^3	2052.29(18)
Z	4
ρ_{calc}g/cm^3	1.218
μ/mm^{-1}	0.651
F(000)	800
Crystal size/mm^3	0.13 × 0.12 × 0.1
Radiation	CuKα (λ = 1.54178)
2Θ range for data collection/°	9.758 to 159.384
Index ranges	$-18 \leq h \leq 20$, $-14 \leq k \leq 13$, $-15 \leq l \leq 15$
Reflections collected	18629
Independent reflections	4077 [R_{int} = 0.0316, R_{sigma} = 0.0278]
Data/restraints/parameters	4077/2/258
Goodness-of-fit on F^2	1.082
Final R indexes [I>=2σ (I)]	R_1 = 0.0352, wR_2 = 0.0887
Final R indexes [all data]	R_1 = 0.0354, wR_2 = 0.0890
Largest diff. peak/hole / e Å^{-3}	0.30/-0.20
Flack parameter	0.24(3)
